# Adaptive genetic differentiation in *Pterocarya stenoptera* (Juglandaceae) driven by multiple environmental variables were revealed by landscape genomics

**DOI:** 10.1186/s12870-018-1524-x

**Published:** 2018-11-27

**Authors:** Jia-Xin Li, Xiu-Hong Zhu, Yong Li, Ying Liu, Zhi-Hao Qian, Xue-Xia Zhang, Yue Sun, Liu-Yang Ji

**Affiliations:** 1 Innovation Platform of Molecular Biology, College of Forestry, Henan Agricultural University, No.95, Wenhua Road, Zhengzhou, 450002 China; 2 Guangdong Provincial Key Laboratory of Plant Resources, School of Life Sciences, Sun Yat-Sen University, No.135, Xingang Xi Road, Guangzhou, 510275 China

**Keywords:** Adaptive genetic differentiation, Environment-associated loci, Genome scans, Landscape genomics, *Pterocarya stenoptera*

## Abstract

**Background:**

The investigation of the genetic basis of local adaptation in non-model species is an interesting focus of evolutionary biologists and molecular ecologists. Identifying these adaptive genetic variabilities on the genome responsible can provide insight into the genetic mechanism of local adaptation.

**Results:**

We investigated the spatial distribution of genetic variation in 22 natural populations of *Pterocarya stenoptera* across its distribution area in China to provide insights into the complex interplay between multiple environmental variables and adaptive genetic differentiation. The Bayesian analysis of population structure showed that the 22 populations of *P. stenoptera* were subdivided into two groups. Redundancy analysis demonstrated that this genetic differentiation was caused by the divergent selection of environmental difference. A total of 44 outlier loci were mutually identified by Arlequin and BayeScan, 43 of which were environment-associated loci (EAL). The results of latent factor mixed model analysis showed that solar radiation in June (Sr6), minimum temperature of the coldest month (Bio6), temperature seasonality (Bio4), and water vapor pressure in January (Wvp1) were associated with the highest numbers of EAL. Sr6 was associated with the ecological habitat of “prefered light”, and Bio6 and Wvp1 were associated with the ecological habitat of “warm and humid environment”.

**Conclusions:**

Our results provided empirical evidence that environmental variables related to the ecological habitats of species play key roles in driving adaptive differentiation of species genome.

**Electronic supplementary material:**

The online version of this article (10.1186/s12870-018-1524-x) contains supplementary material, which is available to authorized users.

## Background

Recently, the investigation of the genetic basis of local adaptation in non-model species has become an interesting focus of evolutionary biologists and molecular ecologists [1, 2]. Locally adapted species are facing selection pressures from temporal climate fluctuations and spatial environment heterogeneity. In response to these selective pressures, species will undergo adaptive changes in phenotypes and phenology [3]. Behind these phenotypic and phenological changes is the adaptive differentiation of genes on the genome. Genome scans enable us to identify these adaptive genes responsible for local adaptation using population genetic analyses. Identifying the genes that control these phenotypic and phenological changes can provide insight into the genetic mechanism of local adaptation [4]. However, the identification of these adaptive genes on the genome responsible for local adaptation remains a great challenge for most non-model species due to the lack of genomic information [5].

Landscape genomics was proposed by Joost et al. [6]. It has been used to uncover the relationship between adaptive genes on the genome and heterogeneous environment variables among natural populations of species [7]. Although most non-model species have no genomic information, molecular markers that do not need prior information and have high density coverage of the genome are suitable for landscape genomic studies of non-model species. Thus, amplified fragment length polymorphisms (AFLPs), inter-simple sequence repeats (ISSRs), and start codon targeted polymorphisms (SCoTs) all conform to the above conditions [4]. Simultaneously, these markers also have the advantages of high polymorphism and high repeatability. However, AFLP and ISSR are neutral molecular markers; the adaptive loci detected by these markers are more likely to be linked to adaptive genes. SCoT is a molecular marker developed based on the short conserved initial codon; it is a non-neutral bias marker that is more biased toward genes [8]. Therefore, it is more suitable for adaptive evolution research compared with the other two markers. In recent years, several reduced-representation genome sequencing (RRGS) technologies have been developed to improve the genome coverage density of molecular markers. These sequencing technologies include genotyping by sequencing [9], restricted site associated DNA [10], and specific-locus amplified fragment sequencing [11]. Although these markers yielded by the RRGS are different from the three molecular markers mentioned above, they contain DNA sequence information. Most of them cannot be annotated because they are in non-coding regions on the genome or the DNA sequence is too short and there is no whole-genome information. In any case, they can give some information about those genes that can be annotated. Recently, landscape genomics studies using these molecular markers have been carried out on many plant and animal species [4].

A large number of landscape genomic studies have proved that environmental variables would drive the adaptive differentiation of some loci on the genome of locally adapted species [12, 13]. However, we do not know why adaptive differentiation occurred in these genes and why environmental factors played key roles in driving adaptive differentiation. During local adaptation, different species in different regions have different adaptive differentiation genes and different driving factors. Are there common reasons behind these differences? Several recent landscape genomic studies have proposed a hypothesis that environmental variables related to ecological habitats play key roles in driving species adaptive evolution; in other words, the genes associated with these environmental variables will undergo adaptive differentiation [2, 14, 15]. More landscape genomic research is needed to test whether this hypothesis applies to other species as well. Another question worthy of discussion is whether divergent selection on the genome from environmental variables plays a decisive role on the spatial genetic structure of species. Given that previous surveys on species population structure used more neutral molecular markers, we pay more attention to the effects of population demographic history and gene flow on them [16]. If the whole genome markers are used, that is, both neutral and non-neutral markers, the genetic differentiation that driven by natural selection from environmental variables will be detected. Population demographic history, gene flow, and natural selection, which will have a greater impact on the spatial genetic structure of species? The answers to these questions will help deepen our understanding of adaptive evolution of species.

*Pterocarya stenoptera* C. DC (Juglandaceae) is a deciduous broad-leaved tree growing in forests below 1500 m above sea level along the stream bank or wet hillside land. It is widely distributed in warm temperate and subtropical zones of China. *Pterocarya stenoptera* prefers light, tolerates waterlogging, likes to grow in warm and humid environment, and can grow on acidic to slightly alkaline soil [17]. Here, 22 natural populations of *P. stenoptera* across its distribution region in China were sampled to investigate the relationship between adaptive genetic variations on the genome and environmental variables by using landscape genomic approach.

In this study, we employed SCoT markers to scan the genome of *P. stenoptera* and identified the adaptive loci by performing correlations between local environmental variables and selected SCoT alleles. The present study aimed to (i) identify the spatial genetic structure of *P. stenoptera*, (ii) evaluate the role of environmental variations on the spatial genetic structure of *P. stenoptera*, and (iii) examine the effects of environmental variables on adaptive differentiation of *P. stenoptera* genome.

## Results

### Population genetic structure

A total of 510 individuals of *P. stenoptera* from 22 wild populations were successfully scored using the 9 SCoT primers, and 1006 unambiguous fragments were identified with sizes varying from 100 bp to 1200 bp. The number of alleles in 9 primers ranged from 53 (SCoT35) to 156 (SCoT25). The lowest number and percentage of polymorphic alleles (*N*_A_ = 126, *PPA* = 12.5) were found in AHXN (P5) population and the highest (*N*_A_ = 261, *PPA* = 25.9) in JSBH (P16) population. Nei’s genetic diversity (*H*_E_) per population varied from 0.0459 in AHXN (P5) to 0.083 in HNTM (P20). Overall, the summary statistics of the genetic diversity analyses of 22 populations of *P. stenoptera* are shown in Table 1.

**Table 1:** Details of population locations, sample size, genetic diversity of 22 populations for *Pterocarya stenoptera*

Population no. and code	Locations	Altitude meters)	Lat.N)/ Long.E)	*N*	*N*_A_	*PPA*	*H*_E_
Group A							
1. JSLM	Lang Mt., Jiangshu	53	31.95/ 120.89	22	181	18.0	0.072
2. SCWD	Wuduzhen, Sichuan	532	31.88/ 104.78	24	178	17.7	0.070
3. HNJG	Jigong Mt., Henan	418	31.81/114.08	23	170	16.9	0.069
4. AHTZ	Tianzhu Mt., Anhui	39	30.67/ 116.49	24	171	17.0	0.058
5. ZJTM	Tianmu Mt., Zhejiang	202	30.28/ 119.46	22	183	18.2	0.061
6. AHXN	Xiuning, Anhui	155	29.78/ 118.17	24	126	12.5	0.045
7. SCEM	Emei Mt., Sichuan	533	29.57/ 103.44	23	156	15.5	0.058
8. JXSQ	Shanqing Mt., Jiangxi	171	28.84/ 118.04	23	153	15.2	0.048
9. JXLH	Longhu Mt., Jiangxi	47	28.12/ 116.97	24	180	17.9	0.062
10. GZFJ	Fengjing Mt., Guizhou	489	27.84/ 108.77	22	181	18.0	0.072
11. YNYB	Yangbi, Yunnan	1445	25.62/ 100.03	23	170	16.9	0.069
Group B							
12. SDTM	Tai Mt., Shandong	304	36.22/117.12	24	139	13.8	0.054
13. SDMM	Meng Mt., Shandong	323	35.56/117.96	24	156	15.5	0.062
14. HNNZ	Nanzhao, Henan	616	33.59/112.18	24	201	20.0	0.067
15. HNXC	Xichuan, Henan	383	33.28/111.12	20	216	21.5	0.079
16. SXWZ	Wuzi Mt., Shaanxi	408	32.95/ 107.84	21	204	20.3	0.078
17. JSBH	Baohua Mt., Jiangshu	189	32.14/119.09	24	261	25.9	0.077
18. HBSN	Shengnongjia, Hubei	657	31.37/ 110.50	24	194	19.3	0.070
19. HBJG	Jiugong Mt., Hubei	74	29.45/ 114.71	24	199	19.8	0.064
20. HNTM	Tianmeng Mt., Hunan	165	29.11/ 110.46	24	231	23.0	0.083
21. FJWY	Wuyi Mt., Fujian	185	27.65/ 117.97	24	178	17.7	0.065
22. HNHM	Heng Mt., Hunan	198	27.26/ 112.72	23	204	20.3	0.074

*N* number of individuals, *N*_A_ number of polymorphic alleles, *PPA* percentage of polymorphic alleles, *H*_E_ Nei’s 1973 measure of gene diversity

**Fig. 1:**
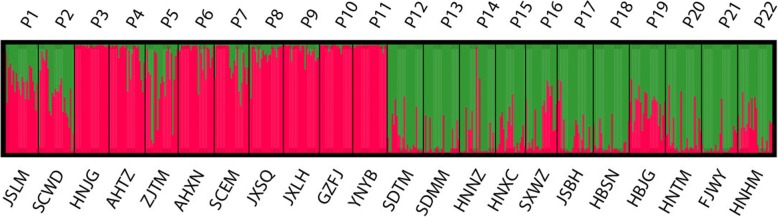
STRUCTURE analyses of 22 sampled populations of *Pterocarya stenoptera*. Each vertical bar shows the proportional representation of two genetic clusters (*K*) for an individual

**Fig. 2:**
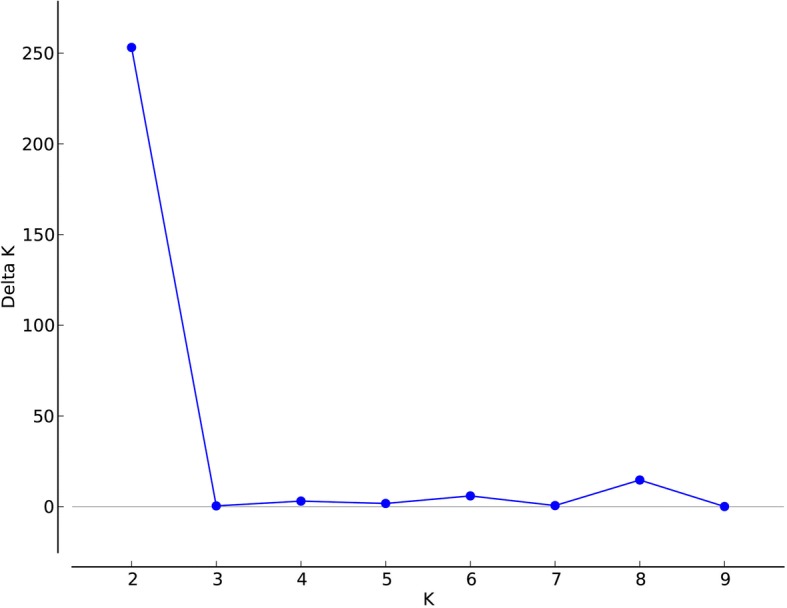
The uppermost hierarchical level of population genetic structure determined according to the values of *ΔK*. *ΔK* was calculated by Structure Harvester

**Fig. 3:**
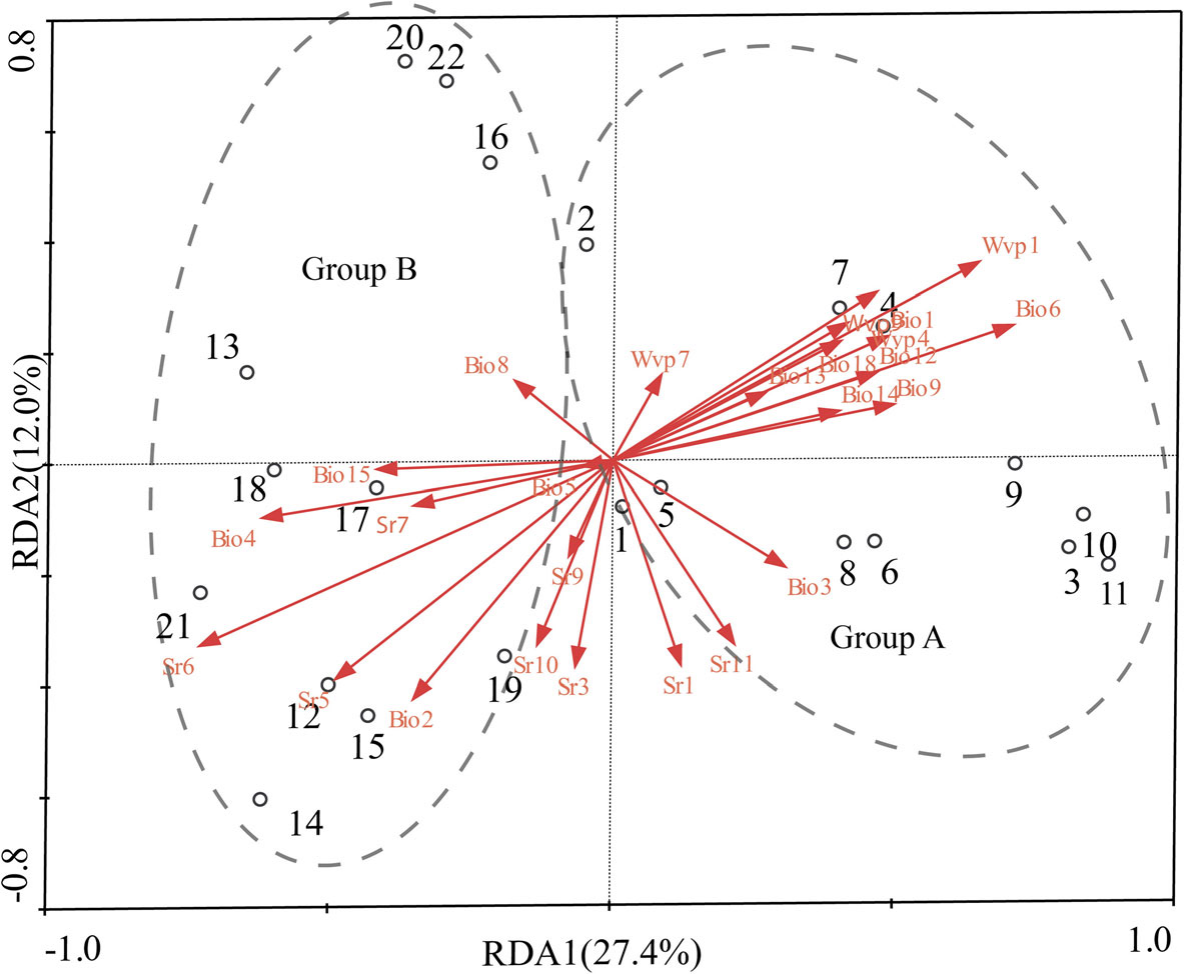
Redundancy analysis of *Pterocarya stenoptera* showing the relative contribution of each environmental variation shaping population genetic structure. The biplot depicts the eigenvalues and lengths of eigenvectors for the RDA

The Bayesian analysis of the population structure of *P. stenoptera* (Fig. 1) demonstrated that the highest *Delta K* value (Fig. 2) was obtained when 22 populations were clustered into two groups. The first group is Group A (P1 to P11), and the second group is Group B (P12 to P22). Despite the 22 populations of *P. stenoptera* could be divided into two groups, the genetic variations between the two groups was very low (6.69%, *F*_CT_ = 0.067, *P* < 0.001; Table 2), and most genetic variations occurred within populations (75.67%, *F*_ST_ = 0.243, *P* < 0.001; Table 2). The value of gene flow (*Nm*) among all populations was 2.022. To detect the roles of the 30 environmental variables in this genetic differentiation and their relative contribution on this differentiation, a constrained linear ordination analysis, redundancy analysis (RDA), was performed. The results of RDA are shown in Table 3 and Fig. 3. Correlations between the genetic variables of 1006 alleles and the 25 environmental variables in axes 1 and 2 were both 1.000. The ratios of the eigenvalues of axes 1 and 2 were 27.4 and 12.0%, respectively. RDA analysis showed that these environmental variables could divide the populations of *P. stenoptera* into two groups, whereas the genetic differentiation between them is very weak (Fig. 3). This is consistent with the result of STRUCTURE and analysis of molecular variance (AMOVA). Five environmental variables were significantly correlated with RDA axes 1 and 2 (Table 3). Among these five environmental variables, mean diurnal range (Bio2), temperature seasonality (Bio4), and minimum temperature of the coldest month (Bio6) were related to temperature; solar radiation in June (Sr6) was related to light; and water vapor pressure in January (Wvp1) was related to air humidity. Bio6 and Sr6 showed the strongest correlation with genetic variables among the five environmental variables.

**Table 2:** Hierarchical AMOVAs for SCoT variation surveyed in *Pterocarya stenoptera*

Source of variation	d.f.	%Total variance	*F*-statistic	*P*-value
Among groups	1	6.69	*F*_CT_ = 0.067	*P* < 0.001
Among populations within groups	20	17.64	*F*_SC_ = 0.189	*P* < 0.001
Within populations	485	75.67	*F*_ST_ = 0.243	*P* < 0.001

**Table 3:** Correlations between environmental variables and the ordination axes

Environmental variable	Axe 1	Axe 2	Axe3	Axe 4
Bio1	0.330	0.226	−0.137	0.184
Bio2	−0.238	−0.440^*^	−0.091	0.033
Bio3	0.208	−0.200	0.043	0.102
Bio4	−0.422^*^	−0.101	− 0.059	− 0.166
Bio5	− 0.036	− 0.011	− 0.116	0.099
Bio6	0.481^*^	0.245	−0.005	0.205
Bio8	−0.119	0.149	0.171	−0.457^*^
Bio9	0.338	0.100	−0.102	0.429^*^
Bio12	0.317	0.160	−0.098	0.440^*^
Bio13	0.184	0.124	−0.253	0.290
Bio14	0.272	0.088	0.082	0.467^*^
Bio15	−0.284	−0.013	− 0.286	−0.204
Bio18	0.281	0.252	−0.103	0.055
Sr1	0.084	−0.381	0.303	0.087
Sr3	−0.044	−0.382	0.295	−0.140
Sr5	−0.332	−0.402	0.171	−0.267
Sr6	−0.496^*^	−0.338	− 0.021	−0.195
Sr7	−0.239	−0.081	− 0.039	0.359
Sr9	−0.052	−0.177	0.123	0.512^*^
Sr10	−0.090	−0.341	0.258	0.292
Sr11	0.148	−0.342	0.319	0.253
Wvp1	0.439^*^	0.363	0.033	0.375
Wvp4	0.316	0.309	−0.075	0.415^*^
Wvp7	0.057	0.157	0.188	0.156
Wvp9	0.273	0.218	0.174	0.304

Statistically significant correlation by ^*^ *P* < 0.05 and ^**^ *P* < 0.01

### Characterization of environment-associated loci (EAL)

**Fig. 4:**
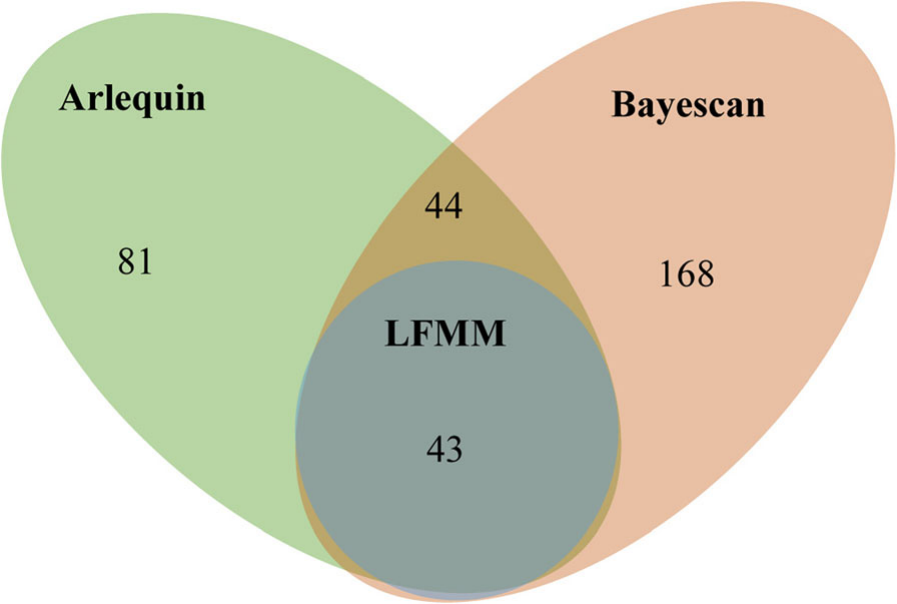
The results of outlier loci detected by Bayescan and Arlequin and EAL were identified by latent factor mixed model analysis

A total of 81 outlier loci (8.1% of 1006 SCoT alleles) with *F*_ST_ *P*-value below 0.05 were identified by using the hierarchical island model in Arlequin (Fig. 4; Additional file 1). Moreover, 168 outlier loci (16.7% of 1006 SCoT alleles) with posterior probability above 0.76 (i.e., log10PO > 0.5) were identified by using Bayesian method in BayeScan (Fig. 4; Additional file 1). To reduce the false positive rate, the loci detected by both methods were considered as outlier loci. Here, a total of 44 mutual loci (4.4% of 1006 SCoT alleles) were detected by both methods. Latent factor mixed model (LFMM) analysis was subsequently performed to verify whether these outlier loci were driven by environmental variables. As a result, 43 EAL (4.0% of 1006 SCoT alleles) associated with at least one environmental variable were identified (Table 4). Among the 25 environment variables that we detected, Sr6, Bio6, Bio4, and Wvp1 were associated with the highest numbers of EAL. The results suggested that they play major role in the genetic differentiation of *P. stenoptera.*

**Table 4:** The EAL as indicated by |z|-score

Locus	Bio1	Bio2	Bio3	Bio4	Bio5	Bio6	Bio8	Bio9	Bio12	Bio13	Bio14	Bio15	Bio18	Sr1	Sr3	Sr5	Sr6	Sr7	Sr9	Sr10	Sr11	Wvp1	Wvp4	Wvp7	Wvp9
5–130			5.750	6.627		4.102	6.370	4.443				6.292		3.921		3.479	4.326				4.226	3.849			
5–196				5.530		6.511		4.623	6.592		5.383	9.446				4.982	6.302					5.807	4.437		5.237
5–226	3.395	6.524				5.259			4.431		4.189		4.744			5.127	6.424					7.350	5.109	3.575	5.669
5–230	4.884	7.314		3.814		6.643		4.253	5.348	3.315	5.151	3.465	5.096			5.858	7.276					8.560	6.311	4.215	6.798
5–289	3.502	5.595				4.895			3.793		3.780		4.392			4.311	5.725					7.043	4.878	3.932	5.700
5–292		5.905				4.384							3.994			3.719	5.343					6.179	4.094	3.805	5.100
5–306	5.871	8.185		6.018		9.322		4.325	5.575		4.964	8.339	3.532	3.754	5.799	9.849	11.633			4.803		9.663	7.428	4.038	7.658
5–319	5.150	7.412		3.753		6.818		4.043	5.600	3.597	5.361	3.620	5.619			5.904	7.916					9.166	6.660	5.129	7.923
5–577		5.655											3.741				3.679					4.527		3.587	4.163
5–619		5.773				3.793							4.034				4.655					5.680	3.697	3.681	4.796
5–877		5.424				4.430							3.877	3.871		3.821	5.148					6.187	4.323	3.556	5.065
9–250			7.615	14.233	8.842	6.514		4.420							3.411	7.837	8.821	10.509	7.195	9.917	4.650	5.896		5.608	
9–534							7.184																	4.497	4.385
16–297	5.349			3.448		5.795		6.500	5.055		5.425	4.318				4.970	4.749		3.602			6.056	6.293		4.844
16–624	3.634	3.972				3.899		5.227	3.955		4.522				4.217	5.440	4.079					4.839	5.491		3.694
18–210												3.680	3.503	5.995	4.266					5.079	6.120				
18–314																		3.630							
18–358														4.155							4.356				
18–529																									
25–223			4.006	3.451						5.108		4.955		6.367	5.469						5.759				
25–337			4.978	6.105	4.560					4.547		5.069		4.282	4.164		3.956	5.477			4.013				
25–371			5.525	7.636	6.057	4.148	3.645		4.730	4.663	4.000						4.592	10.149	7.601	4.558					
25–469	3.426		6.405	6.251		5.556		4.326			3.371	5.857		6.724	4.385		5.046	3.674			7.313	4.946			4.647
25–544			5.823	6.904	4.513	3.695							6.829				4.416	9.988	6.449	4.128				5.164	
25–604			4.371	6.092		5.306							4.002	3.745	3.310		5.296	6.100			3.983	3.934			
25–790			6.826	6.106	3.808									4.014	4.092			7.277	3.487					3.451	
25–795			7.347	5.972	3.614									4.566	4.526			6.892			3.455			3.358	
25–848			7.360	6.860	4.000									3.687	3.443		3.686	6.875						3.731	
27–255	10.044				11.533	6.660	3.296	9.039	13.096	7.561	14.510	7.464	3.538		4.789	5.674	4.410	9.650	9.908	6.121	3.996	8.758	12.591	10.677	12.102
27–274	4.635	3.499				4.608		5.828	4.812	3.303	5.016				3.578	5.235	3.950		3.297			5.181	5.686		4.082
27–609	8.017	5.809			4.056	8.096		7.713	9.997	9.524	7.186		8.121	3.316	5.953	7.187	7.085					8.945	8.807	3.623	6.998
30–655	3.317													5.515	5.200	5.254	3.896	4.212	7.814	8.698	6.657				
31–309	4.912					5.121		7.471	5.057	3.518	5.149				4.259	6.091	4.586		3.312			6.279	6.748		4.229
31–313	3.878			5.167		5.034	3.919	7.016	3.742		4.139					5.617	4.994					6.166	5.567		
31–382											3.338							3.486	4.944				3.509		
31–540	4.699		4.295	4.612		5.793		5.409	3.309		3.652	6.122				4.254	4.864					5.412	5.179		4.321
35–268	6.457		3.985	8.051		8.377		5.185							3.949	8.778	9.610	7.125	5.789	7.972	3.663	8.763	5.855		3.542
35–328				5.022		4.287										5.153	5.330	4.465	4.929	6.173				3.731	
35–333				4.087		3.576										3.974	4.109	4.276	5.065	5.734				3.787	
35–337			7.404	8.565	5.726	3.736				4.734		3.502				3.523	5.619	6.355				3.422			
35–340			6.609	7.941	5.030	3.610				3.467							5.100	5.584							
35–559			5.886	7.066	3.292	4.351	4.269	3.371								3.300	5.275	3.816			3.336	4.205			
35–679			6.468	8.127	5.895	3.365				3.945							5.073	5.867						3.578	

## Discussion

In this study, we investigated the spatial distribution of genetic variation in wild populations of *P. stenoptera* across its distribution area in China to provide insights into the complex interplay between multiple environmental variables and adaptive genetic differentiation of this widespread broad-leaved tree species and thus improve our understanding of the genetic mechanism of local adaptation. Landscape genomics has developed rapidly in the past decade, which has been proved to be an effective method for studying adaptive evolution of species [4]. Here, we revealed the adaptive evolution of *P. stenoptera* in response to environmental variables by using 1006 SCoT alleles.

The role of environmental factors in shaping the spatial genetic structure of species has been a key issue in landscape genomics research [18, 19]. Spatial genetic structure of species is the result of the interaction of multiple factors, e.g., population demographic history, geographical or ecological barriers, transmission mode of seeds and pollen, geological events, and divergent selection of environmental factors [2, 20]. In previous studies on population genetics, more attention was paid to the effects of the first few factors on the population genetic structure [21]. However, the efficient gene flow of species tends to obscure previous genetic structures, especially those based on nuclear genes [16]. Meanwhile, a completely new population genetic structure will be formed because of the divergent selection, genetic drift, and inbreeding. Although genetic drift and inbreeding can also rapidly alter the genetic structure of species, they occur more often in small and isolated populations [22]. However, *P. stenoptera* seems not suitable for this situation, due to a large population size. Meanwhile, there is high gene flow among populations of *P. stenoptera* (*Nm =* 2.022)*.* The efficient gene flow (*Nm* > 1) would avoid the isolation between populations [23]. Therefore, the current genetic structure for *P. stenoptera* based on SCoT marker can hardly be attributed to genetic drift and inbreeding. Our survey showed that the 22 populations of *P. stenoptera* were divided into two groups, i.e., Group A (P1 to P11) and Group B (P12 to P22). Three possible reasons can be used to explain this intraspecific population differentiation. The first is caused by population demographic history. There were two refugia for *P. stenoptera* during the Quaternary glacial period; the present spatial genetic pattern was formed by the spread and redistribution of the population from the two refugia after glaciation. The second is due to geographical or ecological barriers. There is a geographical or ecological barrier between the two groups, which blocks or interferes with the gene flow and leads to the genetic differentiation between the two groups. The third is caused by divergent selection of environmental factors. The two groups are in different habitats, and different environmental factors lead to the allele frequency difference of the naturally selected genes, which eventually leads to the genetic differentiation between the two groups. Assuming that the first reason is true for *P. stenoptera*, the populations as refugia have the highest genetic diversity, and the population that is away from the refugia will gradually reduce its genetic diversity because of the founder effect. However, neither Group A nor Group B has gradient descent from the population with the highest genetic diversity. More importantly, Group A is discontinuous, which is separated by Group B. Thus, it is impossible to have two refugia for *P. stenoptera*. Overall, the first possible reason does not explain the genetic differentiation between the two groups of *P. stenoptera*. The second possible reason is considered to explain the genetic differentiation between the two groups of *P. stenoptera*. There must be ecological or geographical barriers between the two groups. In fact, the populations of the two groups are continuously distributed, and there are no geographical or geographical barriers. Even if the differentiation is caused by the second possible reason, the differentiation between them must be significant. Our results of hierarchical AMOVA (*F*_CT_ = 0.067, *P* < 0.001) do not support this interpretation. By assuming the genetic divergence of *P. stenoptera* caused by the third possible reason, a weak genetic differentiation between the two groups would be expected because of the interaction between strong gene flow and divergent selection of environmental factors. The results of hierarchical AMOVA were consistent with this expectation. Our RDA results (Fig. 3) also showed that environmental variables could slightly separate the two groups of *P. stenoptera*. The populations of Group A live at higher temperature of the coldest month and higher water vapor pressure in January than those of Group B, whereas populations of Group B live at higher value of temperature seasonality and higher solar radiation in June than those of Group A. The genetic differentiation caused by the divergent selection of environmental differences will be maintained due to the existence of heterogeneous environments, which is different from the genetic differentiation caused by genetic drift. Taken together, the third possible reason is more suitable for *P. stenoptera.*

Because of the inherent limitations of SCoT markers, sequence information cannot be obtained [4]. Thus, the genes cannot be annotated for their function. Although we do not know what genes these loci are or which genes they are linked to, we can know which environmental factors these loci are related to. Here, the results of LFMM analysis showed that Sr6, Bio6, Bio4, and Wvp1 were associated with the highest numbers of EAL, which suggested that these environmental variables play a major role on the genetic differentiation of *P. stenoptera* genome. Recent landscape genomic studies have proposed a hypothesis that environmental variables related to ecological habitats of species play key roles in driving adaptive differentiation of species genome [2, 14, 15]. Among the four environmental variables associated with the largest number of adaptive loci, Sr6 was associated with the ecological habitat of “prefered light”, and Bio6 and Wvp1 were associated with the ecological habitat of “warm and humid environment”. *P. stenoptera* blooms from April to May, and its fruits ripen from August to September. Therefore, we speculated that *P. stenoptera* might be more sensitive to light during fruit development. Our RDA results indicate that there is a significant difference in the amount of solar radiation in June (Sr6) between the two groups, which promoted the adaptive differentiation of these related adaptive loci. Bio6 and Wvp1 refer to the minimum temperature of the coldest month and water vapor pressure in January, respectively. Similarly, there are significant differences of the two environmental variables between the two populations. In China, January is the driest month. These two variables were associated with larger numbers of adaptive loci, suggesting that temperature and water vapor in extreme environments were the main causes of their adaptive differentiation. In fact, previous studies have proved that there is a significant difference in the cold resistance of *P. stenoptera* from different provenance [24]. Surprisingly, seasonal variations in temperature (Bio4) might also have a significant impact on the adaptive differentiation of *P. stenoptera*. Overall, the hypothesis that environmental variables related to ecological habitats of species play key roles in driving adaptive differentiation of species genome is also suitable for *P. stenoptera.*

## Conclusions

In the present study, SCoT markers were used to investigate adaptive genetic differentiation in *P. stenoptera*. Our survey showed that the 22 populations of *P. stenoptera* were divided into two groups. Although spatial genetic structure of species is the result of the interaction of multiple factors, our results suggested that the divergent selection of environmental differences play a major role on the genetic differentiation of *P. stenoptera.* Our results also provided empirical evidence that environmental variables related to ecological habitats of species play key roles in driving adaptive differentiation of species genome.

## Methods

### Sample collection

**Fig. 5:**
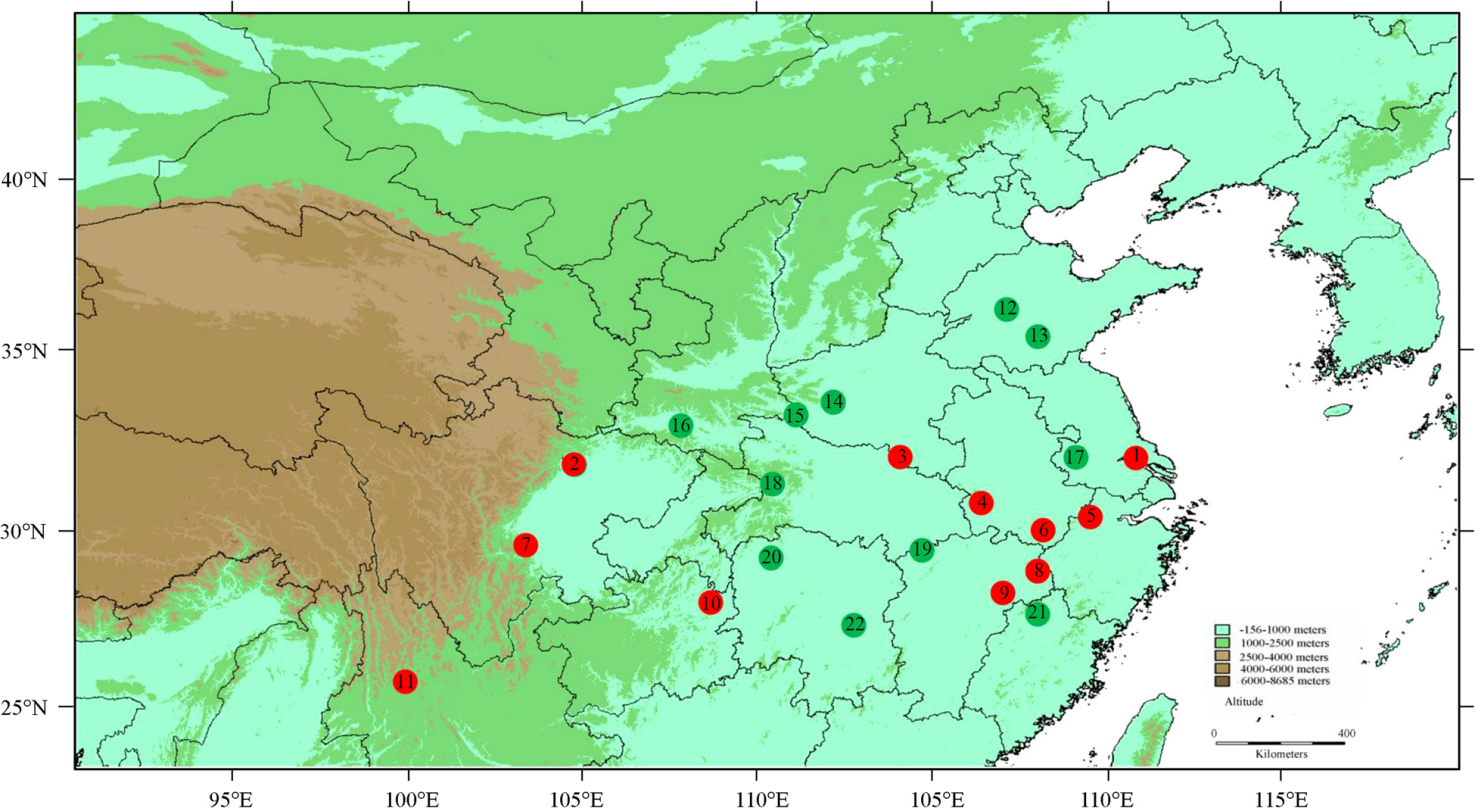
Locations of the 22 sampled *Pterocarya stenoptera* populations. The red and green colors of the populations represent the groups identified by STRUCTURE, red for Group A, green for Group B. Map yielded by software DIVA-GIS 7.5.0, the software and free spatial data were downloaded from http://www.diva-gis.org

A total of 510 individuals from 22 natural populations were sampled from the entire distribution range of *P. stenoptera* in China (Fig. 5). Each population sample contained 20 to 24 individuals (Table 1), and each individual was at least 20 m away from each other. Young, healthy leaves were collected and stored in zip-lock bags containing silica gel at room temperature until DNA extraction. The geographical coordinates and number of samples for each population are shown in Table 1. After identified by Dr. Yong Li, each population deposit a voucher specimen at the herbarium of College of Forestry, Henan Agricultural University, Zhengzhou, China (voucher no. LiPS2017001–2,017,022). No specific permits were required for *P. stenoptera*, all samples were collected following current Chinese regulations.

### Molecular protocols

Genomic DNA was extracted from approximately 30 mg of dried leaves by using the standard Plant DNA Extraction Kit (Tiangen, Beijing, China) protocol. DNA was then quantified using the ND5000 Ultra Micro UV-Vis spectrophotometer (BioTeke, Beijing, China). All individuals were genotyped using SCoT markers. Despite its inherent defects lacking DNA sequence information, the SCoT marker has been used for landscape genomic studies because it has the advantages of non-requirement for genomic information, high repeatability, and high throughput [2, 14, 15]. After preliminary screening of the polymorphism and reproducibility of all SCoT primers [25], nine primers (SCoT5, SCoT9, SCoT16, SCoT18, SCoT25, SCoT27, SCoT30, SCoT31, and SCoT35) were selected for polymerase chain reactions (PCRs). SCoT5, SCoT16, and SCoT25 were 5′ FAM fluorescently labeled primers; SCoT9, SCoT18, and SCoT30 were 5′ HEX primers; SCoT27, SCoT31, and SCoT35 were 5′ TAMRA primers. PCR amplification was carried out in a 20 μL reaction mixture containing 20 ng of template DNA, 10 mM reaction buffer (pH 8.3), 0.2 mM of each dNTP, 0.3 μM primer, and 1 unit of Taq polymerase (Tiangen, Beijing, China). PCRs were performed in a Mastercycler nexus thermocycler (Eppendorf, Hamburg, Germany) with an initial denaturation at 94 °C for 5 min followed by 35 cycles with denaturation at 94 °C for 40 s, primer-specific annealing temperature (52 °C for SCoT27; 56 °C for SCoT5, SCoT9, SCoT16, SCoT18, SCoT30, SCoT31, and SCoT35; and 58 °C for SCoT25) for 40 s, extension at 72 °C for 90 s and with a final extension at 72 °C for 5 min, and termination by a final hold at 4 °C. Finally, 3 μL of PCR products was mixed with 10 μL of HiDi formamide and electrophoresed on an ABI 3730 DNA Analyzer at BGI (Beijing, China). The size of PCR products is determined according to the internal standard LIZ1200 (Applied Biosystems, Foster City, USA).

### Data analysis

The SCoT fragments were identified based on the presence or absence of peaks viewed in GeneMarker 2.2.0 (SoftGenetics, State College, Pennsylvania, USA). The raw information was then transformed into a 1/0 matrix. To reduce the error reading rate of SCoT fragments, the peaks within 100–1200 bp and relative fluorescent units above 200 were scored. Subsequent population genetic analyses were carried out with the basis of the 1/0 matrix from SCoT markers.

Genetic parameters, including polymorphic allele number (*N*_A_), allele frequencies, Nei’s measure of gene diversity (*H*_E_) [26], and percentage of polymorphic alleles (*PPA*), were calculated using AFLP-SURV 1.0 [27]. Genetic structure of the 22 populations of *P. stenoptera* was assessed using the Bayesian-based program STRUCTURE 2.3.4 [28]. The program was run with *K* values from 1 to 10 with 10 replicates for each *K*, and a burn-in period of 10,000 and 10,000 Markov chain Monte Carlo iterations. The admixture model with independent allele frequencies was used for this analysis. The optimal *K* value with the most suitable population clusters was judged according to the *∆K* values introduced by Evanno et al. [29], and this method was executed by Structure Harvester [30]. The average value of the admixture coefficients over 10 runs was calculated using CLUMPP 1.1 [31]. The barplots of STRUCTURE were obtained by DISTRUCT 1.1 [32]. The distribution of genetic differentiation at various levels for the 22 populations of *P. stenoptera* was characterized using hierarchical AMOVA within Arlequin 3.5 [33]. To calculate gene flow (*Nm*) among populations, we also calculated *F*_ST_ based on neutral loci (i.e. excluding all outlier loci identified by Arlequin and BayeScan). The value of gene flow was estimated according to 1/4(1/*F*_ST_ − 1). A total of 43 environmental variables (Additional files 2 and 3), including 11 temperature variables, 8 precipitation variables, 12 solar radiation variables, and 12 water vapor pressure variables, were downloaded from Worldclim (http://www.diva-gis.org/climate) from 1970 to 2000 at 2.5 arcmin resolution and further extracted using DIVA-GIS 7.5 [34]. The strongly correlated environmental variables with a Pearson correlation coefficient above 0.95 were eliminated. The correlation analysis was performed in SPSS 19 (SPSS Inc., Chicago, IL, USA). Thus, the remaining environmental variables were used for the subsequent RDA and environmental association analysis. After removing the strongly correlated environmental variables, 8 temperature variables (Bio1, Bio2, Bio3, Bio4, Bio5, Bio6, Bio8, and Bio9), 5 precipitation variables (Bio12, Bio13, Bio14, Bio15, and Bio18), 8 solar radiation variables (Sr1, Sr3, Sr5, Sr6, Sr7, Sr9, Sr10, Sr11), and 4 water vapor pressure variables (Wvp1, Wvp4, Wvp7, Wvp9) were retained. To infer the influence of the environmental variables to population genetic differentiation, we performed a constrained linear ordination analysis, RDA, in CANOCO 4.5 [35]. Here, allele frequencies per population (Additional file 4) were used as response variable and the remaining 30 uncorrelated environmental variables were used as explanatory variables.

To minimize the false positive rate, two methods were used to identify the mutual outlier loci for subsequent environmental association analysis. The first method is a hierarchical island model in Arlequin 3.5 [33]. The advantage of this approach is that it has better sensitivity to samples with common history and substructure. The program parameters used were as follows: 20,000 coalescent simulations, 100 simulated demes, and the number of simulated groups suggested by the results of STRUCTURE based on all loci. The loci with *F*_ST_ *P*-value below 0.05 were considered as outlier loci, whereas those with total allele frequencies below 0.05 or above 0.95 were removed from the final results. The second method is a Bayesian method in BayeScan 2.0 [36]. The advantage of this approach is that it allows the population samples to have different amounts of genetic drift or different demographic histories [37]. The program parameters used were as follows: sample size of 5000, thinning interval of 10, 20 pilot runs with 5000 run length, 50,000 burn-in iterations, and 10,000 prior odds. The loci with posterior probability over 0.76 were considered as outlier loci. To verify whether these loci are driven by environmental variables, environmental association analysis were performed using LFMM 1.2 [38]. This method based on latent factor mixture model can effectively avoid the misidentification of EAL caused by population history or population structure. The analysis was run with the following parameters: 10,000 sweeps, 1000 burn-in sweeps, and the number of latent factors from the results of STRUCTURE based on neutral loci (excluding all suspected outlier loci identified by Arlequin and BayeScan). The loci with |z| over 3 and *P* below 0.001 were considered as EAL.

## Additional files

Additional file 1: The outlier loci identified by Arlequin and BayeScan. (DOCX 31 kb)

Additional file 2: Fifty-five environmental variables used in this study. (DOCX 16 kb)

Additional file 3: Environmental variables for each location from the WorldClim database. (DOCX 26 kb)

Additional file 4: Allele frequencies of 1006 alleles for each population. (DOCX 253 kb)

## Abbreviations

AFLPs: Amplified fragment length polymorphisms
AMOVA: Analysis of molecular variance
EAL: Environment-associated loci
ISSRs: Inter-simple sequence repeats
PCRs: Polymerase chain reactions
RDA: Redundancy analysis
SCoTs: Start codon targeted polymorphisms
