# Supplementary material for: Adaptive genetic differentiation in Pterocarya stenoptera (Juglandaceae) driven by multiple environmental variables were revealed by landscape genomics
Source: BMC Plant Biol. 2018 Nov 27;18:306. doi: 10.1186/s12870-018-1524-x (PMC6260741; doi:10.1186/s12870-018-1524-x)
Supplement: Supplementary file 1 — The outlier loci identified by Arlequin and BayeScan. (DOCX 31 kb) [file 12870_2018_1524_MOESM1_ESM.docx]

**Additional file 1** The outlier loci identified by Arlequin and BayeScan.

|  | Arlequin | |  |  | BayeScan | |  |
| --- | --- | --- | --- | --- | --- | --- | --- |
| Locus | Observe FST | *FST P-value* |  | Locus | prob | log10(PO) | q value |
| 5-130 | 0.633 | 0.001 |  | 5-130 | 1.000 | 1000 | 0.000 |
|  |  |  |  | 5-158 | 1.000 | 1000 | 0.000 |
| 5-196 | 0.457 | 0.037 |  | 5-196 | 1.000 | 1000 | 0.000 |
| 5-226 | 0.595 | 0.001 |  | 5-226 | 1.000 | 1000 | 0.000 |
| 5-230 | 0.514 | 0.010 |  | 5-230 | 1.000 | 1000 | 0.000 |
|  |  |  |  | 5-256 | 0.884 | 0.880 | 0.005 |
|  |  |  |  | 5-274 | 1.000 | 1000 | 0.000 |
| 5-289 | 0.543 | 0.008 |  | 5-289 | 1.000 | 1000 | 0.000 |
| 5-292 | 0.539 | 0.008 |  | 5-292 | 1.000 | 1000 | 0.000 |
| 5-306 | 0.458 | 0.038 |  | 5-306 | 1.000 | 1000 | 0.000 |
| 5-319 | 0.608 | 0.001 |  | 5-319 | 1.000 | 1000 | 0.000 |
|  |  |  |  | 5-373 | 1.000 | 1000 | 0.000 |
|  |  |  |  | 5-428 | 1.000 | 1000 | 0.000 |
|  |  |  |  | 5-432 | 1.000 | 1000 | 0.000 |
|  |  |  |  | 5-437 | 0.990 | 2.014 | 0.000 |
| 5-577 | 0.462 | 0.034 |  | 5-577 | 1.000 | 1000 | 0.000 |
| 5-596 | 0.083 | 0.023 |  |  |  |  |  |
| 5-619 | 0.520 | 0.013 |  | 5-619 | 1.000 | 1000 | 0.000 |
|  |  |  |  | 5-663 | 0.970 | 1.507 | 0.001 |
|  |  |  |  | 5-779 | 1.000 | 1000 | 0.000 |
| 5-877 | 0.474 | 0.024 |  | 5-877 | 1.000 | 1000 | 0.000 |
|  |  |  |  | 5-883 | 1.000 | 1000 | 0.000 |
| 9-250 | 0.461 | 0.043 |  | 9-250 | 1.000 | 1000 | 0.000 |
|  |  |  |  | 9-266 | 1.000 | 1000 | 0.000 |
| 9-382 | 0.058 | 0.021 |  |  |  |  |  |
| 9-423 | 0.084 | 0.043 |  |  |  |  |  |
| 9-457 | 0.050 | 0.011 |  |  |  |  |  |
|  |  |  |  | 9-476 | 0.996 | 2.396 | 0.000 |
|  |  |  |  | 9-487 | 0.984 | 1.778 | 0.001 |
|  |  |  |  | 9-498 | 0.974 | 1.570 | 0.001 |
|  |  |  |  | 9-501 | 1.000 | 1000 | 0.000 |
| 9-534 | 0.448 | 0.044 |  | 9-534 | 1.000 | 1000 | 0.000 |
|  |  |  |  | 9-600 | 1.000 | 1000 | 0.000 |
|  |  |  |  | 9-606 | 1.000 | 1000 | 0.000 |
|  |  |  |  | 16-131 | 1.000 | 1000 | 0.000 |
|  |  |  |  | 16-196 | 1.000 | 1000 | 0.000 |
| 16-231 | 0.053 | 0.000 |  |  |  |  |  |
| 16-254 | 0.046 | 0.010 |  |  |  |  |  |
|  |  |  |  | 16-286 | 0.935 | 1.159 | 0.002 |
|  |  |  |  | 16-289 | 0.933 | 1.145 | 0.002 |
|  |  |  |  | 16-292 | 1.000 | 1000 | 0.000 |
| 16-297 | 0.583 | 0.001 |  | 16-297 | 1.000 | 1000 | 0.000 |
|  |  |  |  | 16-320 | 1.000 | 1000 | 0.000 |
| 16-423 | 0.076 | 0.017 |  |  |  |  |  |
|  |  |  |  | 16-428 | 0.977 | 1.624 | 0.001 |
| 16-434 | 0.049 | 0.003 |  |  |  |  |  |
|  |  |  |  | 16-437 | 1.000 | 1000 | 0.000 |
|  |  |  |  | 16-463 | 1.000 | 1000 | 0.000 |
| 16-479 | 0.107 | 0.035 |  |  |  |  |  |
| 16-510 | 0.097 | 0.046 |  |  |  |  |  |
| 16-512 | 0.075 | 0.021 |  |  |  |  |  |
|  |  |  |  | 16-516 | 1.000 | 1000 | 0.000 |
| 16-579 | 0.095 | 0.042 |  |  |  |  |  |
| 16-624 | 0.569 | 0.007 |  | 16-624 | 0.991 | 2.062 | 0.000 |
|  |  |  |  | 16-778 | 1.000 | 1000 | 0.000 |
|  |  |  |  | 16-877 | 1.000 | 1000 | 0.000 |
|  |  |  |  | 16-883 | 1.000 | 1000 | 0.000 |
| 18-210 | 0.560 | 0.009 |  | 18-210 | 1.000 | 1000 | 0.000 |
|  |  |  |  | 18-224 | 1.000 | 1000 | 0.000 |
|  |  |  |  | 18-245 | 1.000 | 1000 | 0.000 |
|  |  |  |  | 18-253 | 1.000 | 1000 | 0.000 |
|  |  |  |  | 18-301 | 1.000 | 1000 | 0.000 |
| 18-314 | 0.542 | 0.012 |  | 18-314 | 1.000 | 1000 | 0.000 |
|  |  |  |  | 18-317 | 1.000 | 1000 | 0.000 |
|  |  |  |  | 18-328 | 1.000 | 1000 | 0.000 |
| 18-331 | 0.457 | 0.048 |  | 18-331 | 1.000 | 1000 | 0.000 |
|  |  |  |  | 18-352 | 1.000 | 1000 | 0.000 |
| 18-358 | 0.627 | 0.001 |  | 18-358 | 1.000 | 1000 | 0.000 |
|  |  |  |  | 18-375 | 1.000 | 1000 | 0.000 |
|  |  |  |  | 18-385 | 1.000 | 1000 | 0.000 |
|  |  |  |  | 18-389 | 1.000 | 1000 | 0.000 |
| 18-453 | 0.099 | 0.043 |  |  |  |  |  |
| 18-459 | 0.087 | 0.022 |  |  |  |  |  |
|  |  |  |  | 18-514 | 1.000 | 1000 | 0.000 |
| 18-529 | 0.563 | 0.005 |  | 18-529 | 1.000 | 1000 | 0.000 |
|  |  |  |  | 18-540 | 0.823 | 0.668 | 0.007 |
|  |  |  |  | 18-569 | 1.000 | 1000 | 0.000 |
|  |  |  |  | 18-598 | 1.000 | 1000 | 0.000 |
|  |  |  |  | 18-605 | 1.000 | 1000 | 0.000 |
|  |  |  |  | 18-610 | 1.000 | 1000 | 0.000 |
|  |  |  |  | 18-615 | 1.000 | 1000 | 0.000 |
|  |  |  |  | 18-621 | 1.000 | 1000 | 0.000 |
| 18-632 | 0.061 | 0.018 |  |  |  |  |  |
|  |  |  |  | 18-791 | 1.000 | 1000 | 0.000 |
|  |  |  |  | 18-834 | 1.000 | 1000 | 0.000 |
|  |  |  |  | 25-127 | 1.000 | 1000 | 0.000 |
|  |  |  |  | 25-134 | 1.000 | 1000 | 0.000 |
|  |  |  |  | 25-151 | 1.000 | 1000 | 0.000 |
|  |  |  |  | 25-163 | 1.000 | 1000 | 0.000 |
| 25-168 | 0.095 | 0.038 |  |  |  |  |  |
|  |  |  |  | 25-180 | 1.000 | 1000 | 0.000 |
| 25-223 | 0.492 | 0.024 |  | 25-223 | 1.000 | 1000 | 0.000 |
|  |  |  |  | 25-227 | 0.991 | 2.052 | 0.000 |
|  |  |  |  | 25-240 | 1.000 | 1000 | 0.000 |
|  |  |  |  | 25-245 | 0.998 | 2.744 | 0.000 |
|  |  |  |  | 25-253 | 1.000 | 1000 | 0.000 |
|  |  |  |  | 25-266 | 1.000 | 1000 | 0.000 |
|  |  |  |  | 25-271 | 0.803 | 0.610 | 0.008 |
|  |  |  |  | 25-283 | 0.996 | 2.419 | 0.000 |
|  |  |  |  | 25-287 | 0.984 | 1.789 | 0.001 |
| 25-304 | 0.074 | 0.021 |  |  |  |  |  |
|  |  |  |  | 25-327 | 1.000 | 1000 | 0.000 |
| 25-337 | 0.483 | 0.035 |  | 25-337 | 1.000 | 1000 | 0.000 |
| 25-352 | 0.072 | 0.031 |  |  |  |  |  |
|  |  |  |  | 25-364 | 1.000 | 1000 | 0.000 |
| 25-371 | 0.518 | 0.012 |  | 25-371 | 1.000 | 1000 | 0.000 |
|  |  |  |  | 25-383 | 1.000 | 1000 | 0.000 |
|  |  |  |  | 25-391 | 1.000 | 1000 | 0.000 |
|  |  |  |  | 25-410 | 1.000 | 1000 | 0.000 |
|  |  |  |  | 25-457 | 1.000 | 1000 | 0.000 |
|  |  |  |  | 25-461 | 0.849 | 0.750 | 0.006 |
| 25-469 | 0.477 | 0.032 |  | 25-469 | 1.000 | 1000 | 0.000 |
|  |  |  |  | 25-473 | 1.000 | 1000 | 0.000 |
|  |  |  |  | 25-506 | 1.000 | 1000 | 0.000 |
| 25-522 | 0.038 | 0.007 |  |  |  |  |  |
| 25-544 | 0.512 | 0.015 |  | 25-544 | 1.000 | 1000 | 0.000 |
| 25-551 | 0.104 | 0.028 |  |  |  |  |  |
| 25-604 | 0.581 | 0.024 |  | 25-604 | 1.000 | 1000 | 0.000 |
|  |  |  |  | 25-611 | 1.000 | 1000 | 0.000 |
| 25-790 | 0.449 | 0.042 |  | 25-790 | 1.000 | 1000 | 0.000 |
| 25-795 | 0.447 | 0.046 |  | 25-795 | 1.000 | 3.398 | 0.000 |
|  |  |  |  | 25-812 | 0.903 | 0.968 | 0.004 |
| 25-848 | 0.471 | 0.030 |  | 25-848 | 1.000 | 1000 | 0.000 |
| 25-884 | 0.078 | 0.039 |  |  |  |  |  |
| 25-1062 | 0.061 | 0.022 |  |  |  |  |  |
|  |  |  |  | 25-1084 | 1.000 | 1000 | 0.000 |
|  |  |  |  | 25-1090 | 1.000 | 1000 | 0.000 |
|  |  |  |  | 27-109 | 1.000 | 1000 | 0.000 |
|  |  |  |  | 27-168 | 1.000 | 1000 | 0.000 |
|  |  |  |  | 27-172 | 1.000 | 1000 | 0.000 |
|  |  |  |  | 27-180 | 1.000 | 1000 | 0.000 |
| 27-255 | 0.545 | 0.031 |  | 27-255 | 1.000 | 1000 | 0.000 |
|  |  |  |  | 27-260 | 1.000 | 1000 | 0.000 |
|  |  |  |  | 27-265 | 1.000 | 1000 | 0.000 |
| 27-274 | 0.570 | 0.003 |  | 27-274 | 1.000 | 1000 | 0.000 |
|  |  |  |  | 27-280 | 1.000 | 1000 | 0.000 |
|  |  |  |  | 27-286 | 1.000 | 1000 | 0.000 |
| 27-294 | 0.091 | 0.032 |  |  |  |  |  |
|  |  |  |  | 27-306 | 1.000 | 1000 | 0.000 |
|  |  |  |  | 27-347 | 1.000 | 1000 | 0.000 |
| 27-354 | 0.069 | 0.013 |  |  |  |  |  |
|  |  |  |  | 27-383 | 1.000 | 1000 | 0.000 |
|  |  |  |  | 27-387 | 1.000 | 1000 | 0.000 |
| 27-504 | 0.081 | 0.028 |  |  |  |  |  |
| 27-510 | 0.082 | 0.047 |  |  |  |  |  |
|  |  |  |  | 27-519 | 1.000 | 1000 | 0.000 |
| 27-526 | 0.087 | 0.006 |  |  |  |  |  |
|  |  |  |  | 27-555 | 1.000 | 1000 | 0.000 |
|  |  |  |  | 27-561 | 1.000 | 1000 | 0.000 |
|  |  |  |  | 27-567 | 1.000 | 1000 | 0.000 |
|  |  |  |  | 27-570 | 0.921 | 1.068 | 0.003 |
|  |  |  |  | 27-587 | 1.000 | 1000 | 0.000 |
| 27-609 | 0.512 | 0.020 |  | 27-609 | 1.000 | 1000 | 0.000 |
|  |  |  |  | 27-616 | 1.000 | 1000 | 0.000 |
|  |  |  |  | 27-750 | 1.000 | 1000 | 0.000 |
|  |  |  |  | 27-758 | 1.000 | 1000 | 0.000 |
|  |  |  |  | 30-180 | 0.961 | 1.394 | 0.001 |
| 30-230 | 0.043 | 0.009 |  |  |  |  |  |
|  |  |  |  | 30-306 | 0.986 | 1.854 | 0.000 |
|  |  |  |  | 30-328 | 1.000 | 1000 | 0.000 |
|  |  |  |  | 30-333 | 1.000 | 1000 | 0.000 |
| 30-354 | 0.117 | 0.046 |  |  |  |  |  |
| 30-358 | 0.086 | 0.008 |  |  |  |  |  |
|  |  |  |  | 30-475 | 1.000 | 1000 | 0.000 |
|  |  |  |  | 30-480 | 1.000 | 1000 | 0.000 |
|  |  |  |  | 30-483 | 1.000 | 1000 | 0.000 |
|  |  |  |  | 30-490 | 1.000 | 1000 | 0.000 |
|  |  |  |  | 30-500 | 1.000 | 1000 | 0.000 |
|  |  |  |  | 30-508 | 0.979 | 1.677 | 0.001 |
| 30-532 | 0.061 | 0.024 |  |  |  |  |  |
| 30-538 | 0.049 | 0.009 |  |  |  |  |  |
| 30-632 | 0.070 | 0.015 |  |  |  |  |  |
| 30-655 | 0.453 | 0.045 |  | 30-655 | 1.000 | 1000 | 0.000 |
|  |  |  |  | 30-891 | 0.907 | 0.987 | 0.003 |
|  |  |  |  | 30-896 | 1.000 | 1000 | 0.000 |
|  |  |  |  | 31-102 | 1.000 | 1000 | 0.000 |
|  |  |  |  | 31-106 | 1.000 | 1000 | 0.000 |
| 31-309 | 0.675 | 0.000 |  | 31-309 | 1.000 | 1000 | 0.000 |
| 31-313 | 0.471 | 0.029 |  | 31-313 | 1.000 | 1000 | 0.000 |
| 31-382 | 0.467 | 0.031 |  | 31-382 | 1.000 | 1000 | 0.000 |
|  |  |  |  | 31-390 | 1.000 | 1000 | 0.000 |
|  |  |  |  | 31-408 | 1.000 | 1000 | 0.000 |
|  |  |  |  | 31-412 | 1.000 | 1000 | 0.000 |
|  |  |  |  | 31-416 | 1.000 | 1000 | 0.000 |
|  |  |  |  | 31-424 | 1.000 | 1000 | 0.000 |
|  |  |  |  | 31-450 | 0.986 | 1.835 | 0.000 |
| 31-540 | 0.562 | 0.023 |  | 31-540 | 1.000 | 1000 | 0.000 |
|  |  |  |  | 31-623 | 1.000 | 1000 | 0.000 |
| 35-268 | 0.524 | 0.016 |  | 35-268 | 1.000 | 1000 | 0.000 |
|  |  |  |  | 35-306 | 1.000 | 1000 | 0.000 |
|  |  |  |  | 35-311 | 1.000 | 1000 | 0.000 |
| 35-328 | 0.480 | 0.031 |  | 35-328 | 1.000 | 1000 | 0.000 |
| 35-333 | 0.521 | 0.016 |  | 35-333 | 1.000 | 1000 | 0.000 |
| 35-337 | 0.601 | 0.001 |  | 35-337 | 1.000 | 1000 | 0.000 |
| 35-340 | 0.556 | 0.003 |  | 35-340 | 1.000 | 1000 | 0.000 |
| 35-354 | 0.012 | 0.000 |  |  |  |  |  |
| 35-358 | 0.031 | 0.004 |  |  |  |  |  |
|  |  |  |  | 35-480 | 1.000 | 1000 | 0.000 |
|  |  |  |  | 35-483 | 1.000 | 1000 | 0.000 |
| 35-500 | 0.072 | 0.034 |  |  |  |  |  |
| 35-508 | 0.074 | 0.039 |  |  |  |  |  |
| 35-559 | 0.503 | 0.015 |  | 35-559 | 1.000 | 1000 | 0.000 |
|  |  |  |  | 35-655 | 1.000 | 1000 | 0.000 |
|  |  |  |  | 35-668 | 1.000 | 1000 | 0.000 |
| 35-679 | 0.532 | 0.018 |  | 35-679 | 0.853 | 0.764 | 0.005 |
