# Supplementary material for: Adaptive genetic differentiation in Pterocarya stenoptera (Juglandaceae) driven by multiple environmental variables were revealed by landscape genomics
Source: BMC Plant Biol. 2018 Nov 27;18:306. doi: 10.1186/s12870-018-1524-x (PMC6260741; doi:10.1186/s12870-018-1524-x)
Supplement: Supplementary file 2 — Fifty-five environmental variables used in this study. (DOCX 16 kb) [file 12870_2018_1524_MOESM2_ESM.docx]

**Additional file 2** Fifty-five environmental variables used in this study.

| Temperature  (period 1970-2000) | Bio1: Annual mean temperature ( ℃×10) |
| --- | --- |
|  | Bio2: Mean diurnal range (Mean of monthly (max temp - min temp)) |
|  | Bio3: Isothermality (Bio2/Bio7) (×100) |
|  | Bio4: Temperature seasonality (standard deviation ×100) |
|  | Bio5: Max temperature of warmest month ( ℃×10) |
|  | Bio6: Min temperature of coldest month ( ℃×10) |
|  | Bio7: Temperature annual range (E5-E6) |
|  | Bio8: Mean temperature of wettest quarter ( ℃×10) |
|  | Bio9: Mean temperature of driest quarter ( ℃×10) |
|  | Bio10: Mean temperature of warmest quarter ( ℃×10) |
|  | Bio11: Mean temperature of coldest quarter ( ℃×10) |
| Precipitation  (period 1970-2000) | Bio12: Annual precipitation (mm) |
|  | Bio13: Precipitation of wettest month (mm) |
|  | Bio14: Precipitation of driest month (mm) |
|  | Bio15: Precipitation seasonality (coefficient of variation) |
|  | Bio16: Precipitation of wettest quarter (mm) |
|  | Bio17: Precipitation of driest quarter (mm) |
|  | Bio18: Precipitation of warmest quarter (mm) |
|  | Bio19: Precipitation of coldest quarter (mm) |
| Solar radiation  ( period 1970-2000) | Sr1: Solar radiation in January ( KJ m^-2^ day^-1^) |
|  | Sr2: Solar radiation in February ( KJ m^-2^ day^-1^) |
|  | Sr3: Solar radiation in March ( KJ m^-2^ day^-1^) |
|  | Sr4: Solar radiation in April ( KJ m^-2^ day^-1^) |
|  | Sr5: Solar radiation in May ( KJ m^-2^ day^-1^) |
|  | Sr6: Solar radiation in June ( KJ m^-2^ day^-1^) |
|  | Sr7: Solar radiation in July ( KJ m^-2^ day^-1^) |
|  | Sr8: Solar radiation in August ( KJ m^-2^ day^-1^) |
|  | Sr9: Solar radiation in September ( KJ m^-2^ day^-1^) |
|  | Sr10: Solar radiation in October ( KJ m^-2^ day^-1^) |
|  | Sr11: Solar radiation in November ( KJ m^-2^ day^-1^) |
|  | Sr12: Solar radiation in December ( KJ m^-2^ day^-1^) |
| Water vapor pressure  ( period 1970-2000) | Wvp1: Water vapor pressure in January(kPa) |
|  | Wvp2: Water vapor pressure in February (kPa) |
|  | Wvp3: Water vapor pressure in March (kPa) |
|  | Wvp4: Water vapor pressure in April (kPa) |
|  | Wvp5: Water vapor pressure in May (kPa) |
|  | Wvp6: Water vapor pressure in June (kPa) |
|  | Wvp7: Water vapor pressure in July (kPa) |
|  | Wvp8: Water vapor pressure in August (kPa) |
|  | Wvp9: Water vapor pressure in September (kPa) |
|  | Wvp10: Water vapor pressure in October (kPa) |
|  | Wvp11: Water vapor pressure in November (kPa) |
|  | Wvp12: Water vapor pressure in December (kPa) |
