# Supplementary material for: Adaptive genetic differentiation in Pterocarya stenoptera (Juglandaceae) driven by multiple environmental variables were revealed by landscape genomics
Source: BMC Plant Biol. 2018 Nov 27;18:306. doi: 10.1186/s12870-018-1524-x (PMC6260741; doi:10.1186/s12870-018-1524-x)
Supplement: Supplementary file 3 — Environmental variables for each location from the WorldClim database. (DOCX 26 kb) [file 12870_2018_1524_MOESM3_ESM.docx]

**Additional file 3** Environmental variables for each location from the WorldClim database.

|  | 1.JSLM | 2.SCWD | 3.HNJG | 4.AHTZ | 5.ZJTM | 6.AHXN | 7.SCEM | 8.JXSQ | 9.JXLH | 10.GZFJ | 11.YNYB | 12.SDTM | 13.SDMM | 14.HNNZ | 15.HNXC | 16.SXWZ | 17.JSBH | 18.HBSN | 19.HBJG | 20.HNTM | 21.FJWY | 22.HNHS |
| --- | --- | --- | --- | --- | --- | --- | --- | --- | --- | --- | --- | --- | --- | --- | --- | --- | --- | --- | --- | --- | --- | --- |
| **Bio1** | 15.433 | 15.702 | 14.830 | 16.027 | 15.347 | 16.523 | 16.319 | 16.698 | 18.244 | 15.146 | 15.048 | 11.789 | 12.676 | 14.005 | 15.168 | 13.956 | 15.237 | 11.925 | 15.705 | 16.823 | 18.411 | 17.120 |
| **Bio2** | 7.373 | 7.830 | 8.948 | 8.411 | 8.482 | 8.671 | 7.105 | 8.886 | 8.769 | 7.244 | 10.393 | 8.979 | 10.518 | 10.308 | 9.642 | 8.922 | 8.339 | 9.063 | 7.932 | 7.613 | 9.206 | 7.957 |
| **Bio3** | 23.225 | 27.877 | 26.664 | 26.225 | 25.836 | 26.536 | 26.386 | 28.287 | 27.643 | 25.594 | 44.706 | 24.844 | 27.527 | 29.862 | 28.245 | 28.274 | 24.859 | 30.077 | 25.313 | 25.152 | 30.825 | 25.478 |
| **Bio4** | 890.588 | 738.489 | 885.891 | 875.598 | 883.400 | 884.508 | 710.210 | 820.083 | 828.519 | 757.243 | 459.060 | 994.197 | 1016.157 | 887.434 | 898.903 | 835.972 | 924.088 | 754.817 | 833.512 | 819.364 | 746.864 | 839.313 |
| **Bio5** | 31.384 | 28.728 | 31.120 | 32.332 | 31.936 | 33.040 | 28.912 | 32.556 | 34.160 | 28.776 | 25.764 | 28.752 | 30.608 | 30.548 | 31.908 | 28.984 | 32.036 | 26.520 | 31.308 | 31.404 | 33.256 | 32.604 |
| **Bio6** | -0.364 | 0.640 | -2.440 | 0.260 | -0.896 | 0.364 | 1.984 | 1.144 | 2.436 | 0.472 | 2.516 | -7.388 | -7.600 | -3.972 | -2.228 | -2.572 | -1.508 | -3.612 | -0.028 | 1.136 | 3.392 | 1.372 |
| **Bio7** | 31.748 | 28.088 | 33.560 | 32.072 | 32.832 | 32.676 | 26.928 | 31.412 | 31.724 | 28.304 | 23.248 | 36.140 | 38.208 | 34.520 | 34.136 | 31.556 | 33.544 | 30.132 | 31.336 | 30.268 | 29.864 | 31.232 |
| **Bio8** | 26.079 | 23.601 | 25.513 | 26.641 | 23.652 | 20.629 | 24.113 | 20.601 | 22.177 | 22.431 | 19.942 | 23.471 | 24.656 | 23.281 | 24.688 | 23.217 | 26.393 | 21.016 | 20.055 | 24.575 | 21.995 | 21.055 |
| **Bio9** | 4.332 | 6.142 | 3.519 | 5.143 | 6.361 | 7.315 | 7.025 | 8.197 | 14.083 | 5.594 | 8.822 | -1.188 | -0.538 | 2.679 | 3.866 | 3.473 | 3.654 | 2.281 | 7.000 | 6.471 | 14.651 | 8.562 |
| **Bio10** | 26.079 | 24.527 | 25.513 | 26.641 | 26.046 | 27.101 | 24.715 | 26.396 | 28.035 | 24.286 | 19.942 | 23.471 | 24.656 | 24.817 | 26.262 | 24.128 | 26.393 | 21.016 | 25.749 | 26.645 | 27.021 | 27.159 |
| **Bio11** | 4.332 | 6.142 | 3.519 | 5.143 | 4.291 | 5.361 | 7.025 | 6.258 | 7.711 | 5.594 | 8.822 | -1.188 | -0.538 | 2.679 | 3.866 | 3.473 | 3.654 | 2.281 | 5.139 | 6.471 | 8.783 | 6.567 |
| **Bio12** | 1021 | 916 | 1141 | 1502 | 1383 | 1607 | 1515 | 1787 | 1783 | 1222 | 887 | 749 | 763 | 805 | 839 | 876 | 1027 | 1204 | 1543 | 1391 | 1744 | 1517 |
| **Bio13** | 170 | 224 | 207 | 271 | 220 | 277 | 375 | 314 | 332 | 200 | 149 | 234 | 234 | 156 | 155 | 157 | 196 | 206 | 266 | 238 | 328 | 220 |
| **Bio14** | 31 | 4 | 20 | 32 | 37 | 43 | 18 | 47 | 50 | 28 | 17 | 6 | 8 | 9 | 10 | 8 | 25 | 18 | 40 | 31 | 45 | 50 |
| **Bio15** | 54.791 | 100.091 | 59.006 | 55.201 | 48.132 | 54.956 | 95.320 | 58.009 | 62.928 | 59.646 | 64.425 | 111.628 | 108.811 | 69.759 | 68.210 | 71.251 | 59.773 | 64.073 | 56.186 | 58.837 | 65.549 | 45.109 |
| **Bio16** | 443 | 567 | 501 | 624 | 547 | 713 | 891 | 826 | 865 | 540 | 419 | 487 | 497 | 390 | 396 | 423 | 472 | 526 | 694 | 623 | 867 | 637 |
| **Bio17** | 114 | 18 | 91 | 136 | 143 | 161 | 60 | 181 | 180 | 89 | 67 | 23 | 30 | 37 | 37 | 31 | 100 | 64 | 158 | 110 | 170 | 202 |
| **Bio18** | 443 | 536 | 501 | 624 | 512 | 551 | 890 | 588 | 589 | 477 | 419 | 487 | 497 | 363 | 366 | 375 | 472 | 526 | 554 | 567 | 568 | 446 |
| **Bio19** | 114 | 18 | 91 | 136 | 167 | 201 | 60 | 236 | 242 | 89 | 67 | 23 | 30 | 37 | 37 | 31 | 100 | 64 | 176 | 110 | 226 | 216 |
| **Sr1** | 9558 | 6627 | 8422 | 9531 | 9534 | 9517 | 6321 | 9530 | 9621 | 7235 | 13687 | 10354 | 10405 | 9082 | 8688 | 8378 | 9856 | 8101 | 8531 | 7685 | 9768 | 8464 |
| **Sr2** | 10924 | 7995 | 10209 | 10785 | 10586 | 10563 | 7915 | 10379 | 10298 | 8545 | 15556 | 13051 | 13032 | 10757 | 10342 | 9839 | 11159 | 9779 | 9410 | 9160 | 10518 | 9210 |
| **Sr3** | 13871 | 10566 | 12544 | 13223 | 13135 | 12859 | 10751 | 12370 | 12101 | 11069 | 18131 | 16177 | 16011 | 13455 | 13094 | 12456 | 14114 | 12337 | 11721 | 11591 | 12116 | 11103 |
| **Sr4** | 16531 | 13745 | 15409 | 15574 | 15538 | 15274 | 13882 | 14868 | 14629 | 13769 | 19216 | 19417 | 18970 | 16076 | 15687 | 15259 | 16543 | 14944 | 14219 | 14170 | 14667 | 13455 |
| **Sr5** | 18506 | 15799 | 17606 | 17749 | 17594 | 17291 | 15244 | 16657 | 16270 | 14730 | 18210 | 22278 | 21699 | 18711 | 18168 | 17260 | 18902 | 16756 | 16147 | 15559 | 16209 | 15058 |
| **Sr6** | 18600 | 16533 | 18941 | 18664 | 17909 | 17781 | 15031 | 17262 | 17215 | 16002 | 15596 | 23393 | 22767 | 20337 | 19810 | 19104 | 19325 | 18131 | 16941 | 16805 | 17142 | 16021 |
| **Sr7** | 20654 | 16772 | 19825 | 21259 | 21158 | 21498 | 15103 | 21675 | 22552 | 19250 | 13832 | 20685 | 20222 | 19639 | 19304 | 19778 | 20721 | 19064 | 21094 | 19639 | 22072 | 21834 |
| **Sr8** | 20659 | 16152 | 19149 | 20705 | 20989 | 20951 | 14996 | 20831 | 21055 | 19056 | 14835 | 19269 | 19439 | 18797 | 18620 | 19303 | 20859 | 19435 | 19832 | 19718 | 20754 | 20657 |
| **Sr9** | 16582 | 11555 | 14968 | 16525 | 16624 | 17247 | 11467 | 17428 | 17839 | 15719 | 14763 | 17085 | 16978 | 14434 | 14104 | 13538 | 16521 | 14765 | 16305 | 16117 | 17868 | 17101 |
| **Sr10** | 13780 | 8702 | 11964 | 13558 | 13793 | 13940 | 8558 | 13882 | 14130 | 10704 | 12906 | 14230 | 14346 | 12016 | 11535 | 10465 | 14013 | 11317 | 12743 | 11597 | 14157 | 12817 |
| **Sr11** | 10535 | 7160 | 9302 | 10445 | 10682 | 10736 | 7351 | 10983 | 11140 | 8368 | 13498 | 10741 | 10820 | 9206 | 8910 | 8024 | 10813 | 8788 | 9933 | 8956 | 11243 | 9998 |
| **Sr12** | 8908 | 6026 | 7853 | 8959 | 9125 | 9129 | 5926 | 9317 | 9491 | 7149 | 12584 | 9284 | 9407 | 8232 | 8064 | 7360 | 9277 | 7666 | 8383 | 7568 | 9685 | 8571 |
| **Wvp1** | 0.553 | 0.625 | 0.463 | 0.550 | 0.586 | 0.639 | 0.633 | 0.649 | 0.732 | 0.626 | 0.651 | 0.240 | 0.290 | 0.339 | 0.403 | 0.474 | 0.514 | 0.479 | 0.537 | 0.638 | 0.757 | 0.713 |
| **Wvp2** | 0.600 | 0.708 | 0.539 | 0.621 | 0.663 | 0.728 | 0.718 | 0.753 | 0.843 | 0.696 | 0.685 | 0.291 | 0.344 | 0.412 | 0.461 | 0.538 | 0.583 | 0.530 | 0.628 | 0.712 | 0.878 | 0.820 |
| **Wvp3** | 0.812 | 0.900 | 0.768 | 0.854 | 0.902 | 0.980 | 0.888 | 1.018 | 1.127 | 0.922 | 0.789 | 0.432 | 0.486 | 0.608 | 0.663 | 0.709 | 0.798 | 0.719 | 0.862 | 0.943 | 1.162 | 1.092 |
| **Wvp4** | 1.200 | 1.262 | 1.208 | 1.276 | 1.320 | 1.434 | 1.216 | 1.458 | 1.603 | 1.369 | 1.024 | 0.725 | 0.799 | 0.968 | 1.030 | 1.056 | 1.200 | 1.054 | 1.279 | 1.422 | 1.607 | 1.601 |
| **Wvp5** | 1.660 | 1.648 | 1.652 | 1.769 | 1.801 | 1.942 | 1.520 | 1.930 | 2.107 | 1.789 | 1.409 | 1.146 | 1.253 | 1.377 | 1.459 | 1.434 | 1.678 | 1.420 | 1.723 | 1.894 | 2.070 | 2.130 |
| **Wvp6** | 2.300 | 2.096 | 2.189 | 2.335 | 2.406 | 2.522 | 1.875 | 2.440 | 2.614 | 2.238 | 1.868 | 1.608 | 1.784 | 1.822 | 1.906 | 1.857 | 2.295 | 1.811 | 2.226 | 2.418 | 2.542 | 2.656 |
| **Wvp7** | 3.038 | 2.519 | 2.729 | 2.894 | 2.905 | 2.959 | 2.108 | 2.753 | 2.918 | 2.540 | 2.030 | 2.301 | 2.527 | 2.371 | 2.482 | 2.291 | 2.976 | 2.182 | 2.573 | 2.827 | 2.814 | 2.960 |
| **Wvp8** | 2.976 | 2.440 | 2.613 | 2.806 | 2.824 | 2.884 | 2.071 | 2.698 | 2.863 | 2.464 | 2.010 | 2.192 | 2.408 | 2.310 | 2.381 | 2.192 | 2.889 | 2.060 | 2.524 | 2.715 | 2.750 | 2.923 |
| **Wvp9** | 2.229 | 1.968 | 1.858 | 2.078 | 2.222 | 2.280 | 1.715 | 2.207 | 2.366 | 1.941 | 1.831 | 1.440 | 1.631 | 1.644 | 1.737 | 1.724 | 2.150 | 1.620 | 1.943 | 2.066 | 2.326 | 2.322 |
| **Wvp10** | 1.524 | 1.460 | 1.252 | 1.402 | 1.520 | 1.558 | 1.347 | 1.523 | 1.663 | 1.425 | 1.470 | 0.872 | 1.006 | 1.074 | 1.169 | 1.214 | 1.438 | 1.158 | 1.328 | 1.490 | 1.674 | 1.638 |
| **Wvp11** | 0.997 | 1.007 | 0.796 | 0.904 | 1.002 | 1.040 | 0.974 | 1.036 | 1.138 | 1.003 | 1.026 | 0.485 | 0.578 | 0.656 | 0.736 | 0.800 | 0.923 | 0.799 | 0.866 | 1.038 | 1.170 | 1.116 |
| **Wvp12** | 0.640 | 0.698 | 0.516 | 0.607 | 0.654 | 0.700 | 0.725 | 0.702 | 0.784 | 0.708 | 0.760 | 0.296 | 0.354 | 0.405 | 0.471 | 0.546 | 0.582 | 0.554 | 0.586 | 0.730 | 0.817 | 0.772 |
