# Supplementary material for: Adaptive genetic differentiation in Pterocarya stenoptera (Juglandaceae) driven by multiple environmental variables were revealed by landscape genomics
Source: BMC Plant Biol. 2018 Nov 27;18:306. doi: 10.1186/s12870-018-1524-x (PMC6260741; doi:10.1186/s12870-018-1524-x)
Supplement: Supplementary file 4 — Allele frequencies of 1006 alleles for each population. (DOCX 253 kb) [file 12870_2018_1524_MOESM4_ESM.docx]

**Additional file 4** Allele frequencies of 1006 alleles for each population.

|  | Allele frequency | | | | | | | | | | | | | | | | | | | | | |
| --- | --- | --- | --- | --- | --- | --- | --- | --- | --- | --- | --- | --- | --- | --- | --- | --- | --- | --- | --- | --- | --- | --- |
| Locus | 1.JSLM | 2.SCWD | 3.HNJG | 4.AHTZ | 5.ZJTM | 6.AHXN | 7.SCEM | 8.JXSQ | 9.JXLH | 10.GZFJ | 11.YNYB | 12.SDTM | 13.SDMM | 14.HNNZ | 15.HNXC | 16.SXWZ | 17.JSBH | 18.HBSN | 19.HBJG | 20.HNTM | 21.FJWY | 22.HNHM |
| 5-116 | 0.000 | 0.000 | 0.000 | 0.000 | 0.000 | 0.000 | 0.000 | 0.000 | 0.000 | 0.000 | 0.000 | 0.000 | 0.000 | 0.043 | 0.000 | 0.000 | 0.000 | 0.000 | 0.048 | 0.000 | 0.000 | 0.000 |
| 5-130 | 0.714 | 1.000 | 0.095 | 0.947 | 0.950 | 0.722 | 0.913 | 0.214 | 0.000 | 0.000 | 0.000 | 0.875 | 1.000 | 0.826 | 0.900 | 0.000 | 0.100 | 0.409 | 0.143 | 1.000 | 0.381 | 0.957 |
| 5-147 | 0.000 | 0.000 | 0.000 | 0.000 | 0.000 | 0.000 | 0.000 | 0.000 | 0.000 | 0.000 | 0.000 | 0.125 | 0.208 | 0.043 | 0.000 | 0.000 | 0.000 | 0.000 | 0.000 | 0.000 | 0.000 | 0.000 |
| 5-152 | 0.000 | 0.000 | 0.048 | 0.000 | 0.000 | 0.000 | 0.000 | 0.000 | 0.000 | 0.000 | 0.000 | 0.042 | 0.000 | 0.000 | 0.000 | 0.000 | 0.000 | 0.000 | 0.000 | 0.000 | 0.000 | 0.000 |
| 5-158 | 0.000 | 0.739 | 0.000 | 0.053 | 0.000 | 0.111 | 0.478 | 0.000 | 0.067 | 0.000 | 0.000 | 0.208 | 0.292 | 0.000 | 0.500 | 0.000 | 0.050 | 0.000 | 0.000 | 0.542 | 0.000 | 0.043 |
| 5-168 | 0.000 | 0.000 | 0.000 | 0.000 | 0.000 | 0.000 | 0.000 | 0.000 | 0.000 | 0.000 | 0.000 | 0.125 | 0.000 | 0.043 | 0.000 | 0.000 | 0.000 | 0.000 | 0.000 | 0.000 | 0.000 | 0.000 |
| 5-179 | 0.000 | 0.000 | 0.000 | 0.000 | 0.000 | 0.000 | 0.000 | 0.000 | 0.000 | 0.000 | 0.000 | 0.000 | 0.000 | 0.130 | 0.050 | 0.000 | 0.000 | 0.000 | 0.000 | 0.000 | 0.095 | 0.000 |
| 5-183 | 0.000 | 0.000 | 0.000 | 0.000 | 0.000 | 0.000 | 0.000 | 0.000 | 0.000 | 0.000 | 0.000 | 0.250 | 0.208 | 0.043 | 0.100 | 0.000 | 0.000 | 0.000 | 0.000 | 0.000 | 0.000 | 0.000 |
| 5-190 | 0.000 | 0.000 | 0.000 | 0.000 | 0.000 | 0.000 | 0.000 | 0.000 | 0.000 | 0.000 | 0.000 | 0.167 | 0.000 | 0.043 | 0.200 | 0.000 | 0.000 | 0.000 | 0.000 | 0.000 | 0.000 | 0.000 |
| 5-196 | 0.000 | 0.522 | 0.000 | 0.000 | 0.000 | 0.000 | 0.087 | 0.000 | 0.067 | 0.000 | 0.000 | 0.542 | 0.750 | 0.217 | 0.550 | 0.000 | 0.000 | 0.000 | 0.095 | 0.458 | 0.095 | 0.565 |
| 5-208 | 0.000 | 0.000 | 0.000 | 0.000 | 0.000 | 0.000 | 0.000 | 0.000 | 0.000 | 0.000 | 0.000 | 0.000 | 0.000 | 0.043 | 0.200 | 0.000 | 0.000 | 0.000 | 0.000 | 0.000 | 0.000 | 0.000 |
| 5-213 | 0.000 | 0.000 | 0.000 | 0.000 | 0.000 | 0.000 | 0.174 | 0.000 | 0.000 | 0.000 | 0.000 | 0.208 | 0.375 | 0.043 | 0.200 | 0.000 | 0.000 | 0.000 | 0.000 | 0.208 | 0.143 | 0.087 |
| 5-217 | 0.000 | 0.000 | 0.000 | 0.000 | 0.000 | 0.000 | 0.261 | 0.000 | 0.000 | 0.000 | 0.000 | 0.208 | 0.375 | 0.087 | 0.200 | 0.000 | 0.000 | 0.000 | 0.000 | 0.208 | 0.000 | 0.087 |
| 5-226 | 0.000 | 0.000 | 0.000 | 0.000 | 0.000 | 0.000 | 0.000 | 0.000 | 0.000 | 0.000 | 0.000 | 0.458 | 0.000 | 0.957 | 0.300 | 0.000 | 0.000 | 0.000 | 0.143 | 0.000 | 0.286 | 0.000 |
| 5-230 | 0.000 | 0.000 | 0.000 | 0.000 | 0.000 | 0.000 | 0.000 | 0.000 | 0.000 | 0.000 | 0.000 | 0.417 | 0.125 | 0.913 | 0.350 | 0.000 | 0.000 | 0.091 | 0.143 | 0.000 | 0.190 | 0.000 |
| 5-240 | 0.000 | 0.000 | 0.000 | 0.000 | 0.000 | 0.000 | 0.000 | 0.000 | 0.000 | 0.000 | 0.000 | 0.042 | 0.083 | 0.000 | 0.000 | 0.000 | 0.000 | 0.000 | 0.000 | 0.000 | 0.000 | 0.000 |
| 5-246 | 0.000 | 0.043 | 0.000 | 0.000 | 0.000 | 0.000 | 0.000 | 0.000 | 0.000 | 0.000 | 0.000 | 0.125 | 0.000 | 0.043 | 0.150 | 0.000 | 0.000 | 0.000 | 0.000 | 0.042 | 0.000 | 0.000 |
| 5-256 | 0.000 | 0.087 | 0.000 | 0.000 | 0.000 | 0.000 | 0.087 | 0.000 | 0.000 | 0.000 | 0.000 | 0.458 | 0.042 | 0.565 | 0.400 | 0.000 | 0.000 | 0.182 | 0.048 | 0.208 | 0.238 | 0.000 |
| 5-262 | 0.048 | 0.043 | 0.000 | 0.000 | 0.200 | 0.000 | 0.043 | 0.000 | 0.000 | 0.000 | 0.000 | 0.042 | 0.083 | 0.087 | 0.100 | 0.667 | 0.050 | 0.091 | 0.048 | 0.000 | 0.000 | 0.217 |
| 5-266 | 0.095 | 0.087 | 0.000 | 0.000 | 0.300 | 0.000 | 0.043 | 0.000 | 0.000 | 0.000 | 0.000 | 0.167 | 0.250 | 0.087 | 0.200 | 0.556 | 0.050 | 0.000 | 0.048 | 0.000 | 0.000 | 0.348 |
| 5-274 | 0.000 | 0.304 | 0.190 | 0.000 | 0.100 | 0.000 | 0.000 | 0.000 | 0.000 | 0.000 | 0.048 | 0.708 | 0.458 | 0.826 | 0.800 | 0.000 | 0.250 | 0.182 | 0.286 | 0.167 | 0.333 | 0.043 |
| 5-277 | 0.000 | 0.043 | 0.000 | 0.000 | 0.000 | 0.000 | 0.000 | 0.000 | 0.000 | 0.000 | 0.095 | 0.667 | 0.333 | 0.348 | 0.300 | 0.000 | 0.100 | 0.045 | 0.143 | 0.083 | 0.238 | 0.043 |
| 5-284 | 0.000 | 0.000 | 0.000 | 0.000 | 0.000 | 0.000 | 0.087 | 0.000 | 0.000 | 0.071 | 0.000 | 0.125 | 0.000 | 0.000 | 0.250 | 0.000 | 0.000 | 0.045 | 0.000 | 0.000 | 0.048 | 0.000 |
| 5-289 | 0.000 | 0.043 | 0.000 | 0.000 | 0.000 | 0.000 | 0.000 | 0.000 | 0.000 | 0.000 | 0.000 | 0.500 | 0.000 | 0.913 | 0.450 | 0.000 | 0.000 | 0.091 | 0.381 | 0.000 | 0.333 | 0.000 |
| 5-292 | 0.048 | 0.000 | 0.000 | 0.000 | 0.000 | 0.000 | 0.000 | 0.071 | 0.000 | 0.000 | 0.000 | 0.500 | 0.000 | 0.957 | 0.450 | 0.000 | 0.000 | 0.273 | 0.333 | 0.042 | 0.524 | 0.000 |
| 5-300 | 0.000 | 0.000 | 0.000 | 0.000 | 0.000 | 0.000 | 0.000 | 0.000 | 0.000 | 0.000 | 0.000 | 0.208 | 0.000 | 0.391 | 0.300 | 0.000 | 0.000 | 0.000 | 0.000 | 0.000 | 0.000 | 0.000 |
| 5-306 | 0.000 | 0.000 | 0.000 | 0.000 | 0.000 | 0.056 | 0.000 | 0.071 | 0.000 | 0.000 | 0.000 | 0.792 | 0.417 | 0.435 | 0.500 | 0.000 | 0.050 | 0.136 | 0.143 | 0.042 | 0.571 | 0.000 |
| 5-319 | 0.000 | 0.000 | 0.000 | 0.000 | 0.000 | 0.000 | 0.000 | 0.000 | 0.000 | 0.000 | 0.000 | 0.625 | 0.042 | 0.957 | 0.700 | 0.000 | 0.000 | 0.227 | 0.238 | 0.042 | 0.333 | 0.000 |
| 5-323 | 0.000 | 0.043 | 0.000 | 0.000 | 0.000 | 0.000 | 0.000 | 0.000 | 0.067 | 0.000 | 0.000 | 0.083 | 0.083 | 0.261 | 0.450 | 0.000 | 0.000 | 0.091 | 0.000 | 0.000 | 0.095 | 0.000 |
| 5-329 | 0.000 | 0.130 | 0.000 | 0.000 | 0.000 | 0.000 | 0.000 | 0.000 | 0.067 | 0.000 | 0.000 | 0.042 | 0.042 | 0.087 | 0.300 | 0.000 | 0.000 | 0.045 | 0.048 | 0.208 | 0.048 | 0.261 |
| 5-337 | 0.048 | 0.000 | 0.000 | 0.000 | 0.000 | 0.000 | 0.000 | 0.000 | 0.000 | 0.000 | 0.000 | 0.167 | 0.125 | 0.435 | 0.450 | 0.000 | 0.000 | 0.182 | 0.000 | 0.042 | 0.048 | 0.217 |
| 5-345 | 0.048 | 0.174 | 0.000 | 0.000 | 0.000 | 0.000 | 0.043 | 0.000 | 0.067 | 0.000 | 0.000 | 0.208 | 0.375 | 0.087 | 0.250 | 0.000 | 0.000 | 0.000 | 0.000 | 0.000 | 0.000 | 0.043 |
| 5-350 | 0.000 | 0.000 | 0.000 | 0.000 | 0.000 | 0.000 | 0.000 | 0.000 | 0.000 | 0.000 | 0.000 | 0.000 | 0.000 | 0.043 | 0.050 | 0.000 | 0.000 | 0.000 | 0.000 | 0.000 | 0.000 | 0.000 |
| 5-356 | 0.048 | 0.000 | 0.000 | 0.000 | 0.000 | 0.000 | 0.000 | 0.000 | 0.000 | 0.000 | 0.000 | 0.042 | 0.125 | 0.000 | 0.400 | 0.000 | 0.000 | 0.000 | 0.000 | 0.000 | 0.048 | 0.000 |
| 5-367 | 0.000 | 0.000 | 0.000 | 0.000 | 0.000 | 0.000 | 0.000 | 0.000 | 0.000 | 0.000 | 0.000 | 0.042 | 0.083 | 0.174 | 0.100 | 0.000 | 0.000 | 0.000 | 0.095 | 0.042 | 0.000 | 0.000 |
| 5-373 | 0.857 | 0.957 | 0.429 | 0.895 | 0.500 | 0.889 | 1.000 | 0.643 | 0.667 | 0.786 | 0.429 | 0.500 | 0.917 | 0.087 | 0.550 | 0.833 | 0.600 | 0.455 | 0.048 | 1.000 | 0.429 | 1.000 |
| 5-377 | 0.048 | 0.174 | 0.000 | 0.000 | 0.000 | 0.000 | 0.130 | 0.000 | 0.000 | 0.000 | 0.000 | 0.042 | 0.375 | 0.174 | 0.150 | 0.000 | 0.050 | 0.000 | 0.048 | 0.125 | 0.000 | 0.087 |
| 5-382 | 0.000 | 0.087 | 0.000 | 0.000 | 0.000 | 0.000 | 0.087 | 0.143 | 0.000 | 0.000 | 0.000 | 0.292 | 0.208 | 0.043 | 0.100 | 0.000 | 0.000 | 0.000 | 0.000 | 0.042 | 0.095 | 0.000 |
| 5-387 | 0.000 | 0.087 | 0.000 | 0.158 | 0.000 | 0.000 | 0.000 | 0.000 | 0.000 | 0.000 | 0.000 | 0.333 | 0.000 | 0.261 | 0.200 | 0.000 | 0.000 | 0.000 | 0.000 | 0.083 | 0.238 | 0.000 |
| 5-391 | 0.095 | 0.087 | 0.000 | 0.000 | 0.000 | 0.000 | 0.130 | 0.000 | 0.000 | 0.000 | 0.048 | 0.292 | 0.292 | 0.217 | 0.400 | 0.056 | 0.000 | 0.045 | 0.048 | 0.167 | 0.095 | 0.043 |
| 5-393 | 0.000 | 0.000 | 0.048 | 0.000 | 0.050 | 0.000 | 0.000 | 0.000 | 0.067 | 0.000 | 0.000 | 0.000 | 0.000 | 0.261 | 0.400 | 0.000 | 0.000 | 0.000 | 0.095 | 0.042 | 0.048 | 0.000 |
| 5-396 | 0.000 | 0.000 | 0.000 | 0.000 | 0.000 | 0.000 | 0.000 | 0.000 | 0.000 | 0.000 | 0.000 | 0.000 | 0.000 | 0.217 | 0.200 | 0.000 | 0.000 | 0.000 | 0.000 | 0.000 | 0.000 | 0.000 |
| 5-404 | 0.000 | 0.000 | 0.000 | 0.000 | 0.000 | 0.000 | 0.000 | 0.000 | 0.000 | 0.000 | 0.000 | 0.000 | 0.000 | 0.000 | 0.100 | 0.000 | 0.000 | 0.000 | 0.000 | 0.000 | 0.000 | 0.000 |
| 5-411 | 0.000 | 0.000 | 0.000 | 0.000 | 0.000 | 0.056 | 0.087 | 0.000 | 0.000 | 0.000 | 0.000 | 0.000 | 0.000 | 0.043 | 0.100 | 0.000 | 0.000 | 0.000 | 0.000 | 0.042 | 0.000 | 0.000 |
| 5-418 | 0.000 | 0.000 | 0.000 | 0.000 | 0.000 | 0.000 | 0.000 | 0.000 | 0.000 | 0.000 | 0.000 | 0.000 | 0.042 | 0.130 | 0.100 | 0.000 | 0.000 | 0.045 | 0.000 | 0.000 | 0.095 | 0.087 |
| 5-423 | 0.000 | 0.000 | 0.000 | 0.000 | 0.000 | 0.000 | 0.000 | 0.000 | 0.000 | 0.000 | 0.000 | 0.000 | 0.000 | 0.217 | 0.450 | 0.000 | 0.000 | 0.000 | 0.000 | 0.000 | 0.000 | 0.000 |
| 5-428 | 0.952 | 0.696 | 0.619 | 0.105 | 0.850 | 0.222 | 0.217 | 0.143 | 0.000 | 0.000 | 0.476 | 0.958 | 0.708 | 1.000 | 0.950 | 0.389 | 0.700 | 0.818 | 0.714 | 0.792 | 0.667 | 0.739 |
| 5-432 | 0.952 | 0.000 | 0.000 | 0.000 | 0.550 | 0.000 | 0.000 | 0.000 | 0.000 | 0.000 | 0.000 | 0.375 | 0.417 | 0.130 | 0.450 | 0.056 | 0.550 | 0.000 | 0.190 | 0.000 | 0.000 | 0.174 |
| 5-437 | 0.048 | 0.130 | 0.000 | 0.000 | 0.000 | 0.000 | 0.000 | 0.071 | 0.133 | 0.000 | 0.048 | 0.375 | 0.250 | 0.739 | 0.650 | 0.111 | 0.100 | 0.136 | 0.190 | 0.250 | 0.381 | 0.348 |
| 5-441 | 0.000 | 0.000 | 0.000 | 0.000 | 0.000 | 0.000 | 0.000 | 0.000 | 0.000 | 0.000 | 0.000 | 0.000 | 0.000 | 0.087 | 0.000 | 0.000 | 0.000 | 0.000 | 0.000 | 0.000 | 0.000 | 0.000 |
| 5-448 | 0.000 | 0.000 | 0.048 | 0.000 | 0.000 | 0.000 | 0.000 | 0.000 | 0.000 | 0.000 | 0.048 | 0.000 | 0.000 | 0.000 | 0.000 | 0.000 | 0.000 | 0.000 | 0.000 | 0.000 | 0.000 | 0.000 |
| 5-455 | 0.000 | 0.000 | 0.000 | 0.000 | 0.000 | 0.000 | 0.000 | 0.071 | 0.000 | 0.000 | 0.000 | 0.000 | 0.042 | 0.348 | 0.150 | 0.000 | 0.050 | 0.045 | 0.000 | 0.000 | 0.048 | 0.043 |
| 5-459 | 0.000 | 0.000 | 0.000 | 0.000 | 0.000 | 0.000 | 0.000 | 0.000 | 0.000 | 0.000 | 0.000 | 0.000 | 0.000 | 0.130 | 0.150 | 0.000 | 0.000 | 0.000 | 0.000 | 0.000 | 0.000 | 0.000 |
| 5-467 | 0.000 | 0.000 | 0.000 | 0.000 | 0.050 | 0.000 | 0.000 | 0.000 | 0.000 | 0.000 | 0.000 | 0.042 | 0.042 | 0.130 | 0.000 | 0.000 | 0.000 | 0.000 | 0.048 | 0.000 | 0.000 | 0.000 |
| 5-474 | 0.000 | 0.000 | 0.000 | 0.000 | 0.000 | 0.000 | 0.000 | 0.000 | 0.000 | 0.000 | 0.000 | 0.167 | 0.000 | 0.565 | 0.250 | 0.000 | 0.000 | 0.045 | 0.048 | 0.000 | 0.048 | 0.000 |
| 5-479 | 0.000 | 0.000 | 0.000 | 0.000 | 0.000 | 0.000 | 0.043 | 0.000 | 0.000 | 0.000 | 0.000 | 0.375 | 0.000 | 0.348 | 0.250 | 0.000 | 0.000 | 0.000 | 0.238 | 0.000 | 0.143 | 0.000 |
| 5-490 | 0.000 | 0.000 | 0.000 | 0.000 | 0.000 | 0.000 | 0.000 | 0.000 | 0.000 | 0.000 | 0.000 | 0.042 | 0.000 | 0.087 | 0.000 | 0.111 | 0.000 | 0.000 | 0.000 | 0.000 | 0.000 | 0.000 |
| 5-498 | 0.000 | 0.304 | 0.000 | 0.000 | 0.000 | 0.000 | 0.087 | 0.000 | 0.000 | 0.000 | 0.000 | 0.292 | 0.125 | 0.043 | 0.100 | 0.333 | 0.000 | 0.045 | 0.000 | 0.375 | 0.000 | 0.087 |
| 5-500 | 0.000 | 0.043 | 0.000 | 0.000 | 0.000 | 0.000 | 0.000 | 0.000 | 0.000 | 0.000 | 0.000 | 0.125 | 0.042 | 0.000 | 0.050 | 0.000 | 0.000 | 0.000 | 0.000 | 0.000 | 0.095 | 0.000 |
| 5-506 | 0.000 | 0.043 | 0.000 | 0.000 | 0.000 | 0.000 | 0.043 | 0.000 | 0.000 | 0.071 | 0.000 | 0.000 | 0.000 | 0.000 | 0.000 | 0.000 | 0.000 | 0.000 | 0.000 | 0.000 | 0.048 | 0.000 |
| 5-512 | 0.000 | 0.000 | 0.000 | 0.000 | 0.000 | 0.000 | 0.000 | 0.000 | 0.000 | 0.000 | 0.000 | 0.000 | 0.000 | 0.087 | 0.200 | 0.000 | 0.000 | 0.000 | 0.000 | 0.000 | 0.000 | 0.000 |
| 5-516 | 0.000 | 0.000 | 0.000 | 0.000 | 0.000 | 0.000 | 0.000 | 0.000 | 0.000 | 0.000 | 0.000 | 0.042 | 0.000 | 0.652 | 0.300 | 0.000 | 0.000 | 0.227 | 0.095 | 0.000 | 0.143 | 0.000 |
| 5-521 | 0.000 | 0.000 | 0.000 | 0.000 | 0.000 | 0.000 | 0.000 | 0.000 | 0.000 | 0.000 | 0.000 | 0.042 | 0.000 | 0.348 | 0.150 | 0.000 | 0.000 | 0.000 | 0.000 | 0.000 | 0.000 | 0.000 |
| 5-527 | 0.000 | 0.087 | 0.238 | 0.053 | 0.150 | 0.056 | 0.000 | 0.357 | 0.000 | 0.000 | 0.000 | 0.042 | 0.083 | 0.174 | 0.450 | 0.000 | 0.050 | 0.000 | 0.095 | 0.333 | 0.000 | 0.000 |
| 5-533 | 0.000 | 0.000 | 0.000 | 0.000 | 0.000 | 0.000 | 0.000 | 0.000 | 0.000 | 0.000 | 0.000 | 0.125 | 0.042 | 0.087 | 0.200 | 0.000 | 0.000 | 0.000 | 0.000 | 0.000 | 0.000 | 0.000 |
| 5-539 | 0.000 | 0.000 | 0.000 | 0.000 | 0.050 | 0.000 | 0.000 | 0.071 | 0.133 | 0.000 | 0.048 | 0.167 | 0.042 | 0.261 | 0.300 | 0.000 | 0.000 | 0.000 | 0.000 | 0.000 | 0.143 | 0.304 |
| 5-543 | 0.000 | 0.000 | 0.000 | 0.000 | 0.000 | 0.000 | 0.000 | 0.071 | 0.000 | 0.000 | 0.000 | 0.000 | 0.042 | 0.217 | 0.050 | 0.000 | 0.000 | 0.000 | 0.000 | 0.000 | 0.048 | 0.000 |
| 5-549 | 0.000 | 0.043 | 0.000 | 0.000 | 0.000 | 0.000 | 0.000 | 0.000 | 0.000 | 0.000 | 0.000 | 0.000 | 0.000 | 0.087 | 0.100 | 0.000 | 0.000 | 0.000 | 0.000 | 0.000 | 0.000 | 0.000 |
| 5-557 | 0.000 | 0.000 | 0.000 | 0.000 | 0.000 | 0.000 | 0.000 | 0.000 | 0.000 | 0.000 | 0.000 | 0.000 | 0.000 | 0.174 | 0.200 | 0.000 | 0.000 | 0.000 | 0.000 | 0.000 | 0.000 | 0.000 |
| 5-562 | 0.000 | 0.000 | 0.000 | 0.000 | 0.000 | 0.000 | 0.000 | 0.000 | 0.000 | 0.000 | 0.000 | 0.083 | 0.000 | 0.304 | 0.150 | 0.000 | 0.000 | 0.000 | 0.143 | 0.000 | 0.238 | 0.000 |
| 5-570 | 0.095 | 0.043 | 0.000 | 0.000 | 0.150 | 0.000 | 0.000 | 0.000 | 0.000 | 0.071 | 0.000 | 0.000 | 0.167 | 0.043 | 0.150 | 0.056 | 0.100 | 0.000 | 0.000 | 0.083 | 0.048 | 0.000 |
| 5-577 | 0.000 | 0.000 | 0.000 | 0.000 | 0.000 | 0.000 | 0.000 | 0.000 | 0.000 | 0.000 | 0.048 | 0.292 | 0.000 | 0.826 | 0.350 | 0.000 | 0.000 | 0.227 | 0.333 | 0.000 | 0.476 | 0.000 |
| 5-580 | 0.000 | 0.000 | 0.000 | 0.000 | 0.000 | 0.000 | 0.000 | 0.000 | 0.000 | 0.000 | 0.000 | 0.083 | 0.000 | 0.478 | 0.350 | 0.000 | 0.000 | 0.000 | 0.048 | 0.042 | 0.000 | 0.000 |
| 5-592 | 0.000 | 0.000 | 0.048 | 0.000 | 0.000 | 0.000 | 0.043 | 0.000 | 0.000 | 0.000 | 0.190 | 0.083 | 0.000 | 0.087 | 0.050 | 0.000 | 0.000 | 0.045 | 0.048 | 0.083 | 0.048 | 0.174 |
| 5-596 | 0.095 | 0.130 | 0.000 | 0.000 | 0.100 | 0.056 | 0.174 | 0.000 | 0.067 | 0.000 | 0.000 | 0.125 | 0.208 | 0.043 | 0.300 | 0.056 | 0.050 | 0.045 | 0.000 | 0.333 | 0.048 | 0.261 |
| 5-607 | 0.000 | 0.000 | 0.000 | 0.000 | 0.000 | 0.000 | 0.043 | 0.000 | 0.000 | 0.000 | 0.000 | 0.000 | 0.000 | 0.043 | 0.000 | 0.000 | 0.000 | 0.000 | 0.000 | 0.000 | 0.000 | 0.000 |
| 5-612 | 0.000 | 0.000 | 0.000 | 0.000 | 0.000 | 0.000 | 0.000 | 0.000 | 0.000 | 0.000 | 0.048 | 0.000 | 0.000 | 0.087 | 0.050 | 0.000 | 0.000 | 0.000 | 0.048 | 0.000 | 0.048 | 0.000 |
| 5-619 | 0.000 | 0.000 | 0.000 | 0.000 | 0.000 | 0.000 | 0.000 | 0.000 | 0.000 | 0.000 | 0.000 | 0.375 | 0.000 | 0.870 | 0.500 | 0.000 | 0.000 | 0.227 | 0.381 | 0.000 | 0.476 | 0.000 |
| 5-624 | 0.000 | 0.000 | 0.000 | 0.000 | 0.000 | 0.000 | 0.000 | 0.000 | 0.000 | 0.000 | 0.000 | 0.042 | 0.000 | 0.304 | 0.250 | 0.000 | 0.000 | 0.000 | 0.048 | 0.042 | 0.048 | 0.000 |
| 5-629 | 0.000 | 0.000 | 0.000 | 0.000 | 0.000 | 0.000 | 0.000 | 0.000 | 0.000 | 0.000 | 0.000 | 0.000 | 0.000 | 0.174 | 0.150 | 0.000 | 0.000 | 0.000 | 0.000 | 0.042 | 0.000 | 0.000 |
| 5-643 | 0.000 | 0.000 | 0.000 | 0.000 | 0.000 | 0.000 | 0.000 | 0.000 | 0.000 | 0.000 | 0.000 | 0.042 | 0.000 | 0.000 | 0.000 | 0.000 | 0.000 | 0.000 | 0.000 | 0.000 | 0.000 | 0.000 |
| 5-652 | 0.000 | 0.000 | 0.000 | 0.000 | 0.000 | 0.000 | 0.000 | 0.000 | 0.000 | 0.000 | 0.000 | 0.042 | 0.125 | 0.043 | 0.000 | 0.000 | 0.000 | 0.000 | 0.048 | 0.042 | 0.000 | 0.000 |
| 5-663 | 0.619 | 0.174 | 0.238 | 0.526 | 0.900 | 0.500 | 0.174 | 0.571 | 0.600 | 0.786 | 0.619 | 0.167 | 0.083 | 0.087 | 0.150 | 0.389 | 0.650 | 0.500 | 0.571 | 0.500 | 0.429 | 0.696 |
| 5-669 | 0.000 | 0.000 | 0.000 | 0.000 | 0.000 | 0.000 | 0.000 | 0.000 | 0.000 | 0.000 | 0.000 | 0.000 | 0.000 | 0.000 | 0.100 | 0.000 | 0.000 | 0.000 | 0.000 | 0.000 | 0.000 | 0.000 |
| 5-671 | 0.000 | 0.000 | 0.000 | 0.000 | 0.000 | 0.000 | 0.000 | 0.000 | 0.000 | 0.000 | 0.000 | 0.000 | 0.000 | 0.000 | 0.000 | 0.000 | 0.000 | 0.045 | 0.048 | 0.000 | 0.000 | 0.000 |
| 5-677 | 0.000 | 0.000 | 0.000 | 0.000 | 0.000 | 0.000 | 0.000 | 0.000 | 0.000 | 0.000 | 0.000 | 0.000 | 0.000 | 0.043 | 0.000 | 0.000 | 0.000 | 0.045 | 0.000 | 0.000 | 0.000 | 0.000 |
| 5-683 | 0.000 | 0.000 | 0.048 | 0.000 | 0.000 | 0.000 | 0.000 | 0.000 | 0.133 | 0.000 | 0.000 | 0.000 | 0.000 | 0.043 | 0.000 | 0.000 | 0.000 | 0.000 | 0.000 | 0.000 | 0.000 | 0.000 |
| 5-691 | 0.000 | 0.000 | 0.000 | 0.000 | 0.000 | 0.000 | 0.000 | 0.000 | 0.000 | 0.000 | 0.000 | 0.000 | 0.000 | 0.043 | 0.000 | 0.000 | 0.000 | 0.000 | 0.000 | 0.000 | 0.000 | 0.000 |
| 5-699 | 0.000 | 0.000 | 0.000 | 0.000 | 0.000 | 0.000 | 0.000 | 0.000 | 0.000 | 0.000 | 0.000 | 0.000 | 0.000 | 0.261 | 0.200 | 0.000 | 0.000 | 0.000 | 0.000 | 0.000 | 0.000 | 0.000 |
| 5-703 | 0.000 | 0.000 | 0.000 | 0.000 | 0.000 | 0.000 | 0.000 | 0.000 | 0.000 | 0.000 | 0.000 | 0.000 | 0.000 | 0.174 | 0.250 | 0.000 | 0.000 | 0.000 | 0.000 | 0.000 | 0.000 | 0.000 |
| 5-710 | 0.000 | 0.000 | 0.000 | 0.000 | 0.000 | 0.000 | 0.000 | 0.000 | 0.000 | 0.000 | 0.000 | 0.000 | 0.000 | 0.000 | 0.050 | 0.000 | 0.000 | 0.000 | 0.000 | 0.000 | 0.000 | 0.000 |
| 5-719 | 0.000 | 0.000 | 0.000 | 0.000 | 0.000 | 0.000 | 0.000 | 0.000 | 0.000 | 0.000 | 0.000 | 0.042 | 0.000 | 0.478 | 0.250 | 0.000 | 0.000 | 0.000 | 0.048 | 0.000 | 0.000 | 0.000 |
| 5-726 | 0.000 | 0.043 | 0.000 | 0.000 | 0.000 | 0.000 | 0.000 | 0.000 | 0.000 | 0.000 | 0.000 | 0.000 | 0.000 | 0.087 | 0.100 | 0.000 | 0.000 | 0.000 | 0.000 | 0.000 | 0.000 | 0.000 |
| 5-736 | 0.000 | 0.000 | 0.000 | 0.000 | 0.050 | 0.000 | 0.000 | 0.000 | 0.000 | 0.000 | 0.000 | 0.000 | 0.000 | 0.000 | 0.000 | 0.000 | 0.000 | 0.000 | 0.000 | 0.000 | 0.000 | 0.000 |
| 5-744 | 0.000 | 0.000 | 0.000 | 0.000 | 0.000 | 0.000 | 0.000 | 0.000 | 0.000 | 0.000 | 0.000 | 0.000 | 0.000 | 0.000 | 0.050 | 0.000 | 0.000 | 0.000 | 0.000 | 0.000 | 0.000 | 0.000 |
| 5-749 | 0.000 | 0.000 | 0.000 | 0.000 | 0.000 | 0.000 | 0.000 | 0.000 | 0.000 | 0.000 | 0.000 | 0.000 | 0.000 | 0.000 | 0.050 | 0.000 | 0.000 | 0.000 | 0.000 | 0.000 | 0.000 | 0.000 |
| 5-756 | 0.000 | 0.000 | 0.000 | 0.000 | 0.000 | 0.000 | 0.000 | 0.000 | 0.000 | 0.000 | 0.000 | 0.125 | 0.000 | 0.652 | 0.300 | 0.000 | 0.000 | 0.091 | 0.048 | 0.000 | 0.238 | 0.000 |
| 5-764 | 0.000 | 0.000 | 0.000 | 0.000 | 0.000 | 0.056 | 0.087 | 0.071 | 0.067 | 0.000 | 0.000 | 0.000 | 0.000 | 0.043 | 0.000 | 0.000 | 0.000 | 0.000 | 0.000 | 0.167 | 0.143 | 0.000 |
| 5-768 | 0.000 | 0.000 | 0.000 | 0.000 | 0.000 | 0.111 | 0.000 | 0.000 | 0.000 | 0.000 | 0.000 | 0.125 | 0.083 | 0.000 | 0.150 | 0.056 | 0.000 | 0.000 | 0.000 | 0.083 | 0.048 | 0.174 |
| 5-773 | 0.000 | 0.000 | 0.000 | 0.000 | 0.000 | 0.000 | 0.000 | 0.000 | 0.000 | 0.000 | 0.000 | 0.000 | 0.000 | 0.000 | 0.050 | 0.000 | 0.000 | 0.000 | 0.000 | 0.000 | 0.000 | 0.000 |
| 5-779 | 0.000 | 0.130 | 0.000 | 0.000 | 0.000 | 0.111 | 0.087 | 0.000 | 0.000 | 0.000 | 0.000 | 0.375 | 0.042 | 0.957 | 0.450 | 0.056 | 0.000 | 0.227 | 0.238 | 0.125 | 0.429 | 0.261 |
| 5-783 | 0.000 | 0.000 | 0.000 | 0.000 | 0.000 | 0.000 | 0.000 | 0.000 | 0.000 | 0.000 | 0.000 | 0.000 | 0.000 | 0.217 | 0.050 | 0.000 | 0.000 | 0.000 | 0.000 | 0.000 | 0.000 | 0.000 |
| 5-790 | 0.000 | 0.000 | 0.000 | 0.000 | 0.000 | 0.000 | 0.000 | 0.000 | 0.000 | 0.000 | 0.000 | 0.167 | 0.042 | 0.696 | 0.350 | 0.000 | 0.000 | 0.000 | 0.048 | 0.000 | 0.095 | 0.000 |
| 5-796 | 0.000 | 0.000 | 0.000 | 0.000 | 0.000 | 0.000 | 0.000 | 0.000 | 0.000 | 0.000 | 0.000 | 0.000 | 0.000 | 0.000 | 0.050 | 0.000 | 0.000 | 0.000 | 0.000 | 0.000 | 0.000 | 0.000 |
| 5-808 | 0.000 | 0.000 | 0.000 | 0.000 | 0.000 | 0.000 | 0.000 | 0.000 | 0.000 | 0.000 | 0.000 | 0.167 | 0.000 | 0.652 | 0.350 | 0.000 | 0.000 | 0.091 | 0.000 | 0.000 | 0.000 | 0.000 |
| 5-817 | 0.000 | 0.000 | 0.000 | 0.000 | 0.000 | 0.056 | 0.000 | 0.000 | 0.000 | 0.000 | 0.000 | 0.167 | 0.000 | 0.696 | 0.250 | 0.000 | 0.050 | 0.045 | 0.000 | 0.042 | 0.095 | 0.000 |
| 5-824 | 0.000 | 0.000 | 0.000 | 0.000 | 0.000 | 0.000 | 0.000 | 0.000 | 0.000 | 0.000 | 0.000 | 0.000 | 0.000 | 0.043 | 0.000 | 0.056 | 0.000 | 0.000 | 0.000 | 0.000 | 0.000 | 0.000 |
| 5-842 | 0.000 | 0.000 | 0.000 | 0.000 | 0.000 | 0.000 | 0.000 | 0.000 | 0.000 | 0.000 | 0.000 | 0.000 | 0.000 | 0.087 | 0.000 | 0.000 | 0.000 | 0.000 | 0.000 | 0.000 | 0.000 | 0.000 |
| 5-848 | 0.000 | 0.000 | 0.000 | 0.000 | 0.000 | 0.000 | 0.000 | 0.000 | 0.000 | 0.000 | 0.000 | 0.000 | 0.000 | 0.000 | 0.050 | 0.000 | 0.000 | 0.000 | 0.000 | 0.000 | 0.000 | 0.000 |
| 5-852 | 0.000 | 0.000 | 0.000 | 0.000 | 0.000 | 0.000 | 0.000 | 0.000 | 0.000 | 0.000 | 0.000 | 0.000 | 0.000 | 0.043 | 0.050 | 0.000 | 0.000 | 0.000 | 0.000 | 0.000 | 0.000 | 0.000 |
| 5-858 | 0.000 | 0.000 | 0.000 | 0.000 | 0.000 | 0.000 | 0.000 | 0.000 | 0.000 | 0.000 | 0.000 | 0.000 | 0.000 | 0.000 | 0.100 | 0.000 | 0.000 | 0.000 | 0.000 | 0.000 | 0.000 | 0.000 |
| 5-862 | 0.000 | 0.000 | 0.000 | 0.000 | 0.000 | 0.000 | 0.000 | 0.000 | 0.000 | 0.000 | 0.000 | 0.000 | 0.042 | 0.174 | 0.000 | 0.000 | 0.000 | 0.091 | 0.000 | 0.000 | 0.048 | 0.000 |
| 5-870 | 0.000 | 0.000 | 0.000 | 0.000 | 0.000 | 0.000 | 0.000 | 0.000 | 0.000 | 0.000 | 0.000 | 0.000 | 0.000 | 0.087 | 0.050 | 0.000 | 0.000 | 0.000 | 0.000 | 0.000 | 0.000 | 0.000 |
| 5-877 | 0.000 | 0.000 | 0.000 | 0.000 | 0.000 | 0.000 | 0.000 | 0.000 | 0.000 | 0.000 | 0.000 | 0.417 | 0.000 | 0.826 | 0.350 | 0.000 | 0.000 | 0.136 | 0.286 | 0.000 | 0.333 | 0.000 |
| 5-883 | 0.000 | 0.000 | 0.000 | 0.000 | 0.000 | 0.000 | 0.000 | 0.000 | 0.000 | 0.000 | 0.000 | 0.375 | 0.000 | 0.739 | 0.400 | 0.000 | 0.000 | 0.091 | 0.333 | 0.000 | 0.333 | 0.000 |
| 5-902 | 0.000 | 0.000 | 0.000 | 0.000 | 0.000 | 0.000 | 0.000 | 0.000 | 0.000 | 0.000 | 0.000 | 0.000 | 0.000 | 0.043 | 0.050 | 0.000 | 0.000 | 0.000 | 0.000 | 0.000 | 0.000 | 0.000 |
| 5-908 | 0.000 | 0.000 | 0.000 | 0.000 | 0.000 | 0.000 | 0.000 | 0.000 | 0.000 | 0.000 | 0.000 | 0.000 | 0.000 | 0.000 | 0.050 | 0.000 | 0.000 | 0.000 | 0.000 | 0.000 | 0.000 | 0.000 |
| 5-911 | 0.000 | 0.000 | 0.000 | 0.000 | 0.000 | 0.000 | 0.000 | 0.000 | 0.000 | 0.000 | 0.000 | 0.000 | 0.000 | 0.043 | 0.050 | 0.000 | 0.000 | 0.000 | 0.000 | 0.000 | 0.000 | 0.000 |
| 5-917 | 0.000 | 0.000 | 0.000 | 0.000 | 0.000 | 0.000 | 0.000 | 0.000 | 0.000 | 0.000 | 0.000 | 0.000 | 0.000 | 0.304 | 0.100 | 0.000 | 0.000 | 0.000 | 0.000 | 0.000 | 0.000 | 0.000 |
| 5-929 | 0.000 | 0.000 | 0.000 | 0.000 | 0.000 | 0.000 | 0.000 | 0.000 | 0.000 | 0.000 | 0.000 | 0.000 | 0.000 | 0.435 | 0.000 | 0.000 | 0.000 | 0.000 | 0.000 | 0.000 | 0.000 | 0.000 |
| 5-948 | 0.000 | 0.000 | 0.000 | 0.000 | 0.000 | 0.000 | 0.000 | 0.000 | 0.000 | 0.000 | 0.000 | 0.000 | 0.000 | 0.000 | 0.050 | 0.000 | 0.000 | 0.000 | 0.000 | 0.000 | 0.000 | 0.000 |
| 5-960 | 0.000 | 0.000 | 0.000 | 0.000 | 0.000 | 0.000 | 0.000 | 0.000 | 0.000 | 0.000 | 0.000 | 0.000 | 0.000 | 0.000 | 0.100 | 0.000 | 0.000 | 0.000 | 0.000 | 0.000 | 0.000 | 0.000 |
| 5-966 | 0.000 | 0.000 | 0.000 | 0.000 | 0.000 | 0.000 | 0.000 | 0.000 | 0.000 | 0.000 | 0.000 | 0.000 | 0.042 | 0.043 | 0.000 | 0.000 | 0.000 | 0.000 | 0.000 | 0.000 | 0.000 | 0.000 |
| 5-977 | 0.000 | 0.000 | 0.000 | 0.000 | 0.000 | 0.000 | 0.000 | 0.000 | 0.000 | 0.000 | 0.000 | 0.000 | 0.000 | 0.217 | 0.100 | 0.000 | 0.000 | 0.000 | 0.000 | 0.000 | 0.000 | 0.000 |
| 5-980 | 0.000 | 0.000 | 0.000 | 0.000 | 0.000 | 0.000 | 0.000 | 0.000 | 0.000 | 0.000 | 0.000 | 0.000 | 0.000 | 0.043 | 0.050 | 0.000 | 0.000 | 0.000 | 0.000 | 0.000 | 0.000 | 0.000 |
| 5-993 | 0.000 | 0.000 | 0.000 | 0.000 | 0.000 | 0.000 | 0.000 | 0.000 | 0.000 | 0.000 | 0.000 | 0.000 | 0.000 | 0.043 | 0.000 | 0.000 | 0.000 | 0.000 | 0.000 | 0.000 | 0.000 | 0.000 |
| 5-999 | 0.000 | 0.000 | 0.000 | 0.000 | 0.000 | 0.000 | 0.000 | 0.000 | 0.000 | 0.000 | 0.000 | 0.042 | 0.000 | 0.174 | 0.000 | 0.000 | 0.000 | 0.000 | 0.000 | 0.000 | 0.000 | 0.000 |
| 5-1014 | 0.000 | 0.000 | 0.000 | 0.000 | 0.000 | 0.000 | 0.000 | 0.000 | 0.000 | 0.000 | 0.000 | 0.000 | 0.000 | 0.043 | 0.000 | 0.000 | 0.000 | 0.000 | 0.000 | 0.000 | 0.000 | 0.000 |
| 5-1054 | 0.000 | 0.000 | 0.000 | 0.000 | 0.000 | 0.000 | 0.000 | 0.000 | 0.000 | 0.000 | 0.000 | 0.000 | 0.000 | 0.043 | 0.050 | 0.000 | 0.000 | 0.000 | 0.000 | 0.000 | 0.000 | 0.000 |
| 5-1061 | 0.000 | 0.000 | 0.000 | 0.000 | 0.000 | 0.000 | 0.000 | 0.000 | 0.000 | 0.000 | 0.000 | 0.000 | 0.000 | 0.043 | 0.000 | 0.000 | 0.000 | 0.000 | 0.000 | 0.000 | 0.000 | 0.000 |
| 5-1072 | 0.000 | 0.000 | 0.000 | 0.000 | 0.000 | 0.000 | 0.000 | 0.000 | 0.000 | 0.000 | 0.000 | 0.000 | 0.000 | 0.043 | 0.050 | 0.000 | 0.000 | 0.000 | 0.000 | 0.000 | 0.000 | 0.000 |
| 5-1079 | 0.000 | 0.000 | 0.000 | 0.000 | 0.000 | 0.000 | 0.000 | 0.000 | 0.000 | 0.000 | 0.000 | 0.000 | 0.000 | 0.043 | 0.000 | 0.000 | 0.000 | 0.000 | 0.000 | 0.000 | 0.000 | 0.000 |
| 5-1095 | 0.000 | 0.000 | 0.000 | 0.000 | 0.000 | 0.000 | 0.000 | 0.000 | 0.000 | 0.000 | 0.000 | 0.000 | 0.000 | 0.043 | 0.000 | 0.000 | 0.000 | 0.000 | 0.000 | 0.000 | 0.000 | 0.000 |
| 5-1121 | 0.000 | 0.000 | 0.000 | 0.000 | 0.000 | 0.000 | 0.000 | 0.000 | 0.000 | 0.000 | 0.000 | 0.000 | 0.000 | 0.043 | 0.000 | 0.000 | 0.000 | 0.000 | 0.000 | 0.000 | 0.000 | 0.000 |
| 5-1151 | 0.000 | 0.000 | 0.000 | 0.000 | 0.000 | 0.000 | 0.000 | 0.000 | 0.000 | 0.000 | 0.000 | 0.000 | 0.000 | 0.000 | 0.050 | 0.000 | 0.000 | 0.000 | 0.000 | 0.000 | 0.000 | 0.000 |
| 5-1185 | 0.000 | 0.000 | 0.000 | 0.000 | 0.050 | 0.000 | 0.000 | 0.000 | 0.000 | 0.000 | 0.000 | 0.000 | 0.000 | 0.000 | 0.000 | 0.000 | 0.000 | 0.000 | 0.000 | 0.000 | 0.000 | 0.000 |
| 5-1197 | 0.000 | 0.000 | 0.000 | 0.000 | 0.050 | 0.000 | 0.000 | 0.000 | 0.000 | 0.000 | 0.000 | 0.000 | 0.000 | 0.000 | 0.000 | 0.000 | 0.000 | 0.000 | 0.000 | 0.000 | 0.000 | 0.000 |
| 9-101 | 0.000 | 0.042 | 0.000 | 0.000 | 0.000 | 0.000 | 0.000 | 0.000 | 0.125 | 0.000 | 0.000 | 0.000 | 0.000 | 0.000 | 0.000 | 0.000 | 0.000 | 0.000 | 0.000 | 0.000 | 0.000 | 0.000 |
| 9-112 | 0.000 | 0.000 | 0.000 | 0.000 | 0.000 | 0.077 | 0.000 | 0.000 | 0.000 | 0.000 | 0.000 | 0.000 | 0.000 | 0.000 | 0.000 | 0.048 | 0.000 | 0.000 | 0.000 | 0.000 | 0.000 | 0.000 |
| 9-128 | 0.000 | 0.042 | 0.000 | 0.000 | 0.000 | 0.077 | 0.000 | 0.000 | 0.000 | 0.000 | 0.000 | 0.000 | 0.000 | 0.000 | 0.000 | 0.000 | 0.000 | 0.000 | 0.000 | 0.000 | 0.000 | 0.000 |
| 9-144 | 0.000 | 0.000 | 0.000 | 0.000 | 0.000 | 0.077 | 0.000 | 0.000 | 0.000 | 0.000 | 0.000 | 0.000 | 0.000 | 0.000 | 0.000 | 0.000 | 0.000 | 0.000 | 0.000 | 0.000 | 0.000 | 0.000 |
| 9-156 | 0.000 | 0.000 | 0.000 | 0.000 | 0.000 | 0.000 | 0.000 | 0.000 | 0.000 | 0.000 | 0.000 | 0.000 | 0.000 | 0.000 | 0.050 | 0.000 | 0.000 | 0.000 | 0.000 | 0.000 | 0.000 | 0.000 |
| 9-163 | 0.000 | 0.000 | 0.143 | 0.000 | 0.000 | 0.000 | 0.000 | 0.000 | 0.000 | 0.000 | 0.000 | 0.000 | 0.000 | 0.045 | 0.000 | 0.000 | 0.000 | 0.000 | 0.000 | 0.083 | 0.000 | 0.000 |
| 9-178 | 0.000 | 0.000 | 0.000 | 0.000 | 0.048 | 0.077 | 0.000 | 0.000 | 0.000 | 0.083 | 0.000 | 0.000 | 0.000 | 0.000 | 0.050 | 0.000 | 0.000 | 0.042 | 0.000 | 0.000 | 0.000 | 0.000 |
| 9-194 | 0.000 | 0.000 | 0.000 | 0.000 | 0.000 | 0.000 | 0.000 | 0.000 | 0.000 | 0.000 | 0.111 | 0.000 | 0.000 | 0.000 | 0.000 | 0.000 | 0.000 | 0.000 | 0.000 | 0.000 | 0.000 | 0.000 |
| 9-198 | 0.000 | 0.083 | 0.000 | 0.000 | 0.000 | 0.000 | 0.000 | 0.000 | 0.000 | 0.000 | 0.056 | 0.000 | 0.000 | 0.045 | 0.100 | 0.000 | 0.000 | 0.000 | 0.000 | 0.000 | 0.000 | 0.043 |
| 9-230 | 0.000 | 0.083 | 0.000 | 0.000 | 0.000 | 0.077 | 0.000 | 0.000 | 0.000 | 0.083 | 0.000 | 0.000 | 0.043 | 0.000 | 0.050 | 0.000 | 0.000 | 0.000 | 0.000 | 0.083 | 0.000 | 0.000 |
| 9-238 | 0.000 | 0.042 | 0.000 | 0.000 | 0.000 | 0.000 | 0.000 | 0.000 | 0.000 | 0.000 | 0.056 | 0.048 | 0.000 | 0.000 | 0.000 | 0.000 | 0.000 | 0.000 | 0.000 | 0.000 | 0.136 | 0.000 |
| 9-242 | 0.000 | 0.333 | 0.000 | 0.056 | 0.048 | 0.077 | 0.000 | 0.000 | 0.000 | 0.250 | 0.000 | 0.048 | 0.000 | 0.000 | 0.150 | 0.048 | 0.000 | 0.000 | 0.000 | 0.625 | 0.318 | 0.043 |
| 9-250 | 0.000 | 0.917 | 0.429 | 0.167 | 0.000 | 0.077 | 0.273 | 0.000 | 0.000 | 0.500 | 0.778 | 0.048 | 0.000 | 0.091 | 0.250 | 0.000 | 0.000 | 0.583 | 0.250 | 0.833 | 0.636 | 0.000 |
| 9-256 | 0.000 | 0.083 | 0.000 | 0.000 | 0.048 | 0.077 | 0.000 | 0.000 | 0.000 | 0.000 | 0.056 | 0.000 | 0.043 | 0.000 | 0.100 | 0.000 | 0.042 | 0.000 | 0.000 | 0.000 | 0.000 | 0.043 |
| 9-266 | 1.000 | 1.000 | 0.643 | 0.889 | 1.000 | 0.615 | 1.000 | 0.444 | 0.375 | 0.500 | 0.056 | 0.381 | 0.957 | 0.818 | 0.750 | 1.000 | 1.000 | 0.917 | 0.708 | 0.958 | 1.000 | 1.000 |
| 9-275 | 0.048 | 0.000 | 0.000 | 0.000 | 0.000 | 0.000 | 0.000 | 0.000 | 0.000 | 0.000 | 0.056 | 0.095 | 0.043 | 0.455 | 0.200 | 0.190 | 0.042 | 0.000 | 0.000 | 0.000 | 0.045 | 0.174 |
| 9-283 | 0.000 | 0.042 | 0.000 | 0.000 | 0.000 | 0.000 | 0.000 | 0.000 | 0.250 | 0.000 | 0.000 | 0.048 | 0.000 | 0.000 | 0.000 | 0.000 | 0.000 | 0.000 | 0.000 | 0.000 | 0.000 | 0.000 |
| 9-285 | 0.000 | 0.042 | 0.071 | 0.000 | 0.000 | 0.000 | 0.000 | 0.000 | 0.250 | 0.083 | 0.000 | 0.000 | 0.000 | 0.000 | 0.000 | 0.048 | 0.000 | 0.000 | 0.000 | 0.000 | 0.000 | 0.000 |
| 9-290 | 0.048 | 0.292 | 0.000 | 0.056 | 0.381 | 0.077 | 0.045 | 0.000 | 0.125 | 0.083 | 0.000 | 0.000 | 0.000 | 0.000 | 0.100 | 0.000 | 0.000 | 0.000 | 0.042 | 0.333 | 0.091 | 0.130 |
| 9-299 | 0.000 | 0.000 | 0.000 | 0.000 | 0.000 | 0.000 | 0.000 | 0.000 | 0.000 | 0.000 | 0.000 | 0.000 | 0.000 | 0.045 | 0.000 | 0.000 | 0.000 | 0.000 | 0.000 | 0.000 | 0.000 | 0.000 |
| 9-305 | 0.000 | 0.000 | 0.000 | 0.000 | 0.000 | 0.000 | 0.000 | 0.000 | 0.000 | 0.000 | 0.000 | 0.000 | 0.000 | 0.000 | 0.150 | 0.000 | 0.000 | 0.000 | 0.000 | 0.000 | 0.091 | 0.000 |
| 9-314 | 0.095 | 0.375 | 0.000 | 0.111 | 0.048 | 0.077 | 0.136 | 0.111 | 0.000 | 0.000 | 0.000 | 0.000 | 0.000 | 0.000 | 0.100 | 0.524 | 0.042 | 0.042 | 0.000 | 0.208 | 0.045 | 0.087 |
| 9-319 | 0.000 | 0.333 | 0.071 | 0.056 | 0.095 | 0.000 | 0.091 | 0.111 | 0.000 | 0.000 | 0.000 | 0.000 | 0.043 | 0.136 | 0.100 | 0.619 | 0.042 | 0.042 | 0.000 | 0.083 | 0.000 | 0.000 |
| 9-327 | 0.000 | 0.000 | 0.000 | 0.000 | 0.000 | 0.000 | 0.000 | 0.000 | 0.000 | 0.000 | 0.000 | 0.048 | 0.000 | 0.045 | 0.000 | 0.000 | 0.000 | 0.000 | 0.000 | 0.000 | 0.000 | 0.000 |
| 9-338 | 0.000 | 0.000 | 0.000 | 0.000 | 0.000 | 0.000 | 0.000 | 0.000 | 0.000 | 0.083 | 0.000 | 0.000 | 0.174 | 0.000 | 0.050 | 0.048 | 0.083 | 0.000 | 0.000 | 0.000 | 0.136 | 0.043 |
| 9-346 | 0.000 | 0.000 | 0.000 | 0.000 | 0.000 | 0.000 | 0.000 | 0.000 | 0.000 | 0.000 | 0.000 | 0.000 | 0.000 | 0.000 | 0.100 | 0.000 | 0.000 | 0.000 | 0.000 | 0.000 | 0.136 | 0.000 |
| 9-350 | 0.048 | 0.083 | 0.000 | 0.000 | 0.000 | 0.000 | 0.045 | 0.000 | 0.000 | 0.083 | 0.056 | 0.000 | 0.000 | 0.000 | 0.100 | 0.048 | 0.000 | 0.000 | 0.000 | 0.583 | 0.182 | 0.000 |
| 9-356 | 0.000 | 0.000 | 0.000 | 0.000 | 0.000 | 0.000 | 0.000 | 0.000 | 0.000 | 0.000 | 0.000 | 0.048 | 0.000 | 0.045 | 0.150 | 0.000 | 0.000 | 0.125 | 0.083 | 0.000 | 0.045 | 0.000 |
| 9-363 | 0.000 | 0.042 | 0.000 | 0.000 | 0.000 | 0.000 | 0.000 | 0.000 | 0.125 | 0.083 | 0.000 | 0.000 | 0.000 | 0.045 | 0.100 | 0.000 | 0.000 | 0.083 | 0.000 | 0.042 | 0.091 | 0.000 |
| 9-366 | 0.000 | 0.000 | 0.000 | 0.000 | 0.000 | 0.000 | 0.000 | 0.000 | 0.125 | 0.000 | 0.000 | 0.000 | 0.000 | 0.045 | 0.150 | 0.000 | 0.000 | 0.000 | 0.000 | 0.000 | 0.136 | 0.043 |
| 9-375 | 0.048 | 0.125 | 0.000 | 0.000 | 0.000 | 0.077 | 0.000 | 0.000 | 0.000 | 0.083 | 0.000 | 0.000 | 0.043 | 0.045 | 0.000 | 0.048 | 0.000 | 0.000 | 0.000 | 0.000 | 0.091 | 0.000 |
| 9-382 | 0.000 | 0.125 | 0.000 | 0.000 | 0.095 | 0.000 | 0.000 | 0.000 | 0.000 | 0.000 | 0.056 | 0.000 | 0.043 | 0.091 | 0.250 | 0.000 | 0.000 | 0.167 | 0.125 | 0.042 | 0.136 | 0.000 |
| 9-398 | 0.000 | 0.042 | 0.000 | 0.000 | 0.048 | 0.231 | 0.045 | 0.000 | 0.000 | 0.333 | 0.000 | 0.429 | 0.217 | 0.318 | 0.350 | 0.000 | 0.083 | 0.417 | 0.292 | 0.083 | 0.545 | 0.087 |
| 9-411 | 0.048 | 0.458 | 0.000 | 0.056 | 0.048 | 0.077 | 0.182 | 0.000 | 0.000 | 0.083 | 0.167 | 0.048 | 0.000 | 0.045 | 0.050 | 0.048 | 0.000 | 0.000 | 0.042 | 0.583 | 0.045 | 0.000 |
| 9-420 | 0.000 | 0.458 | 0.000 | 0.000 | 0.048 | 0.000 | 0.091 | 0.000 | 0.000 | 0.083 | 0.111 | 0.000 | 0.130 | 0.091 | 0.100 | 0.048 | 0.000 | 0.042 | 0.000 | 0.417 | 0.182 | 0.043 |
| 9-423 | 0.000 | 0.042 | 0.143 | 0.000 | 0.048 | 0.154 | 0.000 | 0.000 | 0.000 | 0.083 | 0.056 | 0.000 | 0.000 | 0.045 | 0.200 | 0.000 | 0.000 | 0.125 | 0.000 | 0.250 | 0.273 | 0.000 |
| 9-435 | 0.000 | 0.000 | 0.000 | 0.000 | 0.000 | 0.000 | 0.000 | 0.000 | 0.000 | 0.000 | 0.000 | 0.143 | 0.087 | 0.318 | 0.550 | 0.048 | 0.000 | 0.000 | 0.250 | 0.000 | 0.091 | 0.000 |
| 9-448 | 0.190 | 0.000 | 0.000 | 0.000 | 0.095 | 0.308 | 0.000 | 0.000 | 0.250 | 0.333 | 0.000 | 0.000 | 0.261 | 0.045 | 0.050 | 0.048 | 0.083 | 0.000 | 0.083 | 0.208 | 0.455 | 0.000 |
| 9-451 | 0.095 | 0.167 | 0.000 | 0.000 | 0.095 | 0.154 | 0.000 | 0.333 | 0.000 | 0.000 | 0.000 | 0.048 | 0.304 | 0.182 | 0.250 | 0.095 | 0.292 | 0.250 | 0.250 | 0.375 | 0.818 | 0.043 |
| 9-457 | 0.048 | 0.000 | 0.000 | 0.000 | 0.048 | 0.000 | 0.000 | 0.111 | 0.250 | 0.000 | 0.000 | 0.000 | 0.217 | 0.045 | 0.050 | 0.095 | 0.125 | 0.000 | 0.000 | 0.042 | 0.182 | 0.043 |
| 9-466 | 0.000 | 0.042 | 0.000 | 0.000 | 0.000 | 0.000 | 0.000 | 0.000 | 0.125 | 0.167 | 0.000 | 0.143 | 0.000 | 0.045 | 0.200 | 0.000 | 0.000 | 0.250 | 0.417 | 0.083 | 0.455 | 0.000 |
| 9-476 | 0.000 | 0.042 | 0.000 | 0.000 | 0.048 | 0.308 | 0.000 | 0.000 | 0.125 | 0.167 | 0.000 | 0.286 | 0.174 | 0.318 | 0.200 | 0.000 | 0.042 | 0.250 | 0.458 | 0.333 | 0.773 | 0.000 |
| 9-482 | 0.000 | 0.125 | 0.000 | 0.000 | 0.000 | 0.231 | 0.000 | 0.000 | 0.000 | 0.417 | 0.000 | 0.238 | 0.130 | 0.136 | 0.050 | 0.000 | 0.083 | 0.083 | 0.167 | 0.000 | 0.318 | 0.000 |
| 9-487 | 0.048 | 0.125 | 0.000 | 0.056 | 0.048 | 0.308 | 0.000 | 0.000 | 0.000 | 0.250 | 0.000 | 0.143 | 0.043 | 0.000 | 0.050 | 0.048 | 0.083 | 0.500 | 0.458 | 0.167 | 0.727 | 0.000 |
| 9-498 | 0.095 | 0.042 | 0.000 | 0.000 | 0.238 | 0.000 | 0.000 | 0.222 | 0.000 | 0.000 | 0.000 | 0.476 | 0.261 | 0.455 | 0.200 | 0.000 | 0.000 | 0.292 | 0.292 | 0.000 | 0.455 | 0.000 |
| 9-501 | 0.000 | 0.083 | 0.000 | 0.000 | 0.048 | 0.000 | 0.000 | 0.111 | 0.000 | 0.083 | 0.056 | 0.429 | 0.087 | 0.409 | 0.350 | 0.000 | 0.042 | 0.458 | 0.542 | 0.042 | 0.500 | 0.000 |
| 9-506 | 0.000 | 0.000 | 0.000 | 0.000 | 0.048 | 0.000 | 0.000 | 0.000 | 0.000 | 0.083 | 0.000 | 0.095 | 0.304 | 0.000 | 0.000 | 0.000 | 0.000 | 0.167 | 0.125 | 0.042 | 0.409 | 0.000 |
| 9-517 | 0.000 | 0.000 | 0.000 | 0.000 | 0.000 | 0.000 | 0.000 | 0.000 | 0.000 | 0.000 | 0.000 | 0.048 | 0.000 | 0.045 | 0.000 | 0.000 | 0.000 | 0.000 | 0.000 | 0.000 | 0.000 | 0.000 |
| 9-527 | 0.000 | 0.042 | 0.071 | 0.000 | 0.048 | 0.000 | 0.000 | 0.111 | 0.000 | 0.083 | 0.000 | 0.143 | 0.000 | 0.227 | 0.350 | 0.000 | 0.042 | 0.208 | 0.208 | 0.042 | 0.636 | 0.000 |
| 9-534 | 0.000 | 0.000 | 0.000 | 0.000 | 0.000 | 0.077 | 0.000 | 0.000 | 0.000 | 0.167 | 0.000 | 0.381 | 0.087 | 0.318 | 0.450 | 0.000 | 0.000 | 0.583 | 0.667 | 0.042 | 0.682 | 0.000 |
| 9-537 | 0.000 | 0.000 | 0.000 | 0.000 | 0.000 | 0.077 | 0.000 | 0.000 | 0.000 | 0.083 | 0.000 | 0.000 | 0.000 | 0.045 | 0.100 | 0.000 | 0.000 | 0.000 | 0.083 | 0.042 | 0.045 | 0.000 |
| 9-563 | 0.000 | 0.000 | 0.000 | 0.000 | 0.048 | 0.000 | 0.000 | 0.000 | 0.000 | 0.000 | 0.000 | 0.000 | 0.000 | 0.045 | 0.050 | 0.000 | 0.000 | 0.125 | 0.000 | 0.042 | 0.000 | 0.043 |
| 9-572 | 0.000 | 0.000 | 0.000 | 0.000 | 0.000 | 0.077 | 0.000 | 0.000 | 0.000 | 0.083 | 0.000 | 0.000 | 0.000 | 0.000 | 0.000 | 0.000 | 0.000 | 0.000 | 0.000 | 0.000 | 0.000 | 0.000 |
| 9-579 | 0.000 | 0.000 | 0.000 | 0.000 | 0.000 | 0.000 | 0.000 | 0.000 | 0.000 | 0.083 | 0.000 | 0.000 | 0.000 | 0.000 | 0.000 | 0.000 | 0.000 | 0.000 | 0.000 | 0.000 | 0.000 | 0.000 |
| 9-592 | 0.000 | 0.375 | 0.000 | 0.000 | 0.095 | 0.231 | 0.227 | 0.000 | 0.000 | 0.167 | 0.056 | 0.000 | 0.043 | 0.091 | 0.150 | 0.000 | 0.042 | 0.417 | 0.250 | 0.333 | 0.318 | 0.043 |
| 9-600 | 0.476 | 0.208 | 0.000 | 0.000 | 0.238 | 0.308 | 0.000 | 0.000 | 0.000 | 0.250 | 0.056 | 0.333 | 0.261 | 0.364 | 0.200 | 0.143 | 0.292 | 0.708 | 0.708 | 0.333 | 0.864 | 0.130 |
| 9-606 | 0.381 | 0.125 | 0.000 | 0.000 | 0.238 | 0.154 | 0.000 | 0.000 | 0.000 | 0.333 | 0.056 | 0.286 | 0.261 | 0.318 | 0.200 | 0.095 | 0.333 | 0.750 | 0.667 | 0.250 | 0.727 | 0.087 |
| 9-622 | 0.000 | 0.000 | 0.000 | 0.000 | 0.000 | 0.000 | 0.000 | 0.000 | 0.125 | 0.000 | 0.000 | 0.000 | 0.000 | 0.000 | 0.000 | 0.000 | 0.000 | 0.000 | 0.000 | 0.000 | 0.045 | 0.000 |
| 9-633 | 0.048 | 0.042 | 0.000 | 0.000 | 0.000 | 0.077 | 0.091 | 0.000 | 0.000 | 0.000 | 0.000 | 0.000 | 0.087 | 0.000 | 0.100 | 0.000 | 0.083 | 0.083 | 0.000 | 0.000 | 0.227 | 0.000 |
| 9-640 | 0.000 | 0.000 | 0.000 | 0.000 | 0.000 | 0.000 | 0.000 | 0.000 | 0.000 | 0.000 | 0.000 | 0.000 | 0.043 | 0.045 | 0.050 | 0.000 | 0.042 | 0.042 | 0.000 | 0.000 | 0.000 | 0.000 |
| 9-665 | 0.000 | 0.000 | 0.000 | 0.000 | 0.000 | 0.000 | 0.000 | 0.000 | 0.000 | 0.083 | 0.000 | 0.000 | 0.000 | 0.000 | 0.000 | 0.000 | 0.000 | 0.000 | 0.000 | 0.042 | 0.045 | 0.000 |
| 9-675 | 0.143 | 0.000 | 0.000 | 0.000 | 0.000 | 0.000 | 0.000 | 0.444 | 0.125 | 0.000 | 0.000 | 0.000 | 0.348 | 0.045 | 0.050 | 0.000 | 0.083 | 0.125 | 0.375 | 0.000 | 0.182 | 0.217 |
| 9-680 | 0.095 | 0.000 | 0.000 | 0.000 | 0.000 | 0.000 | 0.000 | 0.333 | 0.125 | 0.000 | 0.000 | 0.000 | 0.348 | 0.000 | 0.000 | 0.000 | 0.000 | 0.125 | 0.042 | 0.000 | 0.227 | 0.000 |
| 9-693 | 0.000 | 0.000 | 0.000 | 0.000 | 0.000 | 0.000 | 0.000 | 0.000 | 0.125 | 0.000 | 0.000 | 0.000 | 0.000 | 0.000 | 0.000 | 0.000 | 0.000 | 0.000 | 0.000 | 0.000 | 0.000 | 0.000 |
| 9-710 | 0.000 | 0.000 | 0.000 | 0.000 | 0.000 | 0.077 | 0.000 | 0.000 | 0.000 | 0.000 | 0.000 | 0.000 | 0.000 | 0.000 | 0.000 | 0.000 | 0.000 | 0.000 | 0.000 | 0.000 | 0.000 | 0.000 |
| 9-721 | 0.000 | 0.000 | 0.000 | 0.000 | 0.048 | 0.000 | 0.000 | 0.000 | 0.000 | 0.000 | 0.000 | 0.000 | 0.000 | 0.000 | 0.000 | 0.000 | 0.000 | 0.000 | 0.000 | 0.000 | 0.000 | 0.000 |
| 9-749 | 0.000 | 0.000 | 0.000 | 0.000 | 0.000 | 0.000 | 0.000 | 0.000 | 0.000 | 0.083 | 0.000 | 0.000 | 0.000 | 0.000 | 0.000 | 0.000 | 0.000 | 0.000 | 0.000 | 0.000 | 0.000 | 0.000 |
| 9-763 | 0.000 | 0.000 | 0.000 | 0.000 | 0.000 | 0.000 | 0.000 | 0.000 | 0.000 | 0.167 | 0.000 | 0.000 | 0.000 | 0.000 | 0.050 | 0.000 | 0.000 | 0.000 | 0.000 | 0.000 | 0.045 | 0.000 |
| 9-785 | 0.000 | 0.000 | 0.000 | 0.000 | 0.000 | 0.000 | 0.000 | 0.000 | 0.000 | 0.000 | 0.000 | 0.000 | 0.043 | 0.000 | 0.000 | 0.000 | 0.000 | 0.000 | 0.000 | 0.000 | 0.091 | 0.000 |
| 9-799 | 0.000 | 0.000 | 0.071 | 0.000 | 0.000 | 0.077 | 0.000 | 0.000 | 0.000 | 0.000 | 0.000 | 0.000 | 0.000 | 0.000 | 0.000 | 0.000 | 0.000 | 0.000 | 0.000 | 0.125 | 0.000 | 0.000 |
| 9-811 | 0.000 | 0.000 | 0.000 | 0.000 | 0.000 | 0.000 | 0.000 | 0.000 | 0.000 | 0.000 | 0.000 | 0.000 | 0.000 | 0.000 | 0.000 | 0.000 | 0.000 | 0.042 | 0.000 | 0.000 | 0.000 | 0.000 |
| 9-905 | 0.000 | 0.000 | 0.000 | 0.000 | 0.000 | 0.000 | 0.000 | 0.000 | 0.000 | 0.000 | 0.000 | 0.000 | 0.000 | 0.000 | 0.000 | 0.000 | 0.000 | 0.000 | 0.000 | 0.000 | 0.091 | 0.000 |
| 9-1169 | 0.000 | 0.000 | 0.000 | 0.000 | 0.000 | 0.000 | 0.000 | 0.000 | 0.000 | 0.000 | 0.000 | 0.000 | 0.043 | 0.000 | 0.000 | 0.000 | 0.000 | 0.000 | 0.000 | 0.000 | 0.000 | 0.000 |
| 9-1174 | 0.000 | 0.000 | 0.000 | 0.000 | 0.000 | 0.000 | 0.000 | 0.000 | 0.000 | 0.000 | 0.000 | 0.000 | 0.043 | 0.000 | 0.000 | 0.000 | 0.000 | 0.000 | 0.000 | 0.000 | 0.000 | 0.000 |
| 9-1198 | 0.048 | 0.000 | 0.000 | 0.000 | 0.000 | 0.000 | 0.000 | 0.000 | 0.000 | 0.000 | 0.000 | 0.000 | 0.000 | 0.000 | 0.050 | 0.000 | 0.000 | 0.000 | 0.000 | 0.000 | 0.000 | 0.000 |
| 16-100 | 0.045 | 0.050 | 0.000 | 0.000 | 0.000 | 0.000 | 0.000 | 0.000 | 0.000 | 0.000 | 0.000 | 0.000 | 0.042 | 0.000 | 0.000 | 0.000 | 0.000 | 0.000 | 0.000 | 0.000 | 0.000 | 0.000 |
| 16-103 | 0.045 | 0.050 | 0.000 | 0.083 | 0.000 | 0.000 | 0.000 | 0.000 | 0.000 | 0.000 | 0.043 | 0.000 | 0.000 | 0.000 | 0.000 | 0.000 | 0.042 | 0.000 | 0.000 | 0.000 | 0.000 | 0.000 |
| 16-110 | 0.227 | 0.200 | 0.045 | 0.333 | 0.188 | 0.227 | 0.545 | 0.095 | 0.083 | 0.190 | 0.043 | 0.045 | 0.042 | 0.125 | 0.050 | 0.000 | 0.250 | 0.000 | 0.000 | 0.043 | 0.045 | 0.000 |
| 16-117 | 0.000 | 0.000 | 0.000 | 0.083 | 0.000 | 0.045 | 0.000 | 0.000 | 0.083 | 0.000 | 0.000 | 0.000 | 0.000 | 0.000 | 0.050 | 0.000 | 0.000 | 0.000 | 0.000 | 0.000 | 0.000 | 0.000 |
| 16-121 | 0.000 | 0.000 | 0.000 | 0.042 | 0.063 | 0.000 | 0.045 | 0.000 | 0.000 | 0.000 | 0.043 | 0.000 | 0.000 | 0.000 | 0.000 | 0.000 | 0.042 | 0.000 | 0.000 | 0.000 | 0.000 | 0.000 |
| 16-131 | 0.636 | 0.350 | 0.000 | 0.917 | 0.375 | 0.545 | 0.773 | 0.524 | 0.000 | 0.000 | 0.043 | 0.273 | 0.708 | 0.292 | 0.500 | 0.000 | 0.958 | 0.417 | 0.087 | 0.043 | 0.864 | 0.000 |
| 16-135 | 0.000 | 0.000 | 0.045 | 0.042 | 0.063 | 0.045 | 0.000 | 0.048 | 0.000 | 0.000 | 0.000 | 0.045 | 0.125 | 0.000 | 0.000 | 0.000 | 0.000 | 0.000 | 0.043 | 0.043 | 0.000 | 0.000 |
| 16-144 | 0.000 | 0.000 | 0.000 | 0.250 | 0.000 | 0.091 | 0.000 | 0.000 | 0.125 | 0.095 | 0.000 | 0.000 | 0.000 | 0.000 | 0.050 | 0.000 | 0.000 | 0.000 | 0.000 | 0.000 | 0.000 | 0.000 |
| 16-148 | 0.000 | 0.050 | 0.000 | 0.042 | 0.000 | 0.000 | 0.000 | 0.000 | 0.000 | 0.048 | 0.000 | 0.000 | 0.000 | 0.000 | 0.000 | 0.000 | 0.000 | 0.000 | 0.000 | 0.000 | 0.000 | 0.000 |
| 16-155 | 0.000 | 0.050 | 0.000 | 0.083 | 0.000 | 0.000 | 0.000 | 0.000 | 0.042 | 0.000 | 0.000 | 0.000 | 0.000 | 0.000 | 0.000 | 0.000 | 0.000 | 0.000 | 0.000 | 0.000 | 0.000 | 0.000 |
| 16-160 | 0.000 | 0.050 | 0.000 | 0.000 | 0.000 | 0.000 | 0.045 | 0.000 | 0.042 | 0.048 | 0.000 | 0.000 | 0.000 | 0.000 | 0.000 | 0.000 | 0.000 | 0.000 | 0.000 | 0.000 | 0.000 | 0.000 |
| 16-168 | 0.000 | 0.050 | 0.000 | 0.042 | 0.000 | 0.000 | 0.000 | 0.000 | 0.042 | 0.000 | 0.000 | 0.000 | 0.083 | 0.000 | 0.000 | 0.048 | 0.000 | 0.000 | 0.000 | 0.000 | 0.000 | 0.000 |
| 16-171 | 0.000 | 0.000 | 0.000 | 0.000 | 0.000 | 0.000 | 0.045 | 0.000 | 0.042 | 0.000 | 0.043 | 0.045 | 0.208 | 0.000 | 0.000 | 0.000 | 0.000 | 0.000 | 0.000 | 0.000 | 0.000 | 0.000 |
| 16-180 | 0.000 | 0.000 | 0.000 | 0.000 | 0.000 | 0.000 | 0.000 | 0.000 | 0.083 | 0.095 | 0.000 | 0.000 | 0.000 | 0.000 | 0.000 | 0.000 | 0.000 | 0.000 | 0.000 | 0.000 | 0.000 | 0.000 |
| 16-185 | 0.091 | 0.000 | 0.000 | 0.000 | 0.000 | 0.091 | 0.000 | 0.000 | 0.000 | 0.048 | 0.130 | 0.000 | 0.000 | 0.000 | 0.000 | 0.048 | 0.083 | 0.000 | 0.043 | 0.000 | 0.000 | 0.087 |
| 16-190 | 0.000 | 0.050 | 0.000 | 0.167 | 0.000 | 0.000 | 0.045 | 0.000 | 0.083 | 0.095 | 0.000 | 0.000 | 0.042 | 0.042 | 0.000 | 0.000 | 0.000 | 0.000 | 0.000 | 0.000 | 0.000 | 0.000 |
| 16-196 | 0.000 | 0.000 | 0.000 | 0.708 | 0.188 | 0.500 | 0.318 | 0.333 | 0.208 | 0.333 | 0.130 | 0.045 | 0.042 | 0.000 | 0.100 | 0.000 | 0.000 | 0.000 | 0.043 | 0.000 | 0.000 | 0.130 |
| 16-202 | 0.000 | 0.000 | 0.000 | 0.000 | 0.000 | 0.045 | 0.045 | 0.048 | 0.000 | 0.095 | 0.000 | 0.000 | 0.083 | 0.000 | 0.000 | 0.048 | 0.000 | 0.000 | 0.000 | 0.000 | 0.000 | 0.000 |
| 16-211 | 0.000 | 0.000 | 0.000 | 0.000 | 0.000 | 0.000 | 0.000 | 0.000 | 0.000 | 0.000 | 0.000 | 0.000 | 0.125 | 0.000 | 0.000 | 0.000 | 0.042 | 0.000 | 0.000 | 0.000 | 0.000 | 0.000 |
| 16-216 | 0.000 | 0.000 | 0.000 | 0.000 | 0.000 | 0.045 | 0.000 | 0.000 | 0.042 | 0.095 | 0.043 | 0.000 | 0.000 | 0.000 | 0.000 | 0.000 | 0.000 | 0.000 | 0.000 | 0.000 | 0.000 | 0.043 |
| 16-220 | 0.000 | 0.000 | 0.045 | 0.042 | 0.000 | 0.000 | 0.000 | 0.000 | 0.125 | 0.333 | 0.087 | 0.000 | 0.000 | 0.000 | 0.000 | 0.000 | 0.000 | 0.000 | 0.000 | 0.000 | 0.000 | 0.000 |
| 16-226 | 0.591 | 0.300 | 0.409 | 0.625 | 0.500 | 0.545 | 0.545 | 0.238 | 0.792 | 0.524 | 0.174 | 0.045 | 0.292 | 0.417 | 0.550 | 0.619 | 0.792 | 0.083 | 0.087 | 0.391 | 0.455 | 0.609 |
| 16-231 | 0.136 | 0.300 | 0.273 | 0.167 | 0.000 | 0.273 | 0.182 | 0.190 | 0.375 | 0.476 | 0.261 | 0.455 | 0.542 | 0.333 | 0.150 | 0.381 | 0.167 | 0.125 | 0.130 | 0.130 | 0.227 | 0.087 |
| 16-235 | 0.000 | 0.000 | 0.000 | 0.042 | 0.000 | 0.091 | 0.000 | 0.000 | 0.042 | 0.095 | 0.000 | 0.000 | 0.083 | 0.000 | 0.000 | 0.000 | 0.000 | 0.000 | 0.000 | 0.000 | 0.000 | 0.000 |
| 16-242 | 0.000 | 0.000 | 0.000 | 0.000 | 0.000 | 0.000 | 0.000 | 0.000 | 0.000 | 0.095 | 0.000 | 0.000 | 0.000 | 0.000 | 0.000 | 0.000 | 0.000 | 0.000 | 0.000 | 0.000 | 0.000 | 0.000 |
| 16-246 | 0.000 | 0.000 | 0.000 | 0.000 | 0.000 | 0.000 | 0.000 | 0.000 | 0.042 | 0.048 | 0.043 | 0.000 | 0.250 | 0.042 | 0.000 | 0.048 | 0.000 | 0.000 | 0.043 | 0.000 | 0.000 | 0.043 |
| 16-254 | 0.000 | 0.000 | 0.045 | 0.167 | 0.063 | 0.091 | 0.000 | 0.000 | 0.250 | 0.048 | 0.087 | 0.045 | 0.083 | 0.000 | 0.050 | 0.048 | 0.000 | 0.000 | 0.043 | 0.043 | 0.182 | 0.000 |
| 16-257 | 0.500 | 0.050 | 0.091 | 0.250 | 0.063 | 0.000 | 0.000 | 0.143 | 0.292 | 0.333 | 0.348 | 0.000 | 0.000 | 0.208 | 0.350 | 0.048 | 0.500 | 0.625 | 0.130 | 0.087 | 0.364 | 0.217 |
| 16-264 | 0.000 | 0.100 | 0.000 | 0.000 | 0.000 | 0.000 | 0.000 | 0.000 | 0.000 | 0.048 | 0.000 | 0.045 | 0.042 | 0.000 | 0.000 | 0.048 | 0.000 | 0.000 | 0.000 | 0.000 | 0.000 | 0.000 |
| 16-269 | 0.000 | 0.100 | 0.000 | 0.000 | 0.000 | 0.000 | 0.000 | 0.048 | 0.000 | 0.048 | 0.043 | 0.000 | 0.083 | 0.000 | 0.000 | 0.143 | 0.042 | 0.000 | 0.000 | 0.000 | 0.000 | 0.000 |
| 16-276 | 0.045 | 0.000 | 0.182 | 0.167 | 0.000 | 0.455 | 0.000 | 0.095 | 0.292 | 0.524 | 0.261 | 0.000 | 0.458 | 0.083 | 0.150 | 0.095 | 0.083 | 0.000 | 0.000 | 0.000 | 0.045 | 0.261 |
| 16-280 | 0.000 | 0.000 | 0.045 | 0.042 | 0.000 | 0.000 | 0.000 | 0.000 | 0.000 | 0.000 | 0.000 | 0.000 | 0.042 | 0.000 | 0.050 | 0.048 | 0.000 | 0.000 | 0.000 | 0.000 | 0.000 | 0.000 |
| 16-286 | 0.000 | 0.000 | 0.227 | 0.000 | 0.000 | 0.318 | 0.000 | 0.143 | 0.583 | 0.476 | 0.261 | 0.000 | 0.042 | 0.000 | 0.000 | 0.000 | 0.000 | 0.000 | 0.000 | 0.000 | 0.045 | 0.000 |
| 16-289 | 0.955 | 0.300 | 0.818 | 0.708 | 0.625 | 0.545 | 0.909 | 0.952 | 0.792 | 0.857 | 0.870 | 0.773 | 0.375 | 0.542 | 0.850 | 0.857 | 0.417 | 0.458 | 0.652 | 0.826 | 0.273 | 0.739 |
| 16-292 | 0.091 | 0.350 | 0.727 | 0.458 | 0.250 | 0.545 | 0.136 | 0.810 | 0.833 | 0.810 | 0.783 | 0.227 | 0.500 | 0.375 | 0.100 | 0.000 | 0.542 | 0.542 | 0.304 | 0.174 | 0.727 | 0.174 |
| 16-297 | 0.000 | 0.000 | 0.273 | 0.458 | 0.000 | 0.091 | 0.000 | 0.000 | 0.875 | 0.810 | 0.304 | 0.000 | 0.000 | 0.000 | 0.000 | 0.000 | 0.000 | 0.000 | 0.000 | 0.087 | 0.000 | 0.130 |
| 16-305 | 0.000 | 0.100 | 0.091 | 0.125 | 0.000 | 0.091 | 0.000 | 0.000 | 0.417 | 0.429 | 0.087 | 0.045 | 0.000 | 0.042 | 0.000 | 0.000 | 0.083 | 0.000 | 0.043 | 0.000 | 0.045 | 0.217 |
| 16-309 | 0.000 | 0.000 | 0.091 | 0.000 | 0.000 | 0.000 | 0.000 | 0.000 | 0.375 | 0.286 | 0.043 | 0.000 | 0.167 | 0.083 | 0.050 | 0.000 | 0.000 | 0.000 | 0.000 | 0.087 | 0.000 | 0.043 |
| 16-314 | 0.000 | 0.000 | 0.136 | 0.083 | 0.000 | 0.045 | 0.000 | 0.000 | 0.292 | 0.381 | 0.043 | 0.000 | 0.042 | 0.000 | 0.000 | 0.000 | 0.000 | 0.000 | 0.043 | 0.000 | 0.000 | 0.000 |
| 16-320 | 1.000 | 0.650 | 0.182 | 0.875 | 0.500 | 0.591 | 0.545 | 0.429 | 0.667 | 0.238 | 0.174 | 0.727 | 0.875 | 0.750 | 0.550 | 0.905 | 0.958 | 0.917 | 0.870 | 0.957 | 1.000 | 0.826 |
| 16-325 | 0.000 | 0.100 | 0.045 | 0.000 | 0.000 | 0.000 | 0.000 | 0.000 | 0.125 | 0.143 | 0.000 | 0.000 | 0.042 | 0.000 | 0.000 | 0.000 | 0.000 | 0.000 | 0.000 | 0.043 | 0.000 | 0.000 |
| 16-331 | 0.136 | 0.100 | 0.091 | 0.583 | 0.188 | 0.364 | 0.136 | 0.143 | 0.542 | 0.238 | 0.087 | 0.136 | 0.000 | 0.167 | 0.150 | 0.000 | 0.042 | 0.000 | 0.000 | 0.000 | 0.136 | 0.043 |
| 16-334 | 0.000 | 0.150 | 0.045 | 0.167 | 0.000 | 0.000 | 0.045 | 0.048 | 0.667 | 0.333 | 0.087 | 0.000 | 0.125 | 0.083 | 0.050 | 0.048 | 0.125 | 0.000 | 0.043 | 0.130 | 0.182 | 0.087 |
| 16-337 | 0.227 | 0.100 | 0.091 | 0.208 | 0.000 | 0.000 | 0.182 | 0.000 | 0.042 | 0.000 | 0.565 | 0.000 | 0.042 | 0.167 | 0.150 | 0.048 | 0.333 | 0.167 | 0.000 | 0.087 | 0.000 | 0.043 |
| 16-340 | 0.045 | 0.000 | 0.045 | 0.042 | 0.250 | 0.136 | 0.091 | 0.048 | 0.417 | 0.476 | 0.087 | 0.045 | 0.083 | 0.083 | 0.050 | 0.095 | 0.042 | 0.042 | 0.000 | 0.130 | 0.091 | 0.174 |
| 16-343 | 0.045 | 0.000 | 0.000 | 0.167 | 0.063 | 0.000 | 0.000 | 0.048 | 0.375 | 0.190 | 0.000 | 0.000 | 0.125 | 0.000 | 0.050 | 0.000 | 0.125 | 0.000 | 0.000 | 0.000 | 0.000 | 0.043 |
| 16-347 | 0.136 | 0.150 | 0.000 | 0.250 | 0.000 | 0.045 | 0.045 | 0.048 | 0.375 | 0.238 | 0.000 | 0.045 | 0.083 | 0.000 | 0.000 | 0.000 | 0.042 | 0.000 | 0.043 | 0.043 | 0.045 | 0.000 |
| 16-351 | 0.045 | 0.200 | 0.091 | 0.000 | 0.000 | 0.000 | 0.045 | 0.000 | 0.375 | 0.333 | 0.217 | 0.000 | 0.000 | 0.000 | 0.050 | 0.095 | 0.000 | 0.042 | 0.000 | 0.000 | 0.091 | 0.000 |
| 16-354 | 0.000 | 0.000 | 0.000 | 0.083 | 0.000 | 0.000 | 0.000 | 0.000 | 0.000 | 0.000 | 0.000 | 0.000 | 0.083 | 0.000 | 0.000 | 0.000 | 0.042 | 0.000 | 0.000 | 0.000 | 0.000 | 0.000 |
| 16-360 | 0.000 | 0.000 | 0.045 | 0.000 | 0.000 | 0.000 | 0.000 | 0.000 | 0.083 | 0.048 | 0.000 | 0.000 | 0.000 | 0.000 | 0.050 | 0.048 | 0.000 | 0.000 | 0.000 | 0.000 | 0.000 | 0.043 |
| 16-365 | 0.000 | 0.250 | 0.000 | 0.083 | 0.188 | 0.000 | 0.000 | 0.000 | 0.000 | 0.000 | 0.000 | 0.000 | 0.083 | 0.000 | 0.000 | 0.048 | 0.083 | 0.042 | 0.043 | 0.043 | 0.000 | 0.087 |
| 16-370 | 0.000 | 0.050 | 0.045 | 0.000 | 0.000 | 0.000 | 0.000 | 0.000 | 0.000 | 0.000 | 0.000 | 0.136 | 0.167 | 0.000 | 0.000 | 0.000 | 0.000 | 0.000 | 0.000 | 0.043 | 0.000 | 0.043 |
| 16-374 | 0.045 | 0.000 | 0.000 | 0.083 | 0.000 | 0.045 | 0.000 | 0.000 | 0.000 | 0.000 | 0.000 | 0.045 | 0.083 | 0.000 | 0.000 | 0.000 | 0.000 | 0.000 | 0.000 | 0.130 | 0.000 | 0.043 |
| 16-382 | 0.045 | 0.100 | 0.000 | 0.083 | 0.000 | 0.000 | 0.045 | 0.048 | 0.292 | 0.095 | 0.000 | 0.182 | 0.000 | 0.000 | 0.000 | 0.000 | 0.000 | 0.000 | 0.043 | 0.000 | 0.000 | 0.000 |
| 16-385 | 0.000 | 0.000 | 0.000 | 0.167 | 0.000 | 0.091 | 0.045 | 0.000 | 0.208 | 0.048 | 0.000 | 0.045 | 0.125 | 0.042 | 0.000 | 0.000 | 0.000 | 0.000 | 0.000 | 0.000 | 0.273 | 0.000 |
| 16-389 | 0.045 | 0.200 | 0.045 | 0.042 | 0.000 | 0.045 | 0.000 | 0.000 | 0.167 | 0.095 | 0.000 | 0.045 | 0.000 | 0.000 | 0.000 | 0.000 | 0.000 | 0.000 | 0.000 | 0.000 | 0.091 | 0.000 |
| 16-392 | 0.318 | 0.200 | 0.182 | 0.292 | 0.125 | 0.273 | 0.136 | 0.048 | 0.708 | 0.524 | 0.348 | 0.136 | 0.125 | 0.167 | 0.100 | 0.381 | 0.208 | 0.083 | 0.043 | 0.565 | 0.091 | 0.087 |
| 16-396 | 0.045 | 0.200 | 0.091 | 0.292 | 0.188 | 0.000 | 0.000 | 0.000 | 0.542 | 0.524 | 0.217 | 0.000 | 0.042 | 0.083 | 0.250 | 0.238 | 0.167 | 0.000 | 0.043 | 0.217 | 0.045 | 0.174 |
| 16-401 | 0.045 | 0.000 | 0.000 | 0.000 | 0.063 | 0.000 | 0.000 | 0.000 | 0.042 | 0.000 | 0.043 | 0.000 | 0.042 | 0.000 | 0.000 | 0.000 | 0.000 | 0.042 | 0.043 | 0.000 | 0.045 | 0.000 |
| 16-404 | 0.000 | 0.000 | 0.000 | 0.208 | 0.063 | 0.273 | 0.045 | 0.000 | 0.125 | 0.000 | 0.000 | 0.045 | 0.042 | 0.000 | 0.000 | 0.000 | 0.083 | 0.167 | 0.000 | 0.000 | 0.273 | 0.043 |
| 16-410 | 0.000 | 0.050 | 0.045 | 0.042 | 0.125 | 0.045 | 0.000 | 0.000 | 0.042 | 0.000 | 0.087 | 0.000 | 0.000 | 0.000 | 0.000 | 0.095 | 0.000 | 0.042 | 0.000 | 0.000 | 0.000 | 0.000 |
| 16-416 | 0.000 | 0.000 | 0.000 | 0.000 | 0.000 | 0.000 | 0.000 | 0.000 | 0.000 | 0.048 | 0.043 | 0.000 | 0.000 | 0.042 | 0.000 | 0.048 | 0.000 | 0.000 | 0.000 | 0.043 | 0.000 | 0.000 |
| 16-418 | 0.000 | 0.100 | 0.045 | 0.000 | 0.063 | 0.000 | 0.000 | 0.000 | 0.250 | 0.286 | 0.174 | 0.000 | 0.000 | 0.000 | 0.050 | 0.000 | 0.000 | 0.000 | 0.000 | 0.000 | 0.000 | 0.000 |
| 16-423 | 1.000 | 0.700 | 0.773 | 0.958 | 0.875 | 0.955 | 0.909 | 0.857 | 0.875 | 0.810 | 0.696 | 1.000 | 0.833 | 0.958 | 1.000 | 0.952 | 1.000 | 1.000 | 0.957 | 0.913 | 0.955 | 1.000 |
| 16-428 | 1.000 | 0.500 | 0.591 | 1.000 | 0.813 | 0.864 | 0.818 | 0.857 | 0.750 | 0.667 | 0.391 | 1.000 | 0.792 | 0.958 | 1.000 | 0.810 | 0.875 | 1.000 | 0.957 | 0.957 | 1.000 | 0.913 |
| 16-434 | 0.045 | 0.150 | 0.000 | 0.208 | 0.188 | 0.227 | 0.318 | 0.143 | 0.250 | 0.048 | 0.174 | 0.000 | 0.083 | 0.125 | 0.050 | 0.095 | 0.208 | 0.000 | 0.043 | 0.043 | 0.273 | 0.043 |
| 16-437 | 0.682 | 0.450 | 0.000 | 0.333 | 0.313 | 0.045 | 0.045 | 0.048 | 0.208 | 0.190 | 0.174 | 0.318 | 0.458 | 0.458 | 0.300 | 0.381 | 0.708 | 0.167 | 0.000 | 0.696 | 0.591 | 0.304 |
| 16-442 | 0.000 | 0.100 | 0.045 | 0.000 | 0.000 | 0.000 | 0.000 | 0.000 | 0.000 | 0.000 | 0.043 | 0.000 | 0.000 | 0.042 | 0.050 | 0.048 | 0.000 | 0.000 | 0.000 | 0.043 | 0.000 | 0.000 |
| 16-446 | 0.000 | 0.000 | 0.000 | 0.000 | 0.000 | 0.000 | 0.000 | 0.048 | 0.000 | 0.000 | 0.000 | 0.000 | 0.167 | 0.000 | 0.100 | 0.048 | 0.042 | 0.000 | 0.000 | 0.043 | 0.000 | 0.043 |
| 16-449 | 0.000 | 0.000 | 0.227 | 0.000 | 0.000 | 0.000 | 0.000 | 0.000 | 0.458 | 0.524 | 0.087 | 0.000 | 0.167 | 0.042 | 0.000 | 0.000 | 0.042 | 0.000 | 0.000 | 0.043 | 0.000 | 0.000 |
| 16-455 | 0.000 | 0.050 | 0.000 | 0.000 | 0.000 | 0.000 | 0.000 | 0.000 | 0.000 | 0.000 | 0.043 | 0.000 | 0.042 | 0.083 | 0.000 | 0.000 | 0.000 | 0.000 | 0.000 | 0.130 | 0.000 | 0.043 |
| 16-459 | 0.409 | 0.000 | 0.000 | 0.208 | 0.063 | 0.000 | 0.182 | 0.000 | 0.125 | 0.048 | 0.174 | 0.000 | 0.042 | 0.208 | 0.050 | 0.048 | 0.250 | 0.000 | 0.000 | 0.043 | 0.045 | 0.000 |
| 16-463 | 0.000 | 0.450 | 0.045 | 0.000 | 0.000 | 0.000 | 0.000 | 0.000 | 0.375 | 0.333 | 0.870 | 0.000 | 0.000 | 0.083 | 0.000 | 0.095 | 0.042 | 0.000 | 0.087 | 0.000 | 0.091 | 0.000 |
| 16-469 | 0.045 | 0.050 | 0.000 | 0.000 | 0.000 | 0.000 | 0.000 | 0.000 | 0.000 | 0.000 | 0.000 | 0.136 | 0.000 | 0.000 | 0.000 | 0.048 | 0.000 | 0.000 | 0.000 | 0.043 | 0.000 | 0.000 |
| 16-472 | 0.091 | 0.050 | 0.045 | 0.375 | 0.063 | 0.000 | 0.091 | 0.000 | 0.458 | 0.048 | 0.043 | 0.045 | 0.167 | 0.250 | 0.250 | 0.000 | 0.208 | 0.000 | 0.043 | 0.087 | 0.045 | 0.000 |
| 16-479 | 0.227 | 0.150 | 0.045 | 0.125 | 0.625 | 0.182 | 0.045 | 0.048 | 0.375 | 0.143 | 0.000 | 0.455 | 0.125 | 0.125 | 0.050 | 0.095 | 0.125 | 0.292 | 0.348 | 0.261 | 0.455 | 0.304 |
| 16-485 | 0.000 | 0.050 | 0.000 | 0.000 | 0.063 | 0.000 | 0.045 | 0.000 | 0.042 | 0.048 | 0.000 | 0.000 | 0.000 | 0.000 | 0.000 | 0.000 | 0.000 | 0.000 | 0.000 | 0.000 | 0.045 | 0.217 |
| 16-491 | 0.045 | 0.050 | 0.000 | 0.000 | 0.000 | 0.000 | 0.045 | 0.000 | 0.083 | 0.000 | 0.000 | 0.000 | 0.042 | 0.042 | 0.000 | 0.000 | 0.000 | 0.083 | 0.043 | 0.087 | 0.000 | 0.000 |
| 16-499 | 0.045 | 0.050 | 0.000 | 0.000 | 0.063 | 0.000 | 0.000 | 0.000 | 0.042 | 0.048 | 0.000 | 0.045 | 0.042 | 0.000 | 0.000 | 0.000 | 0.042 | 0.000 | 0.000 | 0.000 | 0.045 | 0.043 |
| 16-507 | 0.045 | 0.000 | 0.000 | 0.000 | 0.000 | 0.000 | 0.000 | 0.000 | 0.042 | 0.000 | 0.000 | 0.045 | 0.167 | 0.000 | 0.000 | 0.000 | 0.000 | 0.000 | 0.000 | 0.000 | 0.000 | 0.043 |
| 16-510 | 0.045 | 0.000 | 0.000 | 0.250 | 0.250 | 0.136 | 0.000 | 0.048 | 0.208 | 0.048 | 0.087 | 0.045 | 0.208 | 0.000 | 0.000 | 0.000 | 0.333 | 0.000 | 0.043 | 0.000 | 0.182 | 0.000 |
| 16-512 | 0.136 | 0.000 | 0.045 | 0.042 | 0.000 | 0.091 | 0.000 | 0.000 | 0.250 | 0.190 | 0.087 | 0.000 | 0.000 | 0.042 | 0.050 | 0.048 | 0.292 | 0.083 | 0.043 | 0.043 | 0.318 | 0.043 |
| 16-516 | 0.409 | 0.600 | 0.000 | 0.167 | 0.000 | 0.045 | 0.227 | 0.000 | 0.417 | 0.286 | 0.000 | 0.091 | 0.458 | 0.042 | 0.050 | 0.619 | 0.083 | 0.708 | 0.174 | 0.435 | 0.136 | 0.087 |
| 16-520 | 0.000 | 0.000 | 0.000 | 0.000 | 0.063 | 0.045 | 0.000 | 0.000 | 0.000 | 0.000 | 0.000 | 0.000 | 0.083 | 0.000 | 0.050 | 0.000 | 0.042 | 0.042 | 0.000 | 0.043 | 0.227 | 0.174 |
| 16-524 | 0.000 | 0.100 | 0.000 | 0.083 | 0.125 | 0.000 | 0.000 | 0.000 | 0.000 | 0.095 | 0.000 | 0.000 | 0.000 | 0.000 | 0.000 | 0.000 | 0.000 | 0.000 | 0.000 | 0.000 | 0.000 | 0.000 |
| 16-527 | 0.045 | 0.050 | 0.000 | 0.000 | 0.000 | 0.000 | 0.000 | 0.000 | 0.250 | 0.000 | 0.000 | 0.045 | 0.000 | 0.000 | 0.000 | 0.000 | 0.042 | 0.042 | 0.000 | 0.000 | 0.045 | 0.000 |
| 16-532 | 0.045 | 0.050 | 0.000 | 0.000 | 0.000 | 0.000 | 0.000 | 0.000 | 0.042 | 0.000 | 0.000 | 0.000 | 0.042 | 0.000 | 0.000 | 0.000 | 0.083 | 0.000 | 0.000 | 0.043 | 0.000 | 0.000 |
| 16-537 | 0.000 | 0.050 | 0.000 | 0.042 | 0.000 | 0.000 | 0.000 | 0.000 | 0.083 | 0.048 | 0.000 | 0.045 | 0.083 | 0.000 | 0.000 | 0.048 | 0.000 | 0.083 | 0.043 | 0.000 | 0.091 | 0.087 |
| 16-539 | 0.045 | 0.150 | 0.000 | 0.042 | 0.063 | 0.000 | 0.000 | 0.000 | 0.000 | 0.000 | 0.000 | 0.000 | 0.042 | 0.000 | 0.000 | 0.095 | 0.000 | 0.542 | 0.000 | 0.130 | 0.318 | 0.130 |
| 16-545 | 0.045 | 0.000 | 0.000 | 0.000 | 0.125 | 0.000 | 0.000 | 0.000 | 0.000 | 0.000 | 0.000 | 0.000 | 0.000 | 0.000 | 0.000 | 0.095 | 0.000 | 0.042 | 0.043 | 0.217 | 0.045 | 0.130 |
| 16-551 | 0.000 | 0.000 | 0.000 | 0.000 | 0.000 | 0.000 | 0.000 | 0.000 | 0.000 | 0.000 | 0.000 | 0.000 | 0.083 | 0.000 | 0.000 | 0.000 | 0.000 | 0.000 | 0.000 | 0.000 | 0.000 | 0.000 |
| 16-558 | 0.000 | 0.050 | 0.045 | 0.125 | 0.063 | 0.000 | 0.000 | 0.000 | 0.542 | 0.190 | 0.043 | 0.045 | 0.000 | 0.000 | 0.000 | 0.000 | 0.000 | 0.000 | 0.174 | 0.000 | 0.318 | 0.000 |
| 16-562 | 0.000 | 0.000 | 0.000 | 0.000 | 0.000 | 0.000 | 0.000 | 0.000 | 0.292 | 0.095 | 0.000 | 0.091 | 0.083 | 0.000 | 0.000 | 0.000 | 0.083 | 0.042 | 0.000 | 0.000 | 0.045 | 0.043 |
| 16-576 | 0.455 | 0.200 | 0.000 | 0.333 | 0.125 | 0.182 | 0.045 | 0.095 | 0.500 | 0.238 | 0.043 | 0.091 | 0.125 | 0.250 | 0.250 | 0.048 | 0.792 | 0.375 | 0.217 | 0.000 | 0.182 | 0.043 |
| 16-579 | 0.182 | 0.100 | 0.000 | 0.333 | 0.125 | 0.227 | 0.227 | 0.048 | 0.000 | 0.000 | 0.000 | 0.091 | 0.083 | 0.083 | 0.100 | 0.000 | 0.042 | 0.000 | 0.000 | 0.000 | 0.273 | 0.000 |
| 16-585 | 0.045 | 0.050 | 0.000 | 0.042 | 0.063 | 0.000 | 0.000 | 0.048 | 0.000 | 0.000 | 0.000 | 0.000 | 0.125 | 0.000 | 0.000 | 0.000 | 0.083 | 0.083 | 0.000 | 0.000 | 0.045 | 0.043 |
| 16-598 | 0.000 | 0.100 | 0.000 | 0.000 | 0.000 | 0.000 | 0.000 | 0.000 | 0.000 | 0.000 | 0.000 | 0.000 | 0.000 | 0.042 | 0.000 | 0.000 | 0.000 | 0.083 | 0.043 | 0.000 | 0.000 | 0.043 |
| 16-610 | 0.000 | 0.000 | 0.000 | 0.000 | 0.000 | 0.000 | 0.000 | 0.000 | 0.000 | 0.000 | 0.087 | 0.000 | 0.000 | 0.000 | 0.000 | 0.000 | 0.042 | 0.000 | 0.000 | 0.000 | 0.000 | 0.000 |
| 16-615 | 0.000 | 0.100 | 0.000 | 0.000 | 0.063 | 0.000 | 0.000 | 0.000 | 0.125 | 0.000 | 0.087 | 0.000 | 0.000 | 0.000 | 0.000 | 0.000 | 0.000 | 0.000 | 0.000 | 0.000 | 0.045 | 0.000 |
| 16-619 | 0.182 | 0.200 | 0.318 | 0.667 | 0.250 | 0.182 | 0.182 | 0.000 | 0.542 | 0.667 | 0.130 | 0.364 | 0.250 | 0.583 | 0.450 | 0.143 | 0.167 | 0.375 | 0.478 | 0.087 | 0.682 | 0.087 |
| 16-624 | 0.045 | 0.050 | 0.000 | 0.000 | 0.063 | 0.000 | 0.000 | 0.000 | 0.667 | 0.762 | 0.000 | 0.000 | 0.000 | 0.000 | 0.000 | 0.000 | 0.000 | 0.042 | 0.087 | 0.000 | 0.000 | 0.043 |
| 16-631 | 0.000 | 0.000 | 0.000 | 0.042 | 0.000 | 0.000 | 0.000 | 0.000 | 0.208 | 0.143 | 0.000 | 0.000 | 0.167 | 0.000 | 0.000 | 0.000 | 0.083 | 0.083 | 0.043 | 0.000 | 0.000 | 0.000 |
| 16-639 | 0.000 | 0.050 | 0.000 | 0.000 | 0.063 | 0.045 | 0.000 | 0.000 | 0.000 | 0.048 | 0.000 | 0.000 | 0.042 | 0.042 | 0.000 | 0.000 | 0.000 | 0.000 | 0.000 | 0.000 | 0.000 | 0.000 |
| 16-655 | 0.045 | 0.000 | 0.000 | 0.000 | 0.063 | 0.000 | 0.000 | 0.000 | 0.000 | 0.000 | 0.000 | 0.000 | 0.125 | 0.042 | 0.000 | 0.000 | 0.000 | 0.000 | 0.000 | 0.000 | 0.045 | 0.000 |
| 16-659 | 0.000 | 0.000 | 0.000 | 0.042 | 0.000 | 0.000 | 0.000 | 0.000 | 0.000 | 0.000 | 0.000 | 0.045 | 0.000 | 0.000 | 0.000 | 0.000 | 0.042 | 0.042 | 0.000 | 0.130 | 0.045 | 0.087 |
| 16-668 | 0.000 | 0.000 | 0.000 | 0.000 | 0.000 | 0.000 | 0.000 | 0.000 | 0.000 | 0.000 | 0.000 | 0.000 | 0.000 | 0.000 | 0.050 | 0.000 | 0.000 | 0.000 | 0.087 | 0.087 | 0.000 | 0.000 |
| 16-679 | 0.000 | 0.050 | 0.000 | 0.000 | 0.000 | 0.000 | 0.000 | 0.000 | 0.000 | 0.000 | 0.000 | 0.000 | 0.042 | 0.000 | 0.000 | 0.000 | 0.000 | 0.000 | 0.087 | 0.043 | 0.000 | 0.000 |
| 16-691 | 0.000 | 0.050 | 0.000 | 0.000 | 0.000 | 0.000 | 0.000 | 0.000 | 0.000 | 0.000 | 0.000 | 0.000 | 0.000 | 0.000 | 0.000 | 0.000 | 0.000 | 0.000 | 0.000 | 0.000 | 0.000 | 0.000 |
| 16-703 | 0.000 | 0.000 | 0.000 | 0.000 | 0.063 | 0.000 | 0.000 | 0.000 | 0.167 | 0.000 | 0.000 | 0.000 | 0.125 | 0.000 | 0.000 | 0.000 | 0.000 | 0.125 | 0.043 | 0.000 | 0.364 | 0.000 |
| 16-708 | 0.000 | 0.050 | 0.000 | 0.000 | 0.000 | 0.000 | 0.000 | 0.000 | 0.000 | 0.048 | 0.000 | 0.000 | 0.042 | 0.000 | 0.000 | 0.000 | 0.000 | 0.000 | 0.000 | 0.000 | 0.000 | 0.000 |
| 16-719 | 0.045 | 0.050 | 0.000 | 0.042 | 0.063 | 0.000 | 0.000 | 0.000 | 0.000 | 0.000 | 0.000 | 0.045 | 0.000 | 0.000 | 0.100 | 0.000 | 0.042 | 0.000 | 0.000 | 0.000 | 0.136 | 0.000 |
| 16-723 | 0.091 | 0.000 | 0.000 | 0.000 | 0.063 | 0.000 | 0.000 | 0.000 | 0.000 | 0.000 | 0.000 | 0.045 | 0.000 | 0.000 | 0.000 | 0.000 | 0.250 | 0.000 | 0.000 | 0.000 | 0.000 | 0.000 |
| 16-733 | 0.000 | 0.000 | 0.045 | 0.000 | 0.000 | 0.000 | 0.000 | 0.000 | 0.000 | 0.000 | 0.000 | 0.000 | 0.208 | 0.000 | 0.000 | 0.000 | 0.000 | 0.000 | 0.000 | 0.043 | 0.000 | 0.043 |
| 16-740 | 0.000 | 0.050 | 0.000 | 0.000 | 0.000 | 0.045 | 0.000 | 0.000 | 0.000 | 0.000 | 0.000 | 0.000 | 0.083 | 0.000 | 0.000 | 0.000 | 0.000 | 0.000 | 0.043 | 0.043 | 0.045 | 0.000 |
| 16-747 | 0.000 | 0.000 | 0.000 | 0.000 | 0.000 | 0.000 | 0.000 | 0.000 | 0.000 | 0.000 | 0.043 | 0.000 | 0.042 | 0.000 | 0.000 | 0.000 | 0.000 | 0.000 | 0.000 | 0.000 | 0.000 | 0.000 |
| 16-756 | 0.000 | 0.100 | 0.000 | 0.000 | 0.000 | 0.000 | 0.000 | 0.000 | 0.000 | 0.000 | 0.000 | 0.045 | 0.083 | 0.000 | 0.000 | 0.000 | 0.000 | 0.083 | 0.000 | 0.043 | 0.318 | 0.000 |
| 16-766 | 0.000 | 0.000 | 0.000 | 0.000 | 0.000 | 0.000 | 0.000 | 0.000 | 0.000 | 0.000 | 0.000 | 0.000 | 0.000 | 0.000 | 0.000 | 0.048 | 0.000 | 0.000 | 0.000 | 0.000 | 0.000 | 0.043 |
| 16-772 | 0.000 | 0.000 | 0.000 | 0.000 | 0.000 | 0.000 | 0.000 | 0.000 | 0.000 | 0.000 | 0.000 | 0.000 | 0.000 | 0.000 | 0.000 | 0.000 | 0.000 | 0.000 | 0.000 | 0.000 | 0.000 | 0.043 |
| 16-778 | 0.864 | 0.500 | 0.136 | 0.625 | 0.813 | 0.318 | 0.364 | 0.143 | 0.292 | 0.048 | 0.000 | 0.818 | 0.750 | 0.500 | 0.450 | 0.857 | 1.000 | 0.875 | 0.957 | 0.870 | 1.000 | 0.783 |
| 16-791 | 0.136 | 0.000 | 0.000 | 0.042 | 0.063 | 0.000 | 0.000 | 0.000 | 0.000 | 0.000 | 0.000 | 0.000 | 0.000 | 0.000 | 0.050 | 0.000 | 0.083 | 0.000 | 0.000 | 0.043 | 0.000 | 0.000 |
| 16-799 | 0.000 | 0.000 | 0.000 | 0.000 | 0.000 | 0.000 | 0.000 | 0.000 | 0.000 | 0.000 | 0.000 | 0.000 | 0.000 | 0.042 | 0.000 | 0.000 | 0.000 | 0.000 | 0.000 | 0.043 | 0.045 | 0.043 |
| 16-808 | 0.000 | 0.000 | 0.000 | 0.083 | 0.000 | 0.045 | 0.000 | 0.000 | 0.000 | 0.000 | 0.043 | 0.227 | 0.125 | 0.000 | 0.000 | 0.000 | 0.167 | 0.208 | 0.087 | 0.000 | 0.318 | 0.000 |
| 16-817 | 0.136 | 0.000 | 0.000 | 0.083 | 0.000 | 0.000 | 0.000 | 0.000 | 0.000 | 0.000 | 0.000 | 0.091 | 0.042 | 0.000 | 0.050 | 0.048 | 0.333 | 0.125 | 0.130 | 0.000 | 0.000 | 0.130 |
| 16-822 | 0.000 | 0.000 | 0.000 | 0.000 | 0.063 | 0.000 | 0.000 | 0.000 | 0.000 | 0.000 | 0.000 | 0.045 | 0.125 | 0.000 | 0.000 | 0.000 | 0.000 | 0.000 | 0.000 | 0.000 | 0.000 | 0.000 |
| 16-833 | 0.000 | 0.000 | 0.000 | 0.000 | 0.000 | 0.000 | 0.000 | 0.000 | 0.000 | 0.000 | 0.000 | 0.000 | 0.125 | 0.000 | 0.000 | 0.000 | 0.000 | 0.000 | 0.000 | 0.000 | 0.000 | 0.043 |
| 16-836 | 0.000 | 0.000 | 0.000 | 0.000 | 0.000 | 0.000 | 0.000 | 0.000 | 0.000 | 0.000 | 0.000 | 0.000 | 0.000 | 0.000 | 0.000 | 0.000 | 0.125 | 0.000 | 0.000 | 0.000 | 0.000 | 0.043 |
| 16-842 | 0.000 | 0.100 | 0.000 | 0.000 | 0.000 | 0.000 | 0.000 | 0.000 | 0.000 | 0.000 | 0.000 | 0.000 | 0.000 | 0.000 | 0.000 | 0.000 | 0.000 | 0.000 | 0.000 | 0.000 | 0.000 | 0.043 |
| 16-846 | 0.000 | 0.050 | 0.000 | 0.000 | 0.063 | 0.000 | 0.000 | 0.000 | 0.000 | 0.000 | 0.000 | 0.000 | 0.000 | 0.000 | 0.000 | 0.000 | 0.000 | 0.000 | 0.000 | 0.000 | 0.000 | 0.000 |
| 16-858 | 0.000 | 0.000 | 0.000 | 0.000 | 0.063 | 0.000 | 0.000 | 0.000 | 0.000 | 0.000 | 0.000 | 0.000 | 0.042 | 0.000 | 0.000 | 0.000 | 0.000 | 0.000 | 0.000 | 0.000 | 0.091 | 0.000 |
| 16-862 | 0.136 | 0.050 | 0.000 | 0.000 | 0.000 | 0.000 | 0.000 | 0.000 | 0.000 | 0.000 | 0.000 | 0.000 | 0.000 | 0.000 | 0.000 | 0.000 | 0.292 | 0.083 | 0.000 | 0.000 | 0.000 | 0.000 |
| 16-873 | 0.000 | 0.000 | 0.000 | 0.000 | 0.000 | 0.000 | 0.000 | 0.000 | 0.000 | 0.000 | 0.000 | 0.000 | 0.000 | 0.042 | 0.000 | 0.000 | 0.042 | 0.083 | 0.000 | 0.000 | 0.000 | 0.000 |
| 16-877 | 0.682 | 0.050 | 0.000 | 0.167 | 0.063 | 0.045 | 0.000 | 0.000 | 0.083 | 0.000 | 0.043 | 0.545 | 0.750 | 0.125 | 0.300 | 0.190 | 0.708 | 0.542 | 0.609 | 0.130 | 0.864 | 0.217 |
| 16-883 | 0.727 | 0.100 | 0.045 | 0.292 | 0.188 | 0.045 | 0.000 | 0.000 | 0.125 | 0.048 | 0.000 | 0.500 | 0.792 | 0.167 | 0.250 | 0.238 | 0.792 | 0.542 | 0.783 | 0.174 | 0.864 | 0.304 |
| 16-887 | 0.000 | 0.000 | 0.000 | 0.000 | 0.000 | 0.000 | 0.000 | 0.000 | 0.000 | 0.000 | 0.000 | 0.000 | 0.000 | 0.042 | 0.000 | 0.000 | 0.000 | 0.000 | 0.000 | 0.000 | 0.000 | 0.043 |
| 16-896 | 0.000 | 0.000 | 0.000 | 0.000 | 0.000 | 0.000 | 0.000 | 0.000 | 0.000 | 0.000 | 0.000 | 0.000 | 0.000 | 0.000 | 0.000 | 0.000 | 0.000 | 0.000 | 0.000 | 0.043 | 0.000 | 0.043 |
| 16-919 | 0.000 | 0.000 | 0.000 | 0.000 | 0.000 | 0.000 | 0.000 | 0.000 | 0.000 | 0.000 | 0.000 | 0.000 | 0.042 | 0.000 | 0.000 | 0.000 | 0.000 | 0.000 | 0.000 | 0.000 | 0.000 | 0.000 |
| 16-923 | 0.000 | 0.000 | 0.000 | 0.000 | 0.000 | 0.000 | 0.000 | 0.000 | 0.000 | 0.000 | 0.000 | 0.000 | 0.042 | 0.000 | 0.050 | 0.000 | 0.042 | 0.042 | 0.000 | 0.000 | 0.091 | 0.000 |
| 16-926 | 0.000 | 0.000 | 0.000 | 0.000 | 0.000 | 0.000 | 0.000 | 0.000 | 0.000 | 0.000 | 0.000 | 0.045 | 0.000 | 0.042 | 0.000 | 0.000 | 0.042 | 0.042 | 0.000 | 0.000 | 0.091 | 0.000 |
| 16-929 | 0.000 | 0.000 | 0.000 | 0.000 | 0.000 | 0.000 | 0.000 | 0.000 | 0.000 | 0.000 | 0.000 | 0.045 | 0.083 | 0.000 | 0.000 | 0.000 | 0.042 | 0.083 | 0.000 | 0.000 | 0.409 | 0.000 |
| 16-959 | 0.000 | 0.050 | 0.000 | 0.000 | 0.000 | 0.000 | 0.000 | 0.000 | 0.000 | 0.000 | 0.000 | 0.000 | 0.000 | 0.000 | 0.000 | 0.000 | 0.000 | 0.000 | 0.000 | 0.000 | 0.000 | 0.000 |
| 16-965 | 0.000 | 0.000 | 0.000 | 0.000 | 0.000 | 0.000 | 0.000 | 0.000 | 0.000 | 0.000 | 0.000 | 0.000 | 0.000 | 0.000 | 0.050 | 0.000 | 0.000 | 0.000 | 0.000 | 0.000 | 0.000 | 0.000 |
| 16-971 | 0.000 | 0.000 | 0.000 | 0.000 | 0.063 | 0.000 | 0.000 | 0.000 | 0.000 | 0.000 | 0.000 | 0.000 | 0.000 | 0.000 | 0.000 | 0.048 | 0.000 | 0.000 | 0.000 | 0.000 | 0.000 | 0.043 |
| 16-981 | 0.000 | 0.000 | 0.000 | 0.000 | 0.000 | 0.000 | 0.000 | 0.000 | 0.000 | 0.000 | 0.000 | 0.045 | 0.083 | 0.000 | 0.000 | 0.048 | 0.083 | 0.042 | 0.043 | 0.000 | 0.227 | 0.043 |
| 16-1000 | 0.000 | 0.050 | 0.000 | 0.000 | 0.000 | 0.000 | 0.000 | 0.000 | 0.000 | 0.000 | 0.000 | 0.000 | 0.083 | 0.000 | 0.000 | 0.048 | 0.250 | 0.000 | 0.000 | 0.000 | 0.000 | 0.043 |
| 16-1024 | 0.000 | 0.000 | 0.000 | 0.000 | 0.000 | 0.000 | 0.000 | 0.000 | 0.000 | 0.000 | 0.000 | 0.000 | 0.042 | 0.000 | 0.000 | 0.000 | 0.000 | 0.000 | 0.000 | 0.000 | 0.000 | 0.000 |
| 16-1035 | 0.000 | 0.050 | 0.000 | 0.000 | 0.000 | 0.000 | 0.000 | 0.000 | 0.000 | 0.000 | 0.000 | 0.000 | 0.000 | 0.000 | 0.000 | 0.000 | 0.000 | 0.000 | 0.000 | 0.000 | 0.000 | 0.000 |
| 16-1040 | 0.000 | 0.050 | 0.000 | 0.000 | 0.000 | 0.000 | 0.000 | 0.000 | 0.000 | 0.000 | 0.000 | 0.000 | 0.000 | 0.000 | 0.000 | 0.000 | 0.000 | 0.000 | 0.000 | 0.000 | 0.000 | 0.000 |
| 16-1073 | 0.000 | 0.000 | 0.000 | 0.000 | 0.000 | 0.000 | 0.000 | 0.000 | 0.000 | 0.000 | 0.000 | 0.000 | 0.000 | 0.000 | 0.000 | 0.048 | 0.042 | 0.000 | 0.000 | 0.000 | 0.000 | 0.000 |
| 16-1094 | 0.000 | 0.000 | 0.000 | 0.000 | 0.000 | 0.000 | 0.000 | 0.000 | 0.000 | 0.000 | 0.000 | 0.000 | 0.000 | 0.000 | 0.000 | 0.000 | 0.042 | 0.000 | 0.000 | 0.000 | 0.000 | 0.000 |
| 16-1101 | 0.000 | 0.000 | 0.000 | 0.000 | 0.000 | 0.000 | 0.000 | 0.000 | 0.000 | 0.000 | 0.000 | 0.000 | 0.000 | 0.000 | 0.000 | 0.000 | 0.042 | 0.000 | 0.000 | 0.000 | 0.000 | 0.000 |
| 16-1112 | 0.000 | 0.000 | 0.000 | 0.000 | 0.063 | 0.000 | 0.000 | 0.000 | 0.000 | 0.000 | 0.000 | 0.000 | 0.000 | 0.000 | 0.000 | 0.000 | 0.000 | 0.000 | 0.000 | 0.000 | 0.000 | 0.000 |
| 16-1121 | 0.045 | 0.000 | 0.000 | 0.000 | 0.000 | 0.000 | 0.000 | 0.000 | 0.000 | 0.000 | 0.000 | 0.000 | 0.000 | 0.000 | 0.000 | 0.000 | 0.042 | 0.000 | 0.000 | 0.000 | 0.000 | 0.000 |
| 16-1176 | 0.000 | 0.000 | 0.000 | 0.000 | 0.000 | 0.000 | 0.000 | 0.000 | 0.000 | 0.000 | 0.000 | 0.000 | 0.000 | 0.000 | 0.000 | 0.000 | 0.083 | 0.000 | 0.000 | 0.000 | 0.000 | 0.000 |
| 16-1189 | 0.000 | 0.000 | 0.000 | 0.000 | 0.000 | 0.000 | 0.000 | 0.000 | 0.000 | 0.000 | 0.000 | 0.000 | 0.000 | 0.000 | 0.000 | 0.000 | 0.042 | 0.000 | 0.000 | 0.000 | 0.000 | 0.000 |
| 18-103 | 0.000 | 0.150 | 0.053 | 0.000 | 0.056 | 0.045 | 0.000 | 0.000 | 0.043 | 0.050 | 0.000 | 0.000 | 0.000 | 0.000 | 0.000 | 0.000 | 0.000 | 0.000 | 0.000 | 0.042 | 0.000 | 0.000 |
| 18-106 | 0.000 | 0.050 | 0.000 | 0.000 | 0.000 | 0.000 | 0.000 | 0.000 | 0.000 | 0.000 | 0.000 | 0.000 | 0.000 | 0.000 | 0.050 | 0.000 | 0.000 | 0.042 | 0.000 | 0.083 | 0.136 | 0.000 |
| 18-116 | 0.000 | 0.100 | 0.000 | 0.000 | 0.000 | 0.000 | 0.000 | 0.000 | 0.000 | 0.000 | 0.000 | 0.000 | 0.042 | 0.000 | 0.000 | 0.000 | 0.000 | 0.000 | 0.000 | 0.042 | 0.045 | 0.000 |
| 18-122 | 0.000 | 0.050 | 0.000 | 0.000 | 0.056 | 0.000 | 0.000 | 0.000 | 0.000 | 0.000 | 0.000 | 0.000 | 0.000 | 0.000 | 0.000 | 0.048 | 0.000 | 0.042 | 0.000 | 0.083 | 0.136 | 0.000 |
| 18-126 | 0.000 | 0.000 | 0.000 | 0.000 | 0.000 | 0.000 | 0.000 | 0.000 | 0.043 | 0.000 | 0.000 | 0.000 | 0.000 | 0.000 | 0.000 | 0.000 | 0.000 | 0.000 | 0.000 | 0.083 | 0.045 | 0.000 |
| 18-134 | 0.000 | 0.000 | 0.000 | 0.000 | 0.000 | 0.000 | 0.000 | 0.000 | 0.000 | 0.000 | 0.000 | 0.000 | 0.042 | 0.000 | 0.000 | 0.000 | 0.000 | 0.042 | 0.000 | 0.083 | 0.227 | 0.000 |
| 18-141 | 0.000 | 0.000 | 0.000 | 0.000 | 0.000 | 0.000 | 0.000 | 0.000 | 0.000 | 0.000 | 0.000 | 0.000 | 0.000 | 0.000 | 0.000 | 0.000 | 0.000 | 0.000 | 0.000 | 0.083 | 0.045 | 0.043 |
| 18-146 | 0.000 | 0.100 | 0.000 | 0.000 | 0.000 | 0.045 | 0.043 | 0.000 | 0.000 | 0.000 | 0.000 | 0.000 | 0.042 | 0.000 | 0.000 | 0.000 | 0.000 | 0.083 | 0.000 | 0.083 | 0.318 | 0.000 |
| 18-152 | 0.000 | 0.000 | 0.000 | 0.000 | 0.000 | 0.000 | 0.000 | 0.000 | 0.000 | 0.000 | 0.000 | 0.000 | 0.000 | 0.000 | 0.000 | 0.048 | 0.000 | 0.000 | 0.000 | 0.042 | 0.045 | 0.217 |
| 18-160 | 0.000 | 0.050 | 0.000 | 0.000 | 0.000 | 0.000 | 0.000 | 0.000 | 0.000 | 0.000 | 0.000 | 0.000 | 0.000 | 0.000 | 0.050 | 0.000 | 0.000 | 0.083 | 0.000 | 0.042 | 0.000 | 0.000 |
| 18-168 | 0.000 | 0.000 | 0.000 | 0.000 | 0.000 | 0.000 | 0.000 | 0.000 | 0.000 | 0.000 | 0.053 | 0.000 | 0.083 | 0.000 | 0.000 | 0.000 | 0.000 | 0.042 | 0.000 | 0.083 | 0.000 | 0.000 |
| 18-176 | 0.000 | 0.150 | 0.000 | 0.000 | 0.000 | 0.000 | 0.000 | 0.000 | 0.000 | 0.000 | 0.000 | 0.000 | 0.167 | 0.000 | 0.100 | 0.048 | 0.000 | 0.167 | 0.000 | 0.375 | 0.227 | 0.217 |
| 18-181 | 0.000 | 0.100 | 0.000 | 0.000 | 0.000 | 0.000 | 0.000 | 0.000 | 0.043 | 0.000 | 0.000 | 0.000 | 0.042 | 0.000 | 0.000 | 0.000 | 0.000 | 0.000 | 0.000 | 0.042 | 0.000 | 0.000 |
| 18-186 | 0.000 | 0.000 | 0.000 | 0.000 | 0.000 | 0.000 | 0.000 | 0.000 | 0.000 | 0.000 | 0.000 | 0.000 | 0.000 | 0.000 | 0.000 | 0.048 | 0.000 | 0.000 | 0.000 | 0.083 | 0.000 | 0.000 |
| 18-196 | 0.000 | 0.000 | 0.000 | 0.048 | 0.000 | 0.000 | 0.000 | 0.000 | 0.000 | 0.000 | 0.000 | 0.042 | 0.208 | 0.000 | 0.000 | 0.095 | 0.000 | 0.292 | 0.000 | 0.083 | 0.182 | 0.087 |
| 18-210 | 0.182 | 0.550 | 0.000 | 0.476 | 0.111 | 0.000 | 0.696 | 0.000 | 0.043 | 0.000 | 0.000 | 0.125 | 0.833 | 0.083 | 0.200 | 0.667 | 0.250 | 0.917 | 0.125 | 0.958 | 0.773 | 0.826 |
| 18-224 | 0.773 | 0.350 | 0.000 | 0.238 | 0.778 | 0.227 | 0.522 | 0.300 | 0.565 | 0.550 | 0.737 | 0.292 | 0.042 | 0.250 | 0.650 | 0.714 | 0.958 | 0.125 | 0.208 | 0.500 | 0.136 | 0.130 |
| 18-236 | 0.000 | 0.000 | 0.000 | 0.000 | 0.000 | 0.000 | 0.000 | 0.000 | 0.000 | 0.000 | 0.000 | 0.167 | 0.250 | 0.000 | 0.000 | 0.000 | 0.000 | 0.000 | 0.000 | 0.042 | 0.000 | 0.043 |
| 18-245 | 0.545 | 0.650 | 0.211 | 0.667 | 0.500 | 0.182 | 0.783 | 0.150 | 0.435 | 0.050 | 0.158 | 0.542 | 0.875 | 0.375 | 0.200 | 0.810 | 0.833 | 0.917 | 0.458 | 0.958 | 0.909 | 0.913 |
| 18-253 | 0.045 | 0.350 | 0.000 | 0.048 | 0.000 | 0.000 | 0.217 | 0.000 | 0.043 | 0.000 | 0.053 | 0.042 | 0.292 | 0.000 | 0.150 | 0.571 | 0.125 | 0.208 | 0.000 | 0.750 | 0.182 | 0.826 |
| 18-258 | 0.000 | 0.000 | 0.000 | 0.000 | 0.000 | 0.000 | 0.000 | 0.000 | 0.000 | 0.000 | 0.000 | 0.042 | 0.042 | 0.000 | 0.000 | 0.000 | 0.000 | 0.000 | 0.000 | 0.000 | 0.000 | 0.130 |
| 18-262 | 0.000 | 0.250 | 0.000 | 0.048 | 0.000 | 0.000 | 0.043 | 0.000 | 0.000 | 0.000 | 0.000 | 0.000 | 0.000 | 0.042 | 0.050 | 0.000 | 0.000 | 0.083 | 0.000 | 0.167 | 0.227 | 0.043 |
| 18-270 | 0.000 | 0.150 | 0.000 | 0.000 | 0.000 | 0.000 | 0.000 | 0.000 | 0.000 | 0.000 | 0.000 | 0.042 | 0.292 | 0.000 | 0.050 | 0.000 | 0.000 | 0.000 | 0.000 | 0.042 | 0.000 | 0.000 |
| 18-276 | 0.000 | 0.100 | 0.000 | 0.000 | 0.000 | 0.000 | 0.000 | 0.000 | 0.000 | 0.000 | 0.000 | 0.000 | 0.083 | 0.000 | 0.000 | 0.000 | 0.000 | 0.000 | 0.000 | 0.083 | 0.000 | 0.087 |
| 18-285 | 0.000 | 0.150 | 0.000 | 0.000 | 0.000 | 0.000 | 0.000 | 0.000 | 0.000 | 0.000 | 0.000 | 0.000 | 0.042 | 0.000 | 0.000 | 0.000 | 0.000 | 0.000 | 0.000 | 0.042 | 0.000 | 0.000 |
| 18-294 | 0.000 | 0.000 | 0.000 | 0.000 | 0.000 | 0.000 | 0.000 | 0.000 | 0.000 | 0.000 | 0.000 | 0.000 | 0.167 | 0.000 | 0.050 | 0.000 | 0.000 | 0.125 | 0.000 | 0.125 | 0.000 | 0.043 |
| 18-301 | 0.045 | 0.450 | 0.000 | 0.095 | 0.000 | 0.000 | 0.174 | 0.000 | 0.000 | 0.000 | 0.000 | 0.042 | 0.417 | 0.208 | 0.050 | 0.476 | 0.083 | 0.542 | 0.000 | 0.458 | 0.091 | 0.478 |
| 18-306 | 0.000 | 0.300 | 0.000 | 0.048 | 0.000 | 0.000 | 0.130 | 0.000 | 0.000 | 0.000 | 0.000 | 0.000 | 0.333 | 0.000 | 0.100 | 0.238 | 0.042 | 0.125 | 0.000 | 0.292 | 0.182 | 0.174 |
| 18-310 | 0.000 | 0.150 | 0.000 | 0.000 | 0.000 | 0.000 | 0.000 | 0.000 | 0.000 | 0.000 | 0.000 | 0.000 | 0.042 | 0.000 | 0.000 | 0.000 | 0.000 | 0.000 | 0.000 | 0.042 | 0.000 | 0.000 |
| 18-314 | 0.091 | 0.150 | 0.000 | 0.000 | 0.333 | 0.045 | 0.000 | 0.000 | 0.043 | 0.000 | 0.000 | 0.208 | 0.792 | 0.250 | 0.200 | 0.667 | 0.250 | 0.708 | 0.333 | 0.833 | 0.818 | 0.783 |
| 18-317 | 0.182 | 0.600 | 0.000 | 0.476 | 0.333 | 0.091 | 0.391 | 0.000 | 0.087 | 0.000 | 0.053 | 0.417 | 0.792 | 0.250 | 0.250 | 0.429 | 0.333 | 0.625 | 0.542 | 0.833 | 0.818 | 0.783 |
| 18-321 | 0.000 | 0.100 | 0.000 | 0.000 | 0.000 | 0.000 | 0.000 | 0.000 | 0.000 | 0.000 | 0.000 | 0.000 | 0.083 | 0.000 | 0.050 | 0.238 | 0.000 | 0.125 | 0.000 | 0.292 | 0.182 | 0.478 |
| 18-328 | 0.364 | 0.250 | 0.000 | 0.333 | 0.389 | 0.000 | 0.304 | 0.000 | 0.174 | 0.000 | 0.000 | 0.250 | 0.708 | 0.083 | 0.150 | 0.476 | 0.333 | 0.792 | 0.333 | 0.625 | 0.773 | 0.826 |
| 18-331 | 0.182 | 0.300 | 0.053 | 0.000 | 0.278 | 0.000 | 0.087 | 0.000 | 0.000 | 0.000 | 0.000 | 0.125 | 0.667 | 0.042 | 0.150 | 0.476 | 0.208 | 0.542 | 0.208 | 0.875 | 0.591 | 0.783 |
| 18-335 | 0.000 | 0.000 | 0.000 | 0.000 | 0.000 | 0.045 | 0.000 | 0.000 | 0.000 | 0.000 | 0.000 | 0.000 | 0.167 | 0.042 | 0.050 | 0.048 | 0.000 | 0.042 | 0.000 | 0.042 | 0.045 | 0.087 |
| 18-341 | 0.000 | 0.000 | 0.000 | 0.000 | 0.056 | 0.000 | 0.000 | 0.000 | 0.000 | 0.000 | 0.000 | 0.000 | 0.167 | 0.000 | 0.000 | 0.095 | 0.000 | 0.083 | 0.000 | 0.083 | 0.045 | 0.043 |
| 18-346 | 0.000 | 0.050 | 0.000 | 0.000 | 0.000 | 0.000 | 0.000 | 0.000 | 0.000 | 0.000 | 0.053 | 0.000 | 0.083 | 0.000 | 0.000 | 0.000 | 0.000 | 0.042 | 0.000 | 0.042 | 0.000 | 0.043 |
| 18-352 | 0.909 | 0.250 | 0.000 | 0.190 | 0.444 | 0.000 | 0.261 | 0.150 | 0.391 | 0.150 | 0.105 | 0.042 | 0.250 | 0.000 | 0.200 | 0.429 | 0.667 | 0.083 | 0.042 | 0.333 | 0.091 | 0.391 |
| 18-358 | 0.091 | 0.500 | 0.000 | 0.095 | 0.111 | 0.000 | 0.174 | 0.000 | 0.000 | 0.000 | 0.000 | 0.042 | 0.792 | 0.000 | 0.100 | 0.619 | 0.125 | 0.875 | 0.000 | 0.875 | 0.727 | 0.783 |
| 18-362 | 0.000 | 0.150 | 0.000 | 0.000 | 0.000 | 0.000 | 0.000 | 0.000 | 0.000 | 0.000 | 0.000 | 0.000 | 0.042 | 0.000 | 0.000 | 0.095 | 0.000 | 0.250 | 0.000 | 0.042 | 0.045 | 0.043 |
| 18-368 | 0.000 | 0.050 | 0.000 | 0.000 | 0.000 | 0.045 | 0.043 | 0.000 | 0.000 | 0.000 | 0.000 | 0.167 | 0.458 | 0.000 | 0.050 | 0.238 | 0.000 | 0.250 | 0.000 | 0.042 | 0.136 | 0.261 |
| 18-375 | 0.045 | 0.300 | 0.105 | 0.000 | 0.000 | 0.000 | 0.043 | 0.000 | 0.000 | 0.000 | 0.000 | 0.292 | 0.583 | 0.125 | 0.100 | 0.286 | 0.125 | 0.667 | 0.125 | 0.167 | 0.136 | 0.696 |
| 18-385 | 0.364 | 0.300 | 0.053 | 0.619 | 0.556 | 0.318 | 0.217 | 0.100 | 0.391 | 0.050 | 0.000 | 0.500 | 1.000 | 0.583 | 0.300 | 0.857 | 0.625 | 0.750 | 0.708 | 0.750 | 0.955 | 0.826 |
| 18-389 | 0.273 | 0.300 | 0.000 | 0.286 | 0.444 | 0.000 | 0.174 | 0.000 | 0.000 | 0.000 | 0.000 | 0.500 | 0.958 | 0.542 | 0.200 | 0.762 | 0.542 | 0.750 | 0.792 | 0.708 | 0.909 | 0.826 |
| 18-393 | 0.000 | 0.050 | 0.000 | 0.000 | 0.000 | 0.000 | 0.000 | 0.100 | 0.000 | 0.000 | 0.000 | 0.083 | 0.292 | 0.000 | 0.000 | 0.000 | 0.000 | 0.000 | 0.000 | 0.042 | 0.000 | 0.000 |
| 18-401 | 0.000 | 0.050 | 0.000 | 0.000 | 0.000 | 0.000 | 0.000 | 0.000 | 0.000 | 0.000 | 0.000 | 0.000 | 0.250 | 0.000 | 0.100 | 0.000 | 0.000 | 0.000 | 0.000 | 0.000 | 0.000 | 0.043 |
| 18-406 | 0.000 | 0.050 | 0.000 | 0.000 | 0.000 | 0.000 | 0.000 | 0.000 | 0.000 | 0.000 | 0.000 | 0.000 | 0.250 | 0.042 | 0.000 | 0.000 | 0.000 | 0.000 | 0.000 | 0.000 | 0.000 | 0.043 |
| 18-414 | 0.000 | 0.050 | 0.000 | 0.000 | 0.000 | 0.000 | 0.000 | 0.000 | 0.000 | 0.000 | 0.000 | 0.000 | 0.333 | 0.000 | 0.000 | 0.000 | 0.000 | 0.042 | 0.000 | 0.000 | 0.000 | 0.087 |
| 18-418 | 0.000 | 0.200 | 0.000 | 0.000 | 0.056 | 0.000 | 0.000 | 0.000 | 0.000 | 0.000 | 0.000 | 0.042 | 0.083 | 0.000 | 0.000 | 0.143 | 0.042 | 0.375 | 0.042 | 0.292 | 0.091 | 0.174 |
| 18-427 | 0.000 | 0.050 | 0.000 | 0.000 | 0.000 | 0.000 | 0.000 | 0.000 | 0.043 | 0.000 | 0.000 | 0.000 | 0.292 | 0.042 | 0.000 | 0.000 | 0.000 | 0.000 | 0.000 | 0.000 | 0.000 | 0.000 |
| 18-435 | 0.000 | 0.000 | 0.000 | 0.000 | 0.056 | 0.000 | 0.000 | 0.000 | 0.000 | 0.000 | 0.000 | 0.000 | 0.292 | 0.000 | 0.000 | 0.000 | 0.000 | 0.000 | 0.000 | 0.042 | 0.136 | 0.000 |
| 18-439 | 0.000 | 0.000 | 0.000 | 0.000 | 0.000 | 0.000 | 0.000 | 0.000 | 0.000 | 0.000 | 0.000 | 0.083 | 0.208 | 0.000 | 0.100 | 0.190 | 0.000 | 0.000 | 0.000 | 0.167 | 0.045 | 0.391 |
| 18-444 | 0.045 | 0.100 | 0.000 | 0.000 | 0.000 | 0.000 | 0.000 | 0.000 | 0.000 | 0.000 | 0.000 | 0.000 | 0.125 | 0.000 | 0.000 | 0.286 | 0.000 | 0.042 | 0.042 | 0.208 | 0.000 | 0.217 |
| 18-449 | 0.000 | 0.050 | 0.000 | 0.048 | 0.056 | 0.000 | 0.043 | 0.000 | 0.000 | 0.100 | 0.000 | 0.083 | 0.167 | 0.000 | 0.000 | 0.429 | 0.000 | 0.208 | 0.000 | 0.583 | 0.136 | 0.217 |
| 18-453 | 0.136 | 0.150 | 0.000 | 0.048 | 0.333 | 0.000 | 0.043 | 0.000 | 0.000 | 0.000 | 0.000 | 0.042 | 0.292 | 0.167 | 0.000 | 0.048 | 0.125 | 0.333 | 0.042 | 0.125 | 0.182 | 0.174 |
| 18-459 | 0.227 | 0.200 | 0.000 | 0.000 | 0.278 | 0.000 | 0.000 | 0.000 | 0.261 | 0.000 | 0.000 | 0.042 | 0.208 | 0.125 | 0.150 | 0.286 | 0.167 | 0.167 | 0.083 | 0.208 | 0.273 | 0.348 |
| 18-468 | 0.000 | 0.050 | 0.000 | 0.000 | 0.056 | 0.045 | 0.000 | 0.000 | 0.000 | 0.000 | 0.000 | 0.000 | 0.000 | 0.083 | 0.000 | 0.048 | 0.000 | 0.042 | 0.042 | 0.042 | 0.000 | 0.174 |
| 18-479 | 0.000 | 0.050 | 0.000 | 0.000 | 0.000 | 0.000 | 0.000 | 0.000 | 0.000 | 0.000 | 0.000 | 0.000 | 0.083 | 0.000 | 0.000 | 0.048 | 0.000 | 0.000 | 0.000 | 0.000 | 0.091 | 0.000 |
| 18-485 | 0.000 | 0.050 | 0.000 | 0.000 | 0.000 | 0.000 | 0.000 | 0.000 | 0.000 | 0.000 | 0.000 | 0.000 | 0.000 | 0.000 | 0.000 | 0.095 | 0.000 | 0.000 | 0.000 | 0.000 | 0.045 | 0.000 |
| 18-496 | 0.000 | 0.000 | 0.000 | 0.000 | 0.000 | 0.000 | 0.000 | 0.000 | 0.000 | 0.000 | 0.000 | 0.125 | 0.417 | 0.000 | 0.000 | 0.000 | 0.000 | 0.000 | 0.000 | 0.000 | 0.000 | 0.130 |
| 18-502 | 0.000 | 0.000 | 0.000 | 0.000 | 0.000 | 0.000 | 0.000 | 0.000 | 0.000 | 0.000 | 0.000 | 0.000 | 0.042 | 0.000 | 0.000 | 0.048 | 0.000 | 0.000 | 0.000 | 0.000 | 0.000 | 0.000 |
| 18-508 | 0.000 | 0.000 | 0.000 | 0.000 | 0.000 | 0.000 | 0.000 | 0.000 | 0.000 | 0.000 | 0.000 | 0.167 | 0.208 | 0.083 | 0.100 | 0.143 | 0.125 | 0.125 | 0.042 | 0.250 | 0.000 | 0.174 |
| 18-514 | 0.500 | 0.250 | 0.000 | 0.000 | 0.333 | 0.000 | 0.130 | 0.050 | 0.043 | 0.150 | 0.368 | 0.000 | 0.167 | 0.208 | 0.300 | 0.524 | 0.917 | 0.000 | 0.042 | 0.208 | 0.045 | 0.130 |
| 18-529 | 0.045 | 0.350 | 0.000 | 0.190 | 0.111 | 0.000 | 0.043 | 0.000 | 0.174 | 0.000 | 0.000 | 0.250 | 0.750 | 0.083 | 0.050 | 0.524 | 0.083 | 0.875 | 0.208 | 0.875 | 0.864 | 0.696 |
| 18-540 | 1.000 | 0.650 | 0.947 | 1.000 | 0.944 | 0.864 | 1.000 | 0.900 | 0.870 | 0.650 | 0.526 | 1.000 | 1.000 | 1.000 | 0.950 | 1.000 | 1.000 | 1.000 | 1.000 | 1.000 | 1.000 | 0.957 |
| 18-549 | 0.000 | 0.000 | 0.000 | 0.000 | 0.000 | 0.000 | 0.000 | 0.000 | 0.000 | 0.000 | 0.000 | 0.042 | 0.250 | 0.000 | 0.000 | 0.143 | 0.000 | 0.042 | 0.000 | 0.167 | 0.000 | 0.522 |
| 18-560 | 0.000 | 0.050 | 0.053 | 0.000 | 0.000 | 0.000 | 0.000 | 0.000 | 0.087 | 0.000 | 0.000 | 0.042 | 0.167 | 0.042 | 0.000 | 0.095 | 0.000 | 0.042 | 0.000 | 0.250 | 0.000 | 0.000 |
| 18-565 | 0.364 | 0.100 | 0.000 | 0.000 | 0.389 | 0.000 | 0.043 | 0.000 | 0.000 | 0.000 | 0.000 | 0.000 | 0.167 | 0.042 | 0.000 | 0.190 | 0.583 | 0.042 | 0.000 | 0.000 | 0.045 | 0.217 |
| 18-569 | 0.455 | 0.150 | 0.000 | 0.143 | 0.611 | 0.000 | 0.130 | 0.000 | 0.130 | 0.000 | 0.000 | 0.333 | 0.500 | 0.250 | 0.000 | 0.333 | 0.708 | 0.583 | 0.083 | 0.667 | 0.455 | 0.478 |
| 18-577 | 0.000 | 0.000 | 0.000 | 0.000 | 0.111 | 0.000 | 0.000 | 0.000 | 0.000 | 0.000 | 0.000 | 0.167 | 0.375 | 0.083 | 0.000 | 0.095 | 0.083 | 0.500 | 0.042 | 0.417 | 0.364 | 0.130 |
| 18-585 | 0.000 | 0.000 | 0.000 | 0.000 | 0.000 | 0.000 | 0.000 | 0.000 | 0.000 | 0.000 | 0.000 | 0.000 | 0.042 | 0.042 | 0.000 | 0.000 | 0.000 | 0.000 | 0.000 | 0.000 | 0.000 | 0.087 |
| 18-590 | 0.000 | 0.150 | 0.000 | 0.000 | 0.000 | 0.000 | 0.000 | 0.000 | 0.000 | 0.000 | 0.000 | 0.000 | 0.125 | 0.000 | 0.000 | 0.048 | 0.042 | 0.000 | 0.000 | 0.000 | 0.000 | 0.043 |
| 18-598 | 0.045 | 0.450 | 0.000 | 0.238 | 0.556 | 0.000 | 0.000 | 0.000 | 0.000 | 0.000 | 0.105 | 0.583 | 0.667 | 0.500 | 0.250 | 0.381 | 0.250 | 0.750 | 0.458 | 0.500 | 0.455 | 0.261 |
| 18-605 | 0.045 | 0.150 | 0.000 | 0.000 | 0.444 | 0.000 | 0.000 | 0.000 | 0.000 | 0.000 | 0.000 | 0.542 | 0.708 | 0.417 | 0.050 | 0.333 | 0.208 | 0.750 | 0.375 | 0.542 | 0.545 | 0.348 |
| 18-610 | 0.318 | 0.100 | 0.000 | 0.190 | 0.111 | 0.000 | 0.174 | 0.000 | 0.000 | 0.000 | 0.000 | 0.083 | 0.292 | 0.417 | 0.050 | 0.476 | 0.458 | 0.250 | 0.292 | 0.292 | 0.636 | 0.609 |
| 18-615 | 0.273 | 0.300 | 0.000 | 0.238 | 0.222 | 0.000 | 0.087 | 0.000 | 0.043 | 0.000 | 0.000 | 0.167 | 0.333 | 0.292 | 0.050 | 0.619 | 0.375 | 0.458 | 0.500 | 0.917 | 0.636 | 0.826 |
| 18-621 | 0.182 | 0.250 | 0.000 | 0.095 | 0.111 | 0.000 | 0.087 | 0.000 | 0.043 | 0.250 | 0.000 | 0.375 | 0.250 | 0.250 | 0.100 | 0.476 | 0.375 | 0.333 | 0.375 | 0.750 | 0.182 | 0.870 |
| 18-627 | 0.182 | 0.000 | 0.000 | 0.000 | 0.000 | 0.000 | 0.043 | 0.000 | 0.000 | 0.000 | 0.000 | 0.292 | 0.250 | 0.125 | 0.050 | 0.095 | 0.458 | 0.208 | 0.333 | 0.042 | 0.273 | 0.261 |
| 18-632 | 0.045 | 0.100 | 0.000 | 0.000 | 0.000 | 0.000 | 0.043 | 0.000 | 0.000 | 0.000 | 0.000 | 0.000 | 0.125 | 0.042 | 0.100 | 0.095 | 0.083 | 0.125 | 0.125 | 0.042 | 0.182 | 0.217 |
| 18-640 | 0.000 | 0.150 | 0.000 | 0.000 | 0.000 | 0.000 | 0.000 | 0.000 | 0.000 | 0.000 | 0.000 | 0.000 | 0.417 | 0.083 | 0.000 | 0.238 | 0.000 | 0.000 | 0.000 | 0.375 | 0.000 | 0.435 |
| 18-651 | 0.000 | 0.000 | 0.000 | 0.000 | 0.000 | 0.000 | 0.000 | 0.000 | 0.000 | 0.000 | 0.000 | 0.000 | 0.375 | 0.000 | 0.000 | 0.095 | 0.000 | 0.000 | 0.000 | 0.250 | 0.000 | 0.348 |
| 18-655 | 0.000 | 0.000 | 0.000 | 0.000 | 0.000 | 0.000 | 0.000 | 0.000 | 0.000 | 0.000 | 0.000 | 0.042 | 0.000 | 0.000 | 0.000 | 0.095 | 0.000 | 0.000 | 0.000 | 0.000 | 0.000 | 0.261 |
| 18-660 | 0.000 | 0.000 | 0.000 | 0.000 | 0.000 | 0.000 | 0.000 | 0.000 | 0.000 | 0.000 | 0.000 | 0.000 | 0.000 | 0.000 | 0.000 | 0.000 | 0.000 | 0.000 | 0.000 | 0.000 | 0.000 | 0.043 |
| 18-670 | 0.000 | 0.050 | 0.000 | 0.000 | 0.000 | 0.000 | 0.000 | 0.000 | 0.000 | 0.000 | 0.000 | 0.000 | 0.042 | 0.000 | 0.000 | 0.000 | 0.000 | 0.000 | 0.000 | 0.000 | 0.000 | 0.043 |
| 18-675 | 0.000 | 0.150 | 0.000 | 0.000 | 0.000 | 0.000 | 0.000 | 0.000 | 0.000 | 0.000 | 0.000 | 0.000 | 0.000 | 0.000 | 0.000 | 0.000 | 0.000 | 0.000 | 0.000 | 0.042 | 0.000 | 0.043 |
| 18-681 | 0.000 | 0.050 | 0.000 | 0.000 | 0.000 | 0.000 | 0.000 | 0.000 | 0.000 | 0.000 | 0.000 | 0.000 | 0.000 | 0.042 | 0.000 | 0.143 | 0.000 | 0.000 | 0.000 | 0.208 | 0.000 | 0.261 |
| 18-689 | 0.000 | 0.000 | 0.000 | 0.000 | 0.000 | 0.000 | 0.000 | 0.000 | 0.000 | 0.000 | 0.000 | 0.000 | 0.000 | 0.000 | 0.000 | 0.190 | 0.000 | 0.042 | 0.000 | 0.167 | 0.045 | 0.304 |
| 18-707 | 0.000 | 0.000 | 0.000 | 0.000 | 0.000 | 0.000 | 0.000 | 0.000 | 0.000 | 0.050 | 0.000 | 0.000 | 0.000 | 0.000 | 0.000 | 0.000 | 0.000 | 0.000 | 0.000 | 0.000 | 0.000 | 0.043 |
| 18-710 | 0.000 | 0.050 | 0.000 | 0.000 | 0.000 | 0.000 | 0.000 | 0.000 | 0.000 | 0.000 | 0.000 | 0.042 | 0.000 | 0.000 | 0.000 | 0.000 | 0.000 | 0.000 | 0.000 | 0.000 | 0.000 | 0.043 |
| 18-721 | 0.000 | 0.050 | 0.000 | 0.000 | 0.000 | 0.000 | 0.000 | 0.000 | 0.000 | 0.000 | 0.000 | 0.042 | 0.000 | 0.000 | 0.000 | 0.000 | 0.000 | 0.000 | 0.000 | 0.000 | 0.000 | 0.000 |
| 18-743 | 0.000 | 0.000 | 0.000 | 0.000 | 0.000 | 0.000 | 0.000 | 0.000 | 0.000 | 0.000 | 0.000 | 0.000 | 0.042 | 0.000 | 0.000 | 0.000 | 0.000 | 0.000 | 0.000 | 0.000 | 0.000 | 0.000 |
| 18-761 | 0.000 | 0.000 | 0.000 | 0.000 | 0.000 | 0.000 | 0.000 | 0.000 | 0.000 | 0.000 | 0.000 | 0.000 | 0.042 | 0.000 | 0.000 | 0.000 | 0.000 | 0.000 | 0.000 | 0.000 | 0.000 | 0.000 |
| 18-766 | 0.000 | 0.000 | 0.000 | 0.000 | 0.000 | 0.000 | 0.000 | 0.000 | 0.000 | 0.000 | 0.000 | 0.000 | 0.042 | 0.125 | 0.000 | 0.190 | 0.000 | 0.083 | 0.000 | 0.417 | 0.045 | 0.391 |
| 18-771 | 0.000 | 0.000 | 0.000 | 0.000 | 0.000 | 0.000 | 0.000 | 0.000 | 0.000 | 0.000 | 0.000 | 0.000 | 0.000 | 0.042 | 0.000 | 0.238 | 0.000 | 0.125 | 0.000 | 0.375 | 0.045 | 0.391 |
| 18-779 | 0.000 | 0.000 | 0.000 | 0.000 | 0.000 | 0.000 | 0.000 | 0.000 | 0.000 | 0.000 | 0.000 | 0.000 | 0.000 | 0.000 | 0.000 | 0.000 | 0.000 | 0.000 | 0.000 | 0.000 | 0.000 | 0.043 |
| 18-786 | 0.000 | 0.000 | 0.000 | 0.000 | 0.000 | 0.000 | 0.000 | 0.000 | 0.000 | 0.000 | 0.000 | 0.000 | 0.000 | 0.000 | 0.000 | 0.048 | 0.000 | 0.000 | 0.000 | 0.000 | 0.000 | 0.043 |
| 18-791 | 0.273 | 0.400 | 0.000 | 0.238 | 0.278 | 0.045 | 0.130 | 0.000 | 0.000 | 0.000 | 0.000 | 0.417 | 0.708 | 0.458 | 0.050 | 0.571 | 0.417 | 0.708 | 0.333 | 0.917 | 0.591 | 0.826 |
| 18-797 | 0.000 | 0.000 | 0.000 | 0.000 | 0.056 | 0.000 | 0.000 | 0.000 | 0.043 | 0.000 | 0.000 | 0.000 | 0.000 | 0.000 | 0.050 | 0.095 | 0.000 | 0.000 | 0.042 | 0.000 | 0.000 | 0.043 |
| 18-804 | 0.000 | 0.000 | 0.000 | 0.000 | 0.000 | 0.000 | 0.000 | 0.000 | 0.000 | 0.000 | 0.000 | 0.000 | 0.000 | 0.000 | 0.000 | 0.000 | 0.000 | 0.000 | 0.000 | 0.042 | 0.000 | 0.000 |
| 18-810 | 0.000 | 0.000 | 0.000 | 0.000 | 0.000 | 0.000 | 0.000 | 0.000 | 0.000 | 0.000 | 0.000 | 0.000 | 0.000 | 0.042 | 0.000 | 0.000 | 0.000 | 0.042 | 0.000 | 0.042 | 0.000 | 0.087 |
| 18-815 | 0.000 | 0.000 | 0.000 | 0.000 | 0.000 | 0.000 | 0.000 | 0.000 | 0.000 | 0.000 | 0.000 | 0.000 | 0.000 | 0.042 | 0.000 | 0.095 | 0.000 | 0.042 | 0.000 | 0.125 | 0.000 | 0.217 |
| 18-825 | 0.000 | 0.000 | 0.000 | 0.000 | 0.000 | 0.000 | 0.000 | 0.000 | 0.000 | 0.000 | 0.000 | 0.042 | 0.000 | 0.083 | 0.000 | 0.000 | 0.000 | 0.000 | 0.000 | 0.042 | 0.000 | 0.000 |
| 18-829 | 0.000 | 0.000 | 0.000 | 0.000 | 0.000 | 0.000 | 0.000 | 0.000 | 0.000 | 0.000 | 0.000 | 0.083 | 0.000 | 0.042 | 0.000 | 0.000 | 0.000 | 0.000 | 0.000 | 0.042 | 0.000 | 0.043 |
| 18-834 | 0.045 | 0.000 | 0.000 | 0.000 | 0.000 | 0.000 | 0.000 | 0.000 | 0.000 | 0.000 | 0.000 | 0.250 | 0.000 | 0.250 | 0.200 | 0.381 | 0.000 | 0.083 | 0.167 | 0.583 | 0.000 | 0.609 |
| 18-851 | 0.000 | 0.000 | 0.000 | 0.000 | 0.000 | 0.000 | 0.000 | 0.000 | 0.000 | 0.000 | 0.000 | 0.000 | 0.000 | 0.000 | 0.000 | 0.000 | 0.000 | 0.000 | 0.000 | 0.000 | 0.000 | 0.043 |
| 18-867 | 0.000 | 0.000 | 0.000 | 0.000 | 0.000 | 0.000 | 0.000 | 0.000 | 0.000 | 0.000 | 0.000 | 0.000 | 0.000 | 0.000 | 0.000 | 0.000 | 0.000 | 0.042 | 0.000 | 0.000 | 0.000 | 0.043 |
| 18-878 | 0.318 | 0.000 | 0.000 | 0.000 | 0.167 | 0.000 | 0.000 | 0.000 | 0.000 | 0.000 | 0.000 | 0.042 | 0.042 | 0.083 | 0.150 | 0.000 | 0.208 | 0.000 | 0.042 | 0.000 | 0.000 | 0.000 |
| 18-891 | 0.000 | 0.050 | 0.000 | 0.000 | 0.000 | 0.000 | 0.000 | 0.000 | 0.000 | 0.000 | 0.000 | 0.000 | 0.000 | 0.000 | 0.000 | 0.000 | 0.000 | 0.000 | 0.000 | 0.000 | 0.000 | 0.000 |
| 18-899 | 0.000 | 0.000 | 0.000 | 0.000 | 0.056 | 0.000 | 0.000 | 0.000 | 0.000 | 0.000 | 0.000 | 0.000 | 0.000 | 0.000 | 0.000 | 0.000 | 0.000 | 0.000 | 0.000 | 0.000 | 0.000 | 0.000 |
| 18-908 | 0.000 | 0.000 | 0.000 | 0.000 | 0.000 | 0.000 | 0.000 | 0.000 | 0.000 | 0.000 | 0.000 | 0.000 | 0.000 | 0.000 | 0.000 | 0.000 | 0.000 | 0.000 | 0.000 | 0.000 | 0.000 | 0.043 |
| 18-910 | 0.000 | 0.000 | 0.000 | 0.000 | 0.000 | 0.000 | 0.000 | 0.000 | 0.000 | 0.000 | 0.000 | 0.000 | 0.000 | 0.000 | 0.000 | 0.000 | 0.000 | 0.000 | 0.000 | 0.042 | 0.000 | 0.000 |
| 18-916 | 0.000 | 0.000 | 0.000 | 0.000 | 0.000 | 0.000 | 0.000 | 0.000 | 0.000 | 0.000 | 0.000 | 0.000 | 0.000 | 0.000 | 0.000 | 0.000 | 0.000 | 0.000 | 0.000 | 0.125 | 0.000 | 0.043 |
| 18-920 | 0.000 | 0.050 | 0.000 | 0.000 | 0.000 | 0.000 | 0.000 | 0.000 | 0.000 | 0.000 | 0.000 | 0.000 | 0.000 | 0.000 | 0.000 | 0.000 | 0.000 | 0.000 | 0.000 | 0.042 | 0.000 | 0.000 |
| 18-925 | 0.000 | 0.000 | 0.000 | 0.000 | 0.000 | 0.000 | 0.000 | 0.000 | 0.000 | 0.000 | 0.000 | 0.000 | 0.000 | 0.000 | 0.000 | 0.000 | 0.000 | 0.000 | 0.000 | 0.167 | 0.000 | 0.130 |
| 18-929 | 0.000 | 0.000 | 0.000 | 0.000 | 0.000 | 0.000 | 0.000 | 0.000 | 0.000 | 0.000 | 0.000 | 0.167 | 0.000 | 0.125 | 0.000 | 0.095 | 0.000 | 0.000 | 0.000 | 0.208 | 0.000 | 0.130 |
| 18-939 | 0.000 | 0.000 | 0.000 | 0.000 | 0.000 | 0.000 | 0.000 | 0.000 | 0.000 | 0.000 | 0.000 | 0.125 | 0.000 | 0.083 | 0.000 | 0.143 | 0.042 | 0.000 | 0.000 | 0.042 | 0.000 | 0.217 |
| 18-1076 | 0.000 | 0.050 | 0.000 | 0.000 | 0.000 | 0.000 | 0.000 | 0.000 | 0.000 | 0.000 | 0.000 | 0.000 | 0.000 | 0.000 | 0.000 | 0.000 | 0.000 | 0.000 | 0.000 | 0.000 | 0.000 | 0.000 |
| 18-1081 | 0.000 | 0.050 | 0.000 | 0.000 | 0.000 | 0.000 | 0.000 | 0.000 | 0.000 | 0.000 | 0.000 | 0.000 | 0.000 | 0.000 | 0.000 | 0.000 | 0.000 | 0.000 | 0.000 | 0.000 | 0.000 | 0.000 |
| 25-105 | 0.000 | 0.042 | 0.478 | 0.000 | 0.000 | 0.167 | 0.000 | 0.050 | 0.125 | 0.091 | 0.478 | 0.000 | 0.083 | 0.048 | 0.053 | 0.048 | 0.000 | 0.000 | 0.045 | 0.000 | 0.000 | 0.174 |
| 25-111 | 0.000 | 0.042 | 0.000 | 0.000 | 0.000 | 0.000 | 0.000 | 0.000 | 0.000 | 0.000 | 0.000 | 0.000 | 0.042 | 0.000 | 0.053 | 0.000 | 0.000 | 0.000 | 0.000 | 0.000 | 0.053 | 0.087 |
| 25-117 | 0.091 | 0.083 | 0.304 | 0.042 | 0.050 | 0.042 | 0.000 | 0.000 | 0.042 | 0.000 | 0.174 | 0.000 | 0.042 | 0.000 | 0.000 | 0.000 | 0.000 | 0.000 | 0.045 | 0.000 | 0.000 | 0.043 |
| 25-121 | 0.000 | 0.000 | 0.043 | 0.292 | 0.000 | 0.000 | 0.000 | 0.000 | 0.000 | 0.045 | 0.000 | 0.087 | 0.000 | 0.048 | 0.000 | 0.000 | 0.000 | 0.000 | 0.000 | 0.000 | 0.000 | 0.087 |
| 25-127 | 0.591 | 0.042 | 0.652 | 0.292 | 0.300 | 0.250 | 0.348 | 0.050 | 0.333 | 0.500 | 0.565 | 0.043 | 0.000 | 0.000 | 0.000 | 0.333 | 0.042 | 0.043 | 0.591 | 0.083 | 0.105 | 0.174 |
| 25-134 | 0.727 | 0.000 | 0.435 | 0.042 | 0.350 | 0.333 | 0.000 | 0.050 | 0.083 | 0.045 | 0.565 | 0.043 | 0.000 | 0.000 | 0.053 | 0.143 | 0.083 | 0.043 | 0.591 | 0.042 | 0.105 | 0.261 |
| 25-141 | 0.000 | 0.000 | 0.087 | 0.000 | 0.000 | 0.000 | 0.174 | 0.000 | 0.000 | 0.000 | 0.000 | 0.000 | 0.083 | 0.143 | 0.053 | 0.000 | 0.000 | 0.000 | 0.045 | 0.000 | 0.000 | 0.000 |
| 25-147 | 0.000 | 0.000 | 0.000 | 0.000 | 0.000 | 0.000 | 0.000 | 0.000 | 0.000 | 0.000 | 0.043 | 0.000 | 0.000 | 0.000 | 0.000 | 0.000 | 0.000 | 0.000 | 0.000 | 0.000 | 0.000 | 0.087 |
| 25-151 | 0.864 | 0.667 | 1.000 | 0.875 | 0.600 | 0.708 | 0.739 | 0.950 | 0.542 | 0.864 | 0.870 | 0.696 | 0.083 | 0.476 | 0.579 | 0.952 | 0.417 | 0.435 | 0.591 | 0.875 | 0.000 | 0.826 |
| 25-156 | 0.000 | 0.000 | 0.043 | 0.042 | 0.150 | 0.000 | 0.000 | 0.000 | 0.000 | 0.000 | 0.000 | 0.043 | 0.042 | 0.095 | 0.000 | 0.000 | 0.000 | 0.261 | 0.136 | 0.000 | 0.053 | 0.000 |
| 25-163 | 0.955 | 0.292 | 0.826 | 0.292 | 0.600 | 0.708 | 0.261 | 0.350 | 0.250 | 0.591 | 0.739 | 0.261 | 0.083 | 0.048 | 0.263 | 0.381 | 0.125 | 0.087 | 0.500 | 0.292 | 0.053 | 0.261 |
| 25-168 | 0.045 | 0.167 | 0.130 | 0.000 | 0.100 | 0.042 | 0.000 | 0.050 | 0.167 | 0.091 | 0.043 | 0.087 | 0.042 | 0.000 | 0.000 | 0.190 | 0.042 | 0.478 | 0.227 | 0.083 | 0.053 | 0.043 |
| 25-174 | 0.045 | 0.000 | 0.043 | 0.000 | 0.100 | 0.000 | 0.000 | 0.000 | 0.000 | 0.000 | 0.000 | 0.000 | 0.000 | 0.048 | 0.000 | 0.000 | 0.000 | 0.043 | 0.000 | 0.000 | 0.000 | 0.000 |
| 25-180 | 0.955 | 0.333 | 0.826 | 0.750 | 0.600 | 0.333 | 0.696 | 0.750 | 0.583 | 0.727 | 1.000 | 0.261 | 0.292 | 0.190 | 0.263 | 0.762 | 0.625 | 0.565 | 0.773 | 0.833 | 0.421 | 0.609 |
| 25-184 | 0.000 | 0.000 | 0.174 | 0.042 | 0.000 | 0.000 | 0.043 | 0.000 | 0.000 | 0.045 | 0.000 | 0.043 | 0.000 | 0.000 | 0.000 | 0.000 | 0.000 | 0.000 | 0.000 | 0.000 | 0.000 | 0.000 |
| 25-188 | 0.227 | 0.000 | 0.000 | 0.083 | 0.300 | 0.000 | 0.043 | 0.000 | 0.000 | 0.000 | 0.000 | 0.000 | 0.000 | 0.000 | 0.000 | 0.000 | 0.042 | 0.000 | 0.045 | 0.000 | 0.000 | 0.000 |
| 25-193 | 0.227 | 0.000 | 0.435 | 0.125 | 0.050 | 0.000 | 0.000 | 0.000 | 0.000 | 0.000 | 0.087 | 0.043 | 0.000 | 0.048 | 0.105 | 0.000 | 0.000 | 0.043 | 0.045 | 0.000 | 0.000 | 0.000 |
| 25-198 | 0.409 | 0.000 | 0.000 | 0.083 | 0.150 | 0.000 | 0.087 | 0.000 | 0.000 | 0.000 | 0.000 | 0.000 | 0.000 | 0.000 | 0.000 | 0.000 | 0.000 | 0.043 | 0.091 | 0.000 | 0.105 | 0.000 |
| 25-202 | 0.273 | 0.000 | 0.000 | 0.083 | 0.100 | 0.000 | 0.000 | 0.000 | 0.000 | 0.000 | 0.043 | 0.000 | 0.000 | 0.000 | 0.000 | 0.000 | 0.000 | 0.043 | 0.045 | 0.000 | 0.105 | 0.000 |
| 25-206 | 0.591 | 0.000 | 0.087 | 0.000 | 0.150 | 0.000 | 0.000 | 0.000 | 0.000 | 0.000 | 0.000 | 0.000 | 0.000 | 0.000 | 0.000 | 0.095 | 0.083 | 0.000 | 0.136 | 0.000 | 0.000 | 0.043 |
| 25-211 | 0.364 | 0.000 | 0.609 | 0.083 | 0.200 | 0.000 | 0.043 | 0.000 | 0.000 | 0.000 | 0.000 | 0.217 | 0.083 | 0.000 | 0.000 | 0.000 | 0.000 | 0.000 | 0.045 | 0.000 | 0.000 | 0.043 |
| 25-214 | 0.273 | 0.000 | 0.130 | 0.000 | 0.150 | 0.000 | 0.087 | 0.000 | 0.000 | 0.000 | 0.043 | 0.000 | 0.000 | 0.000 | 0.053 | 0.000 | 0.083 | 0.043 | 0.045 | 0.000 | 0.000 | 0.000 |
| 25-218 | 0.409 | 0.000 | 0.043 | 0.000 | 0.250 | 0.208 | 0.000 | 0.100 | 0.000 | 0.000 | 0.000 | 0.087 | 0.000 | 0.000 | 0.000 | 0.000 | 0.000 | 0.130 | 0.045 | 0.000 | 0.000 | 0.000 |
| 25-223 | 0.909 | 0.042 | 0.826 | 0.125 | 0.600 | 0.250 | 0.130 | 0.050 | 0.125 | 0.045 | 0.826 | 0.043 | 0.042 | 0.000 | 0.000 | 0.286 | 0.167 | 0.000 | 0.091 | 0.125 | 0.105 | 0.130 |
| 25-227 | 0.727 | 0.042 | 0.348 | 0.000 | 0.500 | 0.167 | 0.043 | 0.550 | 0.167 | 0.045 | 0.130 | 0.087 | 0.000 | 0.048 | 0.053 | 0.238 | 0.125 | 0.348 | 0.591 | 0.000 | 0.053 | 0.130 |
| 25-233 | 0.182 | 0.000 | 0.087 | 0.042 | 0.100 | 0.000 | 0.043 | 0.050 | 0.000 | 0.045 | 0.000 | 0.000 | 0.000 | 0.000 | 0.000 | 0.000 | 0.042 | 0.000 | 0.000 | 0.000 | 0.000 | 0.000 |
| 25-236 | 0.409 | 0.000 | 0.087 | 0.167 | 0.250 | 0.042 | 0.391 | 0.150 | 0.083 | 0.136 | 0.130 | 0.000 | 0.042 | 0.000 | 0.053 | 0.095 | 0.042 | 0.435 | 0.409 | 0.042 | 0.000 | 0.000 |
| 25-240 | 0.864 | 0.458 | 1.000 | 0.583 | 0.650 | 0.958 | 0.565 | 0.950 | 0.667 | 0.727 | 0.913 | 0.391 | 0.292 | 0.333 | 0.368 | 0.905 | 0.708 | 0.783 | 0.773 | 0.750 | 0.316 | 0.522 |
| 25-245 | 0.727 | 0.292 | 0.783 | 0.292 | 0.600 | 0.375 | 0.435 | 0.850 | 0.500 | 0.500 | 0.522 | 0.130 | 0.000 | 0.190 | 0.053 | 0.667 | 0.333 | 0.435 | 0.409 | 0.583 | 0.211 | 0.565 |
| 25-253 | 0.909 | 0.125 | 0.609 | 0.208 | 0.650 | 0.250 | 0.304 | 0.100 | 0.167 | 0.227 | 0.739 | 0.000 | 0.000 | 0.095 | 0.000 | 0.190 | 0.125 | 0.130 | 0.318 | 0.125 | 0.000 | 0.087 |
| 25-259 | 0.545 | 0.042 | 0.435 | 0.000 | 0.350 | 0.208 | 0.043 | 0.050 | 0.042 | 0.000 | 0.478 | 0.043 | 0.000 | 0.095 | 0.053 | 0.048 | 0.125 | 0.261 | 0.136 | 0.042 | 0.053 | 0.043 |
| 25-266 | 0.864 | 0.000 | 0.522 | 0.208 | 0.450 | 0.042 | 0.217 | 0.000 | 0.167 | 0.318 | 0.478 | 0.000 | 0.042 | 0.048 | 0.000 | 0.095 | 0.167 | 0.261 | 0.636 | 0.042 | 0.211 | 0.087 |
| 25-271 | 0.727 | 0.000 | 0.391 | 0.000 | 0.400 | 0.083 | 0.261 | 0.050 | 0.042 | 0.045 | 0.174 | 0.087 | 0.125 | 0.000 | 0.000 | 0.048 | 0.125 | 0.348 | 0.182 | 0.000 | 0.000 | 0.000 |
| 25-278 | 0.364 | 0.000 | 0.000 | 0.000 | 0.100 | 0.000 | 0.000 | 0.000 | 0.042 | 0.000 | 0.043 | 0.000 | 0.042 | 0.000 | 0.000 | 0.000 | 0.083 | 0.000 | 0.000 | 0.000 | 0.000 | 0.000 |
| 25-283 | 0.318 | 0.250 | 0.739 | 0.542 | 0.300 | 0.667 | 0.087 | 0.700 | 0.833 | 0.864 | 0.652 | 0.522 | 0.542 | 0.619 | 0.632 | 0.238 | 0.708 | 0.565 | 0.955 | 0.708 | 0.842 | 0.826 |
| 25-287 | 0.636 | 0.542 | 0.348 | 0.625 | 0.400 | 0.167 | 0.609 | 0.550 | 0.458 | 0.545 | 0.478 | 0.304 | 0.000 | 0.048 | 0.000 | 0.476 | 0.292 | 0.870 | 0.545 | 0.542 | 0.421 | 0.348 |
| 25-293 | 0.409 | 0.000 | 0.304 | 0.000 | 0.300 | 0.083 | 0.087 | 0.100 | 0.042 | 0.000 | 0.000 | 0.043 | 0.042 | 0.000 | 0.000 | 0.000 | 0.042 | 0.043 | 0.182 | 0.000 | 0.000 | 0.000 |
| 25-297 | 0.318 | 0.000 | 0.000 | 0.292 | 0.100 | 0.000 | 0.000 | 0.350 | 0.125 | 0.136 | 0.087 | 0.261 | 0.000 | 0.095 | 0.000 | 0.000 | 0.000 | 0.043 | 0.091 | 0.000 | 0.263 | 0.000 |
| 25-304 | 0.273 | 0.000 | 0.087 | 0.083 | 0.250 | 0.000 | 0.043 | 0.000 | 0.000 | 0.091 | 0.000 | 0.043 | 0.042 | 0.095 | 0.000 | 0.190 | 0.083 | 0.304 | 0.000 | 0.167 | 0.053 | 0.000 |
| 25-308 | 0.682 | 0.000 | 0.348 | 0.042 | 0.300 | 0.000 | 0.043 | 0.000 | 0.042 | 0.045 | 0.130 | 0.043 | 0.000 | 0.000 | 0.000 | 0.000 | 0.000 | 0.130 | 0.318 | 0.042 | 0.263 | 0.000 |
| 25-318 | 0.545 | 0.000 | 0.478 | 0.125 | 0.350 | 0.083 | 0.174 | 0.100 | 0.000 | 0.045 | 0.000 | 0.087 | 0.208 | 0.000 | 0.000 | 0.048 | 0.042 | 0.304 | 0.273 | 0.000 | 0.053 | 0.130 |
| 25-322 | 0.455 | 0.000 | 0.087 | 0.000 | 0.300 | 0.000 | 0.043 | 0.000 | 0.000 | 0.000 | 0.000 | 0.043 | 0.000 | 0.000 | 0.000 | 0.000 | 0.083 | 0.043 | 0.000 | 0.000 | 0.000 | 0.000 |
| 25-327 | 0.545 | 0.000 | 0.391 | 0.000 | 0.500 | 0.167 | 0.000 | 0.550 | 0.250 | 0.000 | 0.174 | 0.000 | 0.083 | 0.000 | 0.000 | 0.048 | 0.083 | 0.261 | 0.500 | 0.000 | 0.316 | 0.000 |
| 25-337 | 0.864 | 0.042 | 0.870 | 0.333 | 0.500 | 0.125 | 0.304 | 0.000 | 0.167 | 0.500 | 0.957 | 0.000 | 0.042 | 0.000 | 0.053 | 0.095 | 0.208 | 0.348 | 0.091 | 0.125 | 0.105 | 0.130 |
| 25-341 | 0.364 | 0.083 | 0.130 | 0.042 | 0.250 | 0.000 | 0.043 | 0.050 | 0.042 | 0.000 | 0.000 | 0.000 | 0.000 | 0.000 | 0.000 | 0.048 | 0.083 | 0.000 | 0.045 | 0.000 | 0.053 | 0.000 |
| 25-352 | 1.000 | 0.958 | 0.957 | 1.000 | 0.950 | 0.792 | 0.957 | 0.950 | 1.000 | 1.000 | 0.913 | 0.913 | 0.917 | 0.667 | 0.895 | 1.000 | 1.000 | 1.000 | 1.000 | 0.958 | 0.895 | 1.000 |
| 25-357 | 0.091 | 0.000 | 0.435 | 0.042 | 0.150 | 0.292 | 0.130 | 0.050 | 0.000 | 0.091 | 0.391 | 0.000 | 0.083 | 0.048 | 0.053 | 0.095 | 0.083 | 0.000 | 0.000 | 0.000 | 0.000 | 0.000 |
| 25-364 | 0.773 | 0.000 | 0.783 | 0.333 | 0.450 | 0.208 | 0.478 | 0.300 | 0.208 | 0.182 | 0.826 | 0.174 | 0.000 | 0.000 | 0.000 | 0.048 | 0.125 | 0.043 | 0.273 | 0.000 | 0.105 | 0.087 |
| 25-371 | 0.864 | 0.792 | 0.609 | 0.208 | 0.400 | 0.000 | 0.348 | 0.000 | 0.000 | 0.000 | 0.826 | 0.000 | 0.000 | 0.000 | 0.000 | 0.143 | 0.083 | 0.043 | 0.045 | 0.083 | 0.105 | 0.043 |
| 25-383 | 0.818 | 0.042 | 0.739 | 0.167 | 0.600 | 0.208 | 0.130 | 0.100 | 0.250 | 0.364 | 0.783 | 0.043 | 0.042 | 0.143 | 0.000 | 0.048 | 0.125 | 0.304 | 0.682 | 0.083 | 0.105 | 0.043 |
| 25-391 | 0.955 | 0.208 | 0.913 | 0.750 | 0.750 | 0.667 | 0.696 | 0.450 | 0.167 | 0.455 | 0.957 | 0.348 | 0.292 | 0.048 | 0.263 | 0.190 | 0.500 | 0.000 | 0.182 | 0.000 | 0.000 | 0.217 |
| 25-397 | 0.591 | 0.000 | 0.217 | 0.000 | 0.200 | 0.125 | 0.043 | 0.000 | 0.000 | 0.000 | 0.217 | 0.000 | 0.000 | 0.000 | 0.000 | 0.048 | 0.125 | 0.043 | 0.091 | 0.000 | 0.000 | 0.000 |
| 25-405 | 0.364 | 0.042 | 0.217 | 0.000 | 0.150 | 0.000 | 0.043 | 0.000 | 0.000 | 0.000 | 0.000 | 0.043 | 0.000 | 0.190 | 0.053 | 0.000 | 0.042 | 0.043 | 0.045 | 0.042 | 0.000 | 0.000 |
| 25-410 | 0.545 | 0.000 | 0.522 | 0.125 | 0.450 | 0.167 | 0.130 | 0.000 | 0.083 | 0.045 | 0.565 | 0.000 | 0.000 | 0.000 | 0.000 | 0.000 | 0.125 | 0.304 | 0.045 | 0.000 | 0.316 | 0.000 |
| 25-422 | 1.000 | 0.792 | 1.000 | 1.000 | 0.950 | 1.000 | 0.913 | 1.000 | 0.875 | 1.000 | 1.000 | 0.913 | 0.833 | 0.619 | 0.895 | 0.952 | 1.000 | 0.783 | 1.000 | 0.917 | 0.474 | 0.826 |
| 25-435 | 0.591 | 0.000 | 0.522 | 0.000 | 0.150 | 0.000 | 0.130 | 0.000 | 0.000 | 0.000 | 0.043 | 0.043 | 0.042 | 0.048 | 0.158 | 0.000 | 0.083 | 0.043 | 0.045 | 0.083 | 0.053 | 0.000 |
| 25-444 | 0.045 | 0.000 | 0.043 | 0.000 | 0.100 | 0.000 | 0.043 | 0.000 | 0.000 | 0.000 | 0.000 | 0.000 | 0.000 | 0.000 | 0.000 | 0.000 | 0.000 | 0.000 | 0.045 | 0.000 | 0.105 | 0.043 |
| 25-448 | 0.045 | 0.000 | 0.043 | 0.042 | 0.050 | 0.000 | 0.087 | 0.000 | 0.083 | 0.000 | 0.000 | 0.000 | 0.083 | 0.000 | 0.000 | 0.000 | 0.000 | 0.000 | 0.091 | 0.000 | 0.105 | 0.043 |
| 25-457 | 0.455 | 0.292 | 0.609 | 0.042 | 0.450 | 0.500 | 0.087 | 0.700 | 0.583 | 0.364 | 0.696 | 0.043 | 0.000 | 0.048 | 0.053 | 0.238 | 0.000 | 0.913 | 0.818 | 0.417 | 0.632 | 0.087 |
| 25-461 | 0.955 | 0.583 | 0.913 | 0.583 | 0.700 | 0.833 | 0.696 | 0.950 | 0.792 | 0.773 | 1.000 | 0.652 | 0.333 | 0.238 | 0.632 | 0.714 | 0.708 | 0.913 | 0.955 | 0.667 | 0.684 | 0.522 |
| 25-469 | 0.636 | 0.125 | 0.870 | 0.167 | 0.300 | 0.375 | 0.174 | 0.350 | 0.458 | 0.136 | 0.913 | 0.000 | 0.000 | 0.000 | 0.000 | 0.048 | 0.083 | 0.043 | 0.136 | 0.042 | 0.000 | 0.217 |
| 25-473 | 0.682 | 0.250 | 1.000 | 0.417 | 0.550 | 0.833 | 0.304 | 0.750 | 0.583 | 0.682 | 0.913 | 0.174 | 0.167 | 0.095 | 0.211 | 0.190 | 0.167 | 0.217 | 0.682 | 0.083 | 0.316 | 0.391 |
| 25-480 | 0.045 | 0.000 | 0.304 | 0.042 | 0.050 | 0.042 | 0.087 | 0.000 | 0.000 | 0.000 | 0.000 | 0.000 | 0.000 | 0.000 | 0.000 | 0.000 | 0.000 | 0.130 | 0.045 | 0.000 | 0.105 | 0.000 |
| 25-487 | 0.045 | 0.042 | 0.435 | 0.000 | 0.050 | 0.042 | 0.217 | 0.000 | 0.000 | 0.000 | 0.000 | 0.043 | 0.083 | 0.000 | 0.000 | 0.000 | 0.000 | 0.043 | 0.000 | 0.000 | 0.053 | 0.000 |
| 25-492 | 0.045 | 0.083 | 0.130 | 0.083 | 0.100 | 0.083 | 0.435 | 0.000 | 0.083 | 0.000 | 0.043 | 0.000 | 0.042 | 0.000 | 0.000 | 0.000 | 0.000 | 0.174 | 0.045 | 0.000 | 0.000 | 0.000 |
| 25-506 | 0.591 | 0.000 | 0.696 | 0.167 | 0.350 | 0.125 | 0.174 | 0.000 | 0.167 | 0.091 | 0.696 | 0.000 | 0.000 | 0.000 | 0.000 | 0.000 | 0.083 | 0.087 | 0.409 | 0.000 | 0.105 | 0.000 |
| 25-511 | 0.045 | 0.000 | 0.000 | 0.000 | 0.000 | 0.000 | 0.043 | 0.000 | 0.000 | 0.000 | 0.000 | 0.000 | 0.000 | 0.000 | 0.000 | 0.000 | 0.000 | 0.000 | 0.000 | 0.000 | 0.000 | 0.000 |
| 25-518 | 0.045 | 0.000 | 0.435 | 0.000 | 0.050 | 0.083 | 0.000 | 0.050 | 0.250 | 0.000 | 0.000 | 0.000 | 0.000 | 0.000 | 0.000 | 0.000 | 0.000 | 0.217 | 0.545 | 0.000 | 0.263 | 0.000 |
| 25-522 | 0.000 | 0.000 | 0.130 | 0.042 | 0.050 | 0.042 | 0.043 | 0.050 | 0.125 | 0.000 | 0.000 | 0.000 | 0.000 | 0.048 | 0.000 | 0.000 | 0.000 | 0.217 | 0.136 | 0.000 | 0.158 | 0.087 |
| 25-526 | 0.045 | 0.000 | 0.174 | 0.000 | 0.150 | 0.000 | 0.043 | 0.000 | 0.000 | 0.000 | 0.043 | 0.000 | 0.042 | 0.000 | 0.000 | 0.000 | 0.000 | 0.000 | 0.045 | 0.000 | 0.000 | 0.000 |
| 25-531 | 0.273 | 0.000 | 0.304 | 0.333 | 0.150 | 0.000 | 0.348 | 0.000 | 0.125 | 0.000 | 0.348 | 0.000 | 0.042 | 0.000 | 0.000 | 0.000 | 0.083 | 0.000 | 0.000 | 0.000 | 0.000 | 0.000 |
| 25-535 | 0.318 | 0.000 | 0.391 | 0.125 | 0.200 | 0.208 | 0.391 | 0.000 | 0.125 | 0.136 | 0.391 | 0.043 | 0.000 | 0.000 | 0.000 | 0.000 | 0.042 | 0.000 | 0.182 | 0.000 | 0.053 | 0.000 |
| 25-544 | 0.091 | 0.042 | 0.565 | 0.375 | 0.050 | 0.083 | 0.739 | 0.000 | 0.042 | 0.000 | 0.609 | 0.000 | 0.042 | 0.000 | 0.000 | 0.000 | 0.000 | 0.000 | 0.000 | 0.000 | 0.000 | 0.000 |
| 25-551 | 0.682 | 0.625 | 0.565 | 0.875 | 0.650 | 0.875 | 0.826 | 0.750 | 0.792 | 0.773 | 0.652 | 0.522 | 0.625 | 0.381 | 0.579 | 0.857 | 0.875 | 0.435 | 0.864 | 0.708 | 0.263 | 0.522 |
| 25-553 | 0.227 | 0.125 | 0.609 | 0.042 | 0.100 | 0.042 | 0.043 | 0.150 | 0.167 | 0.227 | 0.348 | 0.000 | 0.000 | 0.000 | 0.000 | 0.095 | 0.000 | 0.087 | 0.091 | 0.125 | 0.211 | 0.000 |
| 25-560 | 0.000 | 0.000 | 0.130 | 0.125 | 0.050 | 0.000 | 0.000 | 0.050 | 0.083 | 0.091 | 0.304 | 0.000 | 0.000 | 0.000 | 0.000 | 0.000 | 0.000 | 0.087 | 0.045 | 0.000 | 0.000 | 0.000 |
| 25-563 | 0.091 | 0.083 | 0.522 | 0.500 | 0.050 | 0.208 | 0.435 | 0.450 | 0.083 | 0.273 | 0.348 | 0.043 | 0.000 | 0.048 | 0.000 | 0.048 | 0.208 | 0.435 | 0.273 | 0.125 | 0.158 | 0.043 |
| 25-570 | 0.000 | 0.083 | 0.435 | 0.500 | 0.050 | 0.167 | 0.435 | 0.350 | 0.000 | 0.227 | 0.043 | 0.000 | 0.000 | 0.048 | 0.000 | 0.048 | 0.000 | 0.304 | 0.136 | 0.083 | 0.158 | 0.043 |
| 25-574 | 0.000 | 0.000 | 0.087 | 0.000 | 0.000 | 0.000 | 0.087 | 0.000 | 0.000 | 0.000 | 0.000 | 0.000 | 0.000 | 0.000 | 0.000 | 0.000 | 0.000 | 0.000 | 0.000 | 0.000 | 0.000 | 0.000 |
| 25-580 | 0.045 | 0.000 | 0.000 | 0.000 | 0.100 | 0.000 | 0.043 | 0.000 | 0.000 | 0.000 | 0.043 | 0.000 | 0.000 | 0.000 | 0.000 | 0.000 | 0.000 | 0.000 | 0.000 | 0.000 | 0.053 | 0.000 |
| 25-587 | 0.000 | 0.000 | 0.000 | 0.000 | 0.050 | 0.000 | 0.087 | 0.000 | 0.000 | 0.000 | 0.087 | 0.000 | 0.000 | 0.000 | 0.000 | 0.000 | 0.000 | 0.000 | 0.000 | 0.000 | 0.000 | 0.000 |
| 25-594 | 0.000 | 0.042 | 0.000 | 0.000 | 0.000 | 0.000 | 0.043 | 0.000 | 0.000 | 0.000 | 0.043 | 0.000 | 0.000 | 0.000 | 0.000 | 0.000 | 0.000 | 0.000 | 0.000 | 0.000 | 0.000 | 0.000 |
| 25-600 | 0.000 | 0.042 | 0.043 | 0.000 | 0.000 | 0.000 | 0.087 | 0.000 | 0.000 | 0.000 | 0.043 | 0.000 | 0.000 | 0.048 | 0.000 | 0.000 | 0.000 | 0.000 | 0.000 | 0.000 | 0.000 | 0.000 |
| 25-604 | 0.682 | 0.167 | 0.783 | 0.750 | 0.200 | 0.625 | 0.652 | 0.550 | 0.167 | 0.682 | 0.957 | 0.087 | 0.125 | 0.000 | 0.000 | 0.048 | 0.167 | 0.043 | 0.000 | 0.042 | 0.000 | 0.087 |
| 25-611 | 1.000 | 0.375 | 0.957 | 0.917 | 0.850 | 0.750 | 0.870 | 0.650 | 0.208 | 0.682 | 1.000 | 0.435 | 0.750 | 0.333 | 0.421 | 0.476 | 0.917 | 0.000 | 0.091 | 0.208 | 0.105 | 0.478 |
| 25-620 | 0.091 | 0.000 | 0.478 | 0.000 | 0.050 | 0.042 | 0.000 | 0.000 | 0.083 | 0.091 | 0.348 | 0.000 | 0.000 | 0.000 | 0.000 | 0.000 | 0.042 | 0.043 | 0.000 | 0.000 | 0.000 | 0.000 |
| 25-633 | 0.000 | 0.000 | 0.000 | 0.042 | 0.000 | 0.000 | 0.000 | 0.000 | 0.000 | 0.000 | 0.000 | 0.000 | 0.042 | 0.000 | 0.000 | 0.000 | 0.000 | 0.043 | 0.000 | 0.000 | 0.053 | 0.000 |
| 25-640 | 0.000 | 0.000 | 0.043 | 0.000 | 0.000 | 0.000 | 0.043 | 0.000 | 0.000 | 0.000 | 0.000 | 0.000 | 0.000 | 0.000 | 0.000 | 0.000 | 0.000 | 0.000 | 0.000 | 0.000 | 0.000 | 0.000 |
| 25-646 | 0.000 | 0.000 | 0.000 | 0.083 | 0.000 | 0.000 | 0.087 | 0.050 | 0.042 | 0.000 | 0.000 | 0.000 | 0.000 | 0.000 | 0.000 | 0.000 | 0.000 | 0.000 | 0.000 | 0.000 | 0.158 | 0.000 |
| 25-651 | 0.000 | 0.000 | 0.000 | 0.000 | 0.000 | 0.000 | 0.043 | 0.000 | 0.042 | 0.000 | 0.000 | 0.000 | 0.042 | 0.000 | 0.000 | 0.000 | 0.000 | 0.000 | 0.000 | 0.000 | 0.105 | 0.000 |
| 25-655 | 0.000 | 0.000 | 0.043 | 0.000 | 0.000 | 0.000 | 0.087 | 0.000 | 0.000 | 0.000 | 0.000 | 0.000 | 0.125 | 0.000 | 0.000 | 0.000 | 0.000 | 0.130 | 0.000 | 0.000 | 0.211 | 0.000 |
| 25-658 | 0.000 | 0.000 | 0.000 | 0.000 | 0.050 | 0.000 | 0.000 | 0.000 | 0.000 | 0.000 | 0.043 | 0.000 | 0.000 | 0.000 | 0.000 | 0.000 | 0.000 | 0.043 | 0.000 | 0.000 | 0.000 | 0.000 |
| 25-669 | 0.000 | 0.042 | 0.000 | 0.000 | 0.000 | 0.000 | 0.043 | 0.000 | 0.000 | 0.000 | 0.000 | 0.000 | 0.000 | 0.000 | 0.000 | 0.000 | 0.000 | 0.000 | 0.000 | 0.000 | 0.000 | 0.000 |
| 25-674 | 0.227 | 0.708 | 0.609 | 0.625 | 0.450 | 0.542 | 0.826 | 0.700 | 0.750 | 0.409 | 0.130 | 0.435 | 0.625 | 0.333 | 0.316 | 0.714 | 0.125 | 0.391 | 0.591 | 0.167 | 0.684 | 0.478 |
| 25-680 | 0.182 | 0.667 | 0.609 | 0.625 | 0.400 | 0.417 | 0.783 | 0.700 | 0.750 | 0.409 | 0.130 | 0.304 | 0.625 | 0.333 | 0.263 | 0.667 | 0.083 | 0.391 | 0.591 | 0.083 | 0.684 | 0.391 |
| 25-684 | 0.000 | 0.000 | 0.348 | 0.042 | 0.000 | 0.000 | 0.130 | 0.000 | 0.000 | 0.000 | 0.217 | 0.000 | 0.000 | 0.048 | 0.000 | 0.000 | 0.000 | 0.217 | 0.000 | 0.000 | 0.000 | 0.000 |
| 25-688 | 0.000 | 0.000 | 0.348 | 0.000 | 0.000 | 0.000 | 0.043 | 0.000 | 0.000 | 0.000 | 0.043 | 0.000 | 0.042 | 0.000 | 0.000 | 0.000 | 0.000 | 0.000 | 0.000 | 0.000 | 0.000 | 0.000 |
| 25-692 | 0.045 | 0.000 | 0.348 | 0.083 | 0.000 | 0.042 | 0.043 | 0.000 | 0.000 | 0.000 | 0.043 | 0.000 | 0.042 | 0.000 | 0.000 | 0.000 | 0.000 | 0.087 | 0.000 | 0.000 | 0.053 | 0.000 |
| 25-699 | 0.000 | 0.000 | 0.000 | 0.000 | 0.000 | 0.000 | 0.000 | 0.000 | 0.000 | 0.000 | 0.000 | 0.000 | 0.000 | 0.000 | 0.000 | 0.000 | 0.000 | 0.043 | 0.000 | 0.000 | 0.105 | 0.000 |
| 25-705 | 0.000 | 0.000 | 0.000 | 0.000 | 0.000 | 0.000 | 0.000 | 0.000 | 0.000 | 0.000 | 0.000 | 0.000 | 0.000 | 0.000 | 0.000 | 0.000 | 0.000 | 0.000 | 0.000 | 0.000 | 0.053 | 0.000 |
| 25-712 | 0.000 | 0.000 | 0.000 | 0.000 | 0.000 | 0.000 | 0.087 | 0.000 | 0.000 | 0.000 | 0.000 | 0.000 | 0.000 | 0.000 | 0.000 | 0.000 | 0.000 | 0.000 | 0.000 | 0.000 | 0.000 | 0.000 |
| 25-722 | 0.000 | 0.000 | 0.000 | 0.000 | 0.000 | 0.000 | 0.000 | 0.000 | 0.000 | 0.000 | 0.000 | 0.000 | 0.000 | 0.000 | 0.000 | 0.000 | 0.000 | 0.000 | 0.000 | 0.000 | 0.053 | 0.000 |
| 25-729 | 0.000 | 0.000 | 0.000 | 0.125 | 0.000 | 0.000 | 0.000 | 0.000 | 0.000 | 0.000 | 0.000 | 0.000 | 0.000 | 0.000 | 0.000 | 0.000 | 0.000 | 0.000 | 0.045 | 0.000 | 0.053 | 0.000 |
| 25-734 | 0.000 | 0.000 | 0.000 | 0.125 | 0.000 | 0.000 | 0.000 | 0.000 | 0.000 | 0.000 | 0.000 | 0.000 | 0.000 | 0.000 | 0.000 | 0.000 | 0.000 | 0.043 | 0.045 | 0.000 | 0.000 | 0.000 |
| 25-741 | 0.000 | 0.000 | 0.043 | 0.042 | 0.050 | 0.000 | 0.000 | 0.000 | 0.000 | 0.000 | 0.043 | 0.000 | 0.000 | 0.000 | 0.000 | 0.000 | 0.000 | 0.043 | 0.000 | 0.000 | 0.000 | 0.000 |
| 25-746 | 0.000 | 0.000 | 0.087 | 0.208 | 0.000 | 0.000 | 0.174 | 0.000 | 0.000 | 0.000 | 0.000 | 0.000 | 0.000 | 0.000 | 0.000 | 0.000 | 0.000 | 0.043 | 0.000 | 0.000 | 0.000 | 0.000 |
| 25-775 | 0.000 | 0.000 | 0.000 | 0.000 | 0.000 | 0.000 | 0.043 | 0.000 | 0.000 | 0.000 | 0.000 | 0.000 | 0.000 | 0.000 | 0.000 | 0.000 | 0.000 | 0.000 | 0.000 | 0.000 | 0.000 | 0.000 |
| 25-780 | 0.000 | 0.000 | 0.000 | 0.125 | 0.000 | 0.000 | 0.304 | 0.000 | 0.000 | 0.000 | 0.000 | 0.000 | 0.000 | 0.000 | 0.000 | 0.000 | 0.000 | 0.000 | 0.000 | 0.000 | 0.000 | 0.000 |
| 25-790 | 0.045 | 0.000 | 0.565 | 0.375 | 0.050 | 0.208 | 0.391 | 0.000 | 0.000 | 0.000 | 0.652 | 0.000 | 0.000 | 0.000 | 0.000 | 0.000 | 0.000 | 0.043 | 0.000 | 0.000 | 0.000 | 0.000 |
| 25-795 | 0.000 | 0.000 | 0.522 | 0.417 | 0.050 | 0.125 | 0.304 | 0.000 | 0.000 | 0.000 | 0.652 | 0.000 | 0.000 | 0.048 | 0.000 | 0.000 | 0.000 | 0.000 | 0.000 | 0.000 | 0.000 | 0.000 |
| 25-806 | 0.000 | 0.000 | 0.043 | 0.000 | 0.000 | 0.000 | 0.087 | 0.000 | 0.000 | 0.045 | 0.000 | 0.000 | 0.000 | 0.000 | 0.000 | 0.000 | 0.000 | 0.000 | 0.045 | 0.000 | 0.000 | 0.000 |
| 25-812 | 0.000 | 0.000 | 0.522 | 0.375 | 0.000 | 0.000 | 0.261 | 0.000 | 0.083 | 0.045 | 0.565 | 0.000 | 0.000 | 0.000 | 0.000 | 0.000 | 0.000 | 0.000 | 0.000 | 0.000 | 0.000 | 0.000 |
| 25-822 | 0.000 | 0.000 | 0.348 | 0.250 | 0.000 | 0.000 | 0.217 | 0.000 | 0.042 | 0.000 | 0.348 | 0.000 | 0.000 | 0.000 | 0.000 | 0.000 | 0.000 | 0.000 | 0.045 | 0.000 | 0.000 | 0.000 |
| 25-826 | 0.000 | 0.000 | 0.000 | 0.000 | 0.000 | 0.000 | 0.043 | 0.000 | 0.000 | 0.000 | 0.000 | 0.000 | 0.000 | 0.000 | 0.000 | 0.000 | 0.000 | 0.000 | 0.000 | 0.000 | 0.000 | 0.000 |
| 25-834 | 0.000 | 0.000 | 0.000 | 0.000 | 0.000 | 0.000 | 0.043 | 0.000 | 0.000 | 0.000 | 0.000 | 0.000 | 0.000 | 0.000 | 0.000 | 0.000 | 0.000 | 0.000 | 0.000 | 0.042 | 0.000 | 0.000 |
| 25-848 | 0.000 | 0.000 | 0.696 | 0.375 | 0.000 | 0.000 | 0.348 | 0.100 | 0.208 | 0.227 | 0.696 | 0.000 | 0.042 | 0.000 | 0.000 | 0.000 | 0.000 | 0.043 | 0.000 | 0.000 | 0.000 | 0.000 |
| 25-862 | 0.000 | 0.000 | 0.087 | 0.042 | 0.000 | 0.000 | 0.043 | 0.000 | 0.000 | 0.000 | 0.000 | 0.043 | 0.000 | 0.000 | 0.000 | 0.000 | 0.000 | 0.087 | 0.000 | 0.000 | 0.000 | 0.000 |
| 25-870 | 0.000 | 0.000 | 0.087 | 0.458 | 0.000 | 0.000 | 0.000 | 0.350 | 0.208 | 0.318 | 0.043 | 0.000 | 0.000 | 0.000 | 0.000 | 0.048 | 0.042 | 0.391 | 0.091 | 0.000 | 0.316 | 0.304 |
| 25-874 | 0.000 | 0.000 | 0.043 | 0.042 | 0.000 | 0.000 | 0.261 | 0.000 | 0.000 | 0.091 | 0.000 | 0.000 | 0.000 | 0.000 | 0.053 | 0.000 | 0.000 | 0.174 | 0.045 | 0.000 | 0.211 | 0.043 |
| 25-878 | 0.000 | 0.000 | 0.087 | 0.458 | 0.000 | 0.042 | 0.000 | 0.350 | 0.167 | 0.318 | 0.043 | 0.087 | 0.000 | 0.000 | 0.000 | 0.048 | 0.042 | 0.435 | 0.136 | 0.042 | 0.316 | 0.217 |
| 25-884 | 0.000 | 0.083 | 0.087 | 0.083 | 0.000 | 0.000 | 0.261 | 0.000 | 0.000 | 0.091 | 0.000 | 0.000 | 0.000 | 0.000 | 0.000 | 0.000 | 0.000 | 0.174 | 0.045 | 0.042 | 0.263 | 0.130 |
| 25-897 | 0.000 | 0.000 | 0.000 | 0.000 | 0.000 | 0.000 | 0.000 | 0.000 | 0.000 | 0.000 | 0.000 | 0.000 | 0.000 | 0.000 | 0.000 | 0.000 | 0.000 | 0.000 | 0.000 | 0.000 | 0.053 | 0.000 |
| 25-914 | 0.000 | 0.000 | 0.391 | 0.125 | 0.000 | 0.000 | 0.348 | 0.000 | 0.000 | 0.000 | 0.261 | 0.000 | 0.000 | 0.000 | 0.000 | 0.000 | 0.000 | 0.000 | 0.000 | 0.000 | 0.000 | 0.000 |
| 25-922 | 0.000 | 0.000 | 0.087 | 0.333 | 0.000 | 0.000 | 0.217 | 0.000 | 0.000 | 0.000 | 0.174 | 0.000 | 0.000 | 0.000 | 0.000 | 0.000 | 0.000 | 0.000 | 0.000 | 0.000 | 0.000 | 0.000 |
| 25-926 | 0.000 | 0.000 | 0.130 | 0.458 | 0.000 | 0.000 | 0.348 | 0.000 | 0.000 | 0.000 | 0.174 | 0.000 | 0.000 | 0.000 | 0.000 | 0.000 | 0.000 | 0.000 | 0.000 | 0.000 | 0.053 | 0.000 |
| 25-957 | 0.000 | 0.000 | 0.000 | 0.000 | 0.000 | 0.000 | 0.000 | 0.000 | 0.000 | 0.000 | 0.000 | 0.000 | 0.000 | 0.000 | 0.000 | 0.000 | 0.000 | 0.043 | 0.000 | 0.000 | 0.105 | 0.000 |
| 25-966 | 0.000 | 0.000 | 0.000 | 0.000 | 0.000 | 0.000 | 0.000 | 0.000 | 0.000 | 0.000 | 0.000 | 0.000 | 0.000 | 0.000 | 0.000 | 0.000 | 0.000 | 0.000 | 0.000 | 0.000 | 0.053 | 0.000 |
| 25-971 | 0.000 | 0.000 | 0.000 | 0.000 | 0.000 | 0.000 | 0.000 | 0.000 | 0.000 | 0.000 | 0.000 | 0.000 | 0.000 | 0.000 | 0.000 | 0.000 | 0.000 | 0.043 | 0.045 | 0.000 | 0.105 | 0.000 |
| 25-974 | 0.000 | 0.000 | 0.000 | 0.000 | 0.000 | 0.000 | 0.043 | 0.000 | 0.000 | 0.000 | 0.000 | 0.000 | 0.000 | 0.000 | 0.000 | 0.000 | 0.000 | 0.043 | 0.000 | 0.000 | 0.105 | 0.000 |
| 25-983 | 0.000 | 0.000 | 0.000 | 0.000 | 0.000 | 0.000 | 0.043 | 0.000 | 0.000 | 0.000 | 0.000 | 0.000 | 0.000 | 0.000 | 0.000 | 0.000 | 0.042 | 0.000 | 0.000 | 0.042 | 0.000 | 0.000 |
| 25-987 | 0.000 | 0.000 | 0.043 | 0.000 | 0.000 | 0.000 | 0.000 | 0.000 | 0.000 | 0.000 | 0.000 | 0.000 | 0.000 | 0.000 | 0.000 | 0.000 | 0.042 | 0.000 | 0.000 | 0.042 | 0.000 | 0.000 |
| 25-992 | 0.000 | 0.000 | 0.043 | 0.167 | 0.000 | 0.000 | 0.130 | 0.000 | 0.000 | 0.000 | 0.000 | 0.000 | 0.000 | 0.000 | 0.000 | 0.000 | 0.000 | 0.000 | 0.000 | 0.000 | 0.000 | 0.000 |
| 25-998 | 0.000 | 0.000 | 0.000 | 0.125 | 0.000 | 0.000 | 0.174 | 0.000 | 0.000 | 0.000 | 0.000 | 0.000 | 0.000 | 0.000 | 0.000 | 0.000 | 0.000 | 0.000 | 0.000 | 0.000 | 0.000 | 0.000 |
| 25-1013 | 0.000 | 0.000 | 0.000 | 0.000 | 0.000 | 0.000 | 0.000 | 0.000 | 0.000 | 0.000 | 0.000 | 0.000 | 0.000 | 0.000 | 0.000 | 0.000 | 0.000 | 0.000 | 0.000 | 0.000 | 0.053 | 0.000 |
| 25-1020 | 0.000 | 0.000 | 0.000 | 0.000 | 0.000 | 0.000 | 0.000 | 0.000 | 0.000 | 0.000 | 0.000 | 0.000 | 0.000 | 0.000 | 0.000 | 0.000 | 0.000 | 0.043 | 0.000 | 0.000 | 0.000 | 0.000 |
| 25-1031 | 0.000 | 0.000 | 0.000 | 0.000 | 0.000 | 0.000 | 0.000 | 0.000 | 0.000 | 0.000 | 0.000 | 0.000 | 0.000 | 0.000 | 0.000 | 0.000 | 0.000 | 0.043 | 0.000 | 0.000 | 0.053 | 0.000 |
| 25-1037 | 0.000 | 0.000 | 0.043 | 0.000 | 0.000 | 0.000 | 0.043 | 0.050 | 0.000 | 0.000 | 0.000 | 0.043 | 0.000 | 0.000 | 0.000 | 0.000 | 0.000 | 0.043 | 0.045 | 0.000 | 0.158 | 0.000 |
| 25-1045 | 0.000 | 0.042 | 0.043 | 0.000 | 0.000 | 0.000 | 0.000 | 0.050 | 0.042 | 0.000 | 0.000 | 0.043 | 0.000 | 0.000 | 0.000 | 0.000 | 0.000 | 0.174 | 0.045 | 0.000 | 0.105 | 0.000 |
| 25-1052 | 0.000 | 0.042 | 0.043 | 0.000 | 0.000 | 0.000 | 0.000 | 0.000 | 0.042 | 0.000 | 0.000 | 0.000 | 0.000 | 0.000 | 0.000 | 0.000 | 0.083 | 0.043 | 0.091 | 0.000 | 0.211 | 0.043 |
| 25-1057 | 0.000 | 0.000 | 0.000 | 0.000 | 0.000 | 0.000 | 0.000 | 0.050 | 0.000 | 0.000 | 0.000 | 0.000 | 0.000 | 0.000 | 0.000 | 0.000 | 0.083 | 0.130 | 0.091 | 0.000 | 0.263 | 0.043 |
| 25-1062 | 0.000 | 0.042 | 0.000 | 0.000 | 0.000 | 0.042 | 0.043 | 0.100 | 0.000 | 0.000 | 0.000 | 0.043 | 0.042 | 0.000 | 0.000 | 0.000 | 0.208 | 0.130 | 0.136 | 0.042 | 0.211 | 0.087 |
| 25-1067 | 0.045 | 0.083 | 0.043 | 0.042 | 0.000 | 0.042 | 0.043 | 0.100 | 0.000 | 0.000 | 0.000 | 0.130 | 0.042 | 0.000 | 0.000 | 0.000 | 0.167 | 0.391 | 0.227 | 0.125 | 0.211 | 0.087 |
| 25-1072 | 0.045 | 0.042 | 0.130 | 0.042 | 0.050 | 0.083 | 0.043 | 0.050 | 0.000 | 0.000 | 0.000 | 0.087 | 0.083 | 0.048 | 0.000 | 0.000 | 0.042 | 0.435 | 0.273 | 0.125 | 0.053 | 0.174 |
| 25-1077 | 0.000 | 0.042 | 0.087 | 0.000 | 0.050 | 0.000 | 0.043 | 0.000 | 0.000 | 0.000 | 0.000 | 0.087 | 0.042 | 0.000 | 0.000 | 0.000 | 0.042 | 0.174 | 0.091 | 0.000 | 0.053 | 0.087 |
| 25-1084 | 0.000 | 0.167 | 0.261 | 0.917 | 0.000 | 0.208 | 0.696 | 0.300 | 0.500 | 0.773 | 0.304 | 0.000 | 0.000 | 0.048 | 0.053 | 0.286 | 0.083 | 0.043 | 0.182 | 0.167 | 0.158 | 0.261 |
| 25-1090 | 0.000 | 0.042 | 0.261 | 0.917 | 0.000 | 0.208 | 0.696 | 0.250 | 0.667 | 0.636 | 0.261 | 0.000 | 0.000 | 0.095 | 0.053 | 0.190 | 0.125 | 0.043 | 0.227 | 0.125 | 0.158 | 0.130 |
| 25-1100 | 0.000 | 0.000 | 0.087 | 0.000 | 0.000 | 0.000 | 0.000 | 0.000 | 0.000 | 0.000 | 0.000 | 0.000 | 0.000 | 0.000 | 0.000 | 0.000 | 0.000 | 0.000 | 0.000 | 0.000 | 0.000 | 0.000 |
| 25-1111 | 0.000 | 0.000 | 0.000 | 0.042 | 0.050 | 0.000 | 0.000 | 0.000 | 0.000 | 0.000 | 0.000 | 0.000 | 0.000 | 0.000 | 0.000 | 0.000 | 0.000 | 0.043 | 0.000 | 0.000 | 0.000 | 0.000 |
| 25-1120 | 0.000 | 0.000 | 0.043 | 0.000 | 0.000 | 0.000 | 0.000 | 0.000 | 0.000 | 0.000 | 0.000 | 0.000 | 0.000 | 0.000 | 0.000 | 0.000 | 0.000 | 0.000 | 0.000 | 0.000 | 0.000 | 0.000 |
| 25-1130 | 0.000 | 0.000 | 0.000 | 0.042 | 0.000 | 0.000 | 0.000 | 0.000 | 0.000 | 0.000 | 0.000 | 0.000 | 0.000 | 0.000 | 0.000 | 0.000 | 0.000 | 0.000 | 0.000 | 0.000 | 0.053 | 0.000 |
| 25-1137 | 0.000 | 0.000 | 0.043 | 0.083 | 0.000 | 0.000 | 0.087 | 0.000 | 0.000 | 0.000 | 0.000 | 0.000 | 0.000 | 0.000 | 0.000 | 0.000 | 0.000 | 0.000 | 0.000 | 0.000 | 0.053 | 0.000 |
| 25-1143 | 0.000 | 0.000 | 0.087 | 0.250 | 0.000 | 0.000 | 0.000 | 0.000 | 0.000 | 0.000 | 0.130 | 0.000 | 0.000 | 0.000 | 0.000 | 0.000 | 0.000 | 0.000 | 0.000 | 0.000 | 0.000 | 0.000 |
| 25-1154 | 0.000 | 0.000 | 0.000 | 0.083 | 0.000 | 0.000 | 0.261 | 0.000 | 0.000 | 0.000 | 0.000 | 0.000 | 0.000 | 0.000 | 0.000 | 0.000 | 0.000 | 0.000 | 0.000 | 0.000 | 0.000 | 0.000 |
| 25-1171 | 0.000 | 0.000 | 0.000 | 0.000 | 0.050 | 0.000 | 0.000 | 0.000 | 0.000 | 0.000 | 0.000 | 0.000 | 0.000 | 0.000 | 0.000 | 0.000 | 0.000 | 0.000 | 0.000 | 0.000 | 0.053 | 0.000 |
| 25-1176 | 0.000 | 0.000 | 0.000 | 0.000 | 0.050 | 0.000 | 0.000 | 0.000 | 0.000 | 0.000 | 0.000 | 0.000 | 0.000 | 0.000 | 0.000 | 0.000 | 0.000 | 0.000 | 0.000 | 0.000 | 0.000 | 0.000 |
| 25-1198 | 0.000 | 0.000 | 0.000 | 0.000 | 0.000 | 0.000 | 0.000 | 0.000 | 0.000 | 0.000 | 0.043 | 0.000 | 0.000 | 0.000 | 0.000 | 0.000 | 0.000 | 0.000 | 0.000 | 0.000 | 0.000 | 0.000 |
| 27-100 | 0.000 | 0.000 | 0.000 | 0.000 | 0.000 | 0.000 | 0.000 | 0.000 | 0.067 | 0.000 | 0.000 | 0.000 | 0.000 | 0.000 | 0.000 | 0.000 | 0.000 | 0.000 | 0.000 | 0.000 | 0.000 | 0.000 |
| 27-105 | 0.000 | 0.000 | 0.000 | 0.000 | 0.000 | 0.000 | 0.000 | 0.000 | 0.000 | 0.000 | 0.045 | 0.042 | 0.000 | 0.087 | 0.150 | 0.000 | 0.042 | 0.042 | 0.042 | 0.000 | 0.091 | 0.043 |
| 27-109 | 0.429 | 0.083 | 0.364 | 0.417 | 0.100 | 0.417 | 0.565 | 0.950 | 0.000 | 0.000 | 0.091 | 0.875 | 0.542 | 0.652 | 0.450 | 0.429 | 0.625 | 0.875 | 0.625 | 0.042 | 0.682 | 0.348 |
| 27-122 | 0.000 | 0.000 | 0.000 | 0.000 | 0.000 | 0.000 | 0.000 | 0.000 | 0.000 | 0.000 | 0.045 | 0.000 | 0.000 | 0.087 | 0.000 | 0.000 | 0.000 | 0.042 | 0.000 | 0.000 | 0.000 | 0.000 |
| 27-130 | 0.000 | 0.000 | 0.000 | 0.000 | 0.000 | 0.000 | 0.000 | 0.000 | 0.000 | 0.000 | 0.045 | 0.000 | 0.042 | 0.000 | 0.000 | 0.000 | 0.000 | 0.000 | 0.000 | 0.000 | 0.000 | 0.000 |
| 27-136 | 0.000 | 0.000 | 0.045 | 0.000 | 0.000 | 0.000 | 0.000 | 0.000 | 0.067 | 0.000 | 0.000 | 0.000 | 0.000 | 0.000 | 0.000 | 0.048 | 0.000 | 0.000 | 0.000 | 0.000 | 0.000 | 0.000 |
| 27-150 | 0.000 | 0.000 | 0.000 | 0.000 | 0.050 | 0.000 | 0.000 | 0.000 | 0.067 | 0.000 | 0.000 | 0.125 | 0.000 | 0.304 | 0.200 | 0.000 | 0.083 | 0.167 | 0.125 | 0.000 | 0.318 | 0.000 |
| 27-154 | 0.000 | 0.000 | 0.000 | 0.000 | 0.000 | 0.000 | 0.000 | 0.000 | 0.000 | 0.000 | 0.000 | 0.125 | 0.000 | 0.304 | 0.050 | 0.000 | 0.000 | 0.333 | 0.042 | 0.000 | 0.000 | 0.000 |
| 27-160 | 0.000 | 0.000 | 0.000 | 0.000 | 0.000 | 0.000 | 0.000 | 0.050 | 0.000 | 0.000 | 0.000 | 0.000 | 0.000 | 0.043 | 0.000 | 0.000 | 0.000 | 0.000 | 0.000 | 0.000 | 0.000 | 0.000 |
| 27-168 | 0.333 | 0.417 | 0.000 | 0.042 | 0.350 | 0.208 | 0.043 | 0.200 | 0.000 | 0.000 | 0.000 | 0.375 | 0.667 | 0.348 | 0.700 | 0.762 | 0.417 | 0.583 | 0.208 | 0.333 | 0.273 | 0.478 |
| 27-172 | 0.333 | 0.083 | 0.045 | 0.042 | 0.250 | 0.208 | 0.000 | 0.300 | 0.000 | 0.000 | 0.000 | 0.417 | 0.417 | 0.696 | 0.700 | 0.238 | 0.458 | 0.583 | 0.500 | 0.000 | 0.682 | 0.174 |
| 27-180 | 0.190 | 0.125 | 0.000 | 0.000 | 0.200 | 0.083 | 0.000 | 0.200 | 0.000 | 0.000 | 0.000 | 0.333 | 0.625 | 0.348 | 0.550 | 0.571 | 0.458 | 0.042 | 0.292 | 0.208 | 0.682 | 0.261 |
| 27-190 | 0.000 | 0.000 | 0.000 | 0.000 | 0.000 | 0.000 | 0.000 | 0.000 | 0.000 | 0.000 | 0.000 | 0.042 | 0.125 | 0.261 | 0.200 | 0.000 | 0.042 | 0.083 | 0.042 | 0.000 | 0.136 | 0.043 |
| 27-197 | 0.000 | 0.000 | 0.000 | 0.000 | 0.000 | 0.000 | 0.000 | 0.000 | 0.000 | 0.000 | 0.000 | 0.042 | 0.000 | 0.261 | 0.200 | 0.000 | 0.000 | 0.167 | 0.042 | 0.000 | 0.045 | 0.000 |
| 27-200 | 0.000 | 0.000 | 0.045 | 0.000 | 0.000 | 0.000 | 0.000 | 0.000 | 0.000 | 0.000 | 0.045 | 0.000 | 0.042 | 0.000 | 0.000 | 0.000 | 0.000 | 0.000 | 0.000 | 0.000 | 0.000 | 0.000 |
| 27-209 | 0.000 | 0.000 | 0.000 | 0.000 | 0.000 | 0.000 | 0.000 | 0.000 | 0.000 | 0.000 | 0.000 | 0.000 | 0.042 | 0.000 | 0.050 | 0.000 | 0.042 | 0.000 | 0.000 | 0.000 | 0.000 | 0.000 |
| 27-212 | 0.000 | 0.000 | 0.000 | 0.000 | 0.000 | 0.000 | 0.000 | 0.000 | 0.000 | 0.000 | 0.000 | 0.042 | 0.000 | 0.087 | 0.050 | 0.000 | 0.042 | 0.250 | 0.083 | 0.000 | 0.045 | 0.000 |
| 27-217 | 0.000 | 0.000 | 0.000 | 0.000 | 0.000 | 0.000 | 0.000 | 0.000 | 0.000 | 0.000 | 0.000 | 0.000 | 0.000 | 0.087 | 0.050 | 0.000 | 0.000 | 0.250 | 0.000 | 0.000 | 0.000 | 0.000 |
| 27-226 | 0.000 | 0.000 | 0.000 | 0.000 | 0.000 | 0.000 | 0.000 | 0.000 | 0.000 | 0.000 | 0.000 | 0.000 | 0.000 | 0.000 | 0.000 | 0.000 | 0.000 | 0.000 | 0.000 | 0.000 | 0.045 | 0.000 |
| 27-231 | 0.000 | 0.000 | 0.000 | 0.000 | 0.000 | 0.000 | 0.000 | 0.050 | 0.000 | 0.000 | 0.000 | 0.000 | 0.083 | 0.087 | 0.000 | 0.000 | 0.042 | 0.042 | 0.000 | 0.083 | 0.091 | 0.043 |
| 27-239 | 0.000 | 0.000 | 0.000 | 0.000 | 0.000 | 0.000 | 0.000 | 0.000 | 0.000 | 0.000 | 0.000 | 0.000 | 0.000 | 0.000 | 0.000 | 0.048 | 0.000 | 0.042 | 0.000 | 0.125 | 0.000 | 0.043 |
| 27-246 | 0.048 | 0.208 | 0.000 | 0.000 | 0.000 | 0.000 | 0.000 | 0.000 | 0.000 | 0.000 | 0.000 | 0.000 | 0.042 | 0.130 | 0.100 | 0.000 | 0.000 | 0.208 | 0.000 | 0.000 | 0.091 | 0.000 |
| 27-250 | 0.000 | 0.083 | 0.000 | 0.000 | 0.000 | 0.000 | 0.000 | 0.000 | 0.000 | 0.000 | 0.000 | 0.208 | 0.000 | 0.261 | 0.350 | 0.095 | 0.250 | 0.167 | 0.000 | 0.000 | 0.091 | 0.000 |
| 27-255 | 0.762 | 0.833 | 0.364 | 0.167 | 0.250 | 0.042 | 0.826 | 0.050 | 0.000 | 0.071 | 0.909 | 0.958 | 0.583 | 0.870 | 0.850 | 0.905 | 1.000 | 0.958 | 0.792 | 0.833 | 0.455 | 0.261 |
| 27-260 | 0.810 | 0.875 | 0.364 | 0.125 | 0.200 | 0.083 | 0.826 | 0.000 | 0.000 | 0.071 | 0.864 | 0.458 | 0.292 | 0.783 | 0.800 | 0.905 | 1.000 | 0.875 | 0.667 | 0.792 | 0.455 | 0.870 |
| 27-265 | 1.000 | 0.875 | 0.091 | 0.292 | 0.750 | 0.042 | 0.565 | 0.100 | 0.133 | 0.143 | 0.000 | 0.125 | 0.667 | 0.478 | 0.400 | 1.000 | 1.000 | 0.583 | 0.458 | 0.833 | 0.455 | 0.957 |
| 27-274 | 0.952 | 1.000 | 0.955 | 0.833 | 1.000 | 0.875 | 0.957 | 0.950 | 0.067 | 0.143 | 0.955 | 1.000 | 1.000 | 0.957 | 1.000 | 0.952 | 1.000 | 1.000 | 0.958 | 0.958 | 1.000 | 1.000 |
| 27-280 | 0.810 | 0.708 | 0.091 | 0.083 | 0.800 | 0.083 | 0.087 | 0.050 | 0.067 | 0.000 | 0.091 | 0.583 | 0.792 | 0.652 | 0.850 | 0.571 | 1.000 | 0.750 | 0.917 | 0.500 | 0.636 | 0.609 |
| 27-286 | 0.333 | 0.417 | 0.045 | 0.000 | 0.250 | 0.000 | 0.043 | 0.350 | 0.133 | 0.000 | 0.318 | 0.917 | 0.625 | 0.826 | 0.750 | 0.286 | 0.917 | 0.958 | 0.875 | 0.458 | 0.818 | 0.348 |
| 27-294 | 0.000 | 0.292 | 0.136 | 0.042 | 0.050 | 0.000 | 0.174 | 0.000 | 0.067 | 0.000 | 0.182 | 0.083 | 0.042 | 0.000 | 0.200 | 0.048 | 0.042 | 0.417 | 0.125 | 0.125 | 0.136 | 0.000 |
| 27-298 | 0.000 | 0.042 | 0.000 | 0.000 | 0.000 | 0.000 | 0.000 | 0.000 | 0.067 | 0.000 | 0.000 | 0.042 | 0.042 | 0.000 | 0.100 | 0.000 | 0.000 | 0.042 | 0.125 | 0.083 | 0.000 | 0.043 |
| 27-306 | 1.000 | 0.833 | 0.955 | 0.875 | 1.000 | 0.958 | 0.696 | 0.950 | 0.267 | 0.571 | 0.955 | 1.000 | 1.000 | 0.957 | 1.000 | 0.952 | 1.000 | 1.000 | 1.000 | 0.875 | 1.000 | 1.000 |
| 27-317 | 0.000 | 0.000 | 0.000 | 0.000 | 0.000 | 0.000 | 0.000 | 0.000 | 0.067 | 0.000 | 0.000 | 0.083 | 0.042 | 0.087 | 0.050 | 0.000 | 0.000 | 0.000 | 0.000 | 0.000 | 0.045 | 0.000 |
| 27-325 | 0.000 | 0.000 | 0.045 | 0.000 | 0.000 | 0.000 | 0.000 | 0.000 | 0.133 | 0.000 | 0.091 | 0.083 | 0.000 | 0.087 | 0.300 | 0.000 | 0.000 | 0.000 | 0.083 | 0.042 | 0.045 | 0.000 |
| 27-341 | 0.000 | 0.000 | 0.000 | 0.000 | 0.000 | 0.042 | 0.000 | 0.000 | 0.000 | 0.000 | 0.000 | 0.000 | 0.000 | 0.000 | 0.000 | 0.000 | 0.000 | 0.000 | 0.000 | 0.000 | 0.000 | 0.043 |
| 27-347 | 0.000 | 0.000 | 0.000 | 0.000 | 0.000 | 0.083 | 0.000 | 0.400 | 0.000 | 0.000 | 0.000 | 0.375 | 0.042 | 0.391 | 0.150 | 0.000 | 0.500 | 0.667 | 0.292 | 0.042 | 0.500 | 0.000 |
| 27-354 | 0.000 | 0.000 | 0.182 | 0.000 | 0.000 | 0.083 | 0.000 | 0.350 | 0.000 | 0.000 | 0.136 | 0.208 | 0.083 | 0.130 | 0.050 | 0.048 | 0.042 | 0.167 | 0.250 | 0.042 | 0.182 | 0.000 |
| 27-362 | 0.000 | 0.000 | 0.045 | 0.000 | 0.000 | 0.000 | 0.000 | 0.000 | 0.067 | 0.000 | 0.000 | 0.125 | 0.167 | 0.000 | 0.000 | 0.000 | 0.000 | 0.000 | 0.000 | 0.000 | 0.000 | 0.000 |
| 27-366 | 0.000 | 0.000 | 0.000 | 0.000 | 0.000 | 0.000 | 0.000 | 0.000 | 0.067 | 0.000 | 0.000 | 0.125 | 0.167 | 0.043 | 0.000 | 0.000 | 0.000 | 0.000 | 0.000 | 0.000 | 0.000 | 0.000 |
| 27-374 | 0.000 | 0.000 | 0.000 | 0.000 | 0.000 | 0.000 | 0.000 | 0.000 | 0.000 | 0.000 | 0.000 | 0.000 | 0.083 | 0.043 | 0.000 | 0.095 | 0.042 | 0.000 | 0.000 | 0.000 | 0.000 | 0.000 |
| 27-383 | 0.571 | 0.250 | 0.182 | 0.167 | 0.050 | 0.083 | 0.174 | 0.350 | 0.067 | 0.000 | 0.000 | 0.625 | 0.417 | 0.783 | 0.450 | 0.333 | 0.667 | 0.625 | 0.250 | 0.208 | 0.591 | 0.174 |
| 27-387 | 0.571 | 0.375 | 0.273 | 0.125 | 0.100 | 0.083 | 0.217 | 0.350 | 0.067 | 0.000 | 0.000 | 0.625 | 0.583 | 0.826 | 0.450 | 0.619 | 0.750 | 0.625 | 0.250 | 0.250 | 0.636 | 0.348 |
| 27-390 | 0.238 | 0.208 | 0.091 | 0.000 | 0.450 | 0.042 | 0.087 | 0.100 | 0.000 | 0.000 | 0.000 | 0.000 | 0.250 | 0.435 | 0.300 | 0.190 | 0.333 | 0.417 | 0.000 | 0.208 | 0.182 | 0.000 |
| 27-393 | 0.286 | 0.292 | 0.091 | 0.000 | 0.450 | 0.042 | 0.000 | 0.100 | 0.000 | 0.000 | 0.045 | 0.042 | 0.208 | 0.565 | 0.250 | 0.143 | 0.333 | 0.542 | 0.042 | 0.208 | 0.136 | 0.000 |
| 27-397 | 0.000 | 0.000 | 0.000 | 0.000 | 0.000 | 0.000 | 0.000 | 0.000 | 0.000 | 0.000 | 0.000 | 0.000 | 0.000 | 0.130 | 0.000 | 0.000 | 0.000 | 0.000 | 0.000 | 0.000 | 0.000 | 0.000 |
| 27-400 | 0.000 | 0.000 | 0.000 | 0.000 | 0.000 | 0.000 | 0.000 | 0.000 | 0.000 | 0.000 | 0.000 | 0.000 | 0.000 | 0.000 | 0.000 | 0.000 | 0.000 | 0.042 | 0.000 | 0.000 | 0.000 | 0.000 |
| 27-405 | 0.000 | 0.000 | 0.000 | 0.000 | 0.000 | 0.000 | 0.000 | 0.000 | 0.067 | 0.000 | 0.000 | 0.000 | 0.000 | 0.043 | 0.000 | 0.000 | 0.000 | 0.042 | 0.000 | 0.000 | 0.000 | 0.000 |
| 27-410 | 0.000 | 0.000 | 0.000 | 0.125 | 0.100 | 0.000 | 0.000 | 0.000 | 0.000 | 0.000 | 0.000 | 0.000 | 0.083 | 0.043 | 0.000 | 0.000 | 0.042 | 0.000 | 0.000 | 0.125 | 0.045 | 0.000 |
| 27-415 | 0.000 | 0.000 | 0.000 | 0.083 | 0.100 | 0.000 | 0.000 | 0.000 | 0.000 | 0.000 | 0.000 | 0.000 | 0.083 | 0.043 | 0.050 | 0.048 | 0.042 | 0.000 | 0.000 | 0.125 | 0.000 | 0.000 |
| 27-422 | 0.000 | 0.000 | 0.000 | 0.000 | 0.000 | 0.042 | 0.000 | 0.000 | 0.000 | 0.000 | 0.000 | 0.000 | 0.000 | 0.000 | 0.000 | 0.048 | 0.000 | 0.000 | 0.000 | 0.083 | 0.045 | 0.000 |
| 27-428 | 0.048 | 0.000 | 0.045 | 0.000 | 0.000 | 0.000 | 0.000 | 0.000 | 0.000 | 0.000 | 0.000 | 0.250 | 0.292 | 0.304 | 0.300 | 0.095 | 0.458 | 0.250 | 0.167 | 0.000 | 0.364 | 0.000 |
| 27-434 | 0.000 | 0.042 | 0.045 | 0.000 | 0.050 | 0.000 | 0.000 | 0.000 | 0.000 | 0.000 | 0.000 | 0.125 | 0.250 | 0.087 | 0.250 | 0.095 | 0.375 | 0.417 | 0.083 | 0.000 | 0.318 | 0.000 |
| 27-438 | 0.000 | 0.000 | 0.000 | 0.000 | 0.000 | 0.000 | 0.000 | 0.000 | 0.000 | 0.000 | 0.000 | 0.000 | 0.042 | 0.174 | 0.200 | 0.000 | 0.083 | 0.083 | 0.000 | 0.000 | 0.091 | 0.000 |
| 27-450 | 0.286 | 0.375 | 0.182 | 0.000 | 0.000 | 0.000 | 0.217 | 0.000 | 0.000 | 0.000 | 0.545 | 0.208 | 0.042 | 0.087 | 0.300 | 0.143 | 0.250 | 0.250 | 0.083 | 0.208 | 0.000 | 0.043 |
| 27-454 | 0.476 | 0.458 | 0.182 | 0.042 | 0.400 | 0.417 | 0.478 | 0.550 | 0.000 | 0.000 | 0.364 | 0.625 | 0.292 | 0.348 | 0.450 | 0.714 | 0.667 | 0.583 | 0.542 | 0.333 | 0.682 | 0.522 |
| 27-462 | 0.000 | 0.000 | 0.091 | 0.000 | 0.000 | 0.042 | 0.000 | 0.000 | 0.000 | 0.000 | 0.045 | 0.000 | 0.000 | 0.043 | 0.000 | 0.143 | 0.083 | 0.042 | 0.083 | 0.125 | 0.000 | 0.087 |
| 27-469 | 0.048 | 0.000 | 0.000 | 0.000 | 0.000 | 0.000 | 0.000 | 0.000 | 0.000 | 0.000 | 0.045 | 0.000 | 0.000 | 0.000 | 0.000 | 0.000 | 0.000 | 0.000 | 0.000 | 0.000 | 0.000 | 0.000 |
| 27-473 | 0.000 | 0.000 | 0.000 | 0.000 | 0.000 | 0.000 | 0.000 | 0.000 | 0.000 | 0.000 | 0.045 | 0.000 | 0.000 | 0.000 | 0.000 | 0.000 | 0.000 | 0.000 | 0.000 | 0.000 | 0.000 | 0.000 |
| 27-484 | 0.000 | 0.000 | 0.000 | 0.000 | 0.000 | 0.000 | 0.000 | 0.000 | 0.000 | 0.000 | 0.000 | 0.000 | 0.000 | 0.043 | 0.050 | 0.000 | 0.042 | 0.000 | 0.000 | 0.000 | 0.045 | 0.000 |
| 27-490 | 0.000 | 0.000 | 0.000 | 0.000 | 0.000 | 0.000 | 0.000 | 0.000 | 0.000 | 0.000 | 0.000 | 0.000 | 0.000 | 0.087 | 0.050 | 0.000 | 0.042 | 0.000 | 0.000 | 0.000 | 0.045 | 0.000 |
| 27-504 | 0.000 | 0.000 | 0.000 | 0.000 | 0.000 | 0.042 | 0.000 | 0.000 | 0.000 | 0.000 | 0.227 | 0.208 | 0.208 | 0.087 | 0.150 | 0.143 | 0.083 | 0.042 | 0.208 | 0.000 | 0.182 | 0.130 |
| 27-510 | 0.000 | 0.042 | 0.000 | 0.000 | 0.000 | 0.000 | 0.000 | 0.050 | 0.000 | 0.000 | 0.045 | 0.208 | 0.083 | 0.087 | 0.100 | 0.238 | 0.083 | 0.083 | 0.000 | 0.000 | 0.182 | 0.000 |
| 27-519 | 0.714 | 0.625 | 0.545 | 0.125 | 0.350 | 0.250 | 0.478 | 0.650 | 0.067 | 0.000 | 0.955 | 0.792 | 0.708 | 0.609 | 0.700 | 0.667 | 0.750 | 0.917 | 0.333 | 0.500 | 0.864 | 0.391 |
| 27-526 | 0.714 | 0.708 | 0.682 | 0.792 | 0.850 | 0.750 | 0.696 | 0.900 | 0.333 | 0.214 | 0.955 | 0.917 | 0.750 | 0.783 | 0.850 | 0.667 | 0.708 | 0.917 | 0.667 | 0.875 | 0.773 | 0.565 |
| 27-539 | 0.000 | 0.000 | 0.045 | 0.000 | 0.000 | 0.000 | 0.000 | 0.000 | 0.000 | 0.000 | 0.045 | 0.000 | 0.000 | 0.130 | 0.000 | 0.000 | 0.000 | 0.000 | 0.000 | 0.042 | 0.000 | 0.000 |
| 27-543 | 0.000 | 0.000 | 0.000 | 0.000 | 0.000 | 0.000 | 0.000 | 0.000 | 0.000 | 0.000 | 0.000 | 0.000 | 0.000 | 0.130 | 0.000 | 0.000 | 0.000 | 0.042 | 0.000 | 0.000 | 0.000 | 0.000 |
| 27-546 | 0.000 | 0.000 | 0.045 | 0.000 | 0.000 | 0.000 | 0.000 | 0.000 | 0.067 | 0.000 | 0.045 | 0.000 | 0.000 | 0.000 | 0.000 | 0.000 | 0.042 | 0.000 | 0.000 | 0.000 | 0.000 | 0.000 |
| 27-555 | 0.048 | 0.333 | 0.500 | 0.333 | 0.600 | 0.042 | 0.217 | 0.200 | 0.000 | 0.000 | 0.000 | 0.250 | 0.542 | 0.565 | 0.350 | 0.286 | 0.542 | 0.750 | 0.167 | 0.542 | 0.500 | 0.696 |
| 27-561 | 0.048 | 0.375 | 0.500 | 0.208 | 0.750 | 0.042 | 0.174 | 0.200 | 0.000 | 0.000 | 0.045 | 0.250 | 0.500 | 0.522 | 0.350 | 0.333 | 0.542 | 0.833 | 0.167 | 0.667 | 0.500 | 0.783 |
| 27-567 | 0.524 | 0.167 | 0.091 | 0.000 | 0.250 | 0.000 | 0.130 | 0.050 | 0.000 | 0.000 | 0.091 | 0.792 | 0.500 | 0.565 | 0.550 | 0.286 | 0.875 | 0.208 | 0.375 | 0.250 | 0.636 | 0.565 |
| 27-570 | 0.524 | 0.125 | 0.000 | 0.000 | 0.000 | 0.000 | 0.000 | 0.000 | 0.000 | 0.000 | 0.000 | 0.250 | 0.333 | 0.174 | 0.400 | 0.143 | 0.625 | 0.042 | 0.125 | 0.042 | 0.045 | 0.130 |
| 27-575 | 0.333 | 0.000 | 0.045 | 0.000 | 0.000 | 0.000 | 0.000 | 0.000 | 0.000 | 0.000 | 0.000 | 0.208 | 0.208 | 0.087 | 0.100 | 0.143 | 0.583 | 0.083 | 0.083 | 0.000 | 0.091 | 0.000 |
| 27-580 | 0.381 | 0.042 | 0.045 | 0.000 | 0.000 | 0.000 | 0.000 | 0.000 | 0.000 | 0.000 | 0.000 | 0.333 | 0.167 | 0.174 | 0.150 | 0.190 | 0.625 | 0.042 | 0.083 | 0.083 | 0.045 | 0.087 |
| 27-587 | 0.476 | 0.375 | 0.364 | 0.000 | 0.200 | 0.000 | 0.130 | 0.000 | 0.000 | 0.000 | 0.045 | 0.625 | 0.417 | 0.565 | 0.450 | 0.381 | 0.625 | 0.167 | 0.167 | 0.500 | 0.409 | 0.304 |
| 27-592 | 0.381 | 0.417 | 0.409 | 0.042 | 0.300 | 0.125 | 0.130 | 0.050 | 0.200 | 0.357 | 0.364 | 0.667 | 0.625 | 0.609 | 0.700 | 0.524 | 0.625 | 0.333 | 0.417 | 0.417 | 0.500 | 0.652 |
| 27-600 | 0.429 | 0.375 | 0.727 | 0.208 | 0.200 | 0.208 | 0.391 | 0.250 | 0.000 | 0.000 | 0.045 | 0.417 | 0.542 | 0.565 | 0.700 | 0.667 | 0.583 | 0.625 | 0.500 | 0.333 | 0.500 | 0.565 |
| 27-609 | 0.857 | 0.500 | 0.909 | 0.292 | 0.600 | 0.125 | 0.435 | 0.250 | 0.000 | 0.000 | 0.818 | 0.917 | 0.875 | 0.870 | 1.000 | 0.810 | 1.000 | 0.958 | 0.833 | 0.792 | 0.909 | 0.913 |
| 27-616 | 0.429 | 0.417 | 0.409 | 0.083 | 0.550 | 0.042 | 0.174 | 0.100 | 0.000 | 0.000 | 0.682 | 0.792 | 0.667 | 0.652 | 0.650 | 0.381 | 0.667 | 0.833 | 0.792 | 0.500 | 0.818 | 0.739 |
| 27-624 | 0.000 | 0.000 | 0.000 | 0.000 | 0.000 | 0.000 | 0.000 | 0.000 | 0.000 | 0.000 | 0.000 | 0.000 | 0.000 | 0.000 | 0.000 | 0.095 | 0.000 | 0.167 | 0.000 | 0.000 | 0.000 | 0.000 |
| 27-630 | 0.000 | 0.000 | 0.091 | 0.000 | 0.000 | 0.000 | 0.000 | 0.000 | 0.000 | 0.000 | 0.045 | 0.000 | 0.000 | 0.000 | 0.000 | 0.000 | 0.000 | 0.000 | 0.000 | 0.000 | 0.000 | 0.000 |
| 27-637 | 0.000 | 0.000 | 0.091 | 0.000 | 0.000 | 0.000 | 0.000 | 0.000 | 0.000 | 0.000 | 0.000 | 0.083 | 0.042 | 0.087 | 0.050 | 0.000 | 0.000 | 0.000 | 0.042 | 0.042 | 0.000 | 0.000 |
| 27-656 | 0.000 | 0.000 | 0.000 | 0.000 | 0.000 | 0.000 | 0.000 | 0.000 | 0.000 | 0.000 | 0.000 | 0.000 | 0.042 | 0.000 | 0.000 | 0.000 | 0.000 | 0.000 | 0.000 | 0.000 | 0.000 | 0.000 |
| 27-670 | 0.000 | 0.000 | 0.000 | 0.000 | 0.000 | 0.000 | 0.000 | 0.000 | 0.000 | 0.000 | 0.000 | 0.000 | 0.000 | 0.000 | 0.000 | 0.048 | 0.000 | 0.000 | 0.000 | 0.000 | 0.000 | 0.000 |
| 27-675 | 0.000 | 0.000 | 0.136 | 0.000 | 0.000 | 0.000 | 0.000 | 0.000 | 0.000 | 0.000 | 0.045 | 0.000 | 0.083 | 0.000 | 0.000 | 0.095 | 0.000 | 0.042 | 0.083 | 0.000 | 0.000 | 0.000 |
| 27-681 | 0.000 | 0.000 | 0.091 | 0.000 | 0.000 | 0.000 | 0.000 | 0.000 | 0.000 | 0.000 | 0.045 | 0.000 | 0.167 | 0.000 | 0.000 | 0.048 | 0.000 | 0.000 | 0.000 | 0.000 | 0.000 | 0.000 |
| 27-689 | 0.048 | 0.000 | 0.000 | 0.000 | 0.150 | 0.000 | 0.000 | 0.000 | 0.000 | 0.000 | 0.091 | 0.417 | 0.042 | 0.304 | 0.100 | 0.190 | 0.458 | 0.000 | 0.250 | 0.042 | 0.227 | 0.217 |
| 27-695 | 0.048 | 0.042 | 0.000 | 0.000 | 0.100 | 0.000 | 0.000 | 0.000 | 0.000 | 0.000 | 0.136 | 0.500 | 0.042 | 0.217 | 0.000 | 0.238 | 0.417 | 0.000 | 0.208 | 0.042 | 0.182 | 0.130 |
| 27-702 | 0.000 | 0.000 | 0.000 | 0.000 | 0.000 | 0.000 | 0.000 | 0.000 | 0.000 | 0.000 | 0.000 | 0.000 | 0.000 | 0.000 | 0.000 | 0.000 | 0.042 | 0.000 | 0.000 | 0.000 | 0.045 | 0.000 |
| 27-708 | 0.000 | 0.000 | 0.000 | 0.000 | 0.000 | 0.000 | 0.000 | 0.000 | 0.000 | 0.000 | 0.000 | 0.000 | 0.042 | 0.087 | 0.100 | 0.048 | 0.042 | 0.000 | 0.000 | 0.000 | 0.045 | 0.000 |
| 27-712 | 0.048 | 0.000 | 0.000 | 0.000 | 0.000 | 0.000 | 0.000 | 0.000 | 0.000 | 0.000 | 0.000 | 0.000 | 0.042 | 0.043 | 0.050 | 0.048 | 0.083 | 0.000 | 0.000 | 0.000 | 0.045 | 0.000 |
| 27-717 | 0.000 | 0.000 | 0.000 | 0.000 | 0.000 | 0.000 | 0.000 | 0.000 | 0.000 | 0.000 | 0.000 | 0.000 | 0.000 | 0.043 | 0.000 | 0.000 | 0.000 | 0.083 | 0.000 | 0.000 | 0.136 | 0.000 |
| 27-721 | 0.000 | 0.000 | 0.045 | 0.000 | 0.000 | 0.000 | 0.000 | 0.000 | 0.000 | 0.000 | 0.045 | 0.000 | 0.000 | 0.087 | 0.050 | 0.000 | 0.125 | 0.083 | 0.000 | 0.000 | 0.273 | 0.043 |
| 27-728 | 0.000 | 0.000 | 0.000 | 0.000 | 0.000 | 0.000 | 0.000 | 0.000 | 0.000 | 0.000 | 0.000 | 0.000 | 0.000 | 0.043 | 0.050 | 0.000 | 0.000 | 0.000 | 0.000 | 0.000 | 0.000 | 0.000 |
| 27-735 | 0.000 | 0.000 | 0.000 | 0.000 | 0.000 | 0.000 | 0.000 | 0.000 | 0.000 | 0.000 | 0.000 | 0.042 | 0.000 | 0.000 | 0.050 | 0.095 | 0.208 | 0.083 | 0.042 | 0.000 | 0.045 | 0.000 |
| 27-742 | 0.000 | 0.000 | 0.000 | 0.000 | 0.000 | 0.000 | 0.000 | 0.000 | 0.000 | 0.000 | 0.000 | 0.000 | 0.042 | 0.000 | 0.000 | 0.000 | 0.125 | 0.000 | 0.000 | 0.000 | 0.000 | 0.000 |
| 27-750 | 0.571 | 0.042 | 0.364 | 0.000 | 0.150 | 0.000 | 0.000 | 0.000 | 0.000 | 0.000 | 0.000 | 0.417 | 0.458 | 0.435 | 0.350 | 0.381 | 0.583 | 0.167 | 0.042 | 0.250 | 0.636 | 0.130 |
| 27-758 | 0.476 | 0.042 | 0.364 | 0.000 | 0.200 | 0.000 | 0.000 | 0.000 | 0.000 | 0.000 | 0.045 | 0.417 | 0.375 | 0.435 | 0.350 | 0.286 | 0.583 | 0.208 | 0.000 | 0.250 | 0.591 | 0.130 |
| 27-769 | 0.000 | 0.000 | 0.000 | 0.000 | 0.000 | 0.000 | 0.000 | 0.000 | 0.000 | 0.000 | 0.000 | 0.000 | 0.000 | 0.000 | 0.000 | 0.000 | 0.000 | 0.000 | 0.000 | 0.000 | 0.045 | 0.000 |
| 27-771 | 0.000 | 0.000 | 0.000 | 0.000 | 0.000 | 0.000 | 0.000 | 0.000 | 0.000 | 0.000 | 0.000 | 0.000 | 0.000 | 0.000 | 0.000 | 0.000 | 0.000 | 0.000 | 0.000 | 0.000 | 0.045 | 0.000 |
| 27-784 | 0.000 | 0.000 | 0.000 | 0.000 | 0.000 | 0.000 | 0.000 | 0.000 | 0.000 | 0.000 | 0.000 | 0.000 | 0.042 | 0.000 | 0.000 | 0.000 | 0.000 | 0.000 | 0.000 | 0.000 | 0.136 | 0.000 |
| 27-799 | 0.000 | 0.000 | 0.000 | 0.000 | 0.000 | 0.000 | 0.000 | 0.000 | 0.000 | 0.000 | 0.000 | 0.000 | 0.042 | 0.000 | 0.000 | 0.000 | 0.083 | 0.000 | 0.000 | 0.000 | 0.045 | 0.000 |
| 27-808 | 0.048 | 0.000 | 0.000 | 0.000 | 0.000 | 0.000 | 0.000 | 0.000 | 0.000 | 0.000 | 0.000 | 0.000 | 0.042 | 0.000 | 0.000 | 0.048 | 0.167 | 0.000 | 0.000 | 0.000 | 0.045 | 0.000 |
| 27-812 | 0.000 | 0.000 | 0.000 | 0.000 | 0.000 | 0.000 | 0.000 | 0.000 | 0.000 | 0.000 | 0.000 | 0.000 | 0.000 | 0.000 | 0.000 | 0.000 | 0.000 | 0.042 | 0.000 | 0.000 | 0.000 | 0.000 |
| 27-823 | 0.000 | 0.000 | 0.000 | 0.000 | 0.000 | 0.000 | 0.000 | 0.000 | 0.000 | 0.000 | 0.136 | 0.000 | 0.000 | 0.000 | 0.000 | 0.000 | 0.042 | 0.000 | 0.000 | 0.000 | 0.000 | 0.000 |
| 27-829 | 0.000 | 0.000 | 0.000 | 0.000 | 0.000 | 0.000 | 0.000 | 0.000 | 0.000 | 0.000 | 0.091 | 0.000 | 0.000 | 0.000 | 0.000 | 0.048 | 0.125 | 0.000 | 0.000 | 0.000 | 0.045 | 0.043 |
| 27-836 | 0.048 | 0.000 | 0.000 | 0.000 | 0.050 | 0.000 | 0.000 | 0.000 | 0.000 | 0.000 | 0.182 | 0.000 | 0.042 | 0.000 | 0.000 | 0.095 | 0.208 | 0.000 | 0.000 | 0.000 | 0.091 | 0.043 |
| 27-841 | 0.000 | 0.000 | 0.000 | 0.000 | 0.050 | 0.042 | 0.000 | 0.000 | 0.000 | 0.000 | 0.000 | 0.000 | 0.042 | 0.000 | 0.050 | 0.095 | 0.000 | 0.000 | 0.000 | 0.000 | 0.000 | 0.000 |
| 27-849 | 0.000 | 0.000 | 0.000 | 0.000 | 0.000 | 0.042 | 0.000 | 0.000 | 0.000 | 0.000 | 0.000 | 0.000 | 0.042 | 0.000 | 0.000 | 0.048 | 0.042 | 0.000 | 0.000 | 0.000 | 0.000 | 0.000 |
| 27-853 | 0.000 | 0.000 | 0.000 | 0.000 | 0.000 | 0.000 | 0.000 | 0.000 | 0.000 | 0.000 | 0.045 | 0.000 | 0.000 | 0.000 | 0.000 | 0.000 | 0.000 | 0.000 | 0.000 | 0.000 | 0.000 | 0.000 |
| 27-859 | 0.000 | 0.000 | 0.000 | 0.000 | 0.000 | 0.000 | 0.000 | 0.000 | 0.000 | 0.000 | 0.045 | 0.000 | 0.000 | 0.000 | 0.000 | 0.000 | 0.000 | 0.000 | 0.000 | 0.083 | 0.045 | 0.000 |
| 27-870 | 0.000 | 0.083 | 0.045 | 0.000 | 0.000 | 0.000 | 0.000 | 0.000 | 0.000 | 0.000 | 0.045 | 0.000 | 0.000 | 0.000 | 0.000 | 0.095 | 0.417 | 0.042 | 0.000 | 0.042 | 0.136 | 0.000 |
| 27-875 | 0.095 | 0.000 | 0.000 | 0.000 | 0.000 | 0.000 | 0.000 | 0.000 | 0.000 | 0.000 | 0.045 | 0.000 | 0.000 | 0.000 | 0.000 | 0.000 | 0.000 | 0.000 | 0.000 | 0.000 | 0.000 | 0.000 |
| 27-880 | 0.000 | 0.042 | 0.045 | 0.000 | 0.000 | 0.000 | 0.000 | 0.000 | 0.000 | 0.000 | 0.000 | 0.000 | 0.000 | 0.000 | 0.000 | 0.048 | 0.333 | 0.000 | 0.000 | 0.000 | 0.136 | 0.000 |
| 27-885 | 0.000 | 0.000 | 0.000 | 0.000 | 0.000 | 0.000 | 0.000 | 0.000 | 0.000 | 0.000 | 0.045 | 0.000 | 0.042 | 0.000 | 0.050 | 0.000 | 0.042 | 0.000 | 0.000 | 0.000 | 0.000 | 0.000 |
| 27-891 | 0.000 | 0.000 | 0.000 | 0.000 | 0.000 | 0.000 | 0.000 | 0.000 | 0.000 | 0.000 | 0.000 | 0.083 | 0.042 | 0.130 | 0.000 | 0.095 | 0.167 | 0.042 | 0.000 | 0.042 | 0.045 | 0.087 |
| 27-900 | 0.000 | 0.000 | 0.000 | 0.000 | 0.000 | 0.000 | 0.000 | 0.000 | 0.000 | 0.000 | 0.091 | 0.083 | 0.083 | 0.087 | 0.000 | 0.095 | 0.125 | 0.083 | 0.000 | 0.000 | 0.045 | 0.043 |
| 27-954 | 0.000 | 0.000 | 0.000 | 0.000 | 0.000 | 0.000 | 0.000 | 0.000 | 0.000 | 0.000 | 0.000 | 0.000 | 0.042 | 0.000 | 0.000 | 0.048 | 0.000 | 0.000 | 0.000 | 0.000 | 0.000 | 0.000 |
| 27-977 | 0.000 | 0.000 | 0.045 | 0.000 | 0.000 | 0.000 | 0.000 | 0.000 | 0.000 | 0.000 | 0.000 | 0.000 | 0.000 | 0.000 | 0.000 | 0.000 | 0.000 | 0.000 | 0.000 | 0.000 | 0.000 | 0.000 |
| 27-988 | 0.000 | 0.000 | 0.000 | 0.000 | 0.000 | 0.000 | 0.000 | 0.000 | 0.000 | 0.000 | 0.091 | 0.000 | 0.000 | 0.000 | 0.000 | 0.000 | 0.000 | 0.000 | 0.000 | 0.000 | 0.000 | 0.000 |
| 27-992 | 0.000 | 0.000 | 0.000 | 0.000 | 0.000 | 0.000 | 0.000 | 0.000 | 0.000 | 0.000 | 0.045 | 0.000 | 0.000 | 0.000 | 0.000 | 0.000 | 0.000 | 0.000 | 0.000 | 0.000 | 0.000 | 0.000 |
| 27-998 | 0.000 | 0.000 | 0.000 | 0.000 | 0.000 | 0.000 | 0.000 | 0.000 | 0.000 | 0.000 | 0.091 | 0.000 | 0.000 | 0.000 | 0.000 | 0.000 | 0.000 | 0.000 | 0.000 | 0.000 | 0.000 | 0.000 |
| 27-1005 | 0.000 | 0.000 | 0.000 | 0.000 | 0.000 | 0.000 | 0.000 | 0.000 | 0.000 | 0.000 | 0.136 | 0.000 | 0.000 | 0.043 | 0.000 | 0.143 | 0.000 | 0.000 | 0.000 | 0.000 | 0.045 | 0.000 |
| 27-1011 | 0.000 | 0.000 | 0.000 | 0.000 | 0.000 | 0.000 | 0.000 | 0.000 | 0.000 | 0.000 | 0.136 | 0.000 | 0.000 | 0.000 | 0.050 | 0.000 | 0.000 | 0.000 | 0.000 | 0.000 | 0.000 | 0.000 |
| 27-1018 | 0.000 | 0.000 | 0.000 | 0.000 | 0.000 | 0.000 | 0.000 | 0.000 | 0.067 | 0.000 | 0.045 | 0.000 | 0.000 | 0.000 | 0.000 | 0.000 | 0.042 | 0.000 | 0.000 | 0.000 | 0.000 | 0.000 |
| 27-1024 | 0.429 | 0.000 | 0.000 | 0.000 | 0.050 | 0.000 | 0.000 | 0.000 | 0.000 | 0.000 | 0.000 | 0.000 | 0.292 | 0.000 | 0.000 | 0.286 | 0.333 | 0.000 | 0.000 | 0.083 | 0.045 | 0.000 |
| 27-1036 | 0.000 | 0.000 | 0.000 | 0.000 | 0.000 | 0.000 | 0.000 | 0.000 | 0.000 | 0.000 | 0.000 | 0.000 | 0.000 | 0.043 | 0.100 | 0.000 | 0.000 | 0.000 | 0.000 | 0.000 | 0.000 | 0.000 |
| 27-1047 | 0.000 | 0.000 | 0.000 | 0.000 | 0.000 | 0.000 | 0.000 | 0.000 | 0.000 | 0.000 | 0.045 | 0.042 | 0.042 | 0.000 | 0.050 | 0.000 | 0.042 | 0.000 | 0.042 | 0.000 | 0.045 | 0.000 |
| 27-1052 | 0.000 | 0.000 | 0.000 | 0.000 | 0.000 | 0.000 | 0.000 | 0.050 | 0.000 | 0.000 | 0.045 | 0.000 | 0.000 | 0.000 | 0.000 | 0.000 | 0.042 | 0.000 | 0.042 | 0.000 | 0.045 | 0.000 |
| 27-1084 | 0.000 | 0.000 | 0.000 | 0.000 | 0.000 | 0.000 | 0.000 | 0.000 | 0.000 | 0.000 | 0.000 | 0.000 | 0.000 | 0.000 | 0.000 | 0.048 | 0.000 | 0.000 | 0.000 | 0.000 | 0.000 | 0.000 |
| 27-1092 | 0.000 | 0.000 | 0.045 | 0.000 | 0.000 | 0.000 | 0.000 | 0.000 | 0.000 | 0.000 | 0.000 | 0.000 | 0.000 | 0.000 | 0.000 | 0.048 | 0.000 | 0.000 | 0.000 | 0.000 | 0.000 | 0.000 |
| 30-149 | 0.000 | 0.000 | 0.000 | 0.000 | 0.000 | 0.000 | 0.000 | 0.105 | 0.000 | 0.000 | 0.000 | 0.000 | 0.000 | 0.000 | 0.000 | 0.000 | 0.000 | 0.000 | 0.000 | 0.000 | 0.000 | 0.000 |
| 30-153 | 0.000 | 0.000 | 0.000 | 0.000 | 0.000 | 0.000 | 0.000 | 0.105 | 0.000 | 0.000 | 0.000 | 0.000 | 0.000 | 0.000 | 0.000 | 0.000 | 0.000 | 0.000 | 0.000 | 0.000 | 0.000 | 0.000 |
| 30-156 | 0.000 | 0.000 | 0.000 | 0.000 | 0.000 | 0.000 | 0.000 | 0.105 | 0.000 | 0.000 | 0.000 | 0.000 | 0.000 | 0.000 | 0.000 | 0.000 | 0.000 | 0.000 | 0.000 | 0.000 | 0.000 | 0.000 |
| 30-162 | 0.000 | 0.000 | 0.000 | 0.000 | 0.000 | 0.000 | 0.000 | 0.053 | 0.000 | 0.000 | 0.000 | 0.000 | 0.000 | 0.000 | 0.000 | 0.000 | 0.000 | 0.043 | 0.000 | 0.042 | 0.000 | 0.130 |
| 30-180 | 0.000 | 0.167 | 0.174 | 0.583 | 0.000 | 0.043 | 0.609 | 0.158 | 0.364 | 0.333 | 0.043 | 0.000 | 0.000 | 0.000 | 0.000 | 0.048 | 0.000 | 0.000 | 0.000 | 0.042 | 0.000 | 0.130 |
| 30-186 | 0.000 | 0.000 | 0.000 | 0.000 | 0.000 | 0.000 | 0.000 | 0.105 | 0.000 | 0.000 | 0.000 | 0.000 | 0.000 | 0.000 | 0.000 | 0.048 | 0.000 | 0.000 | 0.000 | 0.000 | 0.000 | 0.000 |
| 30-192 | 0.000 | 0.000 | 0.000 | 0.000 | 0.000 | 0.043 | 0.000 | 0.000 | 0.000 | 0.000 | 0.000 | 0.000 | 0.000 | 0.000 | 0.000 | 0.000 | 0.000 | 0.000 | 0.000 | 0.000 | 0.000 | 0.000 |
| 30-202 | 0.000 | 0.000 | 0.000 | 0.000 | 0.000 | 0.000 | 0.000 | 0.158 | 0.000 | 0.000 | 0.000 | 0.000 | 0.000 | 0.000 | 0.000 | 0.000 | 0.000 | 0.000 | 0.000 | 0.000 | 0.000 | 0.000 |
| 30-206 | 0.000 | 0.000 | 0.000 | 0.000 | 0.000 | 0.000 | 0.043 | 0.105 | 0.000 | 0.000 | 0.000 | 0.000 | 0.000 | 0.000 | 0.000 | 0.000 | 0.000 | 0.000 | 0.000 | 0.000 | 0.000 | 0.000 |
| 30-213 | 0.048 | 0.000 | 0.043 | 0.042 | 0.000 | 0.000 | 0.000 | 0.211 | 0.091 | 0.000 | 0.000 | 0.000 | 0.000 | 0.067 | 0.000 | 0.000 | 0.042 | 0.043 | 0.095 | 0.000 | 0.000 | 0.043 |
| 30-230 | 0.000 | 0.000 | 0.087 | 0.125 | 0.000 | 0.043 | 0.087 | 0.105 | 0.182 | 0.111 | 0.043 | 0.053 | 0.000 | 0.000 | 0.053 | 0.000 | 0.250 | 0.043 | 0.000 | 0.042 | 0.000 | 0.000 |
| 30-238 | 0.000 | 0.083 | 0.000 | 0.083 | 0.000 | 0.130 | 0.043 | 0.053 | 0.000 | 0.000 | 0.000 | 0.000 | 0.000 | 0.000 | 0.000 | 0.143 | 0.167 | 0.043 | 0.000 | 0.167 | 0.000 | 0.522 |
| 30-246 | 0.000 | 0.000 | 0.043 | 0.000 | 0.000 | 0.000 | 0.000 | 0.000 | 0.000 | 0.000 | 0.000 | 0.000 | 0.000 | 0.000 | 0.000 | 0.000 | 0.000 | 0.000 | 0.000 | 0.000 | 0.000 | 0.000 |
| 30-258 | 0.000 | 0.000 | 0.000 | 0.000 | 0.067 | 0.000 | 0.043 | 0.000 | 0.000 | 0.000 | 0.000 | 0.000 | 0.000 | 0.000 | 0.000 | 0.000 | 0.000 | 0.000 | 0.000 | 0.000 | 0.000 | 0.000 |
| 30-275 | 0.000 | 0.000 | 0.043 | 0.000 | 0.000 | 0.000 | 0.000 | 0.158 | 0.000 | 0.000 | 0.000 | 0.000 | 0.048 | 0.000 | 0.000 | 0.048 | 0.000 | 0.000 | 0.000 | 0.000 | 0.000 | 0.000 |
| 30-281 | 0.000 | 0.000 | 0.000 | 0.000 | 0.000 | 0.000 | 0.000 | 0.053 | 0.000 | 0.056 | 0.000 | 0.000 | 0.000 | 0.000 | 0.000 | 0.000 | 0.000 | 0.000 | 0.000 | 0.000 | 0.000 | 0.000 |
| 30-288 | 0.095 | 0.042 | 0.000 | 0.000 | 0.000 | 0.043 | 0.043 | 0.211 | 0.000 | 0.000 | 0.000 | 0.000 | 0.000 | 0.000 | 0.000 | 0.000 | 0.042 | 0.043 | 0.000 | 0.000 | 0.000 | 0.000 |
| 30-297 | 0.000 | 0.000 | 0.000 | 0.000 | 0.000 | 0.000 | 0.000 | 0.053 | 0.000 | 0.000 | 0.000 | 0.000 | 0.000 | 0.000 | 0.000 | 0.048 | 0.000 | 0.000 | 0.000 | 0.000 | 0.000 | 0.000 |
| 30-302 | 0.000 | 0.000 | 0.000 | 0.000 | 0.000 | 0.000 | 0.000 | 0.000 | 0.000 | 0.000 | 0.000 | 0.000 | 0.000 | 0.000 | 0.000 | 0.000 | 0.000 | 0.000 | 0.000 | 0.042 | 0.000 | 0.043 |
| 30-306 | 0.429 | 0.542 | 0.783 | 0.500 | 0.333 | 0.391 | 0.652 | 0.579 | 0.455 | 0.111 | 0.696 | 0.105 | 0.095 | 0.533 | 0.526 | 0.857 | 0.292 | 0.174 | 0.190 | 0.875 | 0.188 | 0.696 |
| 30-310 | 0.048 | 0.083 | 0.000 | 0.000 | 0.000 | 0.000 | 0.174 | 0.053 | 0.000 | 0.000 | 0.000 | 0.000 | 0.000 | 0.000 | 0.000 | 0.095 | 0.000 | 0.000 | 0.000 | 0.083 | 0.000 | 0.000 |
| 30-316 | 0.000 | 0.000 | 0.000 | 0.042 | 0.000 | 0.000 | 0.000 | 0.000 | 0.000 | 0.000 | 0.000 | 0.000 | 0.000 | 0.000 | 0.000 | 0.048 | 0.083 | 0.000 | 0.000 | 0.083 | 0.000 | 0.000 |
| 30-323 | 0.000 | 0.000 | 0.000 | 0.042 | 0.000 | 0.000 | 0.000 | 0.158 | 0.000 | 0.056 | 0.000 | 0.000 | 0.000 | 0.000 | 0.000 | 0.048 | 0.083 | 0.000 | 0.000 | 0.083 | 0.000 | 0.348 |
| 30-328 | 0.857 | 0.125 | 0.217 | 0.208 | 0.467 | 0.565 | 0.130 | 0.474 | 0.455 | 0.278 | 0.130 | 0.526 | 0.905 | 0.400 | 0.474 | 0.667 | 0.917 | 0.957 | 0.381 | 0.750 | 0.813 | 0.261 |
| 30-333 | 0.952 | 0.042 | 0.304 | 0.167 | 0.267 | 0.565 | 0.217 | 0.474 | 0.455 | 0.278 | 0.043 | 0.474 | 0.952 | 0.467 | 0.579 | 0.619 | 1.000 | 0.957 | 0.333 | 0.792 | 0.813 | 0.261 |
| 30-350 | 0.000 | 0.000 | 0.000 | 0.000 | 0.000 | 0.000 | 0.000 | 0.053 | 0.000 | 0.000 | 0.000 | 0.000 | 0.000 | 0.000 | 0.000 | 0.000 | 0.000 | 0.000 | 0.000 | 0.000 | 0.125 | 0.000 |
| 30-354 | 0.381 | 0.417 | 0.435 | 0.417 | 0.067 | 0.130 | 0.217 | 0.316 | 0.545 | 0.167 | 0.130 | 0.526 | 0.048 | 0.667 | 0.263 | 0.667 | 0.375 | 0.261 | 0.190 | 0.583 | 0.188 | 0.565 |
| 30-358 | 0.429 | 0.167 | 0.435 | 0.375 | 0.000 | 0.130 | 0.217 | 0.263 | 0.455 | 0.167 | 0.043 | 0.316 | 0.048 | 0.533 | 0.316 | 0.571 | 0.333 | 0.261 | 0.143 | 0.417 | 0.063 | 0.478 |
| 30-365 | 0.048 | 0.042 | 0.043 | 0.042 | 0.000 | 0.000 | 0.087 | 0.000 | 0.000 | 0.000 | 0.000 | 0.000 | 0.048 | 0.000 | 0.000 | 0.000 | 0.000 | 0.000 | 0.000 | 0.000 | 0.000 | 0.043 |
| 30-371 | 0.000 | 0.000 | 0.130 | 0.000 | 0.000 | 0.000 | 0.000 | 0.000 | 0.000 | 0.000 | 0.000 | 0.000 | 0.000 | 0.000 | 0.000 | 0.000 | 0.000 | 0.000 | 0.000 | 0.000 | 0.000 | 0.000 |
| 30-377 | 0.000 | 0.000 | 0.174 | 0.042 | 0.000 | 0.000 | 0.043 | 0.000 | 0.045 | 0.000 | 0.043 | 0.000 | 0.000 | 0.133 | 0.053 | 0.000 | 0.000 | 0.000 | 0.095 | 0.000 | 0.063 | 0.000 |
| 30-387 | 0.000 | 0.000 | 0.087 | 0.125 | 0.000 | 0.000 | 0.043 | 0.000 | 0.000 | 0.056 | 0.000 | 0.000 | 0.000 | 0.000 | 0.000 | 0.000 | 0.042 | 0.000 | 0.000 | 0.000 | 0.000 | 0.000 |
| 30-401 | 0.190 | 0.000 | 0.000 | 0.000 | 0.000 | 0.217 | 0.087 | 0.263 | 0.000 | 0.000 | 0.000 | 0.000 | 0.286 | 0.000 | 0.000 | 0.000 | 0.292 | 0.130 | 0.000 | 0.000 | 0.063 | 0.087 |
| 30-407 | 0.000 | 0.000 | 0.043 | 0.000 | 0.000 | 0.000 | 0.087 | 0.000 | 0.000 | 0.000 | 0.000 | 0.000 | 0.000 | 0.000 | 0.000 | 0.000 | 0.042 | 0.000 | 0.048 | 0.000 | 0.000 | 0.000 |
| 30-410 | 0.000 | 0.000 | 0.043 | 0.000 | 0.000 | 0.000 | 0.000 | 0.105 | 0.000 | 0.000 | 0.000 | 0.000 | 0.000 | 0.000 | 0.000 | 0.000 | 0.000 | 0.000 | 0.000 | 0.000 | 0.000 | 0.000 |
| 30-433 | 0.000 | 0.000 | 0.174 | 0.042 | 0.000 | 0.000 | 0.043 | 0.000 | 0.091 | 0.000 | 0.000 | 0.000 | 0.000 | 0.000 | 0.053 | 0.000 | 0.000 | 0.000 | 0.000 | 0.000 | 0.000 | 0.000 |
| 30-436 | 0.000 | 0.000 | 0.043 | 0.000 | 0.000 | 0.000 | 0.000 | 0.000 | 0.000 | 0.000 | 0.000 | 0.000 | 0.000 | 0.000 | 0.000 | 0.000 | 0.000 | 0.000 | 0.000 | 0.000 | 0.000 | 0.000 |
| 30-439 | 0.000 | 0.000 | 0.043 | 0.000 | 0.000 | 0.000 | 0.000 | 0.000 | 0.000 | 0.000 | 0.000 | 0.000 | 0.000 | 0.000 | 0.000 | 0.000 | 0.000 | 0.000 | 0.000 | 0.000 | 0.000 | 0.000 |
| 30-450 | 0.000 | 0.083 | 0.000 | 0.000 | 0.000 | 0.000 | 0.000 | 0.053 | 0.000 | 0.000 | 0.043 | 0.000 | 0.048 | 0.133 | 0.105 | 0.000 | 0.000 | 0.000 | 0.000 | 0.000 | 0.000 | 0.000 |
| 30-455 | 0.000 | 0.000 | 0.391 | 0.083 | 0.000 | 0.000 | 0.087 | 0.000 | 0.000 | 0.000 | 0.000 | 0.000 | 0.000 | 0.000 | 0.000 | 0.048 | 0.000 | 0.000 | 0.000 | 0.000 | 0.000 | 0.000 |
| 30-475 | 0.048 | 0.333 | 0.696 | 0.417 | 0.000 | 0.130 | 0.565 | 0.421 | 0.727 | 0.167 | 0.478 | 0.158 | 0.000 | 0.467 | 0.421 | 0.143 | 0.000 | 0.043 | 0.476 | 0.083 | 0.063 | 0.217 |
| 30-480 | 0.476 | 0.708 | 0.478 | 0.958 | 0.000 | 0.130 | 0.870 | 0.632 | 0.818 | 0.222 | 0.348 | 0.632 | 0.095 | 0.667 | 0.579 | 0.714 | 0.458 | 0.304 | 0.571 | 0.875 | 0.063 | 0.652 |
| 30-483 | 0.286 | 0.458 | 0.783 | 0.667 | 0.000 | 0.043 | 0.478 | 0.684 | 0.818 | 0.222 | 0.304 | 0.526 | 0.048 | 0.533 | 0.579 | 0.524 | 0.250 | 0.217 | 0.190 | 0.292 | 0.000 | 0.087 |
| 30-490 | 0.095 | 0.708 | 0.565 | 0.708 | 0.000 | 0.174 | 0.696 | 0.158 | 0.545 | 0.111 | 0.261 | 0.000 | 0.000 | 0.000 | 0.263 | 0.286 | 0.042 | 0.174 | 0.238 | 0.667 | 0.063 | 0.435 |
| 30-496 | 0.000 | 0.000 | 0.000 | 0.000 | 0.000 | 0.000 | 0.000 | 0.000 | 0.045 | 0.000 | 0.000 | 0.000 | 0.000 | 0.067 | 0.000 | 0.000 | 0.042 | 0.000 | 0.000 | 0.000 | 0.000 | 0.000 |
| 30-500 | 0.429 | 0.042 | 0.261 | 0.583 | 0.000 | 0.174 | 0.174 | 0.368 | 0.318 | 0.111 | 0.043 | 0.211 | 0.190 | 0.000 | 0.000 | 0.619 | 0.458 | 0.304 | 0.095 | 0.750 | 0.125 | 0.652 |
| 30-508 | 0.381 | 0.042 | 0.174 | 0.417 | 0.000 | 0.174 | 0.043 | 0.368 | 0.273 | 0.111 | 0.043 | 0.263 | 0.190 | 0.067 | 0.000 | 0.667 | 0.417 | 0.348 | 0.000 | 0.625 | 0.438 | 0.609 |
| 30-525 | 0.000 | 0.000 | 0.000 | 0.000 | 0.000 | 0.000 | 0.000 | 0.158 | 0.000 | 0.000 | 0.000 | 0.000 | 0.000 | 0.000 | 0.053 | 0.048 | 0.000 | 0.043 | 0.000 | 0.000 | 0.000 | 0.000 |
| 30-528 | 0.000 | 0.000 | 0.087 | 0.000 | 0.000 | 0.000 | 0.000 | 0.158 | 0.000 | 0.000 | 0.000 | 0.000 | 0.000 | 0.000 | 0.000 | 0.000 | 0.000 | 0.000 | 0.000 | 0.000 | 0.000 | 0.000 |
| 30-532 | 0.095 | 0.000 | 0.000 | 0.042 | 0.000 | 0.000 | 0.130 | 0.053 | 0.045 | 0.000 | 0.000 | 0.000 | 0.000 | 0.000 | 0.053 | 0.286 | 0.083 | 0.130 | 0.000 | 0.167 | 0.063 | 0.000 |
| 30-538 | 0.286 | 0.000 | 0.130 | 0.042 | 0.000 | 0.043 | 0.043 | 0.000 | 0.091 | 0.000 | 0.000 | 0.000 | 0.000 | 0.067 | 0.000 | 0.190 | 0.125 | 0.174 | 0.048 | 0.083 | 0.063 | 0.000 |
| 30-547 | 0.000 | 0.000 | 0.304 | 0.000 | 0.000 | 0.000 | 0.000 | 0.053 | 0.000 | 0.000 | 0.000 | 0.053 | 0.190 | 0.067 | 0.000 | 0.000 | 0.000 | 0.000 | 0.000 | 0.000 | 0.000 | 0.000 |
| 30-556 | 0.000 | 0.000 | 0.043 | 0.000 | 0.000 | 0.000 | 0.000 | 0.000 | 0.000 | 0.000 | 0.000 | 0.000 | 0.000 | 0.000 | 0.000 | 0.000 | 0.000 | 0.000 | 0.000 | 0.042 | 0.000 | 0.000 |
| 30-560 | 0.000 | 0.000 | 0.130 | 0.000 | 0.000 | 0.000 | 0.000 | 0.000 | 0.000 | 0.000 | 0.000 | 0.000 | 0.048 | 0.000 | 0.000 | 0.000 | 0.000 | 0.000 | 0.000 | 0.000 | 0.000 | 0.000 |
| 30-566 | 0.000 | 0.042 | 0.000 | 0.000 | 0.000 | 0.043 | 0.000 | 0.000 | 0.000 | 0.000 | 0.000 | 0.000 | 0.000 | 0.000 | 0.000 | 0.143 | 0.000 | 0.000 | 0.000 | 0.333 | 0.000 | 0.348 |
| 30-584 | 0.048 | 0.000 | 0.000 | 0.000 | 0.000 | 0.000 | 0.000 | 0.053 | 0.045 | 0.000 | 0.000 | 0.000 | 0.000 | 0.000 | 0.000 | 0.048 | 0.125 | 0.000 | 0.000 | 0.125 | 0.000 | 0.000 |
| 30-591 | 0.095 | 0.000 | 0.043 | 0.000 | 0.000 | 0.000 | 0.043 | 0.053 | 0.000 | 0.000 | 0.000 | 0.000 | 0.000 | 0.000 | 0.000 | 0.000 | 0.083 | 0.000 | 0.000 | 0.042 | 0.000 | 0.043 |
| 30-598 | 0.000 | 0.000 | 0.043 | 0.000 | 0.000 | 0.000 | 0.000 | 0.053 | 0.000 | 0.000 | 0.000 | 0.000 | 0.000 | 0.000 | 0.000 | 0.000 | 0.000 | 0.000 | 0.000 | 0.000 | 0.000 | 0.000 |
| 30-603 | 0.000 | 0.042 | 0.000 | 0.000 | 0.000 | 0.000 | 0.000 | 0.000 | 0.000 | 0.000 | 0.000 | 0.000 | 0.000 | 0.067 | 0.000 | 0.000 | 0.000 | 0.000 | 0.000 | 0.000 | 0.000 | 0.000 |
| 30-625 | 0.000 | 0.000 | 0.000 | 0.000 | 0.000 | 0.000 | 0.000 | 0.000 | 0.000 | 0.000 | 0.000 | 0.000 | 0.000 | 0.000 | 0.000 | 0.048 | 0.000 | 0.000 | 0.000 | 0.000 | 0.000 | 0.000 |
| 30-632 | 0.143 | 0.000 | 0.043 | 0.333 | 0.000 | 0.043 | 0.043 | 0.105 | 0.227 | 0.111 | 0.130 | 0.105 | 0.000 | 0.067 | 0.105 | 0.000 | 0.250 | 0.087 | 0.000 | 0.000 | 0.000 | 0.087 |
| 30-640 | 0.143 | 0.000 | 0.000 | 0.125 | 0.000 | 0.043 | 0.043 | 0.105 | 0.182 | 0.056 | 0.000 | 0.000 | 0.000 | 0.000 | 0.000 | 0.000 | 0.250 | 0.000 | 0.000 | 0.000 | 0.000 | 0.000 |
| 30-655 | 0.905 | 0.000 | 0.043 | 0.000 | 0.533 | 0.739 | 0.000 | 0.368 | 0.273 | 0.667 | 0.348 | 0.368 | 0.810 | 0.133 | 0.263 | 0.048 | 0.917 | 0.783 | 0.524 | 0.000 | 0.813 | 0.000 |
| 30-677 | 0.000 | 0.000 | 0.000 | 0.000 | 0.000 | 0.000 | 0.000 | 0.000 | 0.000 | 0.000 | 0.000 | 0.000 | 0.000 | 0.000 | 0.000 | 0.000 | 0.000 | 0.000 | 0.000 | 0.000 | 0.125 | 0.000 |
| 30-684 | 0.000 | 0.000 | 0.000 | 0.000 | 0.000 | 0.000 | 0.043 | 0.053 | 0.000 | 0.000 | 0.000 | 0.000 | 0.048 | 0.000 | 0.000 | 0.000 | 0.000 | 0.000 | 0.000 | 0.000 | 0.000 | 0.000 |
| 30-690 | 0.000 | 0.042 | 0.000 | 0.000 | 0.000 | 0.000 | 0.000 | 0.000 | 0.000 | 0.000 | 0.000 | 0.000 | 0.000 | 0.000 | 0.000 | 0.000 | 0.000 | 0.000 | 0.000 | 0.000 | 0.000 | 0.000 |
| 30-719 | 0.000 | 0.000 | 0.000 | 0.000 | 0.000 | 0.000 | 0.000 | 0.000 | 0.000 | 0.000 | 0.000 | 0.000 | 0.000 | 0.000 | 0.000 | 0.048 | 0.000 | 0.000 | 0.000 | 0.000 | 0.000 | 0.000 |
| 30-743 | 0.000 | 0.000 | 0.000 | 0.000 | 0.000 | 0.000 | 0.043 | 0.000 | 0.000 | 0.000 | 0.000 | 0.000 | 0.000 | 0.000 | 0.000 | 0.000 | 0.000 | 0.000 | 0.000 | 0.000 | 0.000 | 0.000 |
| 30-793 | 0.000 | 0.000 | 0.000 | 0.000 | 0.000 | 0.000 | 0.043 | 0.000 | 0.000 | 0.000 | 0.000 | 0.000 | 0.000 | 0.000 | 0.000 | 0.000 | 0.000 | 0.000 | 0.000 | 0.000 | 0.000 | 0.000 |
| 30-806 | 0.000 | 0.000 | 0.043 | 0.000 | 0.000 | 0.000 | 0.000 | 0.000 | 0.000 | 0.000 | 0.000 | 0.000 | 0.000 | 0.000 | 0.053 | 0.000 | 0.000 | 0.000 | 0.000 | 0.000 | 0.000 | 0.000 |
| 30-809 | 0.000 | 0.000 | 0.043 | 0.000 | 0.000 | 0.000 | 0.000 | 0.000 | 0.000 | 0.000 | 0.000 | 0.000 | 0.000 | 0.000 | 0.053 | 0.000 | 0.000 | 0.000 | 0.000 | 0.000 | 0.000 | 0.000 |
| 30-878 | 0.000 | 0.000 | 0.000 | 0.000 | 0.000 | 0.000 | 0.000 | 0.053 | 0.000 | 0.000 | 0.000 | 0.000 | 0.000 | 0.000 | 0.000 | 0.000 | 0.000 | 0.000 | 0.000 | 0.000 | 0.000 | 0.000 |
| 30-891 | 0.000 | 0.458 | 0.261 | 0.375 | 0.000 | 0.043 | 0.522 | 0.158 | 0.273 | 0.000 | 0.391 | 0.000 | 0.000 | 0.000 | 0.053 | 0.095 | 0.042 | 0.043 | 0.143 | 0.417 | 0.000 | 0.435 |
| 30-896 | 0.048 | 0.500 | 0.391 | 0.583 | 0.000 | 0.130 | 0.696 | 0.053 | 0.045 | 0.111 | 0.478 | 0.000 | 0.000 | 0.000 | 0.000 | 0.048 | 0.042 | 0.000 | 0.048 | 0.292 | 0.000 | 0.435 |
| 30-911 | 0.333 | 0.000 | 0.000 | 0.000 | 0.000 | 0.043 | 0.000 | 0.105 | 0.000 | 0.000 | 0.000 | 0.000 | 0.048 | 0.000 | 0.000 | 0.000 | 0.458 | 0.087 | 0.000 | 0.000 | 0.375 | 0.000 |
| 30-937 | 0.143 | 0.000 | 0.043 | 0.000 | 0.000 | 0.000 | 0.000 | 0.105 | 0.000 | 0.000 | 0.000 | 0.000 | 0.095 | 0.000 | 0.000 | 0.000 | 0.375 | 0.043 | 0.000 | 0.000 | 0.125 | 0.000 |
| 30-974 | 0.048 | 0.000 | 0.000 | 0.000 | 0.000 | 0.000 | 0.000 | 0.105 | 0.000 | 0.000 | 0.000 | 0.000 | 0.000 | 0.000 | 0.000 | 0.000 | 0.000 | 0.000 | 0.000 | 0.000 | 0.000 | 0.000 |
| 30-991 | 0.095 | 0.000 | 0.000 | 0.000 | 0.000 | 0.000 | 0.000 | 0.000 | 0.000 | 0.000 | 0.000 | 0.000 | 0.000 | 0.000 | 0.000 | 0.000 | 0.000 | 0.000 | 0.000 | 0.000 | 0.000 | 0.043 |
| 30-1016 | 0.000 | 0.042 | 0.000 | 0.000 | 0.000 | 0.000 | 0.000 | 0.000 | 0.000 | 0.000 | 0.000 | 0.000 | 0.000 | 0.000 | 0.000 | 0.000 | 0.000 | 0.000 | 0.000 | 0.000 | 0.000 | 0.000 |
| 30-1026 | 0.000 | 0.042 | 0.000 | 0.000 | 0.000 | 0.000 | 0.000 | 0.000 | 0.000 | 0.000 | 0.000 | 0.000 | 0.000 | 0.000 | 0.000 | 0.000 | 0.000 | 0.000 | 0.000 | 0.000 | 0.000 | 0.000 |
| 30-1045 | 0.000 | 0.042 | 0.000 | 0.000 | 0.000 | 0.000 | 0.087 | 0.000 | 0.000 | 0.000 | 0.000 | 0.000 | 0.000 | 0.000 | 0.000 | 0.000 | 0.000 | 0.000 | 0.000 | 0.000 | 0.000 | 0.000 |
| 30-1072 | 0.000 | 0.000 | 0.000 | 0.000 | 0.067 | 0.000 | 0.000 | 0.000 | 0.000 | 0.000 | 0.000 | 0.000 | 0.000 | 0.000 | 0.000 | 0.000 | 0.000 | 0.000 | 0.000 | 0.000 | 0.000 | 0.000 |
| 30-1090 | 0.000 | 0.000 | 0.000 | 0.000 | 0.067 | 0.000 | 0.000 | 0.000 | 0.000 | 0.000 | 0.000 | 0.000 | 0.000 | 0.000 | 0.000 | 0.000 | 0.000 | 0.000 | 0.000 | 0.000 | 0.000 | 0.000 |
| 30-1137 | 0.000 | 0.000 | 0.043 | 0.000 | 0.000 | 0.000 | 0.000 | 0.000 | 0.000 | 0.000 | 0.000 | 0.000 | 0.000 | 0.000 | 0.000 | 0.000 | 0.000 | 0.000 | 0.000 | 0.000 | 0.000 | 0.000 |
| 30-1153 | 0.000 | 0.042 | 0.043 | 0.042 | 0.000 | 0.000 | 0.087 | 0.000 | 0.000 | 0.000 | 0.000 | 0.000 | 0.000 | 0.000 | 0.000 | 0.000 | 0.000 | 0.000 | 0.000 | 0.000 | 0.000 | 0.000 |
| 30-1159 | 0.000 | 0.042 | 0.087 | 0.000 | 0.000 | 0.000 | 0.000 | 0.000 | 0.000 | 0.000 | 0.000 | 0.000 | 0.000 | 0.000 | 0.000 | 0.000 | 0.000 | 0.000 | 0.000 | 0.000 | 0.000 | 0.000 |
| 30-1170 | 0.000 | 0.000 | 0.043 | 0.000 | 0.000 | 0.000 | 0.000 | 0.000 | 0.000 | 0.000 | 0.000 | 0.000 | 0.000 | 0.000 | 0.000 | 0.000 | 0.000 | 0.000 | 0.000 | 0.000 | 0.000 | 0.000 |
| 30-1177 | 0.000 | 0.125 | 0.000 | 0.042 | 0.000 | 0.000 | 0.043 | 0.000 | 0.045 | 0.000 | 0.000 | 0.000 | 0.000 | 0.000 | 0.000 | 0.000 | 0.000 | 0.000 | 0.000 | 0.000 | 0.000 | 0.000 |
| 30-1183 | 0.000 | 0.042 | 0.043 | 0.208 | 0.000 | 0.000 | 0.043 | 0.053 | 0.045 | 0.056 | 0.043 | 0.000 | 0.000 | 0.000 | 0.000 | 0.000 | 0.000 | 0.000 | 0.000 | 0.000 | 0.000 | 0.000 |
| 30-1191 | 0.000 | 0.042 | 0.000 | 0.042 | 0.000 | 0.000 | 0.043 | 0.000 | 0.000 | 0.000 | 0.000 | 0.000 | 0.000 | 0.000 | 0.000 | 0.000 | 0.000 | 0.000 | 0.000 | 0.000 | 0.000 | 0.000 |
| 30-1197 | 0.000 | 0.042 | 0.087 | 0.167 | 0.000 | 0.000 | 0.130 | 0.000 | 0.000 | 0.056 | 0.261 | 0.000 | 0.000 | 0.000 | 0.000 | 0.000 | 0.000 | 0.000 | 0.000 | 0.000 | 0.000 | 0.000 |
| 31-102 | 0.136 | 0.353 | 0.913 | 0.217 | 0.056 | 0.000 | 0.087 | 0.048 | 0.375 | 0.273 | 0.783 | 0.125 | 0.167 | 0.208 | 0.150 | 0.143 | 0.458 | 0.583 | 0.292 | 0.042 | 0.682 | 0.391 |
| 31-106 | 0.136 | 0.235 | 0.913 | 0.130 | 0.056 | 0.000 | 0.087 | 0.048 | 0.208 | 0.227 | 0.739 | 0.083 | 0.083 | 0.167 | 0.100 | 0.095 | 0.375 | 0.583 | 0.250 | 0.000 | 0.682 | 0.391 |
| 31-122 | 0.000 | 0.000 | 0.043 | 0.000 | 0.056 | 0.000 | 0.000 | 0.000 | 0.042 | 0.000 | 0.000 | 0.042 | 0.000 | 0.000 | 0.000 | 0.048 | 0.000 | 0.000 | 0.000 | 0.000 | 0.000 | 0.043 |
| 31-126 | 0.000 | 0.000 | 0.304 | 0.000 | 0.000 | 0.000 | 0.000 | 0.000 | 0.042 | 0.000 | 0.565 | 0.000 | 0.000 | 0.000 | 0.000 | 0.000 | 0.000 | 0.167 | 0.000 | 0.000 | 0.091 | 0.043 |
| 31-159 | 0.000 | 0.000 | 0.000 | 0.000 | 0.000 | 0.000 | 0.000 | 0.000 | 0.000 | 0.000 | 0.000 | 0.000 | 0.000 | 0.000 | 0.050 | 0.000 | 0.000 | 0.000 | 0.000 | 0.000 | 0.000 | 0.000 |
| 31-167 | 0.000 | 0.059 | 0.000 | 0.000 | 0.000 | 0.000 | 0.000 | 0.000 | 0.000 | 0.000 | 0.043 | 0.000 | 0.000 | 0.000 | 0.000 | 0.000 | 0.000 | 0.000 | 0.000 | 0.000 | 0.000 | 0.000 |
| 31-179 | 0.000 | 0.000 | 0.000 | 0.000 | 0.000 | 0.000 | 0.000 | 0.000 | 0.125 | 0.000 | 0.000 | 0.000 | 0.000 | 0.000 | 0.000 | 0.000 | 0.000 | 0.000 | 0.000 | 0.000 | 0.000 | 0.000 |
| 31-186 | 0.000 | 0.000 | 0.000 | 0.000 | 0.000 | 0.000 | 0.000 | 0.000 | 0.083 | 0.000 | 0.043 | 0.000 | 0.000 | 0.000 | 0.000 | 0.000 | 0.000 | 0.000 | 0.000 | 0.000 | 0.000 | 0.000 |
| 31-193 | 0.000 | 0.000 | 0.000 | 0.000 | 0.000 | 0.000 | 0.000 | 0.000 | 0.042 | 0.045 | 0.043 | 0.000 | 0.042 | 0.000 | 0.000 | 0.000 | 0.000 | 0.000 | 0.000 | 0.000 | 0.000 | 0.043 |
| 31-199 | 0.000 | 0.000 | 0.000 | 0.000 | 0.000 | 0.000 | 0.000 | 0.000 | 0.042 | 0.000 | 0.043 | 0.000 | 0.000 | 0.000 | 0.000 | 0.000 | 0.000 | 0.000 | 0.000 | 0.000 | 0.000 | 0.000 |
| 31-205 | 0.000 | 0.000 | 0.000 | 0.000 | 0.000 | 0.000 | 0.000 | 0.000 | 0.000 | 0.000 | 0.000 | 0.000 | 0.042 | 0.000 | 0.000 | 0.000 | 0.000 | 0.000 | 0.000 | 0.000 | 0.000 | 0.000 |
| 31-209 | 0.000 | 0.059 | 0.000 | 0.000 | 0.000 | 0.000 | 0.000 | 0.000 | 0.000 | 0.000 | 0.000 | 0.000 | 0.083 | 0.000 | 0.100 | 0.000 | 0.000 | 0.000 | 0.000 | 0.125 | 0.000 | 0.043 |
| 31-220 | 0.000 | 0.000 | 0.000 | 0.000 | 0.000 | 0.000 | 0.000 | 0.000 | 0.042 | 0.000 | 0.000 | 0.000 | 0.042 | 0.000 | 0.000 | 0.000 | 0.000 | 0.000 | 0.000 | 0.000 | 0.000 | 0.000 |
| 31-224 | 0.000 | 0.000 | 0.000 | 0.000 | 0.000 | 0.000 | 0.043 | 0.000 | 0.000 | 0.000 | 0.000 | 0.000 | 0.042 | 0.000 | 0.000 | 0.143 | 0.042 | 0.000 | 0.000 | 0.000 | 0.000 | 0.000 |
| 31-231 | 0.000 | 0.000 | 0.000 | 0.000 | 0.000 | 0.000 | 0.000 | 0.000 | 0.042 | 0.045 | 0.000 | 0.000 | 0.000 | 0.000 | 0.000 | 0.000 | 0.000 | 0.000 | 0.000 | 0.000 | 0.000 | 0.000 |
| 31-239 | 0.000 | 0.000 | 0.000 | 0.000 | 0.000 | 0.000 | 0.000 | 0.000 | 0.500 | 0.273 | 0.000 | 0.000 | 0.083 | 0.000 | 0.000 | 0.000 | 0.000 | 0.000 | 0.000 | 0.000 | 0.000 | 0.000 |
| 31-245 | 0.091 | 0.176 | 0.217 | 0.043 | 0.000 | 0.000 | 0.043 | 0.000 | 0.375 | 0.273 | 0.174 | 0.000 | 0.125 | 0.000 | 0.050 | 0.238 | 0.042 | 0.000 | 0.000 | 0.333 | 0.000 | 0.435 |
| 31-253 | 0.000 | 0.059 | 0.000 | 0.000 | 0.000 | 0.000 | 0.000 | 0.000 | 0.000 | 0.000 | 0.130 | 0.000 | 0.042 | 0.000 | 0.000 | 0.048 | 0.000 | 0.000 | 0.000 | 0.000 | 0.000 | 0.000 |
| 31-268 | 0.000 | 0.059 | 0.000 | 0.000 | 0.000 | 0.000 | 0.000 | 0.000 | 0.000 | 0.000 | 0.000 | 0.000 | 0.000 | 0.000 | 0.000 | 0.000 | 0.000 | 0.000 | 0.000 | 0.000 | 0.000 | 0.043 |
| 31-274 | 0.000 | 0.059 | 0.087 | 0.000 | 0.000 | 0.000 | 0.000 | 0.000 | 0.000 | 0.000 | 0.217 | 0.000 | 0.042 | 0.000 | 0.050 | 0.000 | 0.000 | 0.000 | 0.000 | 0.000 | 0.000 | 0.043 |
| 31-281 | 0.000 | 0.000 | 0.000 | 0.000 | 0.000 | 0.000 | 0.000 | 0.000 | 0.000 | 0.000 | 0.000 | 0.000 | 0.042 | 0.000 | 0.000 | 0.000 | 0.000 | 0.000 | 0.000 | 0.000 | 0.000 | 0.000 |
| 31-291 | 0.000 | 0.000 | 0.130 | 0.000 | 0.000 | 0.000 | 0.000 | 0.000 | 0.000 | 0.045 | 0.043 | 0.000 | 0.000 | 0.000 | 0.000 | 0.000 | 0.000 | 0.000 | 0.000 | 0.000 | 0.000 | 0.000 |
| 31-300 | 0.136 | 0.118 | 0.087 | 0.000 | 0.000 | 0.000 | 0.087 | 0.000 | 0.000 | 0.000 | 0.130 | 0.125 | 0.000 | 0.167 | 0.100 | 0.381 | 0.125 | 0.125 | 0.083 | 0.167 | 0.000 | 0.391 |
| 31-309 | 0.000 | 0.059 | 0.043 | 0.000 | 0.000 | 0.000 | 0.000 | 0.000 | 0.792 | 0.909 | 0.043 | 0.000 | 0.000 | 0.000 | 0.000 | 0.000 | 0.000 | 0.083 | 0.000 | 0.000 | 0.273 | 0.000 |
| 31-313 | 0.000 | 0.059 | 0.087 | 0.043 | 0.000 | 0.000 | 0.000 | 0.000 | 0.792 | 0.909 | 0.304 | 0.042 | 0.083 | 0.000 | 0.000 | 0.095 | 0.000 | 0.167 | 0.000 | 0.125 | 0.273 | 0.130 |
| 31-317 | 0.000 | 0.118 | 0.000 | 0.000 | 0.000 | 0.000 | 0.043 | 0.000 | 0.333 | 0.455 | 0.043 | 0.042 | 0.250 | 0.000 | 0.000 | 0.048 | 0.000 | 0.208 | 0.000 | 0.167 | 0.364 | 0.261 |
| 31-321 | 0.045 | 0.059 | 0.000 | 0.000 | 0.000 | 0.000 | 0.000 | 0.000 | 0.250 | 0.409 | 0.000 | 0.000 | 0.000 | 0.000 | 0.000 | 0.000 | 0.000 | 0.000 | 0.000 | 0.000 | 0.000 | 0.087 |
| 31-333 | 0.000 | 0.000 | 0.043 | 0.000 | 0.000 | 0.043 | 0.000 | 0.000 | 0.000 | 0.045 | 0.043 | 0.000 | 0.042 | 0.000 | 0.050 | 0.000 | 0.000 | 0.042 | 0.000 | 0.000 | 0.045 | 0.043 |
| 31-342 | 0.000 | 0.059 | 0.000 | 0.000 | 0.000 | 0.000 | 0.000 | 0.000 | 0.042 | 0.045 | 0.000 | 0.000 | 0.042 | 0.000 | 0.000 | 0.000 | 0.000 | 0.000 | 0.000 | 0.000 | 0.000 | 0.000 |
| 31-351 | 0.000 | 0.000 | 0.043 | 0.000 | 0.000 | 0.000 | 0.000 | 0.000 | 0.208 | 0.364 | 0.087 | 0.000 | 0.042 | 0.042 | 0.000 | 0.000 | 0.000 | 0.000 | 0.042 | 0.000 | 0.000 | 0.043 |
| 31-358 | 0.000 | 0.000 | 0.000 | 0.000 | 0.000 | 0.000 | 0.000 | 0.000 | 0.000 | 0.000 | 0.000 | 0.042 | 0.042 | 0.000 | 0.000 | 0.000 | 0.000 | 0.000 | 0.000 | 0.042 | 0.000 | 0.000 |
| 31-365 | 0.000 | 0.000 | 0.000 | 0.000 | 0.000 | 0.000 | 0.000 | 0.000 | 0.000 | 0.000 | 0.043 | 0.000 | 0.167 | 0.000 | 0.000 | 0.000 | 0.000 | 0.000 | 0.000 | 0.000 | 0.000 | 0.000 |
| 31-374 | 0.000 | 0.000 | 0.000 | 0.000 | 0.000 | 0.000 | 0.000 | 0.000 | 0.000 | 0.000 | 0.000 | 0.042 | 0.000 | 0.000 | 0.000 | 0.000 | 0.000 | 0.042 | 0.000 | 0.000 | 0.000 | 0.000 |
| 31-378 | 0.000 | 0.000 | 0.000 | 0.000 | 0.000 | 0.000 | 0.000 | 0.000 | 0.000 | 0.000 | 0.000 | 0.042 | 0.208 | 0.000 | 0.050 | 0.095 | 0.000 | 0.083 | 0.000 | 0.000 | 0.000 | 0.304 |
| 31-382 | 0.000 | 0.000 | 0.000 | 0.043 | 0.000 | 0.000 | 0.000 | 0.000 | 0.083 | 0.364 | 0.043 | 0.000 | 0.625 | 0.000 | 0.000 | 0.190 | 0.000 | 0.417 | 0.042 | 0.333 | 0.864 | 0.609 |
| 31-390 | 0.636 | 0.235 | 0.652 | 0.478 | 0.667 | 0.130 | 0.565 | 0.667 | 0.042 | 0.000 | 0.087 | 0.167 | 0.750 | 0.375 | 0.550 | 0.619 | 0.792 | 0.708 | 0.292 | 0.375 | 0.909 | 0.609 |
| 31-395 | 0.000 | 0.000 | 0.000 | 0.000 | 0.000 | 0.000 | 0.000 | 0.000 | 0.000 | 0.045 | 0.043 | 0.000 | 0.208 | 0.000 | 0.000 | 0.000 | 0.000 | 0.000 | 0.000 | 0.000 | 0.000 | 0.000 |
| 31-403 | 0.000 | 0.000 | 0.000 | 0.000 | 0.000 | 0.000 | 0.000 | 0.000 | 0.042 | 0.000 | 0.043 | 0.000 | 0.000 | 0.000 | 0.000 | 0.000 | 0.000 | 0.000 | 0.000 | 0.000 | 0.000 | 0.043 |
| 31-408 | 0.136 | 0.059 | 0.913 | 0.130 | 0.222 | 0.000 | 0.043 | 0.048 | 0.292 | 0.000 | 0.783 | 0.167 | 0.292 | 0.083 | 0.100 | 0.143 | 0.292 | 0.583 | 0.125 | 0.042 | 0.591 | 0.435 |
| 31-412 | 0.000 | 0.000 | 0.957 | 0.130 | 0.278 | 0.000 | 0.087 | 0.048 | 0.083 | 0.000 | 0.826 | 0.125 | 0.292 | 0.042 | 0.100 | 0.095 | 0.250 | 0.500 | 0.083 | 0.042 | 0.455 | 0.348 |
| 31-416 | 0.136 | 0.000 | 0.000 | 0.261 | 0.167 | 0.783 | 0.000 | 0.571 | 0.083 | 0.045 | 0.391 | 0.125 | 0.042 | 0.125 | 0.150 | 0.286 | 0.333 | 0.333 | 0.667 | 0.000 | 0.364 | 0.217 |
| 31-424 | 0.091 | 0.118 | 0.783 | 0.174 | 0.278 | 0.043 | 0.217 | 0.238 | 0.250 | 0.045 | 0.870 | 0.000 | 0.125 | 0.042 | 0.000 | 0.524 | 0.292 | 0.458 | 0.167 | 0.042 | 0.591 | 0.391 |
| 31-428 | 0.000 | 0.000 | 0.043 | 0.000 | 0.000 | 0.000 | 0.000 | 0.000 | 0.000 | 0.000 | 0.000 | 0.000 | 0.000 | 0.000 | 0.000 | 0.000 | 0.000 | 0.000 | 0.000 | 0.000 | 0.000 | 0.043 |
| 31-435 | 0.000 | 0.118 | 0.217 | 0.000 | 0.111 | 0.000 | 0.087 | 0.095 | 0.000 | 0.091 | 0.826 | 0.000 | 0.083 | 0.042 | 0.000 | 0.095 | 0.083 | 0.250 | 0.042 | 0.042 | 0.045 | 0.304 |
| 31-444 | 0.000 | 0.000 | 0.000 | 0.000 | 0.000 | 0.000 | 0.000 | 0.000 | 0.167 | 0.045 | 0.000 | 0.000 | 0.000 | 0.000 | 0.000 | 0.000 | 0.000 | 0.125 | 0.000 | 0.000 | 0.045 | 0.043 |
| 31-450 | 0.500 | 0.235 | 0.957 | 0.478 | 0.500 | 0.391 | 0.609 | 0.571 | 0.875 | 0.909 | 0.826 | 0.500 | 0.250 | 0.208 | 0.600 | 0.619 | 0.667 | 0.708 | 0.375 | 0.125 | 0.682 | 0.348 |
| 31-456 | 0.000 | 0.000 | 0.043 | 0.000 | 0.000 | 0.000 | 0.000 | 0.000 | 0.000 | 0.000 | 0.043 | 0.000 | 0.000 | 0.000 | 0.050 | 0.000 | 0.000 | 0.000 | 0.000 | 0.000 | 0.000 | 0.043 |
| 31-461 | 0.000 | 0.000 | 0.000 | 0.000 | 0.000 | 0.000 | 0.000 | 0.000 | 0.042 | 0.000 | 0.348 | 0.000 | 0.000 | 0.000 | 0.000 | 0.000 | 0.000 | 0.000 | 0.000 | 0.000 | 0.000 | 0.043 |
| 31-472 | 0.000 | 0.000 | 0.174 | 0.000 | 0.000 | 0.000 | 0.000 | 0.000 | 0.000 | 0.045 | 0.391 | 0.000 | 0.000 | 0.000 | 0.000 | 0.095 | 0.000 | 0.042 | 0.042 | 0.000 | 0.273 | 0.174 |
| 31-483 | 0.000 | 0.059 | 0.000 | 0.000 | 0.000 | 0.000 | 0.000 | 0.000 | 0.000 | 0.000 | 0.043 | 0.000 | 0.000 | 0.000 | 0.000 | 0.000 | 0.000 | 0.000 | 0.000 | 0.000 | 0.000 | 0.000 |
| 31-487 | 0.000 | 0.059 | 0.000 | 0.000 | 0.000 | 0.000 | 0.043 | 0.000 | 0.125 | 0.000 | 0.000 | 0.000 | 0.000 | 0.000 | 0.000 | 0.000 | 0.000 | 0.000 | 0.000 | 0.000 | 0.000 | 0.000 |
| 31-495 | 0.000 | 0.000 | 0.000 | 0.000 | 0.000 | 0.000 | 0.000 | 0.000 | 0.417 | 0.000 | 0.043 | 0.000 | 0.083 | 0.000 | 0.000 | 0.000 | 0.000 | 0.042 | 0.000 | 0.000 | 0.091 | 0.000 |
| 31-501 | 0.000 | 0.000 | 0.000 | 0.000 | 0.000 | 0.000 | 0.000 | 0.000 | 0.250 | 0.000 | 0.000 | 0.042 | 0.000 | 0.000 | 0.000 | 0.000 | 0.000 | 0.000 | 0.000 | 0.000 | 0.091 | 0.000 |
| 31-510 | 0.000 | 0.000 | 0.000 | 0.000 | 0.000 | 0.000 | 0.000 | 0.000 | 0.000 | 0.000 | 0.000 | 0.000 | 0.042 | 0.000 | 0.000 | 0.048 | 0.000 | 0.000 | 0.000 | 0.000 | 0.000 | 0.000 |
| 31-527 | 0.045 | 0.000 | 0.000 | 0.000 | 0.111 | 0.000 | 0.000 | 0.000 | 0.208 | 0.000 | 0.000 | 0.125 | 0.000 | 0.042 | 0.000 | 0.000 | 0.000 | 0.125 | 0.042 | 0.042 | 0.318 | 0.000 |
| 31-536 | 0.000 | 0.000 | 0.043 | 0.000 | 0.000 | 0.000 | 0.000 | 0.000 | 0.000 | 0.000 | 0.000 | 0.000 | 0.000 | 0.000 | 0.000 | 0.000 | 0.000 | 0.000 | 0.000 | 0.000 | 0.000 | 0.000 |
| 31-540 | 0.864 | 0.765 | 0.043 | 0.565 | 0.611 | 0.391 | 0.957 | 0.429 | 0.167 | 0.000 | 0.304 | 1.000 | 1.000 | 0.917 | 0.400 | 0.762 | 0.917 | 0.917 | 1.000 | 0.917 | 0.864 | 0.957 |
| 31-548 | 0.000 | 0.000 | 0.000 | 0.000 | 0.000 | 0.000 | 0.000 | 0.000 | 0.000 | 0.000 | 0.087 | 0.000 | 0.042 | 0.000 | 0.000 | 0.000 | 0.000 | 0.000 | 0.000 | 0.000 | 0.091 | 0.043 |
| 31-552 | 0.000 | 0.000 | 0.000 | 0.000 | 0.000 | 0.000 | 0.000 | 0.000 | 0.042 | 0.000 | 0.000 | 0.000 | 0.083 | 0.000 | 0.000 | 0.000 | 0.000 | 0.000 | 0.000 | 0.000 | 0.000 | 0.000 |
| 31-558 | 0.000 | 0.000 | 0.000 | 0.000 | 0.000 | 0.000 | 0.000 | 0.000 | 0.000 | 0.045 | 0.043 | 0.000 | 0.000 | 0.042 | 0.000 | 0.000 | 0.000 | 0.000 | 0.000 | 0.042 | 0.000 | 0.043 |
| 31-562 | 0.000 | 0.000 | 0.000 | 0.000 | 0.000 | 0.000 | 0.000 | 0.000 | 0.042 | 0.136 | 0.087 | 0.000 | 0.000 | 0.000 | 0.000 | 0.000 | 0.000 | 0.000 | 0.000 | 0.000 | 0.000 | 0.000 |
| 31-568 | 0.000 | 0.000 | 0.000 | 0.000 | 0.000 | 0.000 | 0.000 | 0.000 | 0.000 | 0.000 | 0.000 | 0.000 | 0.042 | 0.000 | 0.000 | 0.000 | 0.000 | 0.000 | 0.000 | 0.000 | 0.000 | 0.000 |
| 31-578 | 0.000 | 0.000 | 0.000 | 0.000 | 0.000 | 0.000 | 0.000 | 0.000 | 0.000 | 0.000 | 0.043 | 0.000 | 0.042 | 0.000 | 0.000 | 0.000 | 0.000 | 0.000 | 0.000 | 0.000 | 0.000 | 0.000 |
| 31-586 | 0.000 | 0.000 | 0.000 | 0.000 | 0.000 | 0.000 | 0.000 | 0.000 | 0.000 | 0.000 | 0.000 | 0.000 | 0.000 | 0.000 | 0.000 | 0.000 | 0.000 | 0.083 | 0.042 | 0.000 | 0.091 | 0.000 |
| 31-598 | 0.000 | 0.118 | 0.000 | 0.000 | 0.000 | 0.000 | 0.000 | 0.000 | 0.000 | 0.000 | 0.000 | 0.000 | 0.042 | 0.000 | 0.000 | 0.048 | 0.000 | 0.125 | 0.000 | 0.125 | 0.045 | 0.087 |
| 31-603 | 0.000 | 0.000 | 0.000 | 0.000 | 0.000 | 0.000 | 0.000 | 0.000 | 0.000 | 0.000 | 0.043 | 0.000 | 0.042 | 0.000 | 0.000 | 0.000 | 0.000 | 0.000 | 0.000 | 0.125 | 0.045 | 0.174 |
| 31-610 | 0.000 | 0.000 | 0.000 | 0.000 | 0.000 | 0.000 | 0.000 | 0.000 | 0.000 | 0.000 | 0.000 | 0.000 | 0.000 | 0.000 | 0.000 | 0.000 | 0.000 | 0.042 | 0.000 | 0.000 | 0.136 | 0.217 |
| 31-615 | 0.000 | 0.000 | 0.000 | 0.000 | 0.000 | 0.000 | 0.087 | 0.000 | 0.000 | 0.000 | 0.000 | 0.000 | 0.000 | 0.000 | 0.000 | 0.048 | 0.000 | 0.000 | 0.000 | 0.042 | 0.045 | 0.217 |
| 31-619 | 0.000 | 0.000 | 0.000 | 0.000 | 0.000 | 0.000 | 0.000 | 0.000 | 0.000 | 0.000 | 0.000 | 0.000 | 0.000 | 0.000 | 0.000 | 0.000 | 0.000 | 0.042 | 0.000 | 0.125 | 0.000 | 0.000 |
| 31-623 | 0.045 | 0.059 | 0.826 | 0.043 | 0.222 | 0.087 | 0.304 | 0.333 | 0.833 | 0.955 | 0.652 | 0.125 | 0.167 | 0.042 | 0.250 | 0.333 | 0.125 | 0.500 | 0.208 | 0.125 | 0.682 | 0.435 |
| 31-631 | 0.000 | 0.000 | 0.000 | 0.000 | 0.000 | 0.000 | 0.000 | 0.000 | 0.000 | 0.045 | 0.000 | 0.000 | 0.000 | 0.000 | 0.000 | 0.000 | 0.000 | 0.042 | 0.000 | 0.000 | 0.000 | 0.043 |
| 31-637 | 0.000 | 0.059 | 0.000 | 0.000 | 0.000 | 0.000 | 0.000 | 0.000 | 0.000 | 0.045 | 0.000 | 0.000 | 0.000 | 0.000 | 0.000 | 0.000 | 0.000 | 0.000 | 0.000 | 0.000 | 0.000 | 0.000 |
| 31-646 | 0.000 | 0.000 | 0.174 | 0.000 | 0.000 | 0.000 | 0.000 | 0.000 | 0.000 | 0.045 | 0.000 | 0.000 | 0.000 | 0.000 | 0.000 | 0.000 | 0.000 | 0.000 | 0.000 | 0.000 | 0.000 | 0.043 |
| 31-651 | 0.000 | 0.000 | 0.130 | 0.000 | 0.000 | 0.000 | 0.000 | 0.000 | 0.000 | 0.000 | 0.000 | 0.000 | 0.042 | 0.000 | 0.000 | 0.000 | 0.000 | 0.000 | 0.000 | 0.000 | 0.000 | 0.000 |
| 31-664 | 0.000 | 0.000 | 0.000 | 0.000 | 0.000 | 0.000 | 0.000 | 0.000 | 0.000 | 0.000 | 0.043 | 0.000 | 0.000 | 0.000 | 0.000 | 0.000 | 0.000 | 0.000 | 0.000 | 0.000 | 0.000 | 0.000 |
| 31-669 | 0.000 | 0.000 | 0.000 | 0.000 | 0.000 | 0.000 | 0.000 | 0.000 | 0.000 | 0.000 | 0.043 | 0.000 | 0.000 | 0.000 | 0.000 | 0.000 | 0.000 | 0.000 | 0.000 | 0.000 | 0.000 | 0.000 |
| 31-673 | 0.000 | 0.000 | 0.000 | 0.000 | 0.000 | 0.000 | 0.000 | 0.000 | 0.042 | 0.045 | 0.043 | 0.000 | 0.000 | 0.000 | 0.000 | 0.000 | 0.000 | 0.000 | 0.000 | 0.000 | 0.000 | 0.000 |
| 31-693 | 0.000 | 0.000 | 0.000 | 0.000 | 0.000 | 0.000 | 0.000 | 0.000 | 0.083 | 0.091 | 0.000 | 0.000 | 0.000 | 0.000 | 0.000 | 0.000 | 0.000 | 0.000 | 0.000 | 0.000 | 0.000 | 0.000 |
| 31-709 | 0.000 | 0.000 | 0.000 | 0.000 | 0.000 | 0.000 | 0.000 | 0.000 | 0.042 | 0.000 | 0.000 | 0.000 | 0.000 | 0.000 | 0.000 | 0.000 | 0.000 | 0.000 | 0.000 | 0.000 | 0.000 | 0.000 |
| 31-729 | 0.000 | 0.000 | 0.000 | 0.000 | 0.000 | 0.000 | 0.000 | 0.000 | 0.000 | 0.000 | 0.087 | 0.000 | 0.000 | 0.000 | 0.000 | 0.000 | 0.000 | 0.000 | 0.000 | 0.000 | 0.000 | 0.000 |
| 31-741 | 0.000 | 0.000 | 0.043 | 0.000 | 0.000 | 0.000 | 0.000 | 0.000 | 0.000 | 0.000 | 0.000 | 0.000 | 0.000 | 0.000 | 0.000 | 0.000 | 0.000 | 0.000 | 0.000 | 0.000 | 0.045 | 0.000 |
| 31-759 | 0.000 | 0.000 | 0.087 | 0.000 | 0.000 | 0.000 | 0.000 | 0.000 | 0.125 | 0.182 | 0.304 | 0.000 | 0.000 | 0.000 | 0.000 | 0.000 | 0.000 | 0.000 | 0.000 | 0.000 | 0.000 | 0.000 |
| 31-779 | 0.000 | 0.000 | 0.043 | 0.000 | 0.000 | 0.000 | 0.000 | 0.000 | 0.000 | 0.000 | 0.000 | 0.000 | 0.000 | 0.000 | 0.000 | 0.000 | 0.000 | 0.000 | 0.000 | 0.000 | 0.000 | 0.043 |
| 31-791 | 0.000 | 0.000 | 0.000 | 0.000 | 0.000 | 0.000 | 0.000 | 0.000 | 0.000 | 0.000 | 0.000 | 0.000 | 0.000 | 0.000 | 0.000 | 0.048 | 0.000 | 0.042 | 0.000 | 0.042 | 0.000 | 0.043 |
| 31-816 | 0.000 | 0.000 | 0.000 | 0.000 | 0.000 | 0.000 | 0.000 | 0.000 | 0.042 | 0.000 | 0.043 | 0.000 | 0.000 | 0.000 | 0.000 | 0.000 | 0.000 | 0.000 | 0.000 | 0.000 | 0.000 | 0.000 |
| 31-832 | 0.000 | 0.000 | 0.000 | 0.000 | 0.000 | 0.000 | 0.000 | 0.000 | 0.000 | 0.000 | 0.000 | 0.000 | 0.000 | 0.000 | 0.000 | 0.000 | 0.000 | 0.042 | 0.000 | 0.000 | 0.000 | 0.000 |
| 31-943 | 0.000 | 0.000 | 0.043 | 0.000 | 0.000 | 0.000 | 0.000 | 0.000 | 0.000 | 0.000 | 0.000 | 0.000 | 0.000 | 0.000 | 0.000 | 0.000 | 0.000 | 0.000 | 0.000 | 0.000 | 0.045 | 0.000 |
| 31-951 | 0.000 | 0.000 | 0.000 | 0.000 | 0.000 | 0.000 | 0.000 | 0.000 | 0.000 | 0.000 | 0.000 | 0.000 | 0.000 | 0.000 | 0.000 | 0.000 | 0.000 | 0.000 | 0.000 | 0.000 | 0.091 | 0.000 |
| 31-1053 | 0.000 | 0.000 | 0.000 | 0.000 | 0.000 | 0.000 | 0.000 | 0.000 | 0.042 | 0.000 | 0.000 | 0.000 | 0.000 | 0.000 | 0.000 | 0.000 | 0.000 | 0.000 | 0.000 | 0.000 | 0.000 | 0.000 |
| 31-1063 | 0.000 | 0.000 | 0.043 | 0.000 | 0.000 | 0.000 | 0.000 | 0.000 | 0.125 | 0.000 | 0.000 | 0.000 | 0.000 | 0.000 | 0.000 | 0.000 | 0.000 | 0.000 | 0.000 | 0.000 | 0.000 | 0.000 |
| 31-1073 | 0.000 | 0.000 | 0.000 | 0.000 | 0.000 | 0.000 | 0.000 | 0.000 | 0.000 | 0.091 | 0.000 | 0.000 | 0.000 | 0.000 | 0.000 | 0.000 | 0.000 | 0.000 | 0.000 | 0.000 | 0.000 | 0.000 |
| 31-1081 | 0.000 | 0.000 | 0.000 | 0.000 | 0.000 | 0.000 | 0.000 | 0.000 | 0.167 | 0.045 | 0.000 | 0.000 | 0.000 | 0.000 | 0.000 | 0.000 | 0.000 | 0.000 | 0.000 | 0.000 | 0.000 | 0.000 |
| 35-104 | 0.000 | 0.000 | 0.000 | 0.000 | 0.143 | 0.000 | 0.000 | 0.000 | 0.000 | 0.000 | 0.000 | 0.000 | 0.000 | 0.000 | 0.000 | 0.000 | 0.000 | 0.000 | 0.000 | 0.000 | 0.111 | 0.000 |
| 35-149 | 0.000 | 0.000 | 0.000 | 0.000 | 0.000 | 0.000 | 0.000 | 0.053 | 0.000 | 0.000 | 0.000 | 0.000 | 0.000 | 0.000 | 0.000 | 0.000 | 0.000 | 0.000 | 0.000 | 0.000 | 0.000 | 0.000 |
| 35-180 | 0.000 | 0.045 | 0.000 | 0.087 | 0.000 | 0.000 | 0.048 | 0.000 | 0.000 | 0.000 | 0.000 | 0.000 | 0.000 | 0.000 | 0.000 | 0.000 | 0.000 | 0.000 | 0.000 | 0.000 | 0.000 | 0.000 |
| 35-193 | 0.000 | 0.000 | 0.043 | 0.000 | 0.000 | 0.095 | 0.000 | 0.000 | 0.000 | 0.000 | 0.000 | 0.000 | 0.000 | 0.000 | 0.000 | 0.100 | 0.000 | 0.000 | 0.000 | 0.083 | 0.000 | 0.043 |
| 35-206 | 0.000 | 0.000 | 0.000 | 0.000 | 0.000 | 0.000 | 0.048 | 0.000 | 0.000 | 0.000 | 0.000 | 0.000 | 0.000 | 0.000 | 0.000 | 0.000 | 0.000 | 0.000 | 0.000 | 0.000 | 0.000 | 0.000 |
| 35-228 | 0.000 | 0.000 | 0.000 | 0.000 | 0.000 | 0.048 | 0.000 | 0.000 | 0.000 | 0.000 | 0.000 | 0.050 | 0.000 | 0.000 | 0.000 | 0.000 | 0.000 | 0.000 | 0.000 | 0.000 | 0.000 | 0.000 |
| 35-238 | 0.000 | 0.000 | 0.000 | 0.000 | 0.000 | 0.048 | 0.000 | 0.053 | 0.000 | 0.000 | 0.000 | 0.000 | 0.000 | 0.000 | 0.000 | 0.000 | 0.000 | 0.000 | 0.000 | 0.000 | 0.000 | 0.217 |
| 35-268 | 0.727 | 0.000 | 0.000 | 0.130 | 0.286 | 0.381 | 0.095 | 0.842 | 0.591 | 0.000 | 0.000 | 1.000 | 0.176 | 0.615 | 0.688 | 0.000 | 0.417 | 0.696 | 1.000 | 0.000 | 0.056 | 0.000 |
| 35-285 | 0.000 | 0.045 | 0.000 | 0.000 | 0.143 | 0.000 | 0.000 | 0.053 | 0.000 | 0.000 | 0.000 | 0.000 | 0.000 | 0.000 | 0.000 | 0.000 | 0.000 | 0.000 | 0.000 | 0.000 | 0.056 | 0.000 |
| 35-306 | 0.364 | 0.545 | 0.043 | 0.478 | 0.143 | 0.095 | 0.476 | 0.316 | 0.045 | 0.000 | 0.043 | 0.000 | 0.000 | 0.538 | 0.438 | 0.800 | 0.125 | 0.043 | 0.071 | 0.792 | 0.111 | 0.522 |
| 35-311 | 0.045 | 0.227 | 0.522 | 0.130 | 0.000 | 0.476 | 0.048 | 0.579 | 0.682 | 0.364 | 0.391 | 0.000 | 0.294 | 0.000 | 0.000 | 0.200 | 0.000 | 0.000 | 0.000 | 0.500 | 0.000 | 0.609 |
| 35-318 | 0.000 | 0.000 | 0.000 | 0.000 | 0.000 | 0.000 | 0.000 | 0.000 | 0.045 | 0.000 | 0.000 | 0.000 | 0.000 | 0.000 | 0.000 | 0.000 | 0.000 | 0.000 | 0.000 | 0.000 | 0.000 | 0.000 |
| 35-328 | 0.864 | 0.000 | 0.043 | 0.000 | 0.000 | 0.286 | 0.000 | 0.316 | 0.000 | 0.000 | 0.000 | 0.200 | 0.706 | 0.154 | 0.250 | 0.250 | 0.917 | 0.696 | 0.071 | 0.292 | 0.722 | 0.130 |
| 35-333 | 0.909 | 0.000 | 0.043 | 0.000 | 0.000 | 0.286 | 0.000 | 0.316 | 0.000 | 0.000 | 0.000 | 0.100 | 0.765 | 0.077 | 0.125 | 0.200 | 0.833 | 0.826 | 0.143 | 0.375 | 0.722 | 0.130 |
| 35-337 | 0.000 | 0.045 | 0.696 | 0.000 | 0.000 | 0.190 | 0.000 | 0.000 | 0.136 | 0.864 | 0.826 | 0.000 | 0.000 | 0.000 | 0.000 | 0.150 | 0.000 | 0.000 | 0.000 | 0.167 | 0.000 | 0.087 |
| 35-340 | 0.000 | 0.000 | 0.435 | 0.000 | 0.000 | 0.143 | 0.000 | 0.000 | 0.136 | 0.773 | 0.652 | 0.000 | 0.000 | 0.000 | 0.000 | 0.000 | 0.000 | 0.000 | 0.000 | 0.042 | 0.000 | 0.000 |
| 35-354 | 0.227 | 0.136 | 0.000 | 0.043 | 0.000 | 0.095 | 0.143 | 0.158 | 0.045 | 0.091 | 0.000 | 0.150 | 0.000 | 0.077 | 0.125 | 0.150 | 0.083 | 0.174 | 0.000 | 0.125 | 0.167 | 0.261 |
| 35-358 | 0.091 | 0.136 | 0.000 | 0.043 | 0.000 | 0.000 | 0.048 | 0.105 | 0.000 | 0.091 | 0.000 | 0.150 | 0.000 | 0.000 | 0.000 | 0.200 | 0.042 | 0.174 | 0.000 | 0.125 | 0.000 | 0.130 |
| 35-364 | 0.000 | 0.045 | 0.043 | 0.000 | 0.000 | 0.000 | 0.048 | 0.000 | 0.045 | 0.091 | 0.087 | 0.000 | 0.000 | 0.000 | 0.000 | 0.000 | 0.000 | 0.000 | 0.000 | 0.042 | 0.000 | 0.043 |
| 35-379 | 0.000 | 0.000 | 0.000 | 0.000 | 0.000 | 0.000 | 0.048 | 0.000 | 0.000 | 0.000 | 0.043 | 0.000 | 0.000 | 0.000 | 0.000 | 0.050 | 0.000 | 0.000 | 0.000 | 0.000 | 0.000 | 0.000 |
| 35-389 | 0.000 | 0.000 | 0.000 | 0.043 | 0.000 | 0.000 | 0.000 | 0.000 | 0.000 | 0.000 | 0.000 | 0.000 | 0.000 | 0.000 | 0.000 | 0.000 | 0.000 | 0.000 | 0.000 | 0.000 | 0.000 | 0.000 |
| 35-400 | 0.000 | 0.000 | 0.043 | 0.000 | 0.000 | 0.000 | 0.000 | 0.000 | 0.000 | 0.000 | 0.000 | 0.000 | 0.000 | 0.000 | 0.000 | 0.000 | 0.042 | 0.000 | 0.000 | 0.000 | 0.000 | 0.000 |
| 35-411 | 0.000 | 0.000 | 0.000 | 0.000 | 0.000 | 0.000 | 0.000 | 0.000 | 0.000 | 0.000 | 0.000 | 0.000 | 0.000 | 0.000 | 0.000 | 0.000 | 0.000 | 0.000 | 0.000 | 0.083 | 0.000 | 0.000 |
| 35-417 | 0.000 | 0.045 | 0.000 | 0.000 | 0.000 | 0.000 | 0.000 | 0.000 | 0.000 | 0.000 | 0.000 | 0.000 | 0.000 | 0.000 | 0.000 | 0.000 | 0.000 | 0.000 | 0.000 | 0.000 | 0.000 | 0.000 |
| 35-445 | 0.000 | 0.045 | 0.000 | 0.000 | 0.000 | 0.048 | 0.000 | 0.105 | 0.045 | 0.000 | 0.000 | 0.000 | 0.059 | 0.077 | 0.000 | 0.000 | 0.000 | 0.000 | 0.000 | 0.000 | 0.000 | 0.000 |
| 35-455 | 0.045 | 0.000 | 0.043 | 0.000 | 0.000 | 0.000 | 0.048 | 0.000 | 0.000 | 0.000 | 0.000 | 0.000 | 0.000 | 0.000 | 0.000 | 0.000 | 0.083 | 0.348 | 0.071 | 0.000 | 0.000 | 0.000 |
| 35-465 | 0.000 | 0.000 | 0.000 | 0.000 | 0.000 | 0.000 | 0.000 | 0.000 | 0.000 | 0.000 | 0.000 | 0.000 | 0.000 | 0.077 | 0.063 | 0.000 | 0.000 | 0.217 | 0.000 | 0.000 | 0.000 | 0.000 |
| 35-475 | 0.000 | 0.045 | 0.000 | 0.043 | 0.000 | 0.000 | 0.048 | 0.000 | 0.000 | 0.000 | 0.000 | 0.000 | 0.000 | 0.000 | 0.000 | 0.000 | 0.000 | 0.000 | 0.000 | 0.000 | 0.000 | 0.000 |
| 35-480 | 0.318 | 0.636 | 0.087 | 0.739 | 0.000 | 0.000 | 0.667 | 0.105 | 0.182 | 0.091 | 0.000 | 0.100 | 0.000 | 0.231 | 0.188 | 0.500 | 0.250 | 0.217 | 0.071 | 0.583 | 0.056 | 0.478 |
| 35-483 | 0.318 | 0.636 | 0.000 | 0.826 | 0.000 | 0.000 | 0.667 | 0.105 | 0.136 | 0.000 | 0.000 | 0.200 | 0.000 | 0.231 | 0.125 | 0.500 | 0.250 | 0.261 | 0.000 | 0.542 | 0.056 | 0.522 |
| 35-490 | 0.000 | 0.364 | 0.000 | 0.217 | 0.000 | 0.000 | 0.381 | 0.000 | 0.000 | 0.000 | 0.000 | 0.000 | 0.000 | 0.000 | 0.000 | 0.100 | 0.000 | 0.000 | 0.071 | 0.083 | 0.000 | 0.217 |
| 35-497 | 0.000 | 0.000 | 0.348 | 0.000 | 0.000 | 0.048 | 0.000 | 0.053 | 0.045 | 0.182 | 0.174 | 0.000 | 0.000 | 0.000 | 0.000 | 0.050 | 0.000 | 0.000 | 0.000 | 0.000 | 0.000 | 0.000 |
| 35-500 | 0.182 | 0.000 | 0.000 | 0.000 | 0.000 | 0.048 | 0.000 | 0.158 | 0.000 | 0.045 | 0.000 | 0.000 | 0.059 | 0.000 | 0.000 | 0.050 | 0.167 | 0.174 | 0.000 | 0.042 | 0.000 | 0.261 |
| 35-508 | 0.136 | 0.000 | 0.000 | 0.043 | 0.000 | 0.095 | 0.000 | 0.158 | 0.000 | 0.000 | 0.000 | 0.000 | 0.059 | 0.000 | 0.000 | 0.000 | 0.208 | 0.130 | 0.000 | 0.000 | 0.056 | 0.261 |
| 35-511 | 0.000 | 0.045 | 0.000 | 0.000 | 0.000 | 0.000 | 0.000 | 0.000 | 0.000 | 0.000 | 0.087 | 0.000 | 0.000 | 0.000 | 0.000 | 0.050 | 0.000 | 0.000 | 0.000 | 0.000 | 0.000 | 0.043 |
| 35-517 | 0.000 | 0.000 | 0.000 | 0.000 | 0.000 | 0.000 | 0.000 | 0.000 | 0.000 | 0.000 | 0.043 | 0.000 | 0.000 | 0.000 | 0.000 | 0.000 | 0.000 | 0.000 | 0.000 | 0.000 | 0.000 | 0.000 |
| 35-534 | 0.000 | 0.000 | 0.000 | 0.000 | 0.000 | 0.000 | 0.000 | 0.000 | 0.000 | 0.000 | 0.000 | 0.000 | 0.000 | 0.000 | 0.000 | 0.150 | 0.000 | 0.043 | 0.000 | 0.000 | 0.000 | 0.000 |
| 35-538 | 0.091 | 0.000 | 0.000 | 0.000 | 0.000 | 0.000 | 0.000 | 0.000 | 0.000 | 0.000 | 0.000 | 0.000 | 0.000 | 0.000 | 0.000 | 0.000 | 0.042 | 0.087 | 0.000 | 0.000 | 0.056 | 0.000 |
| 35-547 | 0.000 | 0.000 | 0.043 | 0.000 | 0.000 | 0.000 | 0.000 | 0.000 | 0.000 | 0.000 | 0.000 | 0.000 | 0.059 | 0.000 | 0.000 | 0.000 | 0.000 | 0.000 | 0.000 | 0.000 | 0.000 | 0.000 |
| 35-559 | 0.000 | 0.000 | 0.391 | 0.000 | 0.000 | 0.238 | 0.000 | 0.105 | 0.273 | 0.773 | 0.609 | 0.000 | 0.000 | 0.000 | 0.000 | 0.000 | 0.000 | 0.000 | 0.000 | 0.000 | 0.000 | 0.043 |
| 35-587 | 0.000 | 0.045 | 0.000 | 0.000 | 0.000 | 0.143 | 0.000 | 0.158 | 0.273 | 0.045 | 0.000 | 0.000 | 0.000 | 0.000 | 0.000 | 0.000 | 0.042 | 0.000 | 0.000 | 0.000 | 0.000 | 0.000 |
| 35-596 | 0.000 | 0.000 | 0.087 | 0.000 | 0.000 | 0.000 | 0.000 | 0.053 | 0.000 | 0.045 | 0.043 | 0.000 | 0.059 | 0.000 | 0.000 | 0.000 | 0.042 | 0.000 | 0.000 | 0.000 | 0.000 | 0.130 |
| 35-603 | 0.000 | 0.000 | 0.087 | 0.000 | 0.000 | 0.000 | 0.000 | 0.000 | 0.000 | 0.000 | 0.043 | 0.000 | 0.059 | 0.000 | 0.000 | 0.000 | 0.000 | 0.000 | 0.000 | 0.000 | 0.000 | 0.174 |
| 35-609 | 0.000 | 0.045 | 0.000 | 0.000 | 0.000 | 0.048 | 0.000 | 0.000 | 0.227 | 0.045 | 0.087 | 0.000 | 0.000 | 0.000 | 0.000 | 0.050 | 0.000 | 0.000 | 0.000 | 0.000 | 0.000 | 0.000 |
| 35-617 | 0.000 | 0.045 | 0.000 | 0.000 | 0.000 | 0.000 | 0.000 | 0.000 | 0.045 | 0.000 | 0.000 | 0.000 | 0.000 | 0.000 | 0.000 | 0.000 | 0.000 | 0.000 | 0.000 | 0.000 | 0.000 | 0.000 |
| 35-640 | 0.000 | 0.000 | 0.000 | 0.000 | 0.000 | 0.000 | 0.000 | 0.000 | 0.000 | 0.000 | 0.000 | 0.000 | 0.000 | 0.000 | 0.000 | 0.000 | 0.042 | 0.000 | 0.000 | 0.000 | 0.000 | 0.000 |
| 35-655 | 0.682 | 0.000 | 0.000 | 0.000 | 0.429 | 0.190 | 0.000 | 0.263 | 0.000 | 0.091 | 0.174 | 0.350 | 0.647 | 0.769 | 0.750 | 0.100 | 0.750 | 0.522 | 0.000 | 0.000 | 0.778 | 0.000 |
| 35-668 | 0.045 | 0.045 | 0.522 | 0.000 | 0.000 | 0.476 | 0.000 | 0.421 | 0.455 | 0.545 | 0.522 | 0.000 | 0.000 | 0.000 | 0.000 | 0.600 | 0.083 | 0.043 | 0.000 | 0.667 | 0.000 | 0.652 |
| 35-675 | 0.000 | 0.000 | 0.304 | 0.000 | 0.000 | 0.048 | 0.000 | 0.053 | 0.091 | 0.227 | 0.217 | 0.000 | 0.000 | 0.000 | 0.000 | 0.250 | 0.000 | 0.000 | 0.000 | 0.125 | 0.056 | 0.391 |
| 35-679 | 0.000 | 0.000 | 0.261 | 0.000 | 0.000 | 0.000 | 0.000 | 0.000 | 0.000 | 0.727 | 0.565 | 0.000 | 0.000 | 0.000 | 0.000 | 0.000 | 0.000 | 0.000 | 0.000 | 0.042 | 0.056 | 0.000 |
| 35-690 | 0.000 | 0.045 | 0.000 | 0.000 | 0.000 | 0.000 | 0.000 | 0.000 | 0.000 | 0.000 | 0.000 | 0.000 | 0.000 | 0.000 | 0.000 | 0.000 | 0.000 | 0.000 | 0.000 | 0.000 | 0.000 | 0.000 |
| 35-860 | 0.000 | 0.000 | 0.000 | 0.000 | 0.000 | 0.000 | 0.000 | 0.000 | 0.000 | 0.000 | 0.000 | 0.050 | 0.000 | 0.000 | 0.000 | 0.000 | 0.000 | 0.000 | 0.000 | 0.000 | 0.000 | 0.000 |
| 35-893 | 0.000 | 0.045 | 0.000 | 0.000 | 0.000 | 0.000 | 0.048 | 0.000 | 0.000 | 0.000 | 0.000 | 0.000 | 0.000 | 0.000 | 0.000 | 0.000 | 0.000 | 0.000 | 0.000 | 0.000 | 0.000 | 0.000 |
